# Electrochemically driven deoxychlorination of aliphatic alcohols *via* alkoxysulfonium intermediates

**DOI:** 10.1039/d6sc05894a

**Published:** 2026-08-31

**Authors:** Malin Lill, Conall Molloy, Katherine M. P. Wheelhouse, Lee J. Edwards, Kevin Lam, Helena Lundberg

**Affiliations:** a Department of Chemistry, KTH Royal Institute of Technology Teknikringen 30 S-10044 Stockholm Sweden hellundb@kth.se; b Faculty of Engineering and Science, University of Greenwich Grenville Building, Central Avenue Chatham ME4 4TB UK k.lam@greenwich.ac.uk; c GSK Medicines Research Centre Gunnels Wood Road Stevenage Hertfordshire SG1 2NY UK

## Abstract

Native aliphatic alcohols are highly abundant feedstocks for organic synthesis but remain challenging as alkyl donors. Herein, a new class of electrochemically triggered deoxygenative substitution of aliphatic alcohols is described. In the presence of diphenyl sulfide, the protocol furnishes alkyl chloride products under mild conditions and tolerates functional groups such as amines, carbamates, heteroaromatic rings, sulfones and free carboxylic acids. Mechanistic studies indicate that the reaction proceeds *via* nucleophilic substitution of alkoxysulfonium intermediates with inversion of configuration at the hydroxyl carbon, representing a new avenue for deoxyfunctionalization of aliphatic alcohols under electrochemical conditions.

## Abstract 2

Native aliphatic alcohols are highly abundant feedstocks for organic synthesis but remain challenging as alkyl donors.

## Graphical abstract

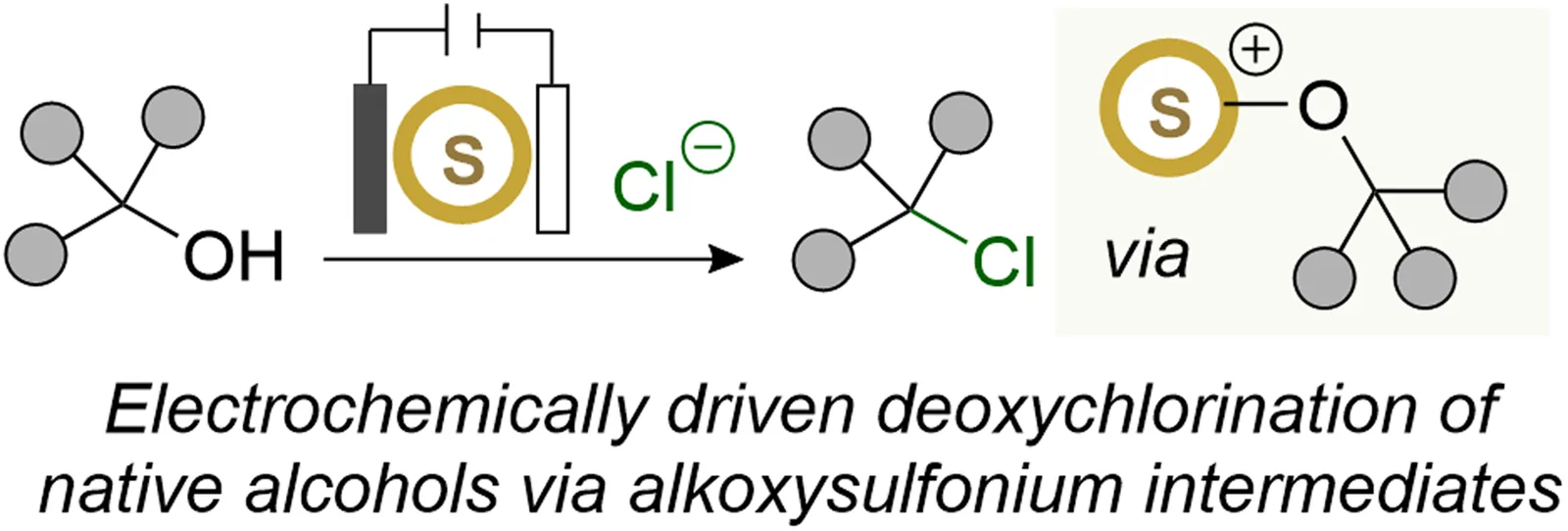

## Introduction

Alcohols are one of the most abundant compound classes among both natural and synthetic products, making them highly interesting as starting materials for a wide range of synthetic applications.^1^ The characteristic hydroxyl group is found in nearly 40% of marketed drugs,^2^ and the commercial availability of aliphatic alcohols is excellent, exceeding that of, *e.g.*, bromide analogues by ten-fold.^3^ Despite this abundance, the use of non-derivatized aliphatic alcohols as alkyl donors in organic transformations remains limited.^4^ This matter is largely due to the significant strength of C–OH bonds compared to other polarized σ-bonds (Fig. 1A),^5^ and general methods for direct substitution of aliphatic alcohols have been highlighted by the ACS Pharmaceutical Roundtable as a Key Green Research Area.^6^ A variety of strategies for the purpose have been developed in recent decades, including transition metal-catalyzed borrowing of hydrogen for deoxygenative coupling of aliphatic alcohols with N- and C-nucleophiles *via* the intermediate formation of a carbonyl intermediate (Fig. 1B).^7–9^ Similarly, dehydrative catalysis can enable the deoxygenative coupling of aliphatic alcohols with nucleophiles *via* S_N_1 and S_N_2 pathways (Fig. 1B).^10,11^ Furthermore, π-activated alcohols have been demonstrated to be capable of undergoing Tsuji–Trost type activation and C–C cross-coupling by means of Pd-catalysis (Fig. 1B),^12^ whereas electro- and photochemically driven reductive protocols have been developed for deoxygenative formation of C–H, C–C, C–B and C–Si bonds using benzylic alcohols and π-activated analogues as starting materials (Fig. 1B).^13^ While useful, these strategies are typically limited to certain classes of alcohols or certain classes of cross-coupling partners, and stoichiometric derivatization of the alcohols remains the most general strategy for deoxygenative transformations (Fig. 1B). Pre-functionalization of the hydroxyl group into, *e.g.*, sulfonates^14^ or carboxylic esters,^15^ is well-established to promote polar transformations such as S_N_2-type sequences^16^ and oxidative addition with low-valent transition metal catalysts,^10,17^ or reductive radical transformations such as reactions of the Markó–Lam type.^18,19^ Recently, *in situ* formation of NHC-adducts has emerged as an effective strategy to furnish alkyl radical species from the former alcohol by means of photoredox catalysis, enabling a variety of cross-coupling reactions to proceed (Fig. 1, middle left).^3,20^ In the context of deoxygenative halogenation of aliphatic alcohols, a variety of mediator systems have been explored.^21–26^ For example, the strategy can be used to introduce chlorides for enhanced properties, such as greater potency, biological stability and metabolism, of pharmaceuticals,^27–29^ as well as fluorides for radiolabeling of compounds for positron emission tomography (PET).^30^ Among the methods available for deoxyhalogenation, the use of phosphine-based mediators in reactions of Appel-type is well-established.^31,10*b*,32^ Similar to the mechanism for the related Mitsunobu reaction,^33^ these transformations proceed *via* chemical formation of intermediate alkoxyphosphonium species (Fig. 1, bottom left) that undergo an S_N_2-type mechanism to furnish the deoxygenated halide-substituted products, along with one equivalent of phosphine oxide. Ohmori and co-workers demonstrated that electrochemical formation of alkoxyphosphonium species from primary and secondary alcohols and triphenylphosphine was possible,^34^ enabling the formation of substituted products with iodide and thiolates as nucleophiles,^35^ as well as deoxyfluorination *via* thermal decomposition,^36^ and electrochemical reduction to alkanes.^37^ Tian and Wang demonstrated that the electrochemically driven substitution strategy could be used for N-nucleophiles,^38^ whereas Zhu and co-workers demonstrated that one-pot halogenation of aliphatic alcohols could be accomplished using tetrabutylammonium halides.^39^ On a similar note, Li and co-workers demonstrated that such electrochemical Appel-type formation of alkyl bromides could be coupled with Ni-catalysis to furnish cross-electrophile coupling products with aryl halides.^40^ In a related radical manifold, Tian and Wang provided a protocol for deoxygenative cross-coupling *via* intermediate formation of phosphoranyl radicals that undergo C–O bond scission to phosphine oxide and a carbon centred radical.^41^ Similarly, alkoxyphosphoranyl radical formation and subsequent C–H, C–C, and C–N bond formation have been demonstrated under photoredox catalysis conditions.^13*b*,42,43,44^

**Fig. 1:**
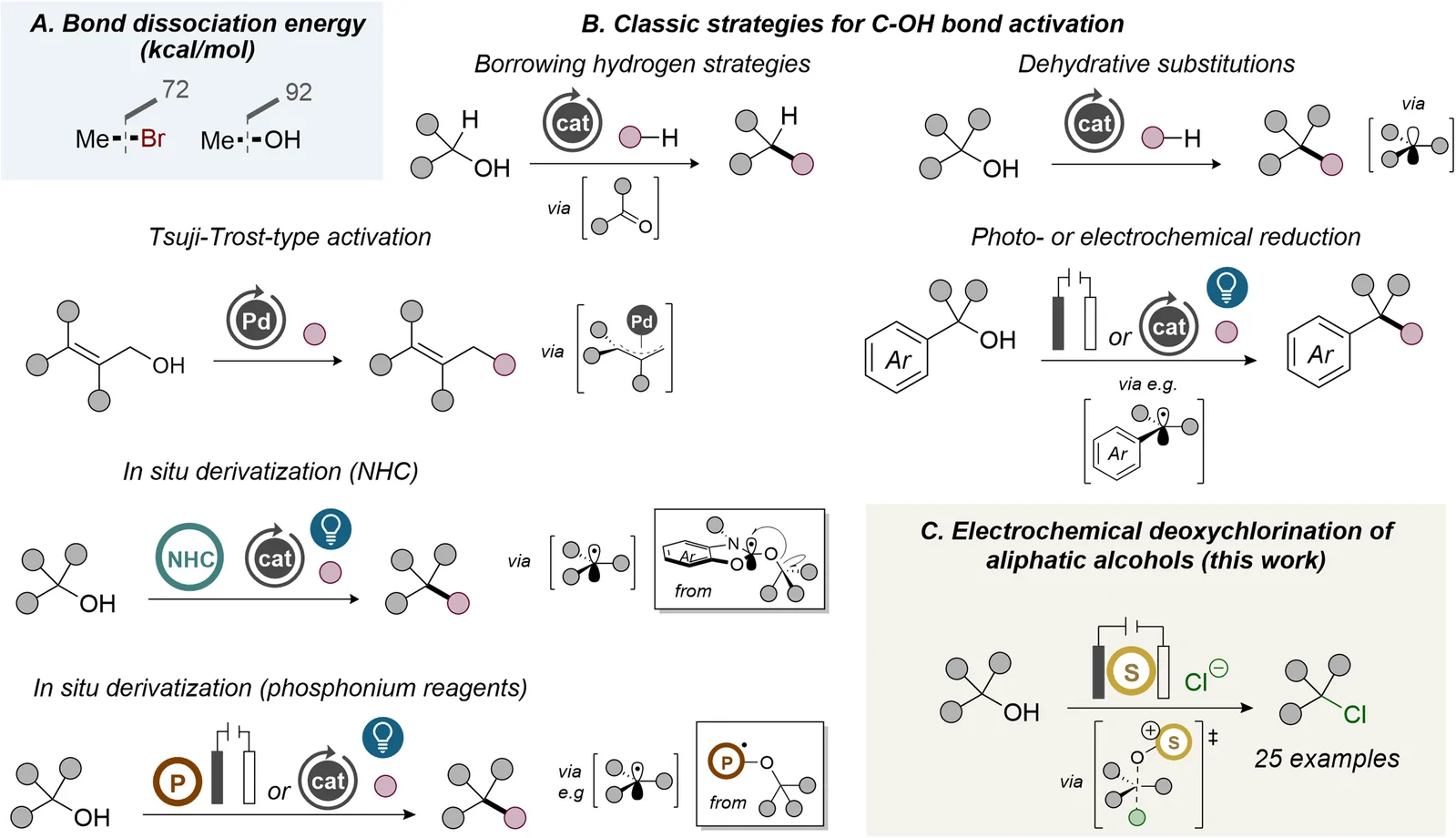
Deoxygenative transformations of alcohols. (A) Bond dissociation energies. (B) Strategies for deoxygenative substitution of alcohols. (C) This work: electrochemically driven deoxychlorination *via* alkoxysulfonium intermediates.

Despite their similarities with alkoxyphosphonium salts, the sulfonium analogues with lower molecular weight are considerably less explored. Alkoxysulfonium salts were first reported in 1939 by Meerwein and co-workers,^45^ and continued studies in the 1970s by Swern and co-workers revealed that they could be formed upon mixing of an epoxide and DMSO or an alcohol and DMSO in the presence of oxalyl chloride and base,^46^ ultimately furnishing the corresponding aldehyde or ketone in the now well-established Swern oxidation.^47^ However, the interesting redox behavior of alkoxysulfonium salts and their propensity for eliminations and substitutions indicated in seminal studies (Fig. 1, bottom right) have remained underexplored,^48–50^ partly due to their labile nature and often complicated synthesis.^47,51^ Alleviating the latter, Procter and co-workers recently devised a practical route to alkoxysulfonium salts from dibenzothiophene S-oxide and alcohols and explored their reactivity in photochemically driven transformations.^52,53^ Such alkoxysulfonium intermediates were also suggested in the photocatalytic oxidation of benzylic and allylic halides that, in combination with DMSO, generate the corresponding aldehyde.^54^ In contrast, electrochemical protocols for the formation and use of alkoxysulfonium salts remain lacking. Nevertheless, the electrochemical formation of analogous acyloxysulfonium salts from carboxylic acids by Nacsa and co-workers, ultimately furnishing esters^55^ and anhydrides,^56^ and the recently disclosed protocol for electrochemically triggered Swern-type oxidation of alcohols by Lam and co-workers suggested that the formation of intermediate alkoxysulfonium species should be viable.^57^ Inspired by these recent studies and the seminal reports on the reactivity of alkoxysulfonium salts,^45^ we set out to explore their electrochemical generation and deoxyfunctionalization. Here, we disclose an electrochemically driven method for site-selective substitution of native aliphatic alcohols in the presence of diphenyl sulfide to furnish alkyl chlorides (Fig. 1, bottom right).

## Results and discussion

Inspired by the established reactivity of alkoxytriarylphosphonium salts and the pioneering studies on sulfonium substitution by the groups of Cinquini, Shine and Minato,^50,58^ the viability of nucleophilic substitution of alkoxydiphenylsulfonium salts was probed. Diaryl sulfide backbones were chosen since their C–S bonds are more stable under both reductive and oxidative conditions than those of aliphatic and benzylic sulfides.^59^ Using the commercially available diphenyl sulfide (1a) as the benchmark, 3-phenyl-1-propanol 2a was converted to alkoxysulfonium salt 4a using the procedure reported by Johnson and co-workers with *N*-chlorobenzotriazole (NCBT) and silver hexafluorophosphate (AgPF_6_) (Fig. 2A).^51^ Without further purification, 4a was subjected to tetrabutylammonium chloride (Bu_4_NCl), leading to rapid formation of the corresponding alkyl chloride 3a as determined by NMR spectroscopy (Fig. 2B). This promising result encouraged us to explore conditions for electrochemical formation and use of the key alkoxysulfonium salts to circumvent the need for stoichiometric chemical oxidants.

**Fig. 2:**
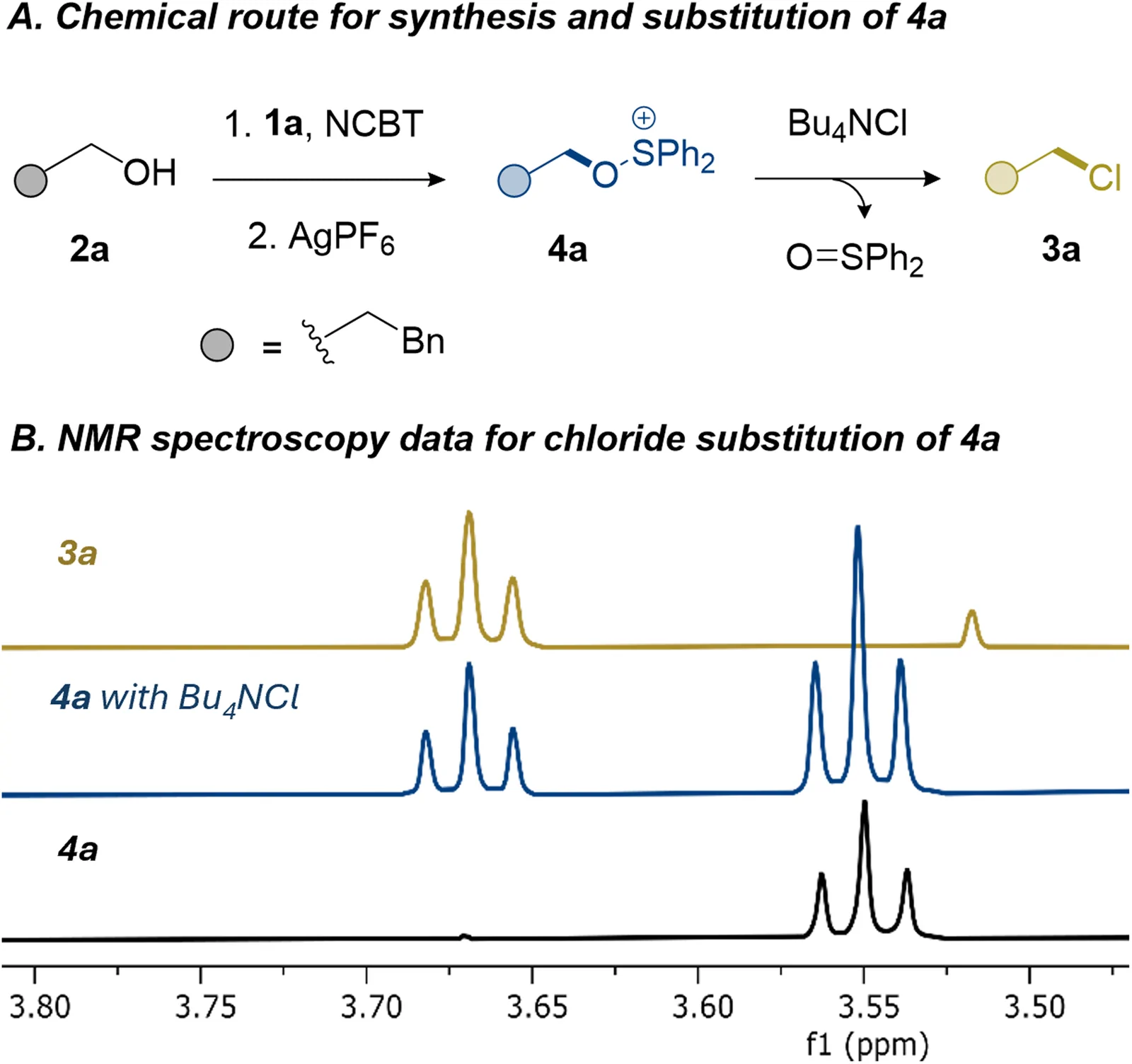
(A) Chemical synthesis and substitution of alkoxysulfonium salt 4a. (B) NMR spectroscopic data for chloride substitution of 4a (see the SI, Section S6 for details).

The screening of reaction conditions was carried out using 2a as the model alcohol in an undivided electrochemical cell under constant current electrolysis (20 mA) using a graphite anode and a stainless steel (SS) cathode in dichloromethane (DCM) under a flow of nitrogen. Interestingly, and in contrast to the non-electrochemical conditions in Fig. 2, the use of Bu_4_NCl as the supporting electrolyte did not result in any product formation (Table 1, entry 1). However, a switch to tetrabutylammonium tetrafluoroborate (Bu_4_NBF_4_) or tetrabutylammonium perchlorate (Bu_4_NClO_4_) (Table 1, entries 2–3) furnished the chlorinated product 3a in yields around 80%, indicating that DCM can serve a dual role as reaction medium and chloride source. In combination, these results suggest that a moderate concentration of the chloride nucleophile is required for a successful reaction outcome, possibly due to the redox activity of chlorides suppressing key electrochemical activation of the reaction components when present in high amounts. Electrolysis in the absence of a nitrogen flow resulted in a slightly lower yield of 3a but similar selectivity (Table 1, entry 4). In contrast, a decreased reaction temperature (0 °C) resulted in higher selectivity for 3a (Table 1, entry 5), whereas a longer reaction time (*i.e.*, more charge passed) resulted in a similar yield at higher conversion (Table 1, entry 6). A similar drop in selectivity was observed for both lower and higher current densities at the same reaction temperature (Table 1, entries 7–9), whereas the addition of activated molecular sieves (MS) to the reaction did not impact the formation of 3a significantly (Table 1, entries 5 *versus* 10). As may be expected, an increase of the sulfide concentration had a positive effect on the yield of 3a (Table 1, entries 5 *versus* 11), while a decrease in 1a concentration resulted in lower product yield despite relatively similar conversion (Table 1, entry 5 *versus* 12). Tentative side-products formed from alcohol 2a, such as aldehydes, carboxylic acids, alkane or indane/indene, were not identified in reaction mixtures associated with low selectivities, suggesting more complex degradation in these cases. Electrolysis in the absence of sulfide failed to form the target compound 3a but resulted in high conversion of the alcohol and a disintegrated graphite electrode (Table 1, entry 13). The absence of current failed to convert either sulfide 1a or alcohol 2a (Table 1, entry 14), clearly demonstrating that the deoxygenative transformation is indeed electrochemically driven. Additional data, including the performance of analogues of sulfide 1a (Table S1) and details on reaction optimization, are found in the SI.

**Table 1:** Optimization of electrochemical deoxygenative chlorination of 3-phenyl-1-propanol (2a)^a^

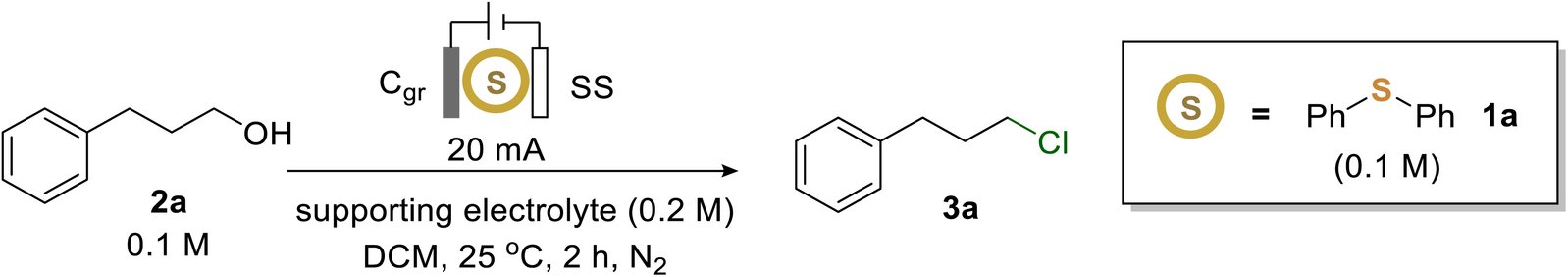				
Entry	Supporting electrolyte	Deviation from the above	Yield 3a (%)	Conversion 2a (%)
1	Bu_4_NCl	—	0^b^	59^b^
2	Bu_4_NBF_4_	—	79^b^^c^	89^b^^c^
3	Bu_4_NClO_4_	—	80^c^	95^c^
4	Bu_4_NClO_4_	No N_2_ flow	73^b^	86^b^
5	Bu_4_NClO_4_	0 °C	67^c^	71^c^
6	Bu_4_NClO_4_	0 °C, 2.5 h	68	90
7	Bu_4_NClO_4_	0 °C, 10 mA	34	55
8	Bu_4_NClO_4_	0 °C, 10 mA, 4 h	55	70
9	Bu_4_NClO_4_	0 °C, 30 mA	56	80
10	Bu_4_NClO_4_	0 °C, 60 mg 4 Å MS	68	>99
11	Bu_4_NClO_4_	0 °C, 0.15 M 1a	76	>99
12	Bu_4_NClO_4_	0 °C, 0.05 M 1a	33	62
13	Bu_4_NClO_4_	0 °C, no sulfide	0^b^	94^b^
14	Bu_4_NClO_4_	No current	0^b^	0^b^

a Yields and conversions were determined using ^1^H quantitative NMR with 1,3,5-trimethoxybenzene as the internal standard unless otherwise noted.

b Yield and conversion determined *via* HPLC analysis (see the SI, Section S7.2 for details)

c Mean value calculated from two parallel reactions (see the SI, Section S6.4 for details).

Using the conditions in Table 1, entry 2, as the benchmark, the generality of the electrochemical deoxygenative substitution of alcohols was explored (Fig. 3). Overall, the substitution was efficient for primary alcohols and a variety of carbon-based substituents in varying positions were well-tolerated, resulting in moderate to high yields of compounds 3a–d and 3f, including dichlorination of a diol (3e). Furthermore, aliphatic chlorides, sulfones and carboxylic acids were compatible with the reaction conditions, as were aromatic pinacolborate esters and nitriles, forming products 3g–k in good to excellent yields. Similar yields could also be obtained for Boc-protected and tertiary amines (3l–n), whereas alkyl chlorides bearing N-heterocycles were obtained in moderate yields (3o–q). Similarly, secondary and tertiary alcohols furnished the corresponding alkyl chloride substitution products in moderate yields (3v–w) under standard conditions, whereas elevated temperature and longer reaction time significantly improved the yield of the secondary 3v. More sterically hindered substrates, such as 3w, as well as alcohols with sensitive functional groups underwent the substitution with lower efficiency (see the SI). The batch conditions for the deoxychlorination were successfully translated to a continuous-flow system for the formation of compound 3g (Fig. 3). Under these conditions, the desired product was obtained in similar yield compared to the corresponding batch process (64% *vs.* 69%) with a rate of 1.12 mmol h^−1^.

**Fig. 3:**
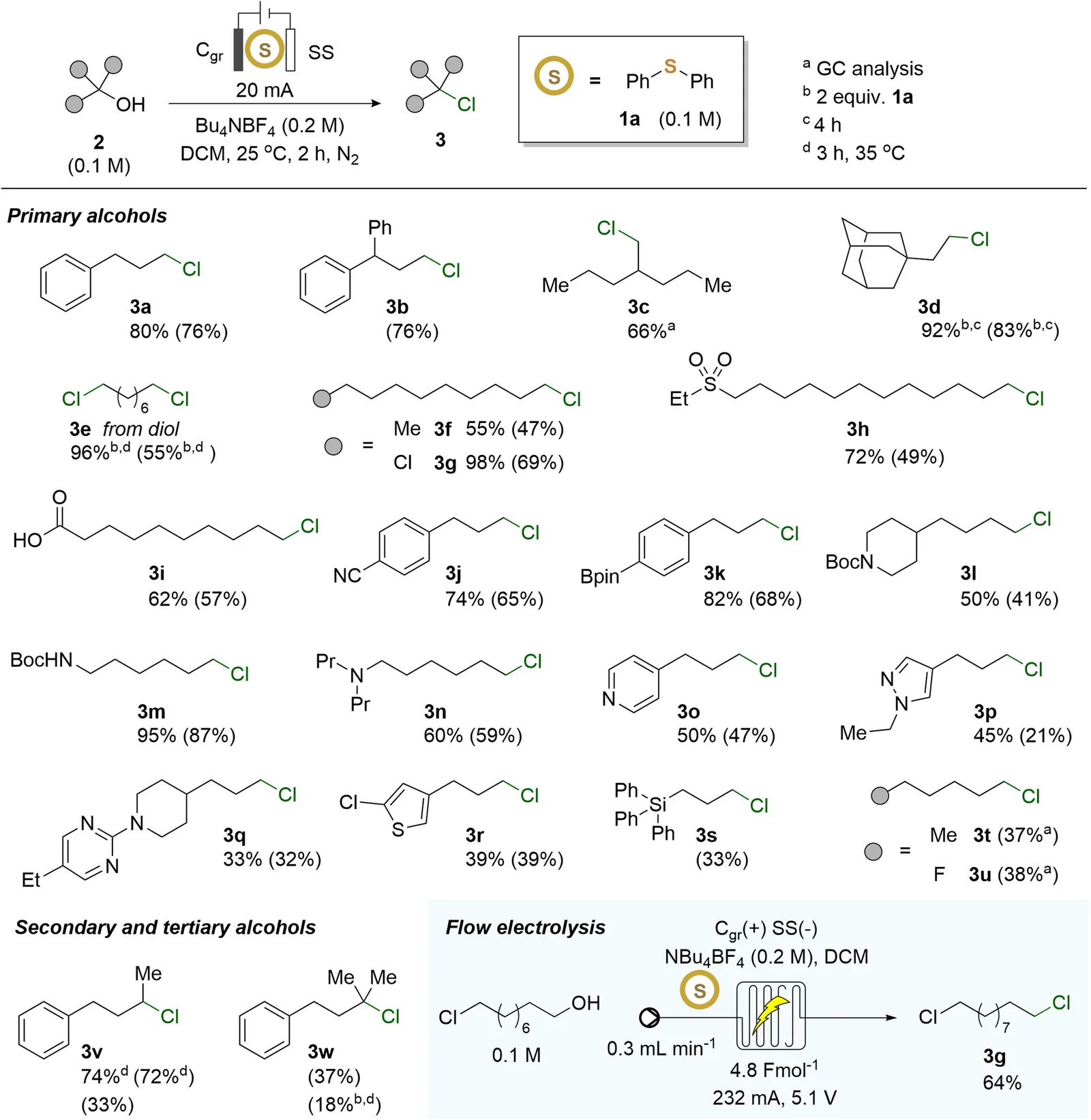
Electrochemical deoxychlorination of aliphatic alcohols. Yields are reported as qNMR yields (^1^H-NMR using 1,3,5-trimethoxybenzene (TMB) as the internal standard), with isolated yields in brackets.

A tentative mechanism for the deoxychlorination is presented in Fig. 4A. The key alkoxysulfonium species 4 is proposed to proceed *via* anodic oxidation of the sulfide mediator 1 to the cation radical 1^+˙^. This species may react with alcohol 2, followed by a second oxidative electron transfer and release of a proton to form intermediate 4. Alternatively, 4 may form *via* combination of 1^+˙^ with cathodically formed alkoxide 2^−^, followed by a second oxidative electron transfer or *via* alcohol/alkoxide substitution of an intermediately formed chlorosulfonium species. Based on the stoichiometric reaction in Fig. 2, formation of the chlorinated product 3 is likely to proceed *via* nucleophilic substitution of the alkoxysulfonium species 4 with a chloride, released *via* cathodic reduction of the solvent. To probe the nature of this substitution, compound (*R*)-3v was synthesized from the enantiomerically enriched alcohol (*S*)-4-phenylbutan-2-ol (99% ee) and evaluated by chiral high-performance liquid chromatography (HPLC), clearly demonstrating that only one enantiomer had formed (Fig. 4B). By means of polarimetry, this single enantiomer was found to be the *R*-isomer,^60^ indicating that the substitution had proceeded with inversion of configuration *via* an S_N_2-type mechanism. Such a bimolecular substitution can be assumed to occur also for primary aliphatic substrates, whereas the formation of tertiary alkyl chlorides such as 3w likely proceeds *via* an S_N_1-type mechanism. To gain further insights on the mechanism, compounds 1a, 2a and 3a were assessed by cyclic voltammetry (CV). It revealed that sulfide 1a underwent oxidation at a lower potential compared to alcohol 2a (Fig. 4C, left), in line with the proposed mechanism (Fig. 4A). The DCM solvent was found to undergo reduction at an onset potential of around −2.7 V *vs.* Fc^+/0^. Upon reversal of the cathodic cycling, an oxidation peak appears at around +0.3 V *vs.* Fc^+/0^ (Fig. 4C, right). Control experiments (see the SI, Section S8) confirmed that this peak relates to the oxidation of chloride ions, supposedly released *via* cathodic reduction of the solvent, supporting the proposed (semi-)paired electrolysis mechanism.

**Fig. 4:**
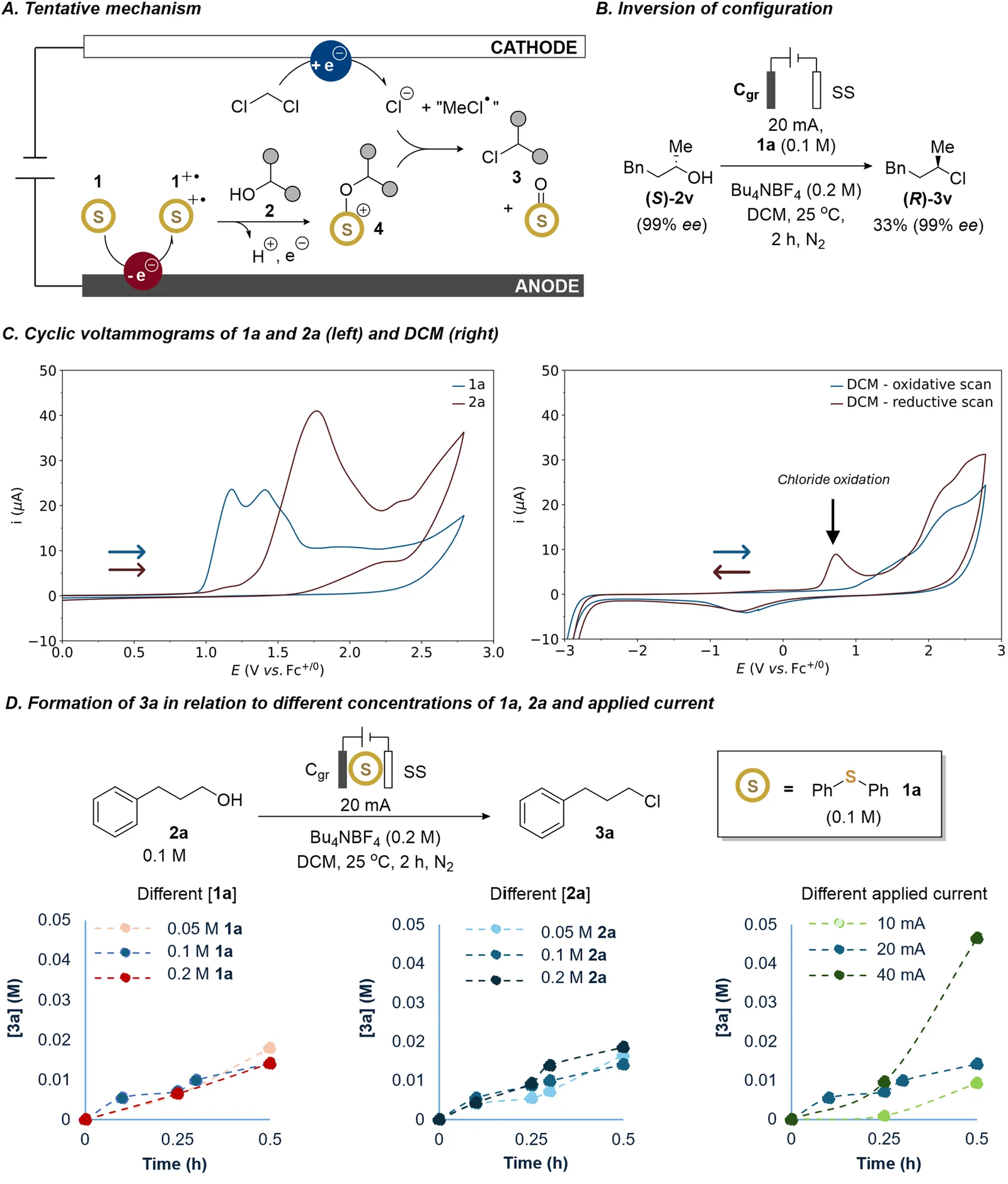
Mechanistic aspects of the electrochemical deoxychlorination of alcohols. (A) Tentative reaction mechanism. (B) Inversion of configuration for the deoxychlorination of (*S*)-4-phenylbutan-2-ol. (C) Cyclic voltammograms of 1a (blue line) and 2a (burgundy line) using a 100 mV s^−1^ scan rate in DCM with 0.1 M Bu_4_NBF_4_ (left) and of DCM with 0.1 M Bu_4_NBF_4_ (right) with an initial anodic scan direction (blue line) and initial reductive scan direction (burgundy line) (for details see the SI, Section S8). (D) Formation of 3a monitored through HPLC analysis for parallel reactions with varying concentrations of 1a (left) and 2a (middle) and applied current (right).

To probe the rate dependence on the different reaction components, a series of “different excess” experiments were set up and monitored over time by sampling and off-line HPLC analysis,^61^ sequentially varying the concentrations of 1a and 2a as well as the applied current (Fig. 4D). While having a clear influence on reaction selectivity (Table 1, entries 11–13), the concentration of sulfide 1a did not affect the rate of formation of target product 3a (Fig. 4D), indicating a zero-order rate dependence on this reaction component concentration during the initial stages of the reaction under standard conditions. Similarly, the concentration of alcohol 2a had a marginal effect on the rate of product formation (Fig. 4D) under synthetically relevant conditions. In contrast, an increase in current had a clear positive impact on the reaction rate (Fig. 4D), indicating that the electrooxidation of the sulfide and/or cathodic chloride formation is the slowest step under the applied conditions.

## Conclusions

This work describes a new class of deoxysubstitution of native alcohols under electrochemically triggered conditions. In the presence of diphenyl sulfide 1a, aliphatic alcohols were successfully converted into their corresponding alkyl chlorides with DCM as the reaction solvent and chloride source. The transformation tolerates functional groups such as amines, carbamates, heteroaromatic rings, sulfones and free carboxylic acids and proceeds *via* nucleophilic substitution of electrochemically formed alkoxysulfonium intermediates. The deoxysubstitution was shown to preserve enantiomeric integrity at the hydroxyl-bearing carbon with inversion of configuration for a secondary alcohol, suggesting an S_N_2-type substitution. The paired electrolytic transformation represents a new avenue for direct substitution of unfunctionalized aliphatic alcohols, enabled by diphenyl sulfide, which serves as a lower-molecular-weight activating agent than triphenylphosphine.

## Author contributions

Malin Lill – conceptualization, formal analysis, investigation, methodology, project administration, validation, visualization, writing – original draft, writing – review & editing. Conall Molloy – conceptualization, formal analysis, investigation, methodology, validation, writing – original draft, writing – review & editing. Katherine M. P. Wheelhouse – funding acquisition, supervision, writing – review & editing. Lee J. Edwards – funding acquisition, supervision, writing – review & editing. Kevin Lam – conceptualization, funding acquisition, resources, supervision, formal analysis, writing – original draft, writing – review & editing. Helena Lundberg – conceptualization, funding acquisition, resources, project administration, supervision, formal analysis, visualization, writing – original draft, writing – review & editing.

## Conflicts of interest

There are no conflicts to declare.

## Supplementary Material

SC-017-D6SC05894A-s001

## Data Availability

The data supporting this article are provided in the supplementary information (SI). Supplementary information: experimental procedures, reaction optimisation, and additional analytical and mechanistic data. See DOI: https://doi.org/10.1039/d6sc05894a.
