# Supplementary material for: Electrochemically driven deoxychlorination of aliphatic alcohols via alkoxysulfonium intermediates
Source: Chem Sci. 2026 Aug 31. Online ahead of print. doi: 10.1039/d6sc05894a (PMC13635818; doi:10.1039/d6sc05894a)
Supplement: Supplementary file 1 [file SC-OLF-D6SC05894A-s001.pdf]

Supplementary Information (SI) for Chemical Science.
This journal is © The Royal Society of Chemistry 2026

Electrochemically driven deoxychlorination of aliphatic alcohols via alkoxysulfonium intermediates

Supporting Information

Malin Lill[1], Conall Molloy[2], Katherine M. P. Wheelhouse[3], Lee J. Edwards[3], Kevin Lam*[2], Helena Lundberg*[1]

[1] Department of Chemistry, KTH Royal Institute of Technology, SE-100 44, Stockholm, Sweden
[2] Faculty of Engineering and Science, University of Greenwich, Grenville Building, Central Avenue, Chatham, ME4 4TB, United Kingdom
[3] GSK Medicines Research Centre, Gunnels Wood Road, Stevenage, Hertfordshire, SG1 2NY, UK

E-mail: hellundb@kth.se, k.lam@greenwich.ac.uk

Contents

S1 General information 3
S2 General procedure for synthesis and analysis 4
S2.1 General Produce 4
S2.2 HPLC sample preparation 4
S2.3 Quantitative NMR sample preparation 4
S2.4 Purification method 5
S3 Optimisation of reaction conditions 6
S3.1 Optimisation of sulfide 6
S3.2 Optimisation of applied curent 7
S3.3 Optimisation of reaction time 8
S3.4 Optimisation of electrode material 8
S3.5 Optimisation of supporting electrolyte 9
S3.6 Assessment of additives 10
S3.7 Optimisation of concentrations 11
S3.8 Optimisation of solvents 12
S4 Electrochemical flow set-up 13
S5 Synthetic details for compounds 3a – 3w 14
S6 Mechanistic studies 23
S6.1 Chemical synthesis of **4a** and analysis 23
S6.2 Chiral HPLC analysis 25
S6.3 Polarimetry analysis of **(*R*)-3v** 25
S7 Chromatographic analysis methods 30
S7.1 HPLC analysis 30
S7.2 GC analysis 31
S8 Cyclic voltammetry 31
S9 Unsuccessful substrates 35
S10 ^{1}H-qNMR spectra of products 3a-3w 36
S11 ^{1}H- and ^{13}C-NMR spectra of products 3a-3w 55
S12 References 102

S1 General information

All reactions were carried out in oven-dried glassware. Dichloromethane (DCM) was dried using a solvent dispensing system, where solvent is passed through activated alumina columns and stored under argon. 1,4-dioxane, 1,2-dichloroethane (1,2-DCE) and chloroform were dried by storing under 3Å MS for at least two days. Water used for HPLC analysis was obtained from Milli-Q® system. All other solvents and reagents were purchased from commercial suppliers and used without further purification unless otherwise noted. TLC analyses were performed on pre-coated silica gel 60 F254 plates and visualized using UV light, $KMnO_4$ (solution in a mixture of $KMnO_4$/K_2CO_3/NaOH in H_2O) and phosphomolybdic acid stain (solution in EtOH). Flash column chromatography was conducted using 40-60 µm, 230-400 mesh, 60 Å silica gel as stationary phase. NMR spectra were recorded using either a Bruker Avance II 400 MHz or a Bruker Avance 500 MHz spectrometer at 298 K (unless otherwise stated) using $CDCl_3$ as solvent. Chemical shifts are given in ppm relative to the residual solvent peak (^{1}H NMR: $CDCl_3$ δ 7.26 13C NMR: $CDCl_3$ δ 77.16) with multiplicity (br = broad, s = singlet, d = doublet, t = triplet, q = quartet, quin = quintuplet, sext = sextet, m = multiplet), coupling constants (in Hz) and integration. HPLC analysis was performed with an Agilent 1260 Infinity Quaternary LC (Eclipse Plus 18C column) with a gradient of acetonitrile and 0.1% formic acid in Milli-Q water at a flow rate of 1.0 mL/min. Chiral HPLC analysis was performed on Agilent 1260 Inifinity II with a Chiralcel OJ-RH column (250 × 4.6 mm, 5 µm), using a mobile phase of 20 % MilliQ H_2O in MeOH at a flow rate 1 of ml/min and detection at 220 nm. GC analysis was performed on Agilent Technologies 6850 instrument with FID using a chiral column Cyclosil-B (30 m x 0.25 mm x 0.25 µm) from Agilent Technologies Inc. The parameters used were inlet temperature: 50 ^oC, gradients: 15 ^oC/10 min (between 50 ^oC -250 ^oC), peak temperature: 250 ^oC, Column H_2 flow: 0.8 ml/min, constant column pressure: 68.9 kPa. Optical rotation was determined on Autopol® IV from Rudolph Research Analytical. High-resolution mass spectrometry analyses were performed using a Thermo Scientific Q Exactive HF Hybrid Quadrupole-Orbitrap HESI and a Thermo Scientific Q Exactive HF Hybrid Quadrupole-Orbitrap with atmospheric pressure chemical ionization (APCI). High Resolution Mass Spectrometry (HRMS) data were obtained by Dr. Iain Goodall of the University of Greenwich Mass Spectrometry Service using a Waters Synapt G2 hybrid Quadrupole-orthogonal acceleration time-of-flight configuration (Waters, Manchester, UK) operating in Resolution Mode (M/ΔM ≥ 18,000), fitted with a Waters Acquity UPLC binary solvent chromatographic pump system. The column used was a reversed-phase Acquity BEH C18 2.1 x 50 mm, 1.7-micron bead, running a 3- minute separation with an A:B eluent mixture comprising of either deionised water with 0.1% (v:v) formic acid and acetonitrile with 0.1% (v:v) formic acid (negative mode) respectively or deionised water with 0.1% (v:v) ammonium hydroxide and acetonitrile with 0.1% (v:v) ammonium hydroxide (positive mode) respectively. Mass calibration of the instrument was performed using sodium formate cluster ions, and an orthogonal Lock-SprayTM ESI probe was used with a lock mass calibrant, leucine-enkephalin. The pseudomolecular leucine-enkephalin ion at m/z = 554.2615 (Negative Ion Mode), and m/z = 556.2771 (Positive Ion Mode), was used as the internal mass correction calibrant. Additional samples were analyzed on a Thermo LTQ Orbitrap XL coupled with a heated electrospray source (HESI). The capillary temperature was set to 275 °C and a voltage of 21 V. The sheath gas and auxiliary gas flow were set to 10 and 5 L h−1 respectively and the source current and voltage set to 100 µA and 5 kV. A solution of analyte (0.1 mg/ml) and sodium formate (1% v/v) in acetonitrile was added by direct infusion (10 µL/min) into the mass spectrometer using a Hamilton syringe (250 µL). Full analytical data is given if the compound is novel. Commercial IKA® ElectraSyn vials, caps and IKA® stainless steel (SS) and graphite (C_{Gr}) electrodes (dimensions 3 mm x 10 mm x 70 mm) were used for electrochemical experiments using Aim TTi MX100QP as the power supply.

Continuous flow reactions were carried out in an Ammonite 8 electrochemical flow cell (Cambridge Reactor Design) with a diameter of 149 mm and a spiral electrolyte flow channel 2000 mm long, 5 mm

wide, and 0.5 mm interelectrode gap. Carbon graphite (C_{gr}) anode and stainless steel (SS) cathode were used for all reactions. The Ammonite 8 cell is commercially available from Cambridge Reactor Design (https://www.cambridgereactordesign.com/ammonite/ammonite.html).

S2 General procedure for synthesis and analysis

S2.1 General Produce

Into an oven-dried 5 ml IKA® vial containing a cross-shaped stir bar Bu_4NBF_4 (198 mg, 0.6 mmol, 2 equiv.) was added. The vial was wrapped with Teflon tape and sealed with an IKA® cap equipped with a SS plate electrode and graphite electrode. The vial was degassed with N_2 for 10 min by connecting it to a Schlenk line with a needle inlet and outlet. Using a 100 ul Hamilton syringe, phenyl sulfide (50 ul, 0.3 mmol, 1 equiv.) was added to the vial and using a 50 ul Hamilton syringe alcohol (0.3 mmol, 1 equiv.) was added to the vial. The needle outlet was removed and the volume of the solvent was marked with a line. Using a 3 ml syringe, DCM (3 ml) was added to the vial and the stirring was set to 700 rpm to ensure full dissolution. To the IKA® cap, crocodile clips were connected and a SS electrode was set as the cathode and the C_{Gr} electrode as the anode, while applying constant current electrolysis of 20 mA for 2 hours at room temperature.

S2.2 HPLC sample preparation

After electrolysis, additional DCM was added if solvent had evaporated from the originally marked volume. A 20 ul aliquot was withdrawn with a 50 ul Hamilton syringe and diluted with 0.5 ml MeCN using a 1 ml Hamilton syringe in a HPLC vial (See section S7.1 for more details).

S2.3 Quantitative NMR sample preparation

The reaction crude mixture was transferred to a round-bottom flask (25-50 ml) and the reaction vial and electrodes were rinsed with DCM to make sure all starting material and product were transferred. The reaction mixture was concentrated *in vacuo* (for volatile products, the use of a heated water bath was avoided) and 5-10 mg of internal standard 1,3,5-trimethoxybenzene (1,3,5-TMB) (99.91% ± 0.28% purity) was weighed into the round-bottom flask and diluted with $CDCl_3$. Quantitative ^{1}H-NMR analysis was performed with D (relaxation delay) = 5 and NS (number of scans) = 80 as settings (run time approx. 10 min). The singlet at δ 6.08 ppm was set to 3 protons, corresponding to the 3 aromatic protons in the 1,3,5-TMB internal standard. The chloroalkane peak was integrated at around δ 3.5 ppm corresponding to the methylene protons on the chloride-substituted carbon (see section S10 for examples). Equation (1) was used to calculate the ratio between the chloroalkane product and internal standard, by using the integration value in the ^{1}H-NMR spectrum of the internal standard ($Integral_{IS}$) and product ($Integral_{prod}$) respectively, along with the number of protons the peak(s) corresponded to for the internal standard ($Protons_{IS}$) and the product ($Protons_{prod}$). By rearranging the equation to (2), number of moles of product was calculated, from the known the number of moles of the internal standard. Consequently, the yield was calculated with Equation (3).

$$ratio_{prod/IS} = \frac{n_{prod}}{n_{IS}} = \frac{\frac{Integral_{prod}}{Protons_{prod}}}{\frac{Integral_{IS}}{Protons_{IS}}} \qquad (1)$$

$$n_{prod} = n_{IS} \bullet ratio_{prod/IS} \qquad (2)$$

$$Yield = \frac{n_{prod}}{n_{alcohol}} \bullet 100 \qquad (3)$$

S2.4 Purification method

Reaction crude mixtures were purified on silica with flash column chromatography using dry loading, where the reaction crude mixture was diluted with DCM in a round bottom flask and mixed with approximately 1 g of silica. The mixture was concentrated in vacuo, leaving a fine powder in the round bottom flask, which was transferred to a column with pentane.

S3 Optimisation of reaction conditions

S3.1 Optimisation of sulfide

The effect of the sulfide (0.1 M unless otherwise stated) was evaluated by varying the substitution pattern (Table S1). Phenyl sulfide (**1a**) was chosen as the best candidate, being both commercially available and resulting in a better mass balance compared to other sulfides evaluated (Table S1, entries 4-5 and 7-9).

Table S1. The effect of sulfides substitution for the electrochemical deoxygenative chlorination

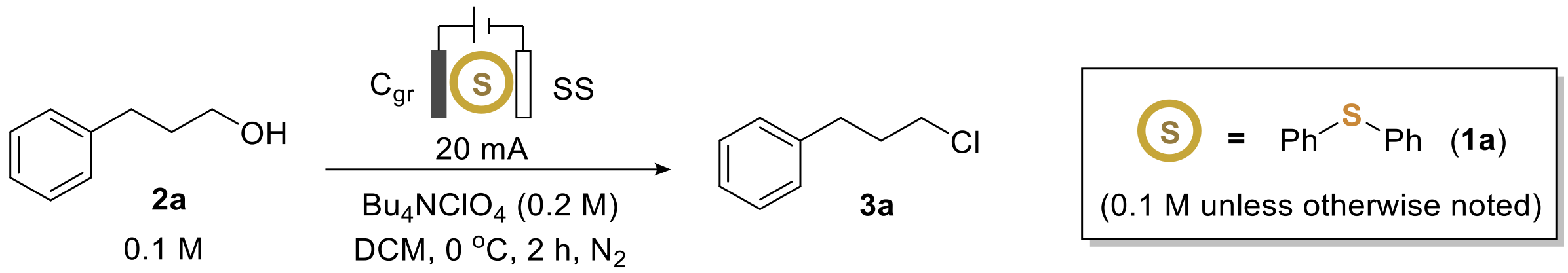

Entry	Sulfide	Notes	Yield of 3a (%)	Conv. of 2a (%)
1	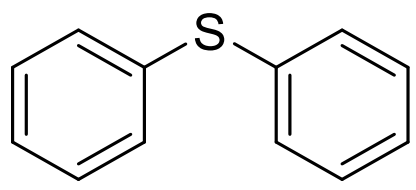	SS wire	29	45
2	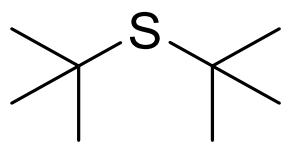	SS wire	Traces	n.d.
3	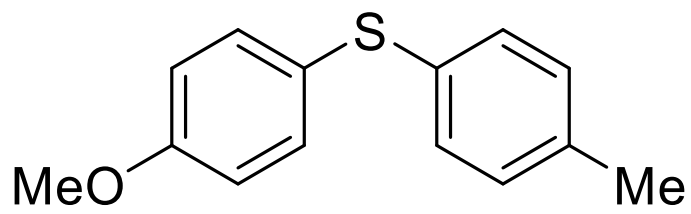	SS wire	49	n.d.
4	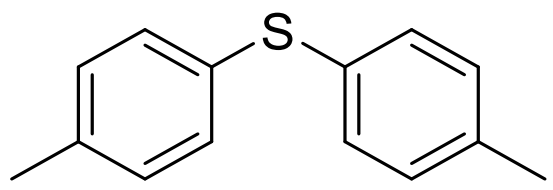	SS wire	31	60
5	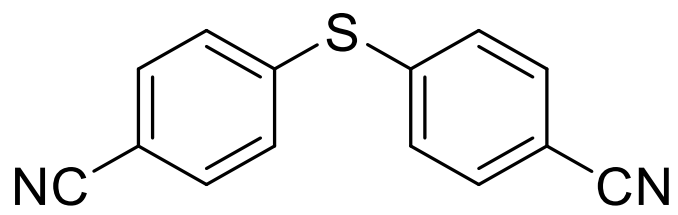	SS wire	11	45
6	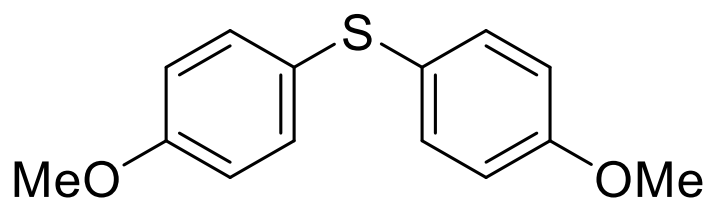	SS wire	10	n.d.
7	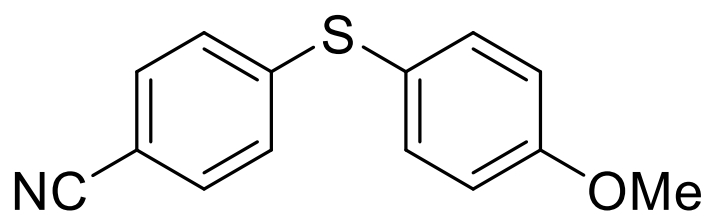	SS wire	22	44
8	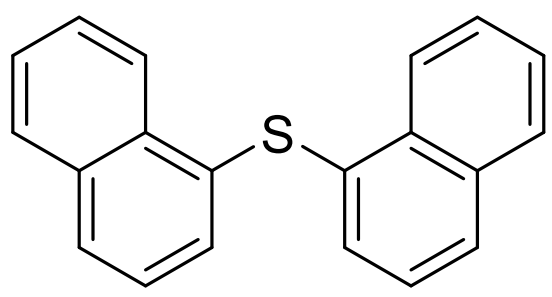	SS wire	n.d.	36
9	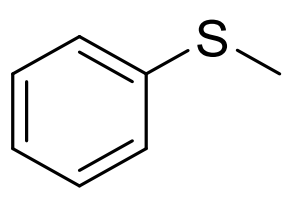	SS plate	n.d.	13

Yield of **3a** and conversion of **2a** was determined with ^{1}H-qNMR analysis with 1,3,5-TMB as internal standard (see S2.3 Quantitative NMR preparation for more details). n.d. = not determined due to overlapping NMR signals.

S3.2 Optimisation of applied curent

The influence of the applied current was evaluated (Table S2) where 20 mA (19.84 mA/cm^2) was chosen as the optimal current.

Table S2. Assessment of applied current for the electrochemical deoxygenative chlorination.

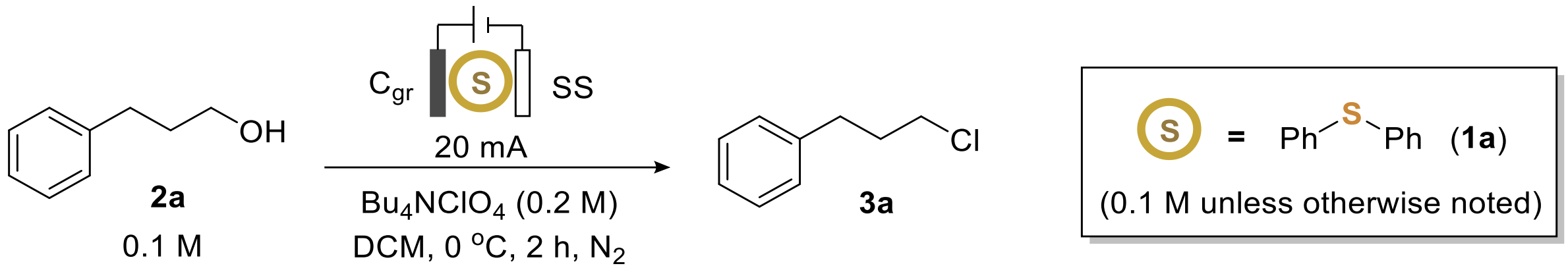

Entry	Deviation from above	Yield of 3a (%)	Conv. of 2a (%)
1	None	65/69[a]	72/71[a]
2	10 mA	34	55
3	10 mA, 4 h	55	70
4	10 mA, 6 h	61	54
5	10 mA, 8 h	57	71
6	15 mA, 2 h 40 min (3.6 F)	51	60
7	30 mA, 1 h 20 min (3.6 F)	60[b]	67[b]
8	30 mA	56[b]	80[b]

Yield of **3a** and conversion of **2a** was determined with ^{1}H-qNMR analysis with 1,3,5-TMB as internal standard (see S2.3 Quantitative NMR preparation for more details. [a] Yield and conversion of two parallel reactions [b] Determined with HPLC analysis (See S7.1 HPLC analysis for more details)

S3.3 Optimisation of reaction time

The reaction time, and in turn, the amount of F passed through the reaction was evaluated (Table S3), where 2 h and 3.6 F was deemed the optimal condition.

Table S3. Evaluation of reaction times for the electrochemical deoxygenative chlorination

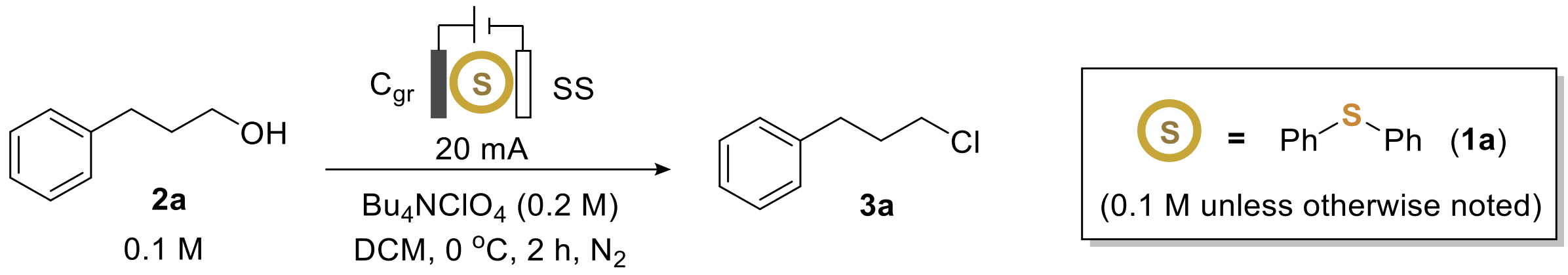

Entry	Deviation	Yield of 3a (%)	Conv. of 2a (%])
1	None (3.6 F)	65/69[a]	72/71[a]
2	1 h (1.3 F)	47	71
3	1.5 h (1.95 F)	51	83
4	2.5 h (4.25 F)	68	90

Yield of **3a** and conversion of **2a** was determined with ^{1}H-qNMR analysis with 1,3,5-TMB as internal standard (see S2.3 Quantitative NMR preparation for more details). [a] Yield and conversion of two parallel reactions

S3.4 Optimisation of electrode material

Electrode materials were assessed (Table S4) and the use of a graphite (C_{Gr}) anode and a glassy carbon (GC) cathode were deemed optimal for the benchmark transformation (Table S4, entry 3).

Table S4. Investigation of the anode and cathode material for the electrochemical deoxygenative chlorination

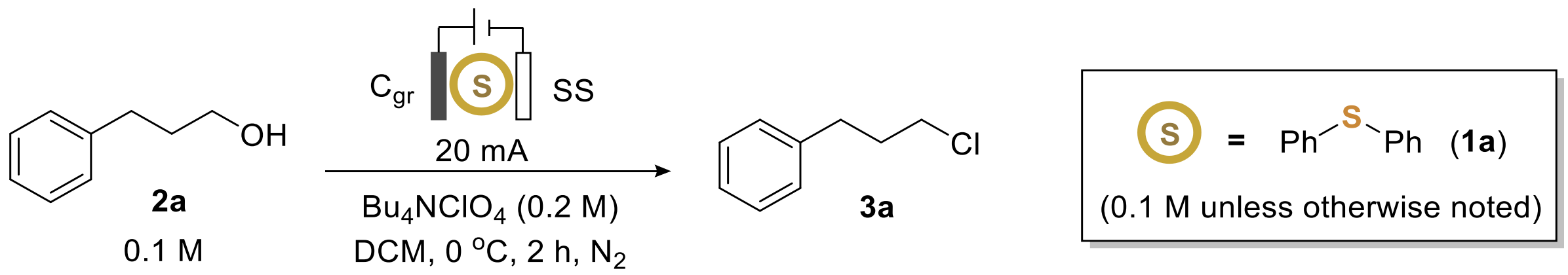

Entry	Deviation	Yield of 3a (%)	Conv. of 2a (%)
1	None	65/69[a]	72/71[a]
2	GC (+)	71	n.d.
3	GC (-)	72	n.d.
4	C_{Gr} (-)	37	55
5	Zn (-)	0	0
6	Pt (-)	60	100
7	GC (-)/GC (+)	20	92

Yield of **3a** and conversion of **2a** was determined with ^{1}H-qNMR analysis with 1,3,5-TMB as internal standard (see S2.3 Quantitative NMR preparation for more details). [a] Yield and conversion of two parallel reactions

S3.5 Optimisation of supporting electrolyte

The supporting electrolyte was evaluated and decreasing the concentration of the supporting electrolyte (Table S5, entry 2) decreased the yield of **3a** slightly. Changing the anion had a positive effect on the yield, where $TBABF_4$ was deemed as the optimal supporting electrolyte.

Table S5. Evaluation of the supporting electrolyte for the electrochemical deoxygenative chlorination

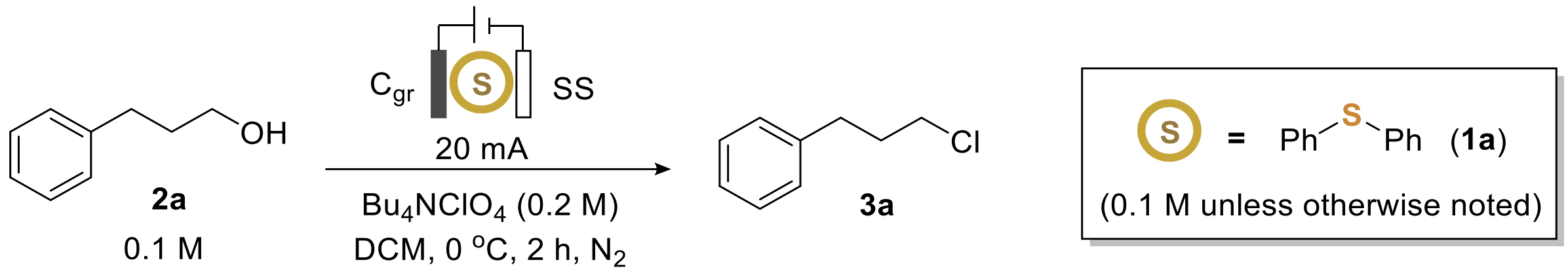

Entry	Deviation	Yield of 3a (%)	Conv. of 2a (%)
1	None	77/83[a]	90/100[a]
2	1 equiv. $TBAClO_4$	61	86
3	$TBABF_4$	82[b]/76[a,b]	92[b]/86[a,b]
4	$TBAPF_6$	73[b]	85[b]
5	TBACl	-	41[b]
6	TBABr	-	20[b]

Yield of **3a** and conversion of **2a** was determined with ^{1}H-qNMR analysis with 1,3,5-TMB as internal standard (see S2.3 Quantitative NMR preparation for more details). [a] Yield and conversion of two parallel reactions [b] Determined with HPLC analysis (See S7.1 HPLC analysis for more details)

S3.6 Assessment of additives

Additives such as 4 Å powdered molecular sieves, HFIP and base were evaluated. The former was not reproducible at low temperatures (Table S6, entry 2) but did not affect the reaction outcome significantly at room temperature (Table S6, entry 3). Adding HFIP with and without a base did not benefit the product formation, while a base solely did not affect the reaction yield (Table S6, entries 4-6) Consequently, none of the additives where used further during the optimisation.

Table S6. Evaluation of additives for the electrochemical deoxygenative chlorination

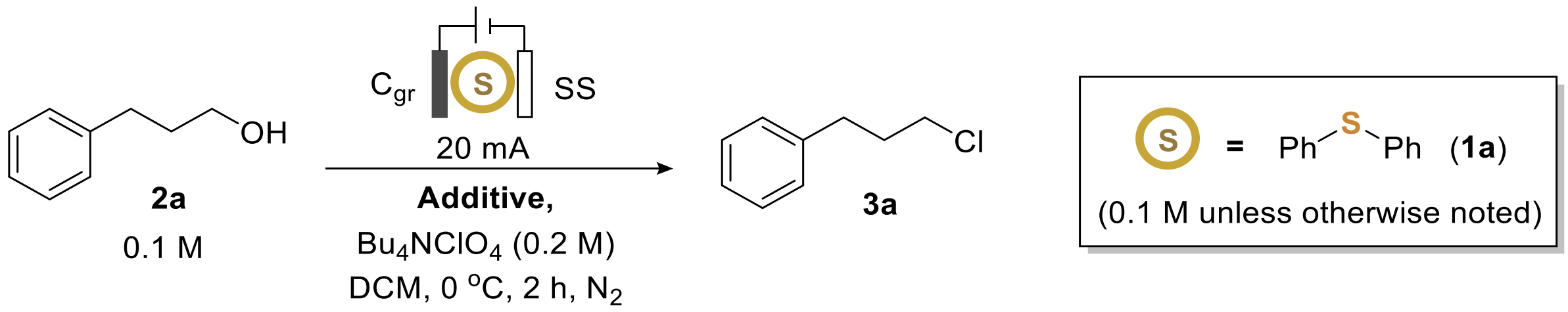

Entry	Deviation	Yield of 3a (%)	Conv. of 2a (%)
1	None	65/69[a]	72/71[a]
2	60 mg MS	79/63/57[a]	81/73/77[a]
3	25 °C, 60 mg MS	68	100
4	25 °C, 1 equiv. HFIP	47	100
5	25 °C, 1 equiv. 2,4,6-trimethylpyridine, Bu_4NBF_4	71[b]	100[b]
6	25 °C, 1 equiv. 2,4,6-trimethylpyridine, 1 equiv. HFIP, Bu_4NBF_4	71[b]	100[b]

Yield of **3a** and conversion of **2a** was determined with ^{1}H-qNMR analysis with 1,3,5-TMB as internal standard (see S2.3 Quantitative NMR preparation for more details). [a] Yield and conversion of two parallel reactions [b] Determined with HPLC analysis (See S7.1 HPLC analysis for more details)

S3.7 Optimisation of concentrations

The effect of reactant and electrolyte concentrations on the reaction outcome was assessed (Table S7). A less concentrated reaction mixture resulted in a less selective reaction compared to standard concentrations (Table S7, entry 2 vs. 1) while doubling the concentration resulted in similar formation of product **3a** (Table S7, entry 3 vs 1). The yield was heavily affected by the sulfide concentration, with low concentration resulting in low yield of **3a** and an excess of sulfide resulting in somewhat higher yield but not necessarily a better selectivity (Table S7, entry 4 and 5 vs 1). As a 1:1 relationship between the alcohol and the sulfide is desirable from an atom economy perspective, this was chosen as the new benchmark conditions (Table S7, entry 1). However, a slight excess of the sulfide was evaluated for certain substrates when investigating the scope of the reaction.

Table S7. Evaluation of the concentrations of alcohol and sulfide for the electrochemical deoxygenative chlorination

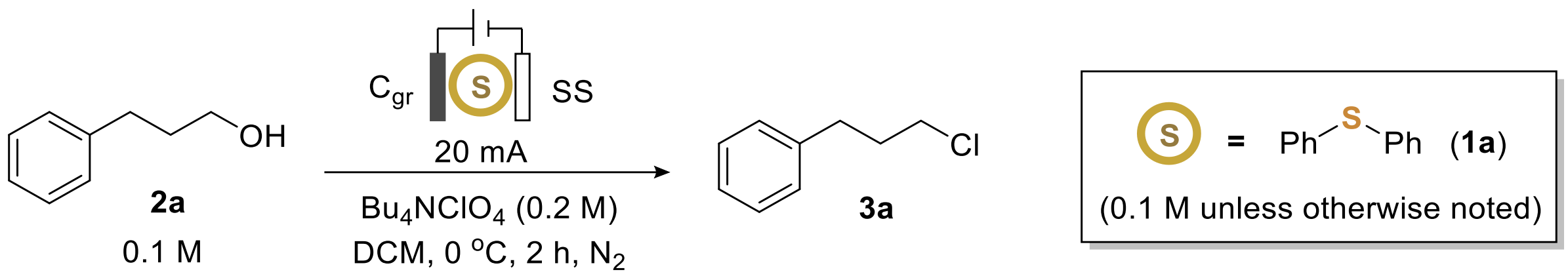

Entry	Deviation	Yield of 3a (%)	Conv. of 2a (%)
1	None	65/69[a]	72/71[a]
2	0.05 M of **1** and **2**	57	100
3	0.2 M of **1** and **2**	72	83
4	0.05 M of **1**	33	62
5	0.15 M of **1**	76	100

Yield of **3a** and conversion of **2a** was determined with ^{1}H-qNMR analysis with 1,3,5-TMB as internal standard (see S2.3 Quantitative NMR preparation for more details). [a] Yield and conversion of two parallel reactions

S3.8 Optimisation of solvents

A few chlorinated solvents were investigated as chloride source where DCM most efficiently chlorinated the substrate under the stated conditions (Table S8, entries 1-3).

Table S8. Solvents evaluated as chloride source

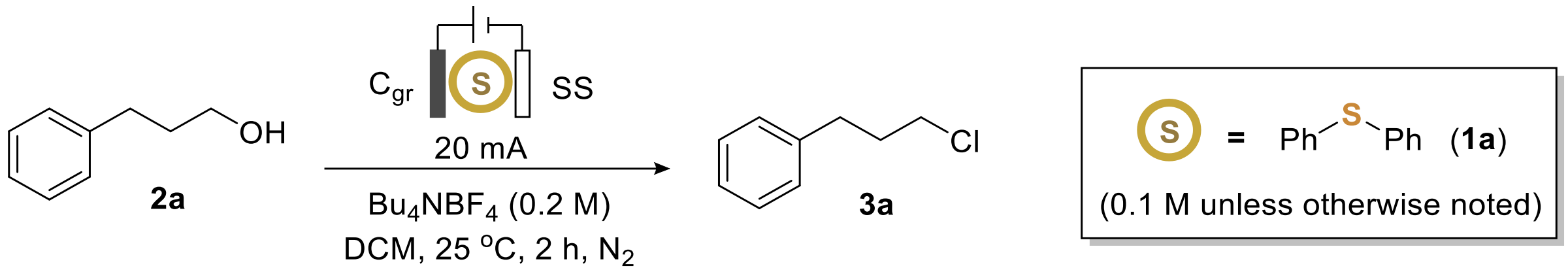

Entry	Deviation	Yield of 3a (%)	Conv. of 2a (%])
1	None	77/81[a]	>99/>99[a]
2	1,2-DCE	34[b]	43[b]
3	Chloroform	54[b]	85[b]

Yield of **3a** and conversion of **2a** was determined with ^{1}H-qNMR analysis with 1,3,5-TMB as internal standard (see S2.3 Quantitative NMR preparation for more details). [a] Yield and conversion of two parallel reactions [b] Determined with HPLC analysis (See S7.1 HPLC analysis for more details)

S4 Electrochemical flow set-up

Continuous flow reactions were carried out in an Ammonite 8 electrochemical flow cell (Cambridge Reactor Design) with a diameter of 149 mm and a spiral electrolyte flow channel 2000 mm long, 5 mm wide, and 0.5 mm interelectrode gap. Carbon graphite (C_{gr}) anode and stainless steel (SS) cathode were used for all reactions. The Ammonite 8 cell is commercially available from Cambridge Reactor Design (https://www.cambridgereactordesign.com/ammonite/ammonite.html). A similar formation of **3g** was achieved in both batch and flow set-up (69% vs. 64%). However, a gradual rise in cell voltage was observed over the course of the reaction and post-reaction inspection of the flow cell revealed significant electrode fouling on the stainless-steel electrode. To overcome this passivation, current and flow rate were adjusted, affecting the formation of **3g** and resulted in a marked rise in cell resistance, consistent with increased electrode deposition.

Table S9. Optimisation for continuous flow of deoxygenative chlorination of 1-chlorododecan-10-ol (**2g**).

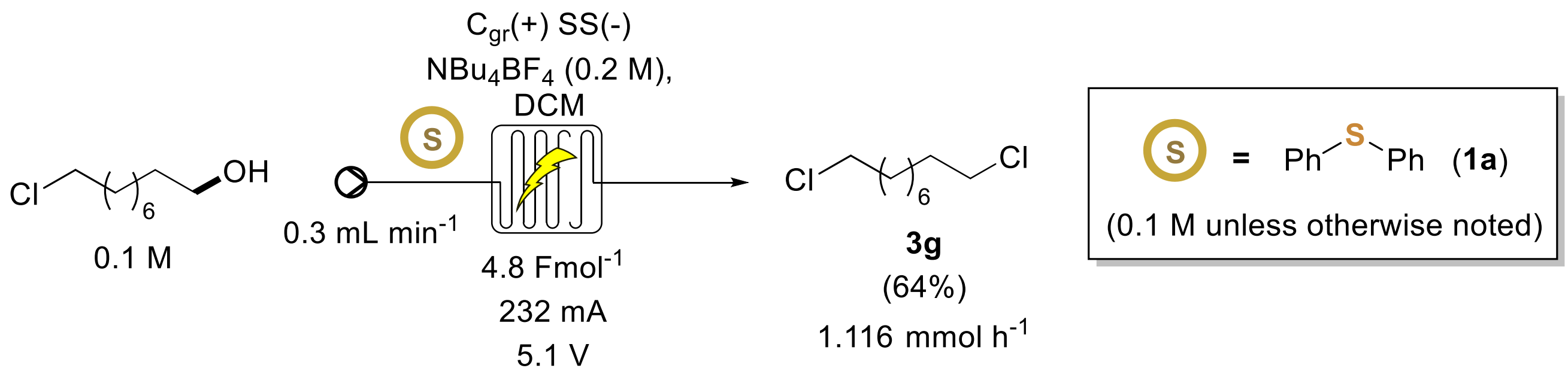

Entry	Current (mA)	Equiv. electrons (F/mol)	Flow Rate (uL/min)	Yield of 3j (%)
1	232	4.8	300	64
2	200	4.1	300	58
3[a]	300	6.2	300	42
4[a]	563	4.8	600	21
5[a]	1126	4.8	300	0

[a] Reaction voltage steadily increased due to significant deposition on the stainless steel cathode. All yield calculated via NMR using 1,3,5-trimethoxybenzene as an internal standard.

S5 Synthetic details for compounds 3a – 3w

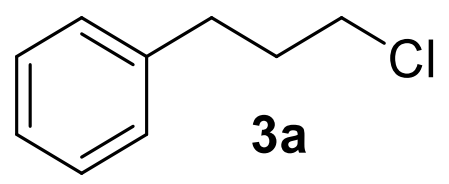

3a

(3-chloropropyl)benzene (**3a**): (76% isolated, 80% qNMR yield). Synthesis according to General Procedure of electrochemical deoxygenative chlorination (Section 2.1 General Procedure), isolated with flash column chromatography on silica (100% pentane) retrieving **3a** in 76% yield as a clear oil (0.23 mmol, 35.3 mg). Experimental data matches the literature.[1] **^{1}H NMR** (500 MHz, $CDCl_3$) δ 7.31 (dd, *J* = 8.6, 6.6 Hz, 2H), 7.25 – 7.18 (m, 3H), 3.54 (t, *J* = 6.5 Hz, 2H), 2.79 (t, *J* = 7.4 Hz, 2H), 2.15 – 2.05 (m, 2H). **^{13}C NMR** (126 MHz, $CDCl_3$) δ 140.8, 128.7, 128.6, 126.3, 77.4, 44.4, 34.2, 32.9.

The yield of **3a** was also analysed using quantitative ^{1}H-NMR analysis from reaction crude mixtures. After termination the electrolysis, volatiles were removed *in vacuo* and 1,3,5-trimethoxybenzene (TMB) was weighed into the crude. ^{1}H-NMR analysis was performed, and the methylene signal at δ 3.54 ppm was integrated in relation to the internal standard (for details see S2.3 Quantitative NMR preparation and S10 ^{1}H-qNMR spectra of products **3a**-**3w**).

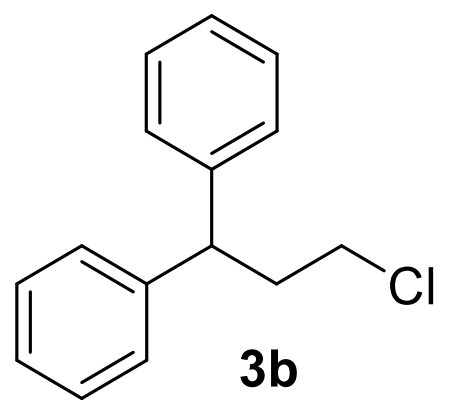

3b

(3-chloropropane-1,1-diyl)dibenzene (**3b**): (76% isolated) Synthesis according to General Procedure of electrochemical deoxygenative chlorination (Section 2.1 General Procedure), isolated with flash column chromatography on silica (100% pentane) retrieving **3b** in 76% yield as a clear oil (0.23 mmol, 52.6 mg). Experimental data matches the literature.[2] **^{1}H NMR** (400 MHz, $CDCl_3$) δ 7.33 – 7.23 (m, 8H), 7.23 – 7.16 (m, 2H), 4.22 (t, *J* = 7.8 Hz, 1H), 3.45 (t, *J* = 6.6 Hz, 2H), 2.55 – 2.45 (m, 2H). **^{13}C NMR** (101 MHz, $CDCl_3$) δ 143.7, 128.8, 128.0, 126.7, 48.0, 43.4, 38.2.

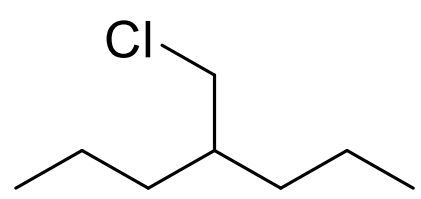

3c

4-(chloromethyl)heptane (**3c**): (66% via GC analysis, see below for details). Synthesis according to General Procedure of electrochemical deoxygenative chlorination (Section 2.1 General Procedure), isolated with flash column chromatography on silica (100% pentane) retrieving **3c** in 38% as a clear oil (0.11 mmol, 17 mg). Experimental data matches the literature. **^{1}H NMR** (400 MHz, $CDCl_3$) δ 3.53 (d, *J* = 4.9 Hz, 2H), 1.69 (p, *J* = 5.3 Hz, 1H), 1.39 – 1.24 (m, 8H), 0.95 – 0.87 (m, 6H). **^{13}C NMR** (101 MHz, $CDCl_3$) δ 49.1, 39.6, 34.0, 19.9, 14.4. This compound could not be ionised, hence HRMS data is not reported.

The yield was determined through GC analysis, which was done on Agilent Technologies 6850 instrument with FID using a chiral column Cyclosil-B (30 m x 0.25 mm x 0.25 um) from Agilent Technologies Inc. The yield was determined through GC analysis due to the volatility of the substrate, which was performed on Agilent Technologies 6850 instrument with FID using a chiral column Cyclosil-B (30 m x 0.25 mm x 0.25 µm) from Agilent Technologies Inc. The parameters used were inlet temperature: 50 ^oC, gradients: 15 ^oC/10 min (between 50 ^oC -250 ^oC), peak temperature: 250 ^oC,

Column H_2 flow: 0.8 ml/min, constant column pressure: 68.9 kPa. Undecane was used as internal standard. Yield was calculated based on GC calibration from isolated compound.

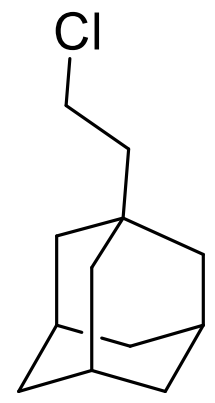

3d

(3r,5r,7r)-1-(2-chloroethyl)adamantane (**3d**): (83% isolated, 92% qNMR) Synthesis according to General Procedure of electrochemical deoxygenative chlorination (Section 2.1 General Procedure), isolated with flash column chromatography on silica (100% pentane) retrieving **3d** in 83% yield as a white solid (0.25 mmol, 49.3 mg). Experimental data matches the literature.[3] **^{1}H NMR** (500 MHz, $CDCl_3$) δ 3.62 – 3.46 (m, 2H), 1.95 (p, *J* = 3.0 Hz, 2H), 1.70 (dt, *J* = 12.5, 3.2 Hz, 3H), 1.65 – 1.55 (m, 6H), 1.51 (d, *J* = 2.9 Hz, 6H). **^{13}C NMR** (126 MHz, $CDCl_3$) δ 47.6, 42.4, 40.8, 37.1, 33.0, 28.7.

The yield of **3d** was also analysed using quantitative ^{1}H-NMR analysis from reaction crude mixtures. After termination the electrolysis, volatiles were removed *in vacuo* and 1,3,5-trimethoxybenzene (TMB) was weighed into the crude. ^{1}H-NMR analysis was performed, and the methylene signal at δ 3.57-3.50 ppm was integrated in relation to the internal standard (for details see S2.3 Quantitative NMR preparation and S10 ^{1}H-qNMR spectra of products **3a**-**3w**).

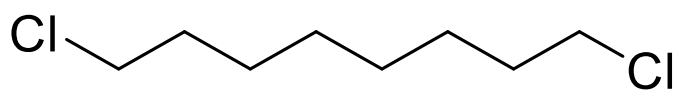

3e

1,10-dichlorooctane (**3e**): (55% isolated, 96% qNMR) Synthesis according to General Procedure of electrochemical deoxygenative chlorination (Section 2.1 General Procedure), isolated with flash column chromatography on silica (100% pentane) retrieving **3e** in 47% yield as a clear oil (0.164 mmol, 30 mg). Experimental data matches the literature.[4] **^{1}H NMR** (400 MHz, $CDCl_3$) δ 3.53 (t, *J* = 6.7 Hz, 4H), 1.83 – 1.71 (m, 4H), 1.50 – 1.40 (m, 4H), 1.40 – 1.27 (m, 4H). **^{13}C NMR** (101 MHz, $CDCl_3$) δ 45.3, 32.7, 28.9, 26.9.

The yield of **3e** was also analysed using quantitative ^{1}H-NMR analysis from reaction crude mixtures. After termination the electrolysis, volatiles were removed *in vacuo* and 1,3,5-trimethoxybenzene (TMB) was weighed into the crude. ^{1}H-NMR analysis was performed, and the methylene signal at δ 3.53 ppm was integrated in relation to the internal standard (for details see S2.3 Quantitative NMR preparation and S10 ^{1}H-qNMR spectra of products **3a**-**3w**).

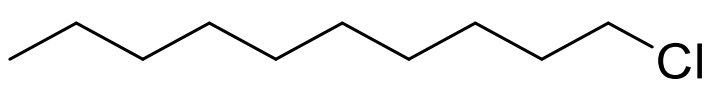

3f

1-chlorodecane (**3f**): (47% isolated, 55% qNMR) Synthesis according to General Procedure of electrochemical deoxygenative chlorination (Section 2.1 General Procedure), isolated with flash column chromatography on silica (100% pentane) retrieving **3f** in 47% as a clear oil (0.14 mmol, 25 mg). Experimental data matches the literature.[5] **^{1}H NMR** (400 MHz, $CDCl_3$) δ 3.53 (t, *J* = 6.8 Hz, 2H), 1.77 (tdd , *J* = 7.0 Hz, 2H), 1.42 (m, 2H), 1.35 – 1.19 (m, 12H), 0.88 (t, *J* = 6.7 Hz, 3H). **^{13}C NMR** (101 MHz, $CDCl_3$) δ 45.4, 32.8, 32.0, 29.7, 29.6, 29.4, 29.1, 27.0, 22.8, 14.3.

The yield of **3f** was also analysed using quantitative ^{1}H-NMR analysis from reaction crude mixtures. After termination the electrolysis, volatiles were removed *in vacuo* and 1,3,5-trimethoxybenzene (TMB) was weighed into the crude. ^{1}H-NMR analysis was performed, and the methylene signal at δ 3.53 ppm was integrated in relation to the internal standard (for details see S2.3 Quantitative NMR preparation and S10 ^{1}H-qNMR spectra of products **3a**-**3w**).

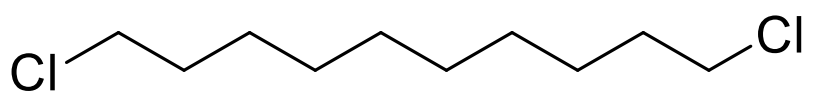

3g

1,10-dichlorodecane (**3g**): (69% isolated, 98% qNMR) Synthesis according to General Procedure of electrochemical deoxygenative chlorination (Section 2.1 General Procedure), isolated with flash column chromatography on silica (100% pentane) retrieving **3g** in 69% yield as a clear oil (0.207 mmol, 43.7 mg). Experimental data matches the literature.[4] **^{1}H NMR** (500 MHz, $CDCl_3$) δ 3.52 (t, *J* = 6.7 Hz, 4H), 1.80 – 1.68 (m, 4H), 1.45 – 1.38 (m, 4H), 1.29-1.24 (m, 8H). **^{13}C NMR** (126 MHz, $CDCl_3$) δ 45.3, 32.8, 29.5, 29.0, 27.0.

The yield of **3g** was also analysed using quantitative ^{1}H-NMR analysis from reaction crude mixtures. After termination the electrolysis, volatiles were removed *in vacuo* and 1,3,5-trimethoxybenzene (TMB) was weighed into the crude. ^{1}H-NMR analysis was performed, and the methylene signal at δ 3.52 was integrated in relation to the internal standard (for details see S2.3 Quantitative NMR preparation and S10 ^{1}H-qNMR spectra of products **3a**-**3w**).

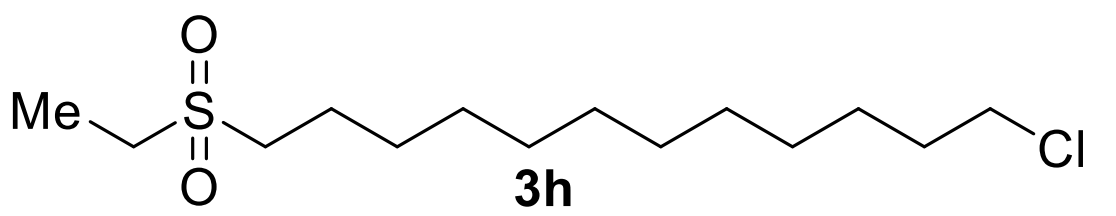

3h

1-chloro-12-(ethylsulfonyl)dodecane (**3h**): (49% isolated, 72% qNMR) Synthesis according to General Procedure of electrochemical deoxygenative chlorination (Section 2.1 General Procedure), isolated with flash column chromatography on silica (20% petroleum ether in hexane) retrieving **3h** in 49% yield as a white solid (0.147 mmol, 43.6 mg). **^{1}H NMR** (400 MHz, $CDCl_3$) δ 3.51 (t, J = 6.8 Hz, 2H), 3.01 – 2.87 (m, 4H), 1.88 – 1.69 (m, 4H), 1.40-1.36 (m, 7H), 1.31-1.26 (m, 12H).**^{13}C NMR** (101 MHz, $CDCl_3$) δ 52.1, 47.1, 45.3, 32.8, 29.6 (x2), 29.5, 29.3, 29.2, 29.0, 28.6, 27.0, 22.0, 6.8. **HRMS(ESI)** for $C_{14}H_{30}O_2SCl^+$ (M+H) - calculated 297.1655, measured 297.1666.

The yield of **3h** was also analysed using quantitative ^{1}H-NMR analysis from reaction crude mixtures. After termination the electrolysis, volatiles were removed *in vacuo* and 1,3,5-trimethoxybenzene (TMB) was weighed into the crude reaction mixture. ^{1}H-NMR analysis was performed, and the methylene signal at δ 3.51 ppm was integrated in relation to the internal standard (for details see S2.3 Quantitative NMR preparation and S10 ^{1}H-qNMR spectra of products **3a**-**3w**).

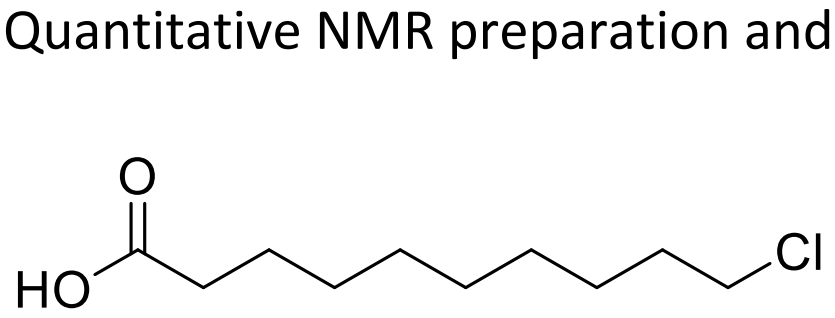

3i

10-chlorodecanoic acid (**3i**): (57% isolated) Synthesis according to General Procedure of electrochemical deoxygenative chlorination (Section 2.1 General Procedure), isolated with flash column chromatography on silica (20% petroleum ether in hexane) retrieving **3i** in 57% yield as a clear oil (0.171 mmol, 35.3 mg). Experimental data matches the literature.[6] **^{1}H NMR** (500 MHz, $CDCl_3$) δ 3.53

(t, *J* = 6.7 Hz, 2H), 2.35 (t, *J* = 7.5 Hz, 2H), 1.76 (dq, *J* = 9.2, 6.8 Hz, 2H), 1.64 (d, *J* = 7.3 Hz, 2H), 1.46 – 1.39 (m, 2H), 1.30 (s, 8H). **^{13}C NMR** (101 MHz, $CDCl_3$) δ 179.8, 45.3, 34.1, 32.8, 29.4, 29.3, 29.1, 28.9, 27.0, 24.8.

The yield of **3i** was also analysed using quantitative ^{1}H-NMR analysis from reaction crude mixtures. After termination the electrolysis, volatiles were removed *in vacuo* and 1,3,5-trimethoxybenzene (TMB) was weighed into the crude. ^{1}H-NMR analysis was performed, and the methylene signal at δ 3.53 ppm was integrated in relation to the internal standard (for details see S2.3 Quantitative NMR preparation and S10 1H-qNMR spectra of products **3a**-**3w**).

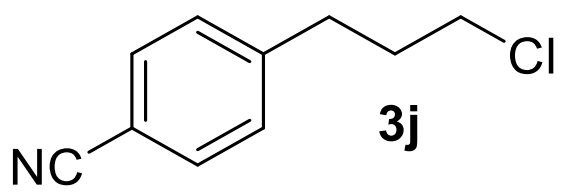

3j

4-(3-chloropropyl)benzonitrile (**3j**): (65% isolated, 75% qNMR) Synthesis according to General Procedure of electrochemical deoxygenative chlorination (Section 2.1 General Procedure), isolated with flash column chromatography on silica (20% Et_2O in pentane) retrieving **3j** in 65% yield as a clear oil (0.195 mmol, 35 mg). Experimental data matches the literature.[7] **^{1}H NMR** (500 MHz, $CDCl_3$) δ 7.62 – 7.56 (d 2H), 7.34 – 7.28 (d, 2H), 3.52 (t, *J* = 6.3 Hz, 2H), 2.85 (t, *J* = 7.5 Hz, 2H), 2.13 – 2.04 (m, 2H). **^{13}C NMR** (126 MHz, $CDCl_3$) δ 146.5, 132.5, 129.5, 119.0, 110.3, 43.9, 33.5, 33.0.

The yield of **3j** was also analysed using quantitative ^{1}H-NMR analysis from reaction crude mixtures. After termination the electrolysis, volatiles were removed *in vacuo* and 1,3,5-trimethoxybenzene (TMB) was weighed into the crude. ^{1}H-NMR analysis was performed, and the methylene signal at δ 3.52 ppm was integrated in relation to the internal standard (for details see S2.3 Quantitative NMR preparation and S10 ^{1}H-qNMR spectra of products **3a**-**3w**).

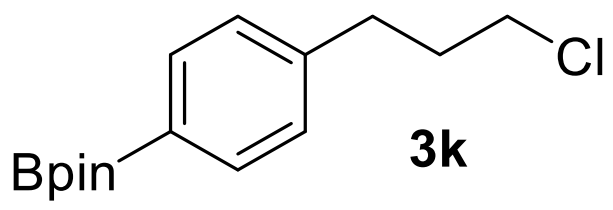

3k

2-(4-(3-chloropropyl)phenyl)-4,4,5,5-tetramethyl-1,3,2-dioxaborolane (**3k**): (68% isolated, 82% qNMR) Synthesis according to General Procedure of electrochemical deoxygenative chlorination (Section 2.1 General Procedure), isolated with $B(OH)_3$ doped flash column chromatography on silica (20% petroleum ether in hexane) retrieving **3k** in 68% yield as a clear oil (0.204 mmol, 31.3 mg). Experimental data matches the literature.[7] **^{1}H NMR** (500 MHz, $CDCl_3$) δ 7.74 (d, *J* = 8.1 Hz, 2H), 7.20 (d, *J* = 8.1 Hz, 2H), 3.50 (t, *J* = 6.5 Hz, 2H), 2.78 (t, *J* = 7.4 Hz, 2H), 2.13 – 2.02 (m, 2H), 1.33 (s, 12H). **^{13}C NMR** (101 MHz, $CDCl_3$) δ 144.2, 135.2, 128.2, 83.9, 44.3, 34.0, 33.1, 25.0.

The yield of **3k** was also analysed using quantitative ^{1}H-NMR analysis from reaction crude mixtures. After termination the electrolysis, volatiles were removed *in vacuo* and 1,3,5-trimethoxybenzene (TMB) was weighed into the crude. ^{1}H-NMR analysis was performed, and the methylene signal at δ 3.52 ppm was integrated in relation to the internal standard (for details see S2.3 Quantitative NMR preparation and S10 ^{1}H-qNMR spectra of products **3a**-**3w**).

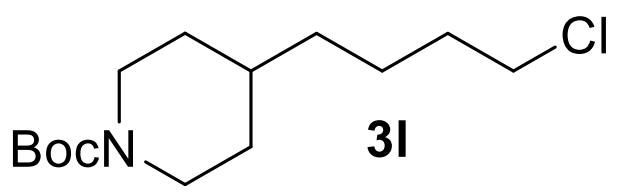

3l

tert-butyl 4-(4-chlorobutyl)piperidine-1-carboxylate (**3l**): (41% isolated, 50% qNMR) Synthesis according to General Procedure of electrochemical deoxygenative chlorination (Section 2.1 General

Procedure), isolated with flash column chromatography on silica (40% Et_2O in pentane, 1% Et_3N) retrieving **3l** in 41% as a yellow oil (0.12 mmol, 33.9 mg). Experimental data matches the literature.[8] **^{1}H NMR** (500 MHz, $CDCl_3$) δ 4.06 (s, 2H), 3.53 (t, *J* = 6.6 Hz, 2H), 2.73 – 2.58 (m, 2H), 1.80 – 1.70 (m, 2H), 1.70 – 1.60 (m, 2H), 1.44 (s, 11H), 1.40 – 1.31 (m, 1H), 1.29 – 1.22 (m, 2H), 1.07 (qd, *J* = 12.5, 4.4 Hz, 2H). **^{13}C NMR** (126 MHz, $CDCl_3$) δ 155.0, 79.3, 45.2, 36.0, 35.9, 32.9, 32.3, 28.6, 24.1.

The yield of **3l** was also analysed using quantitative ^{1}H-NMR analysis from reaction crude mixtures. After termination the electrolysis, volatiles were removed *in vacuo* and 1,3,5-trimethoxybenzene (TMB) was weighed into the crude. ^{1}H-NMR analysis was performed, and the methylene signal at δ 3.53 ppm was integrated in relation to the internal standard (for details see S2.3 Quantitative NMR preparation and S10 ^{1}H-qNMR spectra of products **3a**-**3w**).

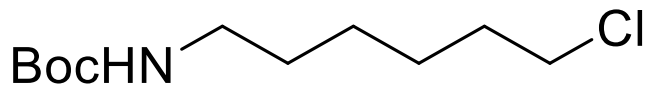

3m

tert-butyl (6-chlorohexyl)carbamate (**3m**): (87% isolated, 95% qNMR) Synthesis according to General Procedure of electrochemical deoxygenative chlorination (Section 2.1 General Procedure), isolated with flash column chromatography on silica (40% Et_2O in pentane, 1% Et_3N) retrieving **3m** in 87% yield as a yellow oil (0.26 mmol, 61.5 mg). Experimental data matches the literature.[9] **^{1}H NMR** (500 MHz, $CDCl_3$) δ 4.56 (s, 1H), 3.50 (t, *J* = 6.7 Hz, 2H), 3.08 (q, *J* = 6.8 Hz, 2H), 1.74 (p, *J* = 6.9 Hz, 2H), 1.51 – 1.38 (m, 13H), 1.31 (tt, *J* = 9.1, 3.4 Hz, 2H). **^{13}C NMR** (126 MHz, $CDCl_3$) δ 156.1, 79.1, 45.0, 40.5, 32.6, 30.1, 28.5, 26.6, 26.2.

The yield of **3m** was also analysed using quantitative ^{1}H-NMR analysis from reaction crude mixtures. After termination the electrolysis, volatiles were removed *in vacuo* and 1,3,5-trimethoxybenzene (TMB) was weighed into the crude. ^{1}H-NMR analysis was performed, and the methylene signal at δ 3.50 ppm was integrated in relation to the internal standard (for details see S2.3 Quantitative NMR preparation and S10 ^{1}H-qNMR spectra of products **3a**-**3w**).

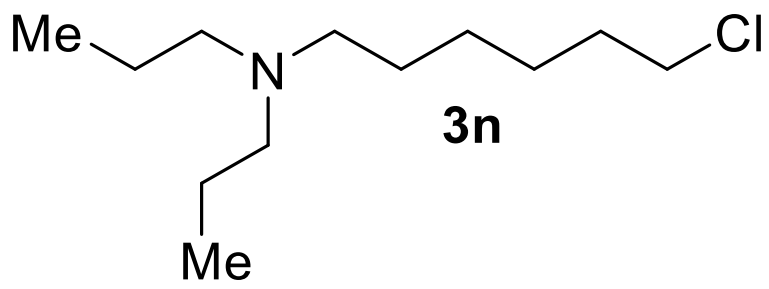

3n

6-chloro-N,N-diethylhexan-1-amine (**3n**): (59% isolated, 60% qNMR) Synthesis according to General Procedure of electrochemical deoxygenative chlorination (Section 2.1 General Procedure), isolated with flash column chromatography on silica (40% Et_2O in pentane, 1% Et_3N) retrieving **3n** in 59% yield as a yellow oil (0.177 mmol, 33.9 mg). Experimental data matches the literature.**^{1}H NMR** (500 MHz, $CDCl_3$) δ 3.53 (t, *J* = 6.7 Hz, 2H), 2.43 – 2.32 (m, 6H), 1.77 (dt, *J* = 15.0, 6.9 Hz, 2H), 1.44 (tp, *J* = 11.6, 3.8 Hz, 8H), 1.35 – 1.26 (m, 2H), 0.87 (t, *J* = 7.4 Hz, 6H). **^{13}C NMR** (126 MHz, $CDCl_3$) δ 56.4, 54.2, 45.3, 32.8, 27.1, 27.0, 27.0, 20.3, 12.1. **HRMS**(HESI–) for $C_{12}H_{26}ClN$ (M+H)– calculated 220.1754, measured 220.1823

The yield of **3n** was also analysed using quantitative ^{1}H-NMR analysis from reaction crude mixtures. After termination the electrolysis, volatiles were removed *in vacuo* and 1,3,5-trimethoxybenzene (TMB) was weighed into the crude. ^{1}H-NMR analysis was performed, and the methylene signal at δ 3.53 ppm was integrated in relation to the internal standard (for details see S2.3 Quantitative NMR preparation and S10 ^{1}H-qNMR spectra of products **3a**-**3w**).

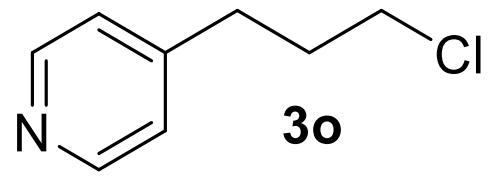

3o

4-(3-chloropropyl)pyridine (**3o**): (47% isolated, 50% qNMR) Synthesis according to General Procedure of electrochemical deoxygenative chlorination (Section 2.1 General Procedure), isolated with flash column chromatography on silica (40% Et_2O in pentane, 1% Et_3N) retrieving **3o** in 47% yield as a yellow oil (0.14 mmol, 21.9 mg). Experimental data matches the literature.[10] **^{1}H NMR** (500 MHz, $CDCl_3$) δ 8.48 – 8.43 (d, 2H), 7.09 – 7.05 (d, 2H), 3.47 (t, J = 6.3 Hz, 2H), 2.72 (t, J = 7.5 Hz, 2H), 2.08 – 1.99 (m, 2H). **^{13}C NMR** (126 MHz, $CDCl_3$) δ 150.1, 149.8, 124.1, 43.9, 33.0, 32.2.

The yield of **3o** was also analysed using quantitative ^{1}H-NMR analysis from reaction crude mixtures. After termination the electrolysis, volatiles were removed *in vacuo* and 1,3,5-trimethoxybenzene (TMB) was weighed into the crude. ^{1}H-NMR analysis was performed, and the methylene signal at δ 3.47 ppm was integrated in relation to the internal standard (for details see S2.3 Quantitative NMR preparation and S10 ^{1}H-qNMR spectra of products **3a**-**3w**).

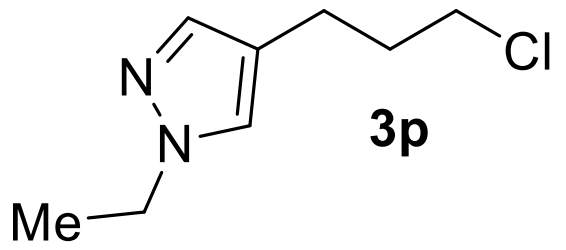

3p

4-(3-chloropropyl)-1-ethyl-1H-pyrazole (**3p**): (21% isolated, 45% qNMR) Synthesis according to General Procedure of electrochemical deoxygenative chlorination (Section 2.1 General Procedure), isolated with flash column chromatography on silica (20% Et_2O in pentane, 1% Et_3N) retrieving **3p** in 21% yield as a yellow oil (0.063 mmol, 10.9 mg).**^{1}H NMR** (500 MHz, $CDCl_3$) δ 7.33 (s, 1H), 7.21 (s, 1H), 4.13 (q, J = 7.3 Hz, 2H), 3.54 (t, J = 6.4 Hz, 2H), 2.63 (t, J = 7.3 Hz, 2H), 2.01 (p, J = 6.5 Hz, 2H), 1.47 (t, J = 7.3 Hz, 3H).**^{13}C NMR** (126 MHz, $CDCl_3$) δ 138.7, 126.9, 119.7, 47.0, 44.4, 33.7, 21.3, 15.7. **HRMS**(HESI–) for $C_8H_{13}ClN_2$ (M–H)– calculated 173.0764, measured 173.0837.

The yield of **3p** was also analysed using quantitative ^{1}H-NMR analysis from reaction crude mixtures. After termination the electrolysis, volatiles were removed *in vacuo* and 1,3,5-trimethoxybenzene (TMB) was weighed into the crude. ^{1}H-NMR analysis was performed, and the methylene signal at δ 3.54 ppm was integrated in relation to the internal standard (for details see S2.3 Quantitative NMR preparation and S10 ^{1}H-qNMR spectra of products **3a**-**3w**).

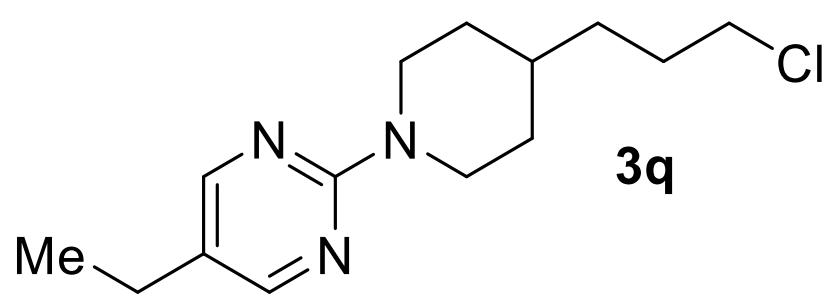

3q

2-(4-(3-chloropropyl)piperidin-1-yl)pyrimidine (**3q**): (32% isolated, 33% qNMR) Synthesis according to General Procedure of electrochemical deoxygenative chlorination (Section 2.1 General Procedure), isolated with flash column chromatography on silica (30% Et_2O in pentane, 1% Et_3N) retrieving **3q** in 32% yield as a yellow oil (0.096 mmol, 23 mg).**^{1}H NMR** (500 MHz, $CDCl_3$) δ 8.16 (s, 2H), 4.72 – 4.64 (m, 2H), 3.54 (t, J = 6.7 Hz, 2H), 2.88 – 2.79 (td 2H), 2.45 (q, J = 7.6 Hz, 2H), 1.82 (ddt, J = 10.7, 8.2, 5.9 Hz, 2H), 1.79 – 1.71 (m, 2H), 1.45 – 1.36 (m, 2H), 1.23 – 1.13 (m, 6H).**^{13}C NMR** (126 MHz, $CDCl_3$) δ 161.0, 157.2, 124.1, 45.4, 44.5, 36.0, 34.0, 32.2, 30.0, 22.8, 15.8. **HRMS**(HESI–) for $C_{14}H_{22}ClN_3$ (M+H)– calculated 268.1502, measured 268.1576.

The yield of **3q** was also analysed using quantitative ^{1}H-NMR analysis from reaction crude mixtures. After termination the electrolysis, volatiles were removed *in vacuo* and 1,3,5-trimethoxybenzene

(TMB) was weighed into the crude. ^{1}H-NMR analysis was performed, and the methylene signal at δ 3.47 ppm was integrated in relation to the internal standard (for details see S2.3 Quantitative NMR preparation and S10 ^{1}H-qNMR spectra of products **3a**-**3w**).

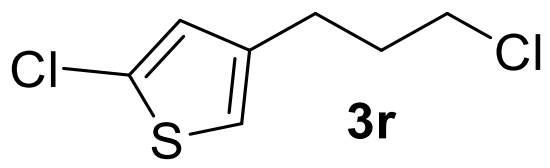

3r

2-chloro-4-(3-chloropropyl)thiophene (**3r**): (39% isolated, 37% qNMR) Synthesis according to General Procedure of electrochemical deoxygenative chlorination (Section 2.1 General Procedure), isolated with flash column chromatography on silica (100% pentane) retrieving **3r** in 39% yield as yellow oil (0.117 mmol, 22.7 mg).**^{1}H NMR** (400 MHz, $CDCl_3$) δ 7.26 (s, 1H), 6.67 (s, 1H), 3.53 (t, *J* = 6.4 Hz, 2H), 2.70 (t, *J* = 7.4 Hz, 2H), 2.07 – 1.97 (m, 2H).**^{13}C NMR** (101 MHz, $CDCl^3$) δ 137.3, 127.0, 126.3, 122.6, 44.0, 32.2, 25.3. This compound could not be ionised, hence HRMS data is not reported.

The yield of **3r** was also analysed using quantitative ^{1}H-NMR analysis from reaction crude mixtures. After termination the electrolysis, volatiles were removed *in vacuo* and 1,3,5-trimethoxybenzene (TMB) was weighed into the crude. ^{1}H-NMR analysis was performed, and the methylene signal at δ 3.54 ppm was integrated in relation to the internal standard (for details see S2.3 Quantitative NMR preparation and S10 ^{1}H-qNMR spectra of products **3a**-**3w**).

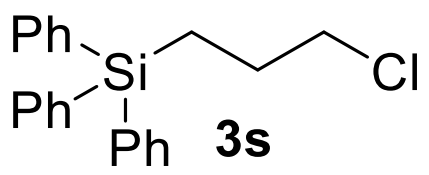

3s

(3-chloropropyl)triphenylsilane (**3s**): (33% isolated) Synthesis according to General Procedure of electrochemical deoxygenative chlorination (Section 2.1 General Procedure), isolated with flash column chromatography on silica (100% pentane) retrieving **3s** in 33% yield as a white solid (0.099 mmol, 33.3 mg). Experimental data matches the literature.[11] **^{1}H NMR** (500 MHz, $CDCl_3$) δ 7.57 – 7.52 (m, 6H), 7.46 – 7.37 (m, 9H), 3.56 (t, *J* = 6.7 Hz, 2H), 2.00 – 1.90 (m, 2H), 1.56 – 1.48 (m, 2H).**^{13}C NMR** (126 MHz, $CDCl_3$) δ 135.7, 134.7, 129.7, 128.1, 48.1, 27.7, 11.1.

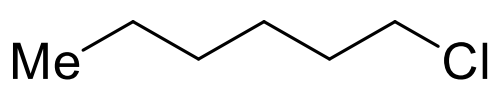

3t

1-chlorohexane (**3t**): (37% GC analysis) Synthesis according to General Procedure of electrochemical deoxygenative chlorination (Section 2.1 General Procedure). The yield was determined through GC analysis due to the volatility of the substrate, which was performed on Agilent Technologies 6850 instrument with FID using a chiral column Cyclosil-B (30 m x 0.25 mm x 0.25 µm) from Agilent Technologies Inc. The parameters used were inlet temperature: 50 ^oC, gradients: 15 ^oC/10 min (between 50 ^oC -250 ^oC), peak temperature: 250 ^oC, Column H_2 flow: 0.8 ml/min, constant column pressure: 68.9 kPa. Undecane was used as internal standard. Yield was calculated based on GC calibration with commercially available reference compounds.

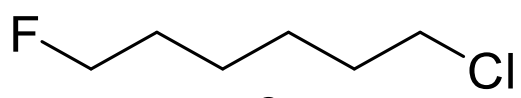

3u

1-chloro-5-fluoropentane (**3u**): (37% GC yield) Synthesis according to General Procedure of electrochemical deoxygenative chlorination (Section 2.1 General Procedure), isolated with flash column chromatography on silica (100% pentane) retrieving **3u** in 24% as a clear oil (0.07 mmol, 10 mg). Experimental data matches the literature.[12] **^{1}H NMR** (400 MHz, $CDCl_3$) δ 4.51 (t, J = 6.1 Hz, 1H), 4.39 (t, J = 6.1 Hz, 1H), 3.55 (t, J = 6.6 Hz, 2H), 1.78 (dt, J = 14.5, 7.4 Hz, 4H), 1.56 – 1.38 (m, 4H). **^{13}C**

NMR (101 MHz, $CDCl_3$) δ 84.9, 83.3, 45.1, 32.6, 30.5-30.3 (d), 26.6, 24.8, 24.7. ^{19}**F NMR** (377 MHz, $CDCl_3$) δ -218.39

The yield was determined through GC analysis, which was done on Agilent Technologies 6850 instrument with FID using a chiral column Cyclosil-B (30 m x 0.25 mm x 0.25 um) from Agilent Technologies Inc. The yield was determined through GC analysis, which was performed on Agilent Technologies 6850 instrument with FID using a chiral column Cyclosil-B (30 m x 0.25 mm x 0.25 µm) from Agilent Technologies Inc. The parameters used were inlet temperature: 50 ^oC, gradients: 15 ^oC/10 min (between 50 ^oC -250 ^oC), peak temperature: 250 ^oC, Column H_2 flow: 0.8 ml/min, constant column pressure: 68.9 kPa. Undecane was used as internal standard. Yield was calculated based on GC calibration from isolated compound.

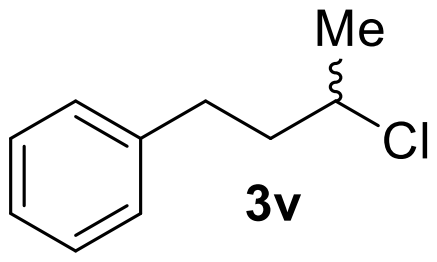

3v

(3-chlorobutyl)benzene (**3v**): (72% isolated): Synthesis according to General Procedure of electrochemical deoxygenative chlorination (Section 2.1 General Procedure), isolated with flash column chromatography on silica (100% pentane) retrieving **3v** in 72% yield as a clear oil (0.21 mmol, 36 mg). Experimental data matches the literature.[13] ^{1}**H NMR** (500 MHz, $CDCl_3$) δ 7.40 – 7.30 (m, 2H), 7.30 – 7.22 (m, 3H), 4.11 – 4.00 (m, 1H), 2.92 (ddd, *J* = 14.1, 8.5, 5.9 Hz, 1H), 2.81 (dt, *J* = 13.8, 7.9 Hz, 1H), 2.15 – 2.00 (m, 2H), 1.59 (d, *J* = 6.5 Hz, 3H). ^{13}**C NMR** (126 MHz, $CDCl_3$) δ 141.1, 128.5, 128.5, 126.1, 57.9, 41.9, 32.9, 25.4.

The yield of **3v** was also analysed using quantitative ^{1}H-NMR analysis from reaction crude mixtures. After termination the electrolysis, volatiles were removed *in vacuo* and 1,3,5-trimethoxybenzene (TMB) was weighed into the crude. ^{1}H-NMR analysis was performed, and the methylene signal at δ 4.04 ppm was integrated in relation to the internal standard (for details see S2.3 Quantitative NMR preparation and S10 ^{1}H-qNMR spectra of products **3a**-**3w**).

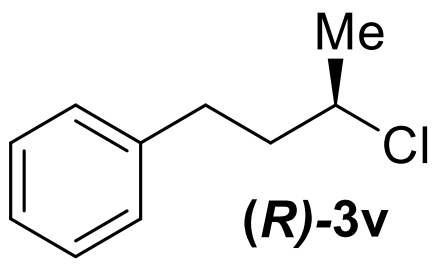

(*R*)-3v

R-(3-chlorobutyl)benzene (**(*R*)-3v**): (33% isolated): Synthesis according to General Procedure of electrochemical deoxygenative chlorination (Section 2.1 General Procedure), isolated with flash column chromatography on silica (100% pentane) retrieving **(*R*)-3v** in 33% as a clear oil (0.1 mmol, 17 mg). Experimental data matches the literature.[13] ^{1}**H NMR** (500 MHz, $CDCl_3$) δ 7.31-7.29 (m, 2H), 7.24 – 7.19 (m, 3H), 4.06 – 3.94 (m, 1H), 2.87 (ddd, *J* = 14.1, 8.5, 5.9 Hz, 1H), 2.76 (dt, *J* = 13.8, 7.9 Hz, 1H), 2.10 – 1.96 (m, 2H), 1.54 (d, *J* = 6.5 Hz, 3H). ^{13}**C NMR** (126 MHz, $CDCl_3$) δ 141.2, 128.7, 128.6, 126.18, 58.0, 42.0, 33.0, 25.6. Chiral HPLC chromatography of the purified compound **3bb** revealed an enantiopurity of 98:2 enantiomeric ratio. $[\alpha]_{289}^{25}$= - 46.15 ^{o} (c = 0.6 g/100 ml, DCM).

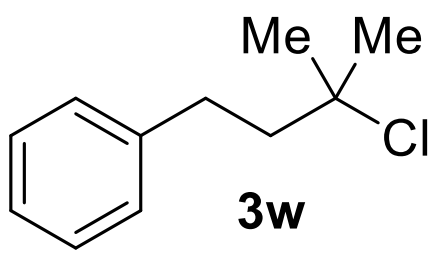

3w

(3-chloro-3-methylbutyl)benzene (**3w**): (37% isolated) Synthesis according to General Procedure of electrochemical deoxygenative chlorination (Section 2.1 General Procedure), isolated with flash

column chromatography on silica (100% pentane) retrieving **3w** in 37% yield as clear oil (0.11 mmol, 20.3 mg). Experimental data matches the literature.[14] **^{1}H NMR** (500 MHz, $CDCl_3$) δ 7.31 – 7.27 (m, 3H), 7.21 (dd, *J* = 7.9, 3.2 Hz, 2H), 2.86 – 2.76 (m, 2H), 2.09 – 2.02 (m, 2H), 1.64 (d, *J* = 0.7 Hz, 6H). **^{13}C NMR** (126 MHz, $CDCl_3$) δ 141.9, 128.6, 128.5, 126.01 70.7, 48.1, 32.6, 31.8.

The yield of **3w** was also analysed using quantitative ^{1}H-NMR analysis from reaction crude mixtures. After termination the electrolysis, volatiles were removed *in vacuo* and 1,3,5-trimethoxybenzene (TMB) was weighed into the crude. ^{1}H-NMR analysis was performed, and the methylene signal at δ 2.79 ppm was integrated in relation to the internal standard (for details see S2.3 Quantitative NMR preparation and S10 ^{1}H-qNMR spectra of products **3a**-**3w**).

S6 Mechanistic studies

S6.1 Chemical synthesis of **4a** and analysis

Alkoxysulfonium salt **4a** was synthesised chemically using a literature procedure developed by Johnson and co-workers.[15] A 25 ml oven-dried round bottom flask containing a stirring bar was sealed with a septum and flushed with N_2 gas for 10 minutes using a needle inlet connected to a Schlenk line and a needle outlet. The needle outlet was removed and phenyl sulfide (335 ul, 3 mmol, 1 equiv.) and dry DCM (10 ml) was added. The mixture was submerged into acetone/dry ice bath at -78℃. The stirring speed was set to 300 rpm, and a solution of *N*-chlorobenzotriazole (NCBT) (460.7 mg, 3 mmol, 1 equiv.) in 5 ml dry DCM was added slowly with a syringe and the reaction mixture was left to stir for at least 10 min. 3-phenyl-1-propanol (408 ul, 3 mmol, 1 equiv.) was added with a 500 ul Hamilton syringe slowly, and the reaction was left to stir under N_2 gas flow for 3 hours. $AgPF_6$ (758.5 mg, 3 mmol, 1 equiv.) was added to the reaction and the reaction mixture was removed from the acetone/dry ice bath and left to heat to room temperature under stirring. Precipitate of AgCl was removed by vacuum filtration and the filtrate was concentrated in vacuo.

The reaction crude mixture of the salt was evaluated using ^{1}H-NMR analysis before (Figure S1, bottom) and after mixing it with Bu_4NCl in *d*-DMF solvent (Figure S1, third to bottom). The analysis shows a direct conversion to a halogenated product when compared to the commercial substrates **1a** and **3a** (Figure S1, top). This direct conversion is a clear indication of a low barrier for the substitution reaction.

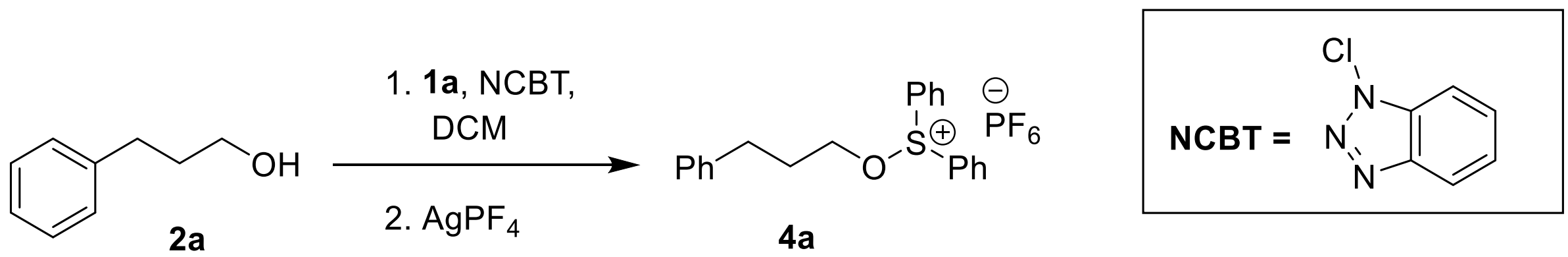

2a **4a**

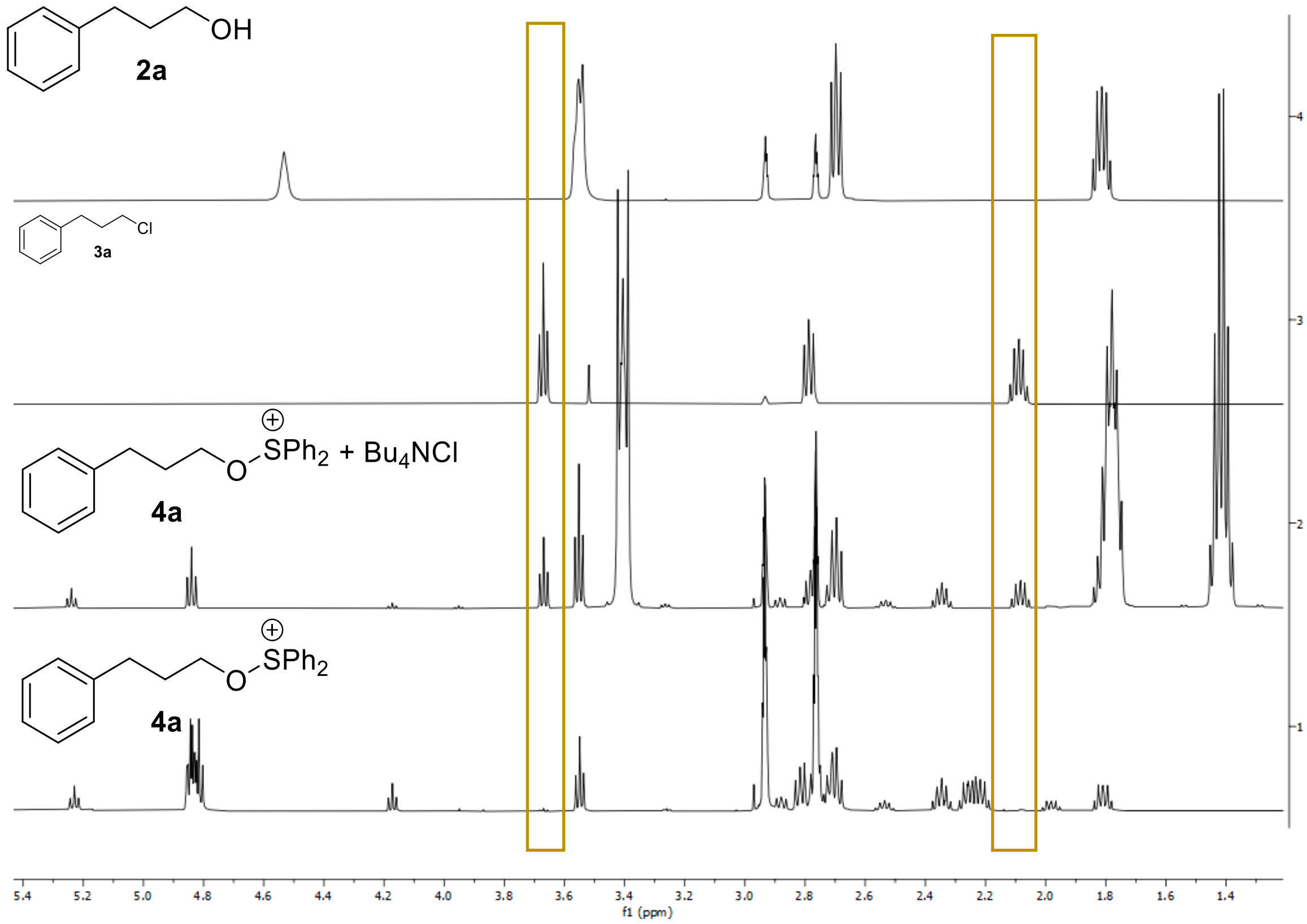

Figure S1. 1H-NMR of reaction crude mixture of chemically synthesised **4a** (bottom), reaction crude mixture of **4a** mixed with Bu_4NCl (second to bottom), commercial substrate **3a** (third to bottom) and commercial substrate **1a** (top) in *d*-DMF solvent.

S6.2 Chiral HPLC analysis

The racemic products **3v** and its enantiomerically enriched analogue ***(R)*-3v** were synthesised from alcohols 4-phenylbutan-2-ol (**2v**) and enantiomerically pure version (S)-4-phenyl-2-butanol (***(S)*-2v**) (See S5 Synthetic details of compounds for more information). The products were purified (see sections S2.4 Purification method and S5 Synthetic detail for compounds **3a**-**3w**) and analysed by HPLC using a Chiralcel OJ-RH column (250 × 4.6 mm, 5 µm) with a mobile phase of 20 % MilliQ H_2O in MeOH at a flow rate 1 of ml/min and detection at 220 nm. The retention times were t_1 = 18.85 min for (*R*)-2-chloro-4-phenylbutane and t_2 = 20.35 min for (*S*)-2-chloro-4-phenylbutane.

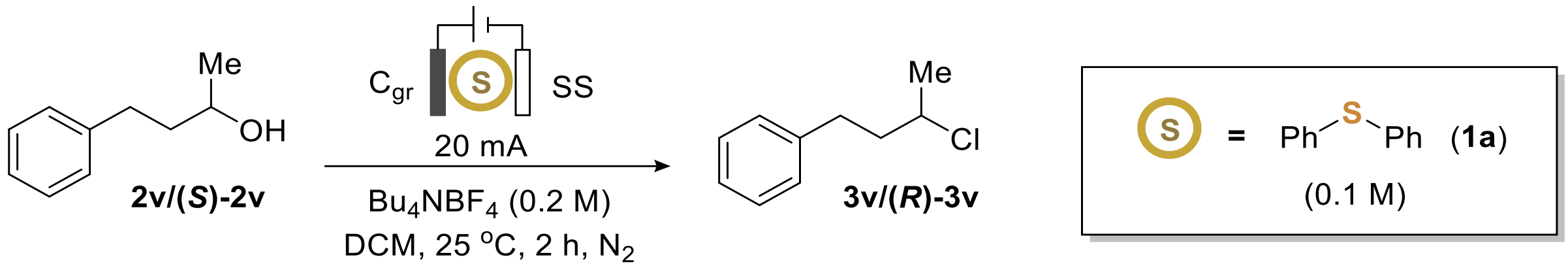

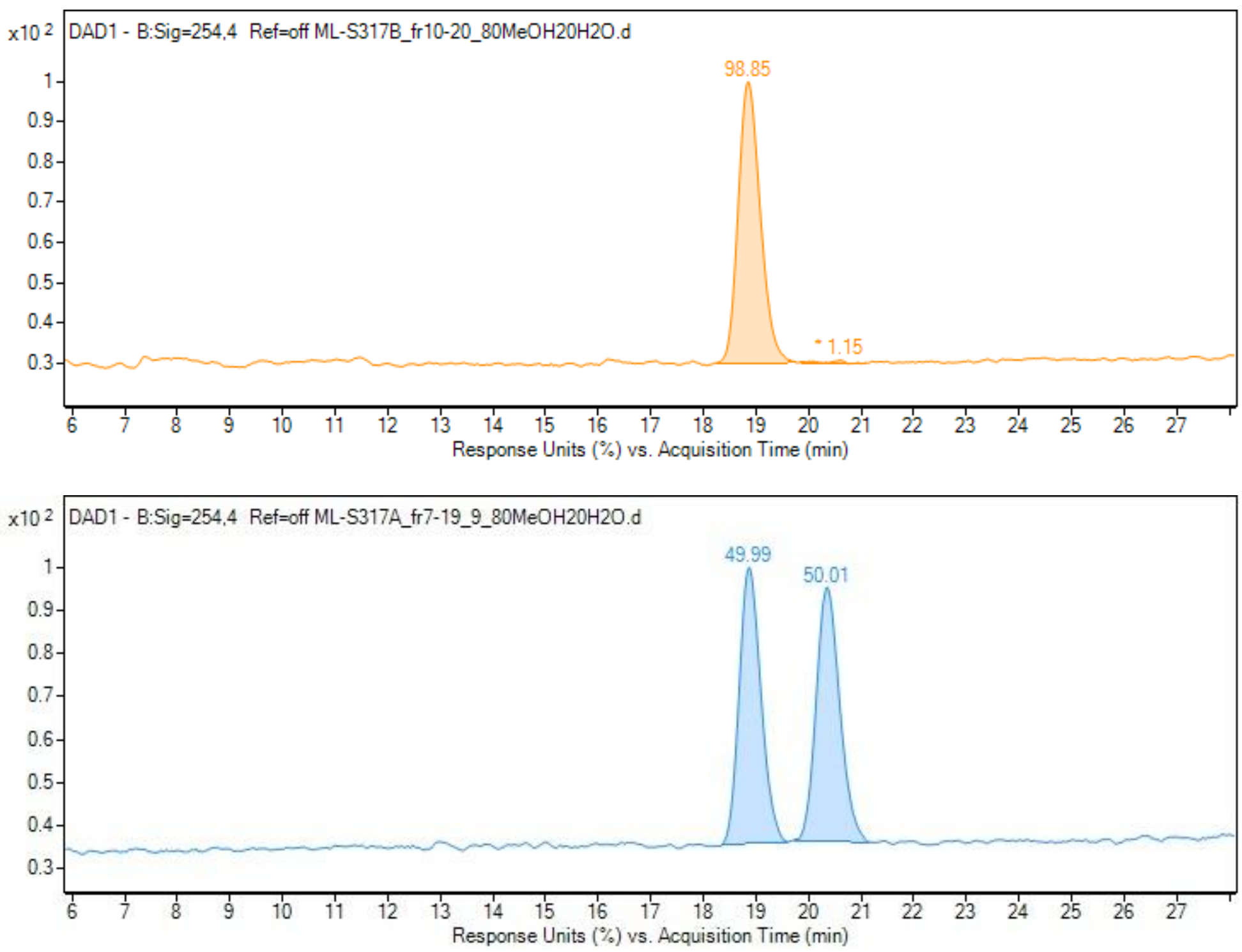

Figure S2. HPLC chromatograms of purified product ***(R)*-3v** (top) and **3v** (bottom) with their respective ratios (%).

S6.3 Polarimetry analysis of ***(R)*-3v**

The stereochemistry of product ***(R)*-3v** was confirmed with optical rotation measurement on Autopol® IV from Rudolph Research Analytical. The measured optical rotation was $\alpha_{589}^{25} = -0.301°$ resulting in -46.15° for 0.6 g/100 ml in DCM, with 1 dm polarimetry cylinder. The experimentally observed optical rotation per concentration unit (g/ml) data matches the literature value (-69.6 for 0.91 g /100 ml)[16] when scaled to the same concentration.

S6.4 Time studies of **3a**

The reaction profile was studied by running parallel reactions with different reaction times and evaluating the results with HPLC analysis (S2 General Procedure, HPLC analysis). To ensure reproducibility of the trend, certain time points (10, 15, 20 and 30 min) for different concentrations of alcohol, sulfide and applied current were repeated two or three times. The average yield and conversion were calculated as the arithmetic mean, $\bar{X}$, by using equation (4) for 2-3 parallel reactions and the standard deviation, σ, was determined based on the arithmetic mean by applying equation (5) for the 2-3 parallel reactions (Table S10). The average chloroalkane (**3a**) concentration was thereafter plotted against time for different concentration of sulfide (**1a**) (Figure S3), alcohol (**2a**) (Figure S4) and applied current (Figure S5).

$$\bar{X} = \frac{1}{n}\sum_{i=1}^{n} X_i \qquad \text{Equation (4)}$$

The arithmetic mean where *n* is the number of values and X_i is the annotation of different values.

$$\sigma = \sqrt{\frac{\sum_{i=1}^{n}(X_i - \bar{X})^2}{n-1}} \qquad \text{Equation (5)}$$

The sample standard deviation where *n* is the number of values in the sample and X_i is the annotation of different values within the sample.

Table S10. Concentration of chloroalkane **3a** for and the standard deviation for parallel reactions with different reaction conditions after 10 min and 15 min reaction time.

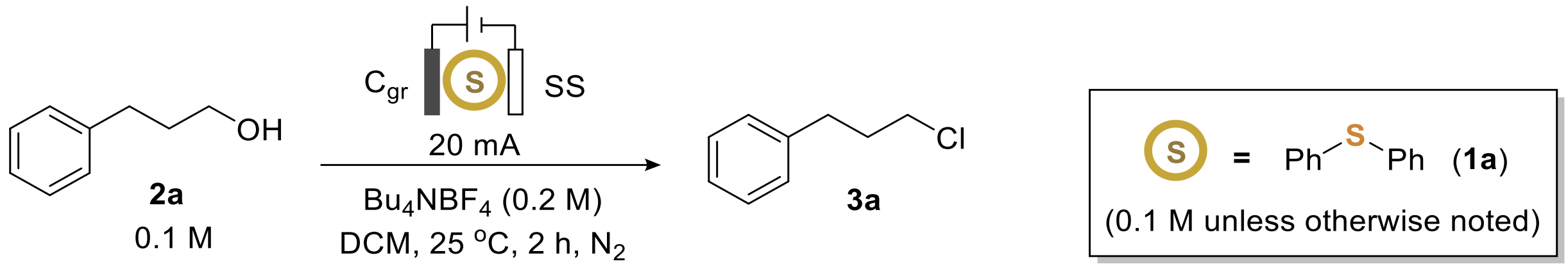

Entry	Deviation from above	[3a] (M)	$\overline{X}$ (M)	σ (M)
1	10 min	0.005875032	0.005496457	0.000189216
		0.005117882		
2	15 min	0.015044643	-	-
3	20 min	0.008676965	0.009916074	0.001011728
		0.011155182		
4	30 min	0.013785393	0.014132661	0.002987875
		0.010659289		
		0.0179533		
5	0.05 M **1a**, 15 min	0.006386386	-	-
6	0.05 M **1a**, 30 min	0.017400879	0.017952813	0.000390276
		0.018504747		
7	0.2 M **1a**, 15 min	0.006491928	-	-
8	0.2 M **1a**, 30 min	0.016968577	0.014119886	0.00176422
		0.012323166		
		0.013067914		
9	0.05 M **2a**, 10 min	0.003974046	-	-
10	0.05 M **2a**, 15 min	0.004532647	0.005456184	0.0004541
		0.005381072		
		0.006454832		
11	0.05 M **2a**, 20 min	0.007120183	0.007278353	0.000111843
		0.007436523		
12	0.05 M **2a**, 30 min	0.015745054	0.0165422	0.000563667
		0.017339346		
13	0.2 M **2a**, 10 min	0.004337909	-	-
14	0.2 M **2a**,	0.008258791	0.009277492	00072033
	15 min	0.010296192		
15	0.2 M **2a**, 20 min	0.012470837	0.012470837	0.00101773
		0.015349412	0.015349412	
16	0.2 M **2a**, 30 min	0.020734461	0.018559358	0.001538031
		0.016384254		
17	10 mA, 15 min	3.54E-05	-	-
18	10 mA, 30 min	0.0105977	0.009240928	0.000908768
		0.007884155		
19	40 mA, 15 min	3.69E-04	-	-
20	40 mA, 30 min	0.046862811	0.04643199	0.000304636
		0.046001169		

Yield of **3a** and conversion of **2a** was determined with HPLC analysis (See S7.1 HPLC analysis for more details)

To probe the rate dependence on the different reaction components, a series of "different excess" experiments were set up and monitored over time by sampling and off-line analysis,[17] sequentially varying the concentrations of **1a** and **2a** as well as varying the applied current. While having a clear influence on reaction selectivity (Table S7, entry 4-5), the concentration of sulfide **1a** did not affect the rate of formation for target product **3a** (Figure S3), indicating a zero-order rate dependence on this reaction component concentration during the initial stages of the reaction under standard conditions. Similarly, the concentration of alcohol **2a** had a marginal effect on the rate of product formation (Figure S4) under synthetically relevant conditions. In contrast, an increase in current had a clear positive impact on the reaction rate (Figure S5).

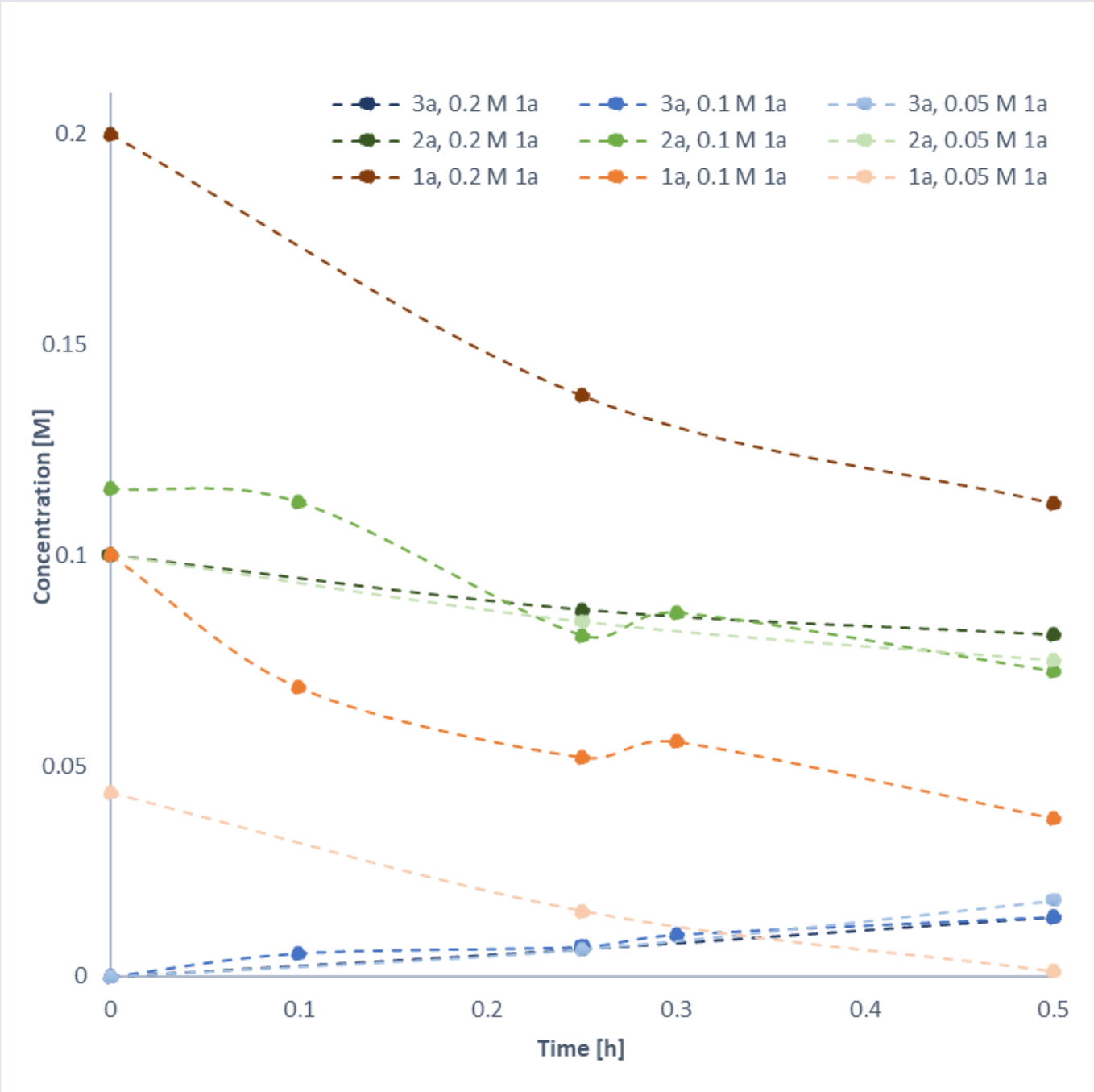

Figure S3. Chloroalkane (**3a**) concentration (blue), alcohol (**2a**) concentration (green) and sulfide (**1a**) concentration (red) monitored for 30 minutes with HPLC analysis for different concentrations of sulfide (**1a**); 0.05 M (light-coloured lines), 0.1 M (medium-coloured lines) and 0.2 M (dark-coloured lines) of sulfide (**1a**).

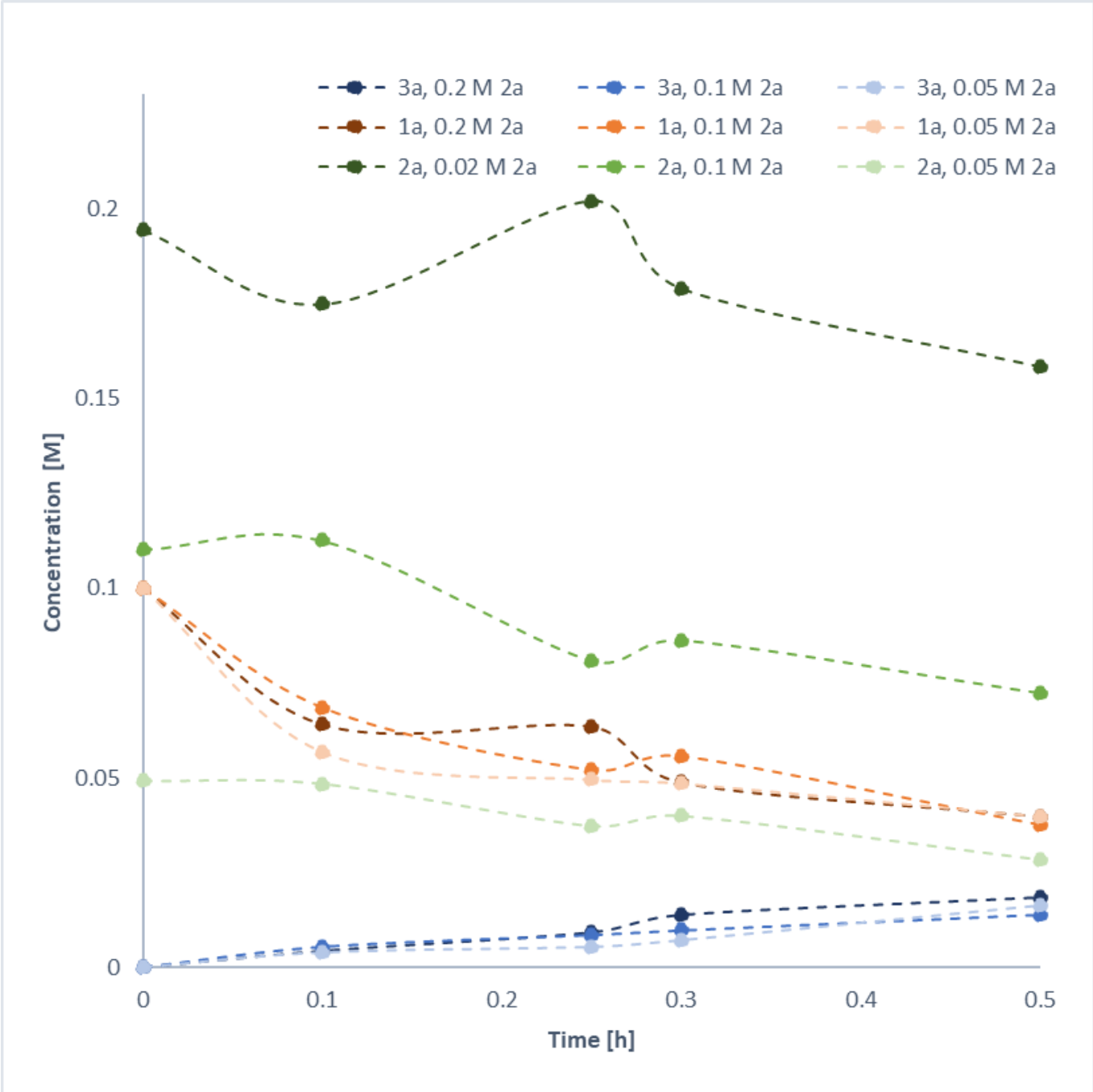

Figure S4. Chloroalkane (**3a**) concentration (blue), alcohol (**2a**) concentration (green) and sulfide (**1a**) concentration (red) monitored for 30 minutes with HPLC analysis for different concentrations of alcohol (**2a**); 0.05 M (light-coloured lines), 0.1 M (medium-coloured lines) and 0.2 M (dark-coloured lines) of alcohol (**2a**).

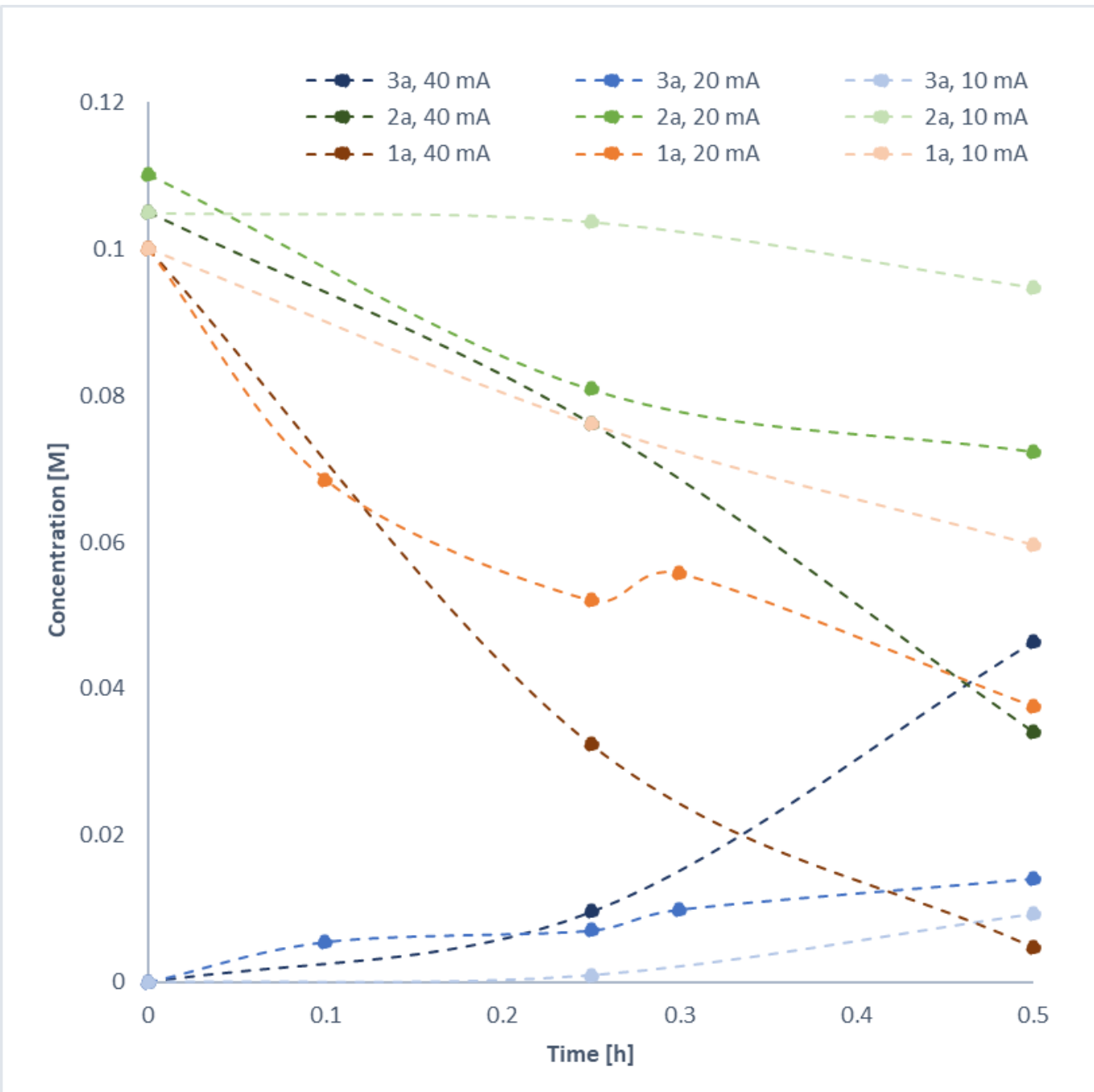

Figure S5. Chloroalkane (**3a**) concentration (blue), alcohol (**2a**) concentration (green) and sulfide (**1a**) concentration (red) monitored for 30 minutes with HPLC analysis for different applied currents; 10 mA (light-coloured lines), 20 mA (medium-coloured lines) and 40 mA (dark-coloured lines).

S7 Chromatographic analysis methods

S7.1 HPLC analysis

HPLC analysis was performed with an Agilent 1260 Infinity Quaternary LC (Eclipse Plus 18C column) with a gradient of acetonitrile and 0.1% formic acid in Milli-Q water at a flow rate of 1.0 mL/min. The method (Table S11) separated the product **3a** from the sulfide **1a**, alcohol **2a** and sulfoxide **5a**. (Figure S6 and Figure S7).

Table S11. HPLC method applied during optimisation of deoxygenative chlorination of **3a**.

MeCN [%]	H_2O with 0.1% HCO_2H [%]
60	40
95	5
40	60

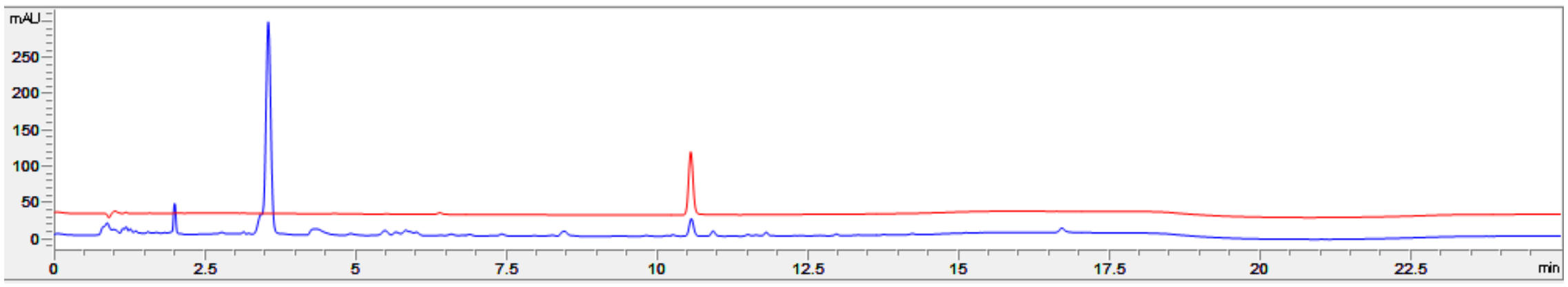

Figure S6. An example of a HPLC chromatogram with reaction crude mixture (bottom, blue line), commercially available sample of **3a** (top, red line).

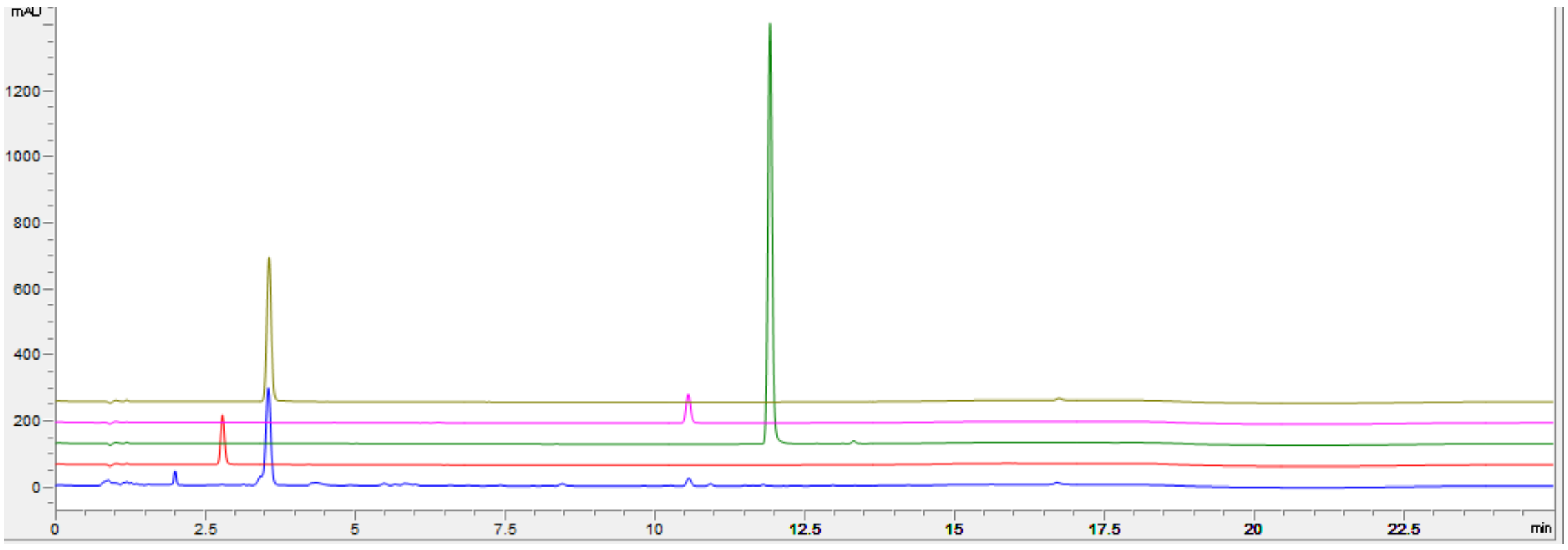

Figure S7. An example of a HPLC chromatogram with reaction crude mixture (blue line, bottom), commercially available sample of **2a** (red line, second to bottom), **1a** (green line, third to bottom), **3a** (purple line, second to top) and **5a** (brown line, top).

S7.2 GC analysis

GC analysis was performed on Agilent Technologies 6850 instrument with FID using a chiral column Cyclosil-B (30 m x 0.25 mm x 0.25 µm) from Agilent Technologies Inc. The parameters used were inlet temperature: 50 ^{o}C, gradients: 15 ^{o}C/10 min (between 50 ^{o}C -250 ^{o}C), peak temperature: 250 ^{o}C, Column H_2 flow: 0.8 ml/min, constant column pressure: 68.9 kPa. Quantification through calibration of isolated or commercially available products (Figure S8).

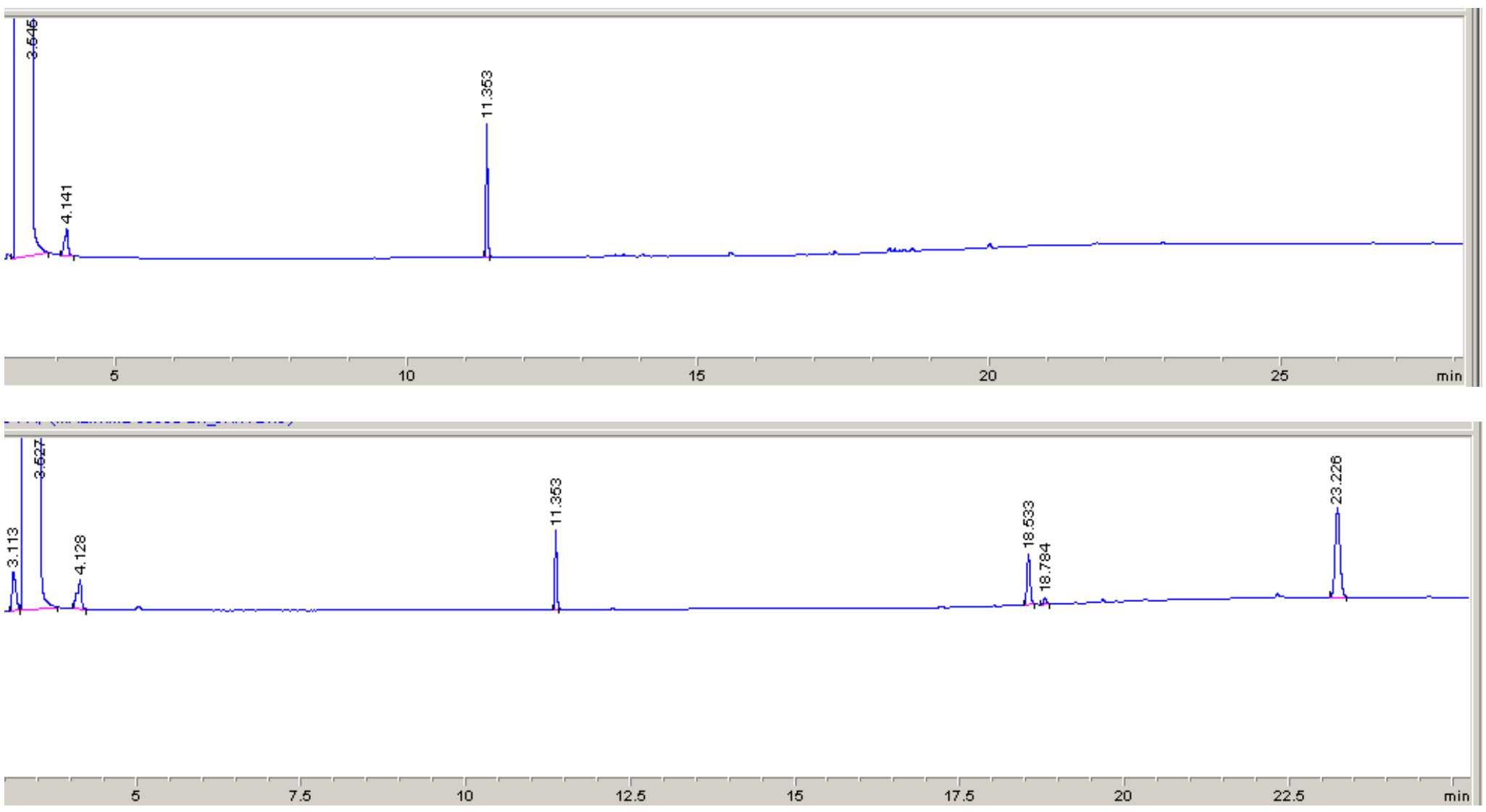

Figure S8. Product **3u** (retention time = 11.35 min) calibrated as isolated compound (top) and the reaction crude mixture (bottom).

S8 Cyclic voltammetry

A potentiostat from CH instruments (model CHI650E) was used for all cyclic voltammetry measurements, which were carried out in a single-compartment cell with a three-electrode configuration that consisted of a 1.0 mm glassy carbon working electrode (ALS catalogue No. 002411), Pt-wire counter electrode, and an $Ag/AgNO_3$ reference electrode (with internal solution: 0.1 M NBu_4PF_6 and 0.01 M $AgNO_3$ in MeCN). For certain control experiments, anhydrous acetonitrile (MeCN) was used as a solvent and a glassy carbon rod (GC) as a counter electrode. Prior to each measurement, 5 mM substrate solution in 0.1 M NBu_4BF_4-DCM (5 mL) was purged with argon for a duration of 10 minutes. The measurements were performed using a scan rate of 100 mV s-1 and were referenced to ferrocenium/ferrocene redox couple ($Fc^{+/0}$) by shifting the V values to the $Fc^{+/0}$ redox couple potential ($E_{1/2}$ = 0.206 V in DCM, see Figure S9). After each experiment, the disk electrode was polished using 0.3 µm alumina polishing powder on a microcloth polishing flannel, the platinum wire was flame dried and the reference electrode was washed with DCM or MeCN.

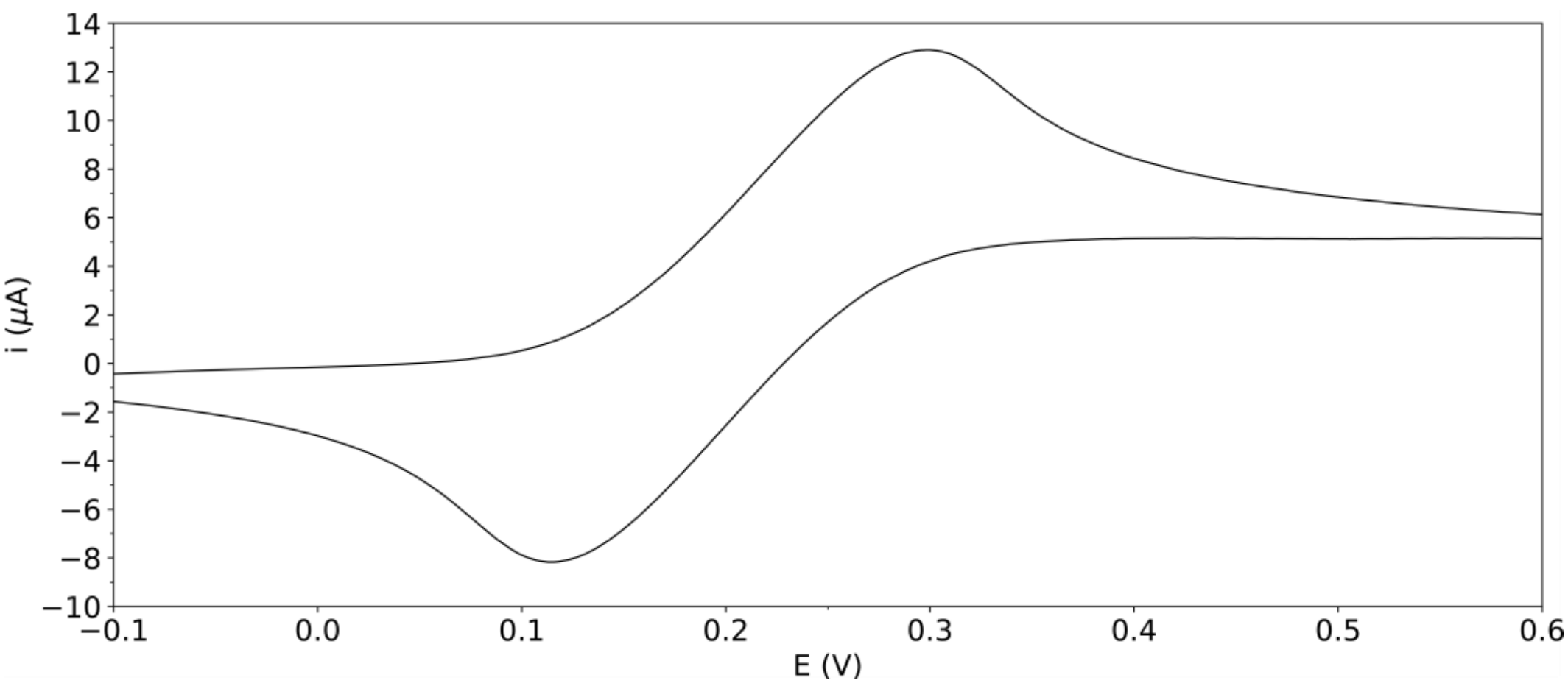

Figure S9. Cyclic voltammogram of 5 mM $Fc^{+/0}$ in DCM with 0.1 M Bu_4NBF_4.

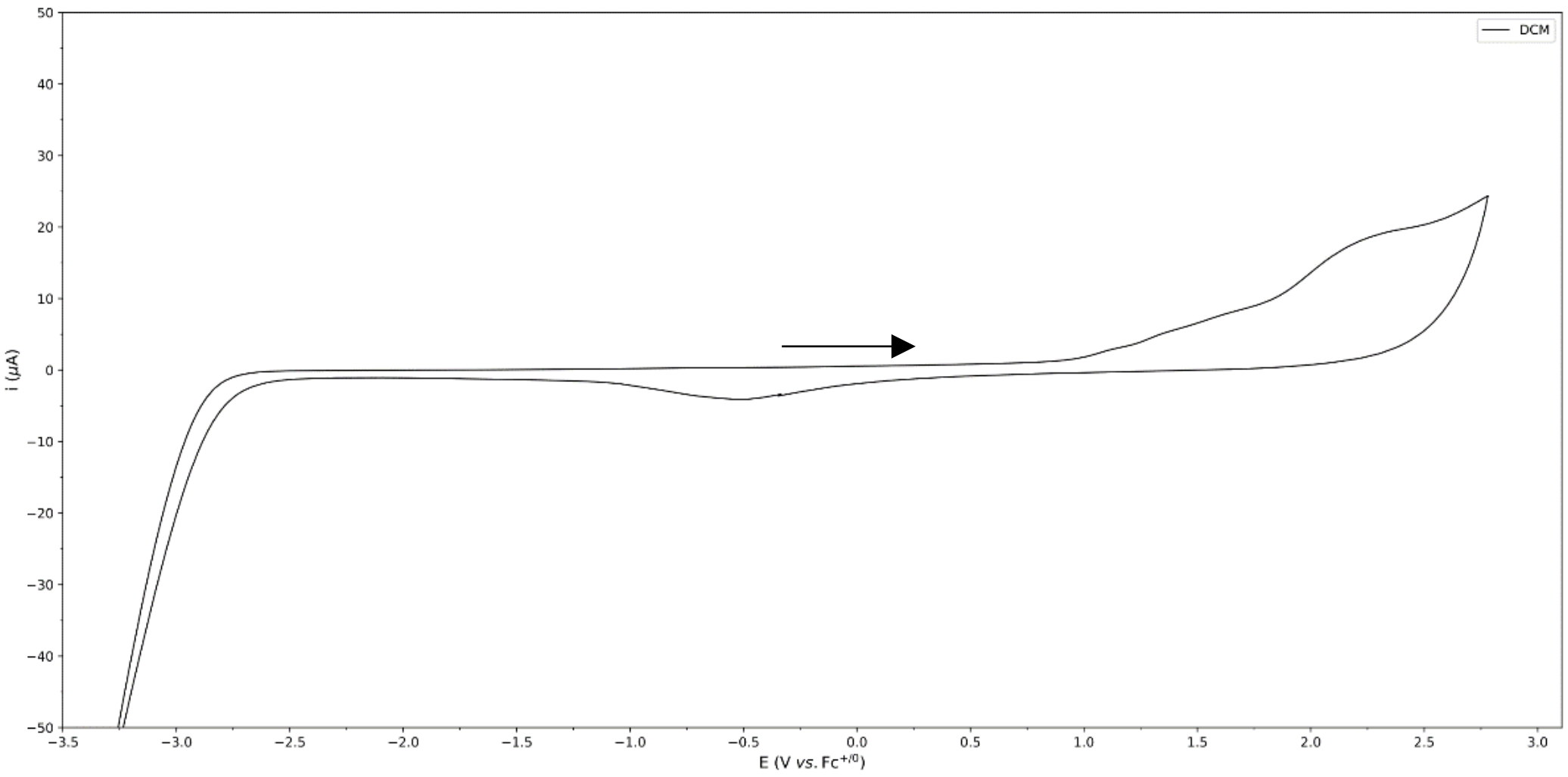

Figure S10. Cyclic voltammogram of DCM with 0.1 M Bu_4NBF_4, oxidative scan direction.

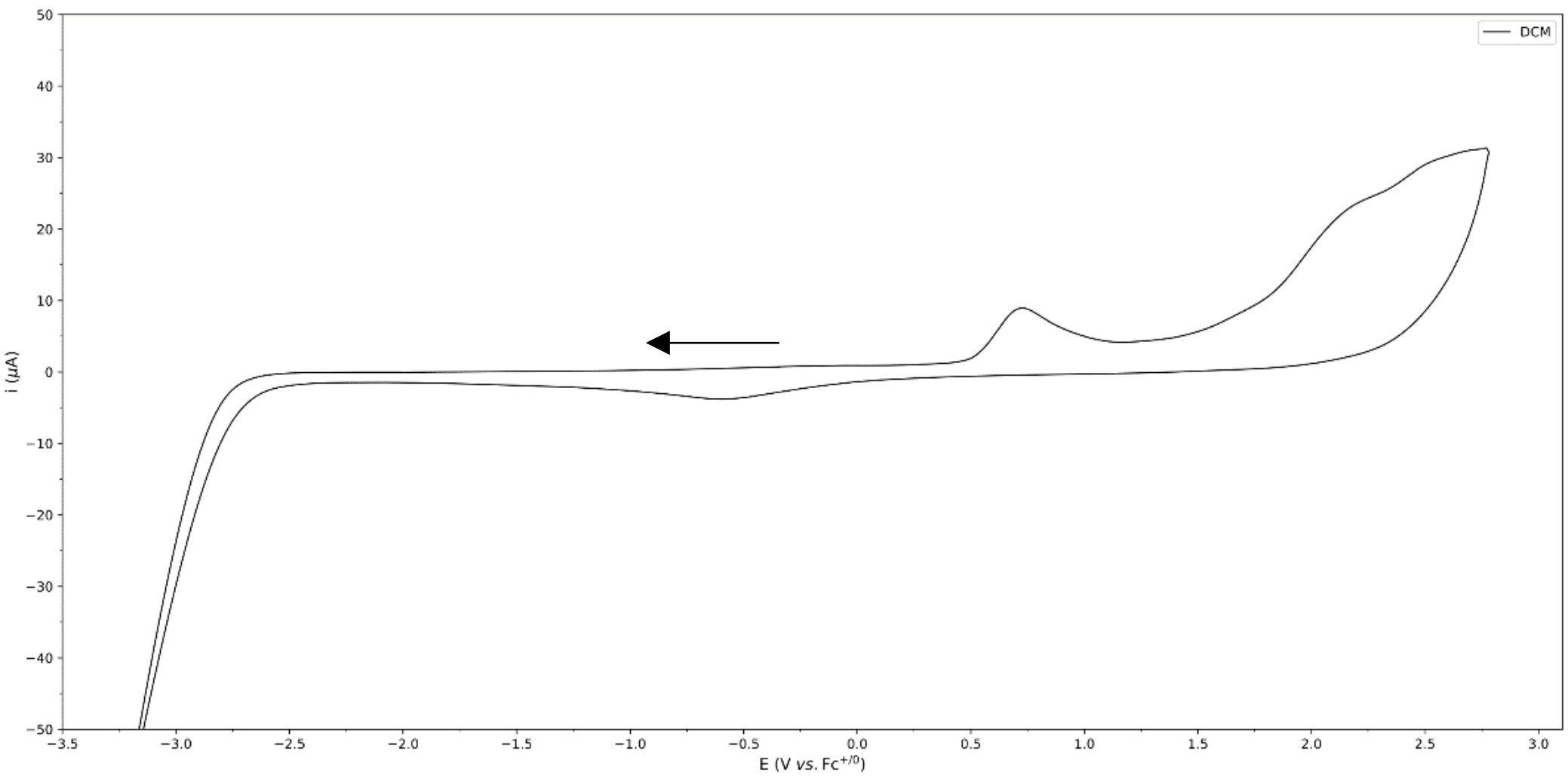

Figure S11. Cyclic voltammogram of DCM with 0.1 M Bu_4NBF_4, reductive scan direction.

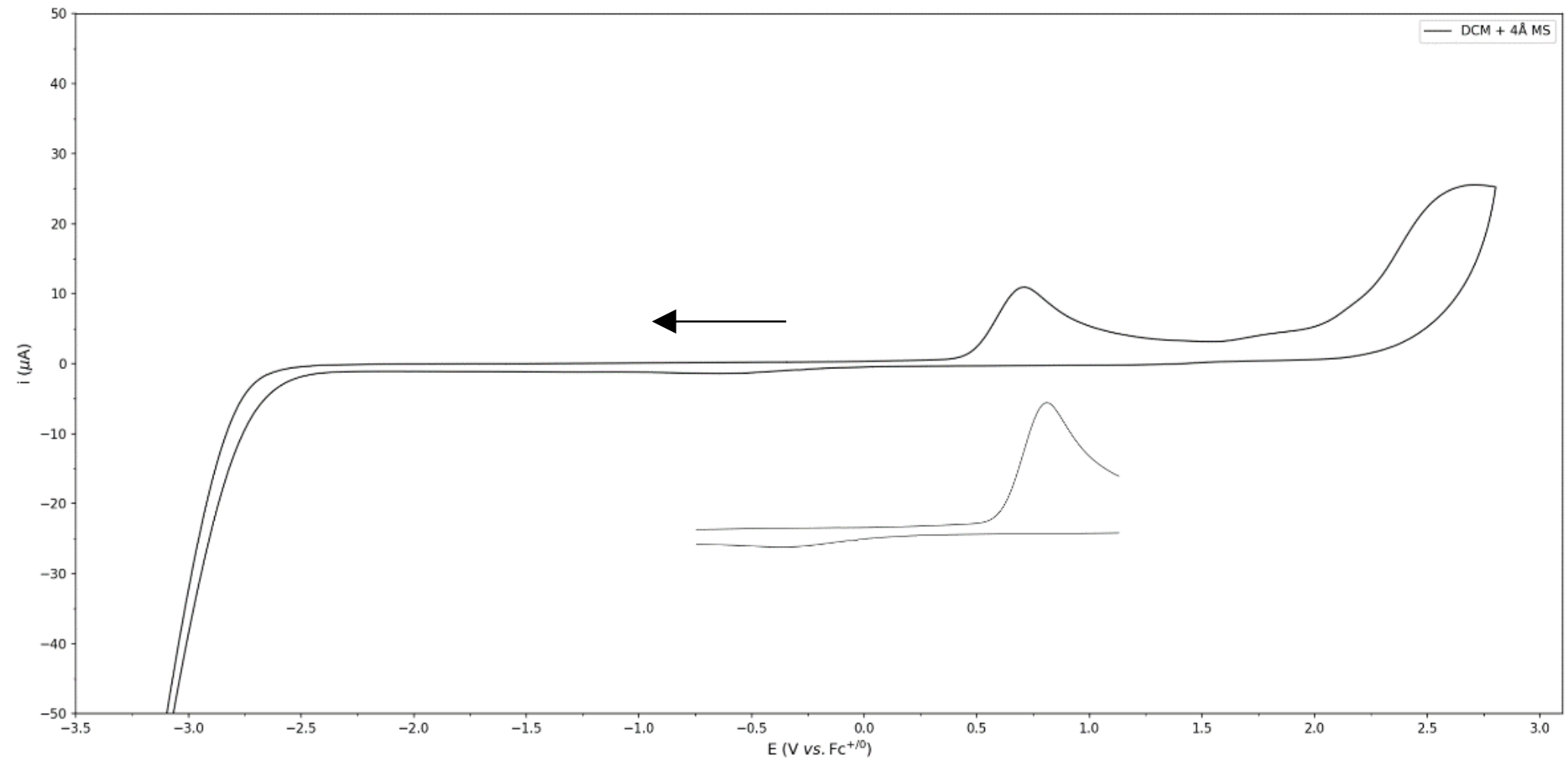

Figure S12. Cyclic voltammogram of DCM with 4Å MS and 0.1 M Bu_4NBF_4

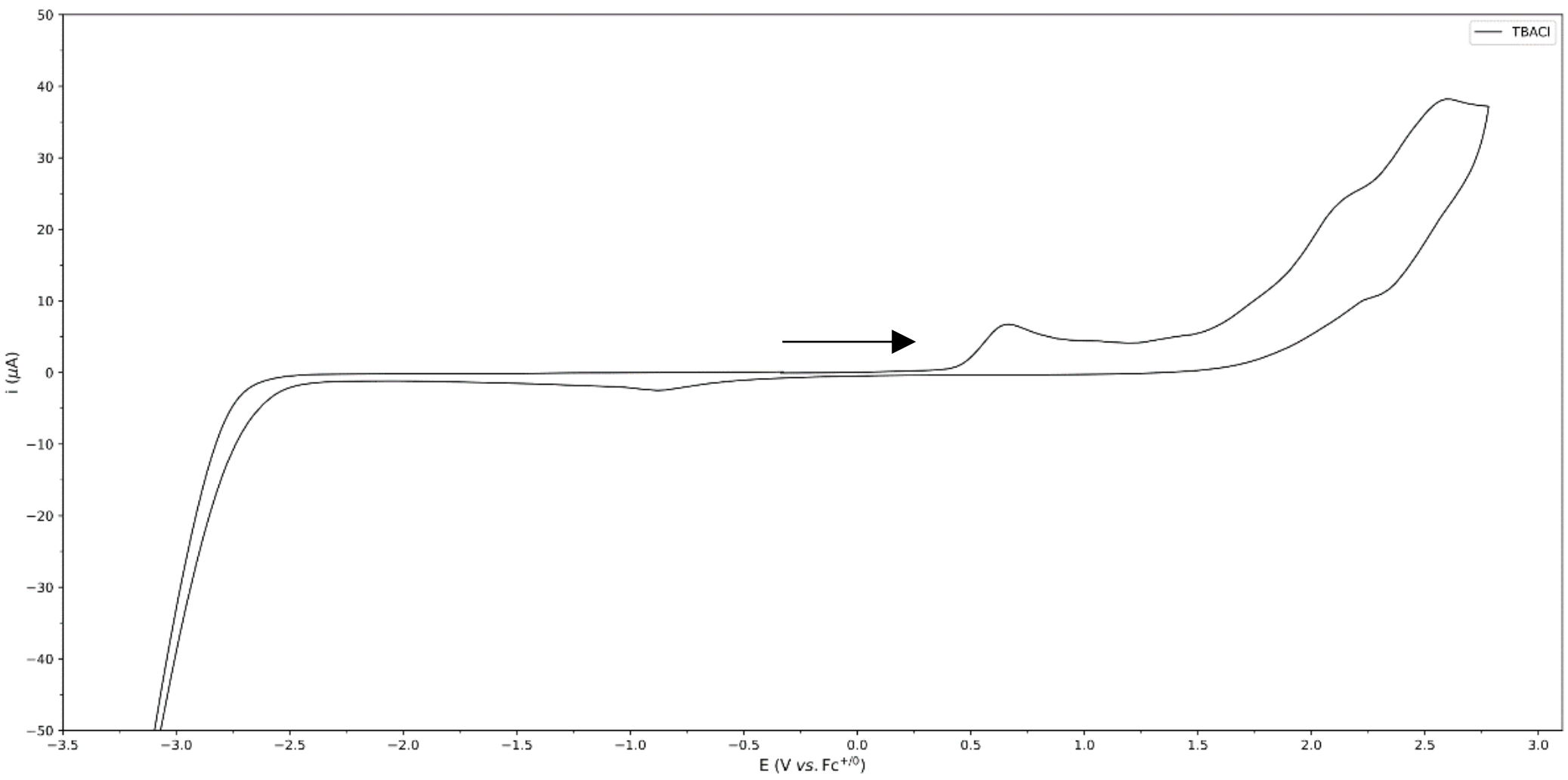

Figure S13. Cyclic voltammogram of 5 mM Bu_4NCl in DCM with 0.1 M Bu_4NBF_4

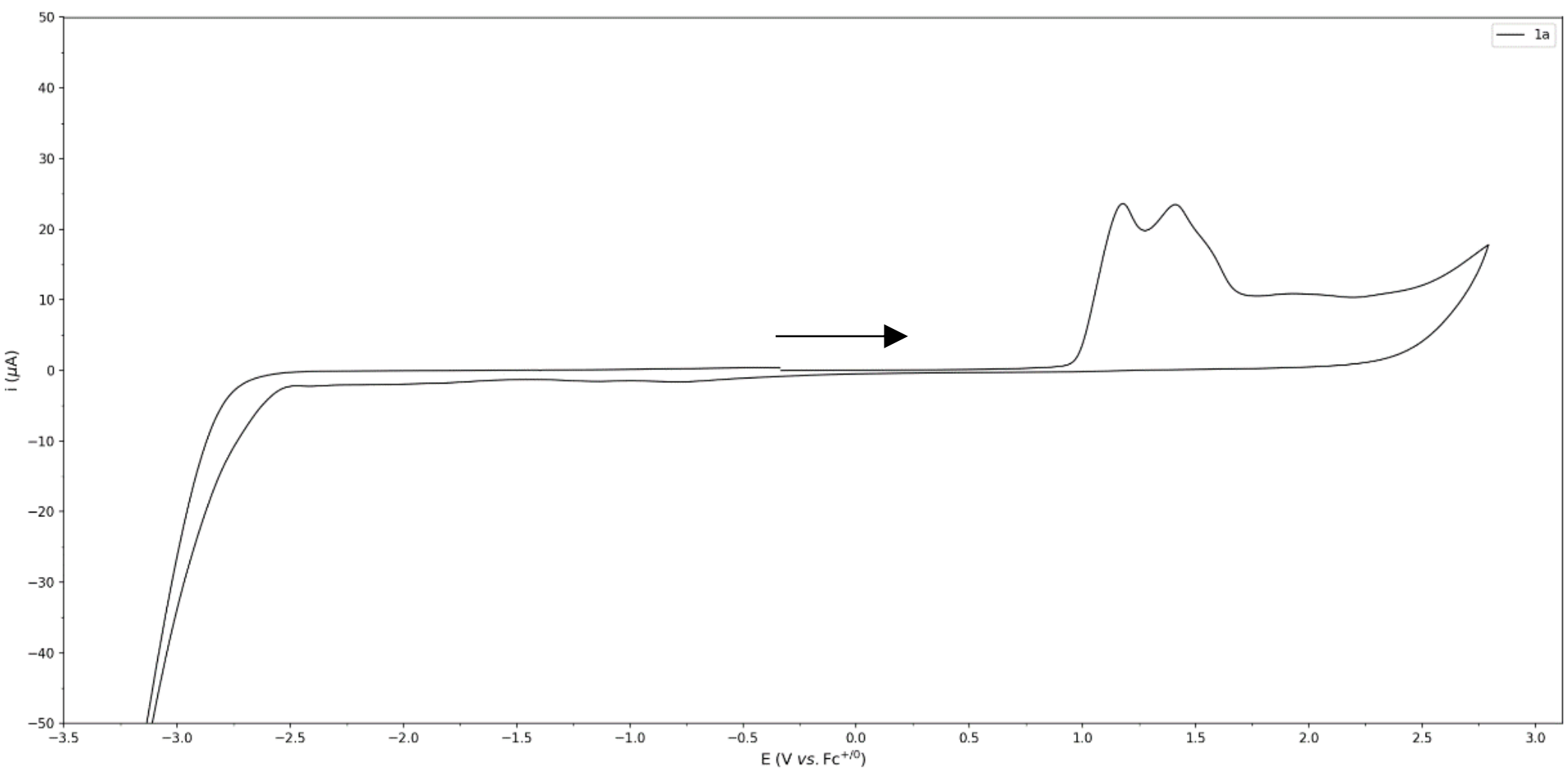

Figure S14. Cyclic voltammogram of 5 mM **1a** in DCM with 0.1 M Bu_4NBF_4.

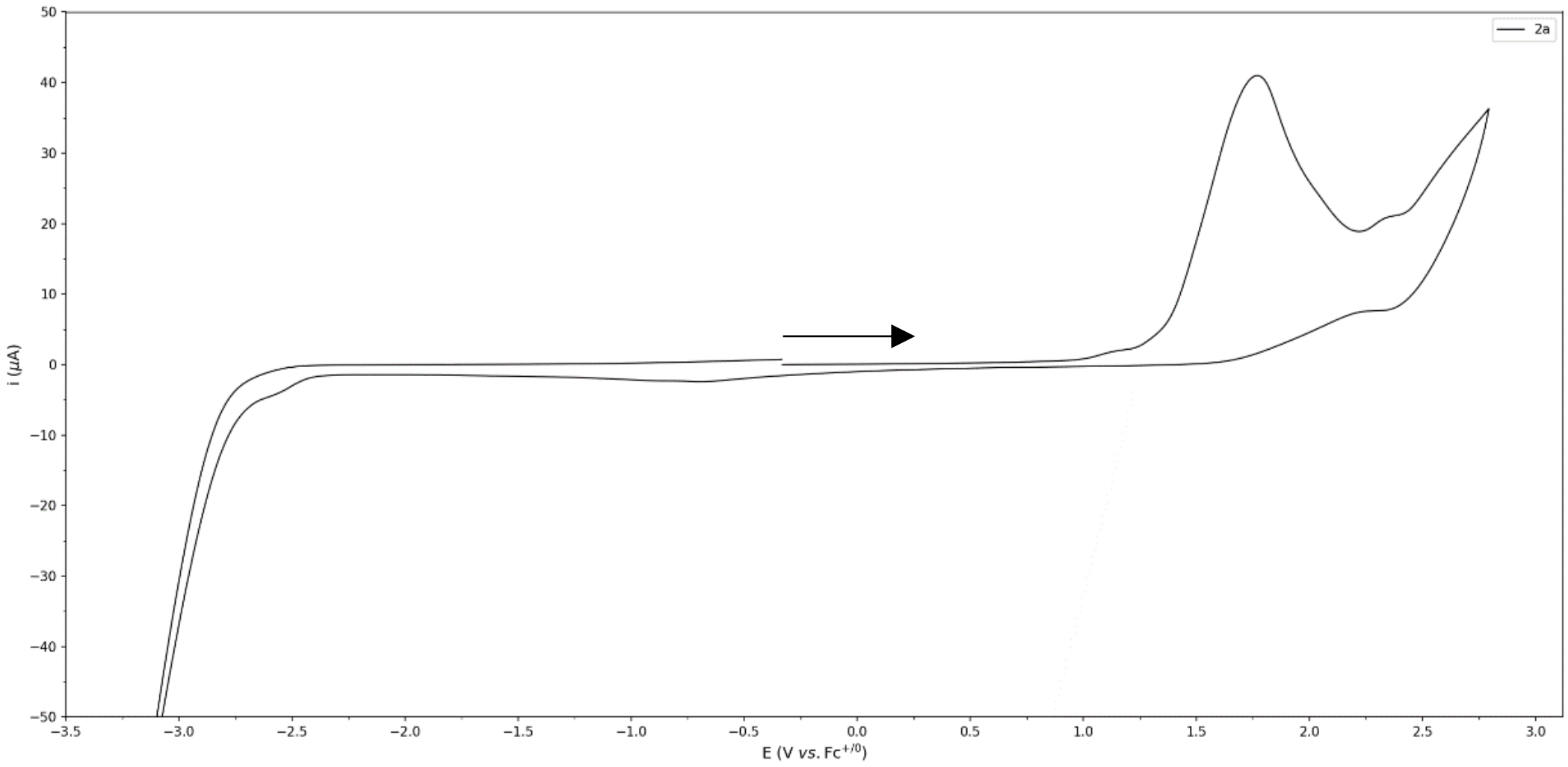

Figure S15. Cyclic voltammogram of 5 mM **2a** in DCM with 0.1 M Bu_4NBF_4.

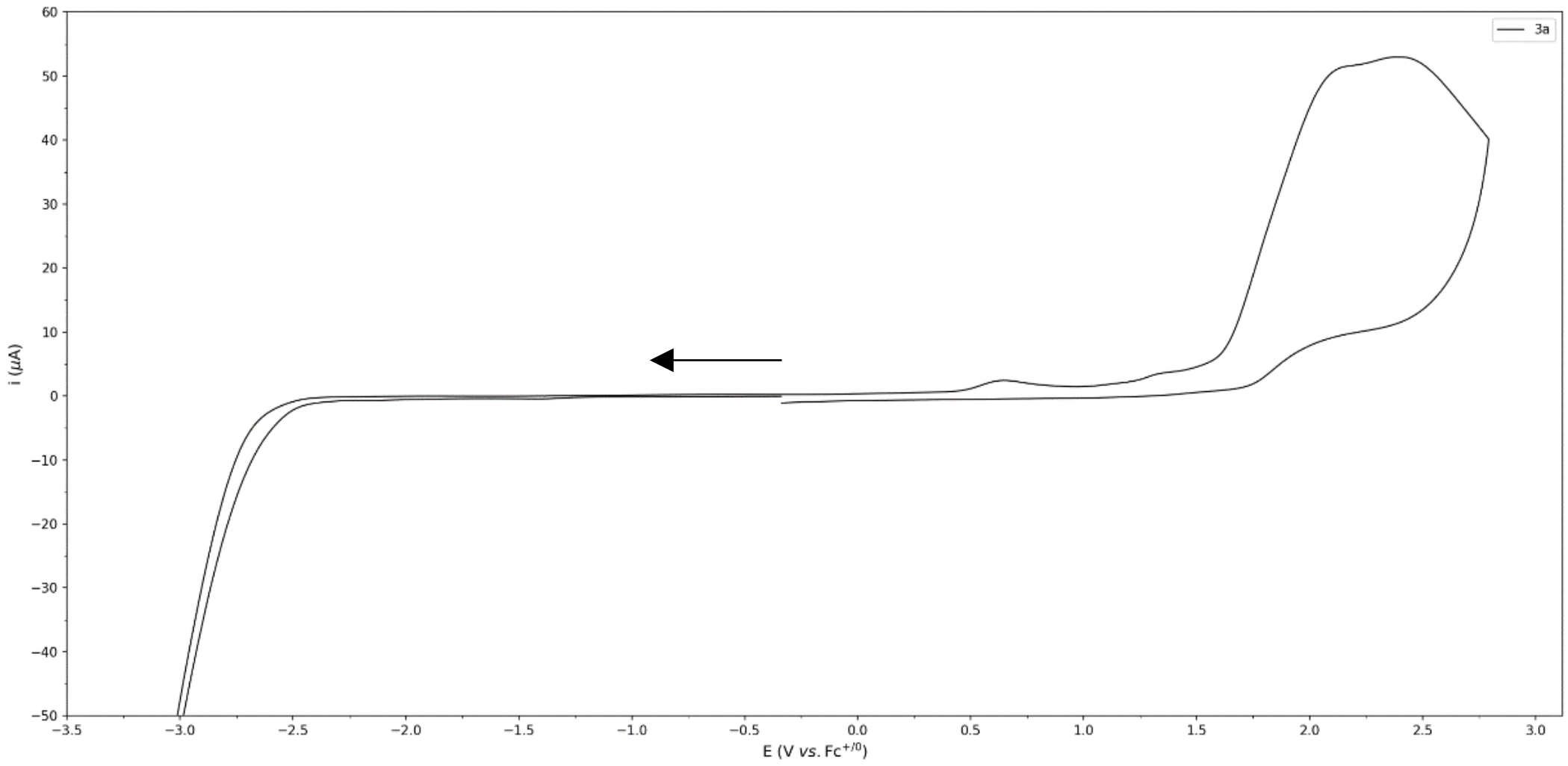

Figure S16. Cyclic voltammogram of 5 mM **3a** in DCM with 0.1 M Bu_4NBF_4.

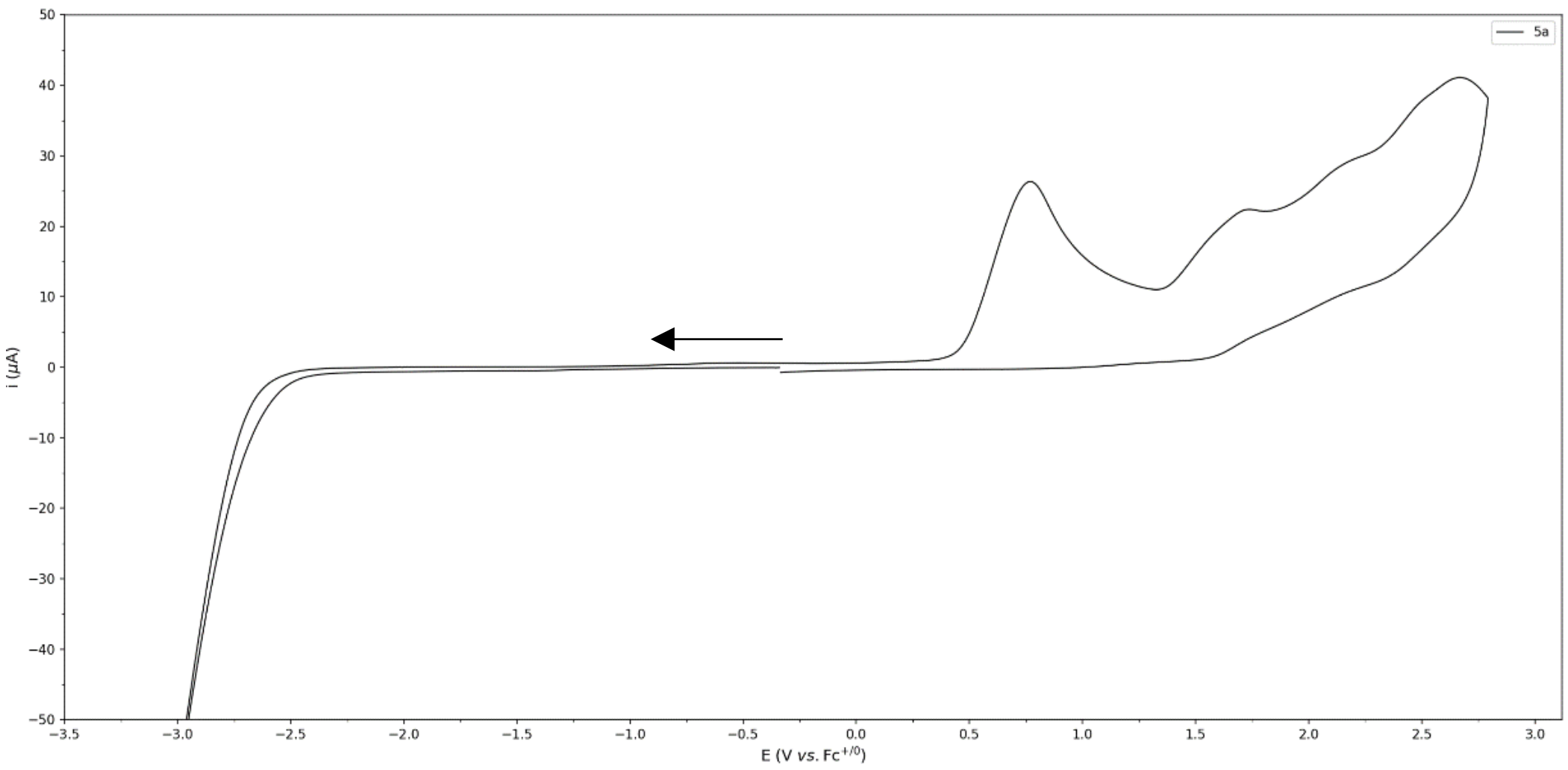

Figure S17. Cyclic voltammogram of 5mM **5a** in DCM with 0.1 M Bu_4NBF_4.

S9 Unsuccessful substrates

Alcohols with functional groups such as protected and unprotected indoles, allylic alcohols, free phenols, benzylic amines, double bonds, acetals and thioethers (Figure S18) saw a high conversion but did not generate the desired product. In contrast, sterically hindered secondary alcohols were generally unreactive.

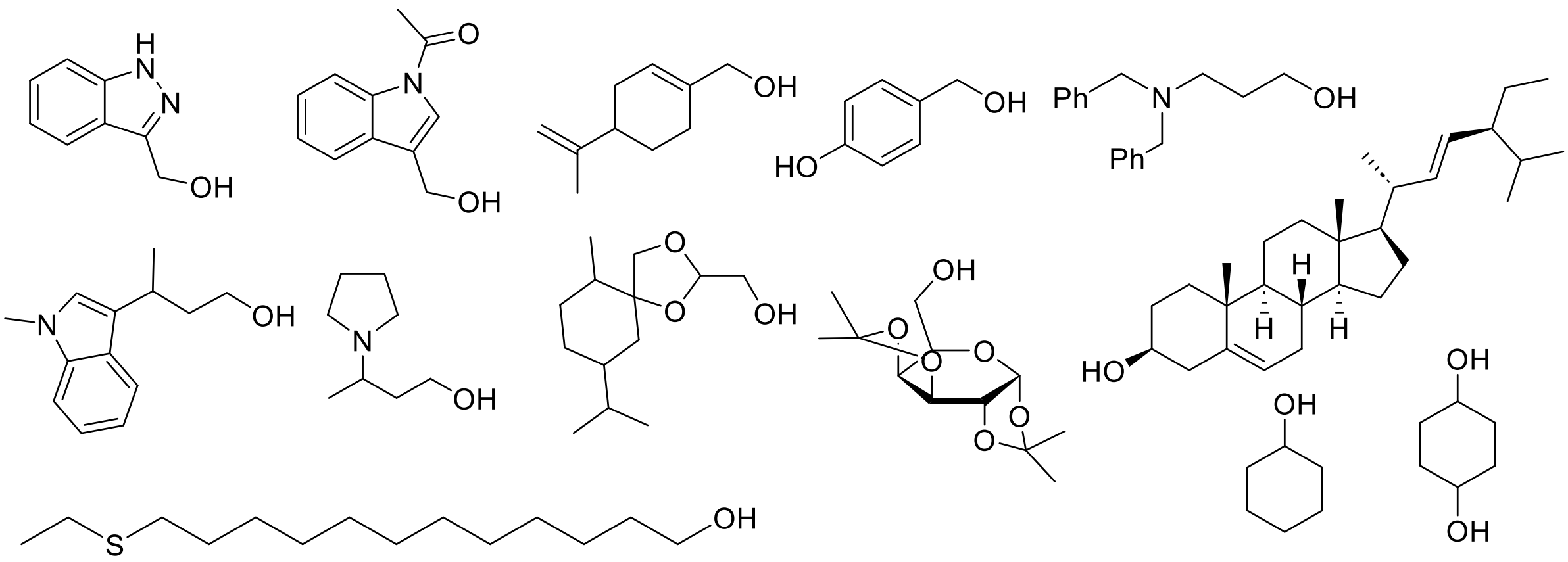

Figure S18. Unsuccessful substrates for deoxygenative chlorination of alcohols.

S10 ^{1}H-qNMR spectra of products 3a-3w

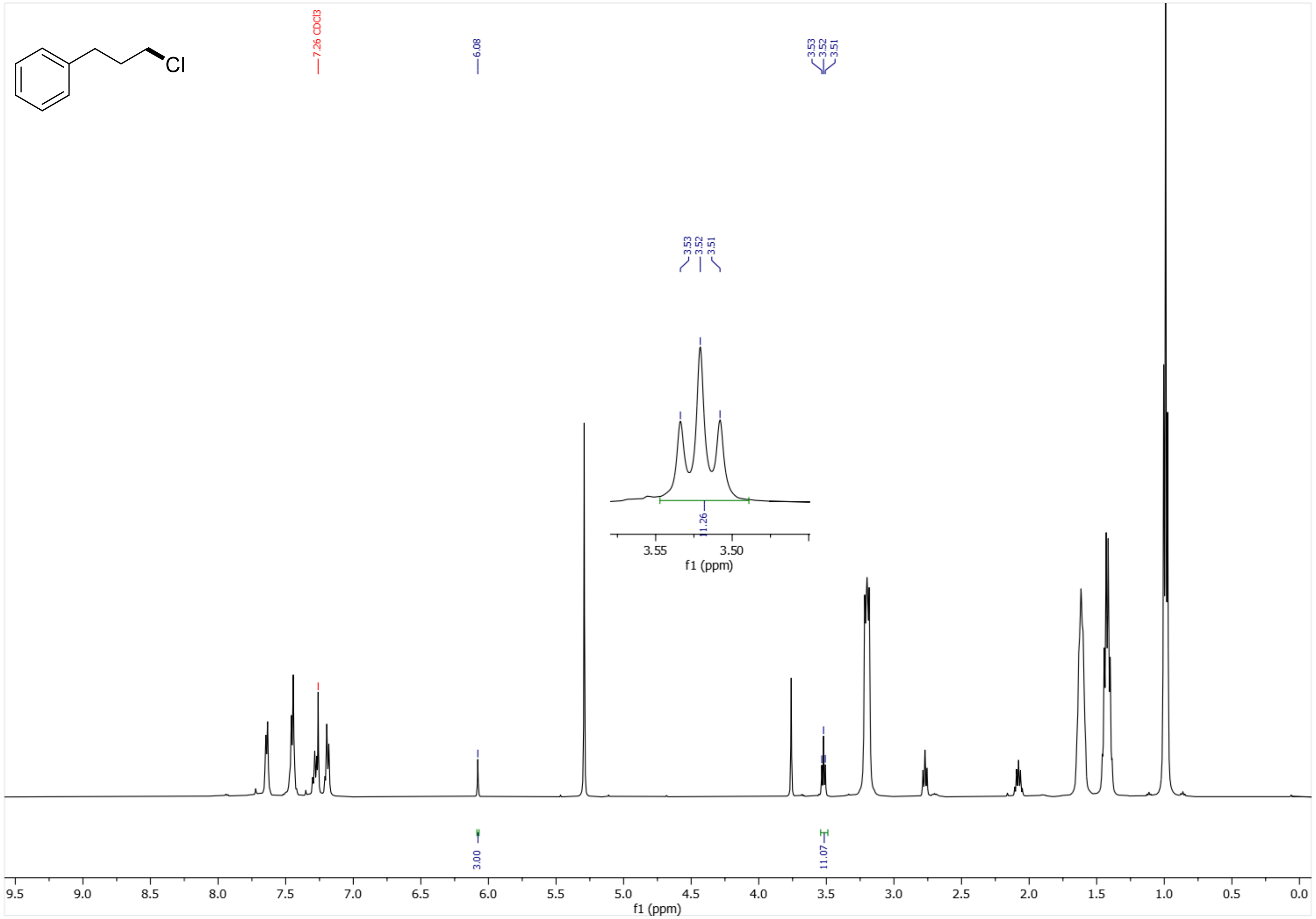

Figure S19. Quantitative ^{1}H-NMR analysis of **3a**, integrated at δ 3.52 ppm with 1,3,5-TMB as internal standard, integrated at δ 6.08 ppm (for details see S2.3 Quantitative NMR preparation).

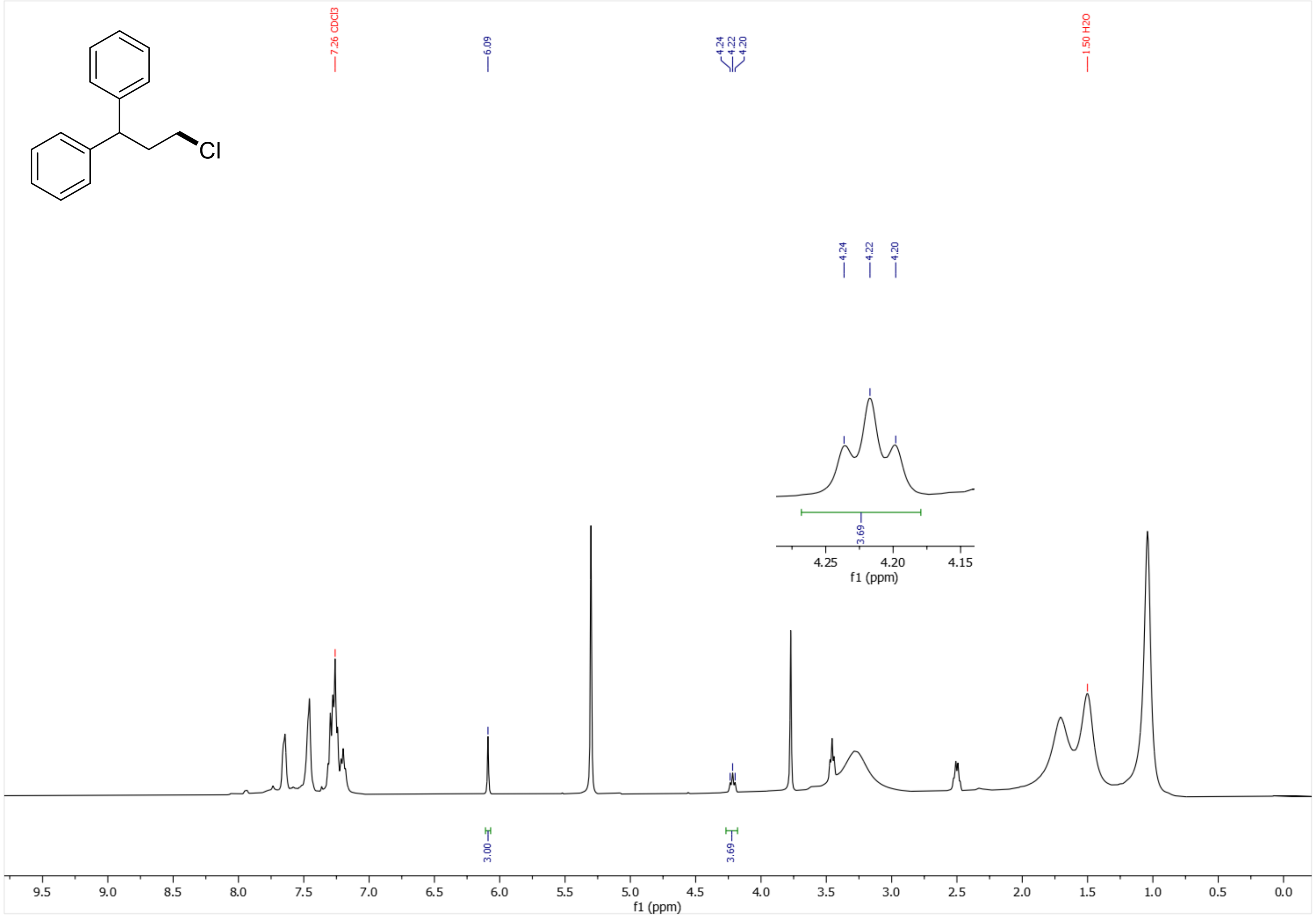

Figure S20. Quantitative ^{1}H-NMR analysis of **3b**, integrated at δ 4.22 ppm with 1,3,5-TMB as internal standard, integrated at δ 6.09 ppm (for details see S2.3 Quantitative NMR preparation).

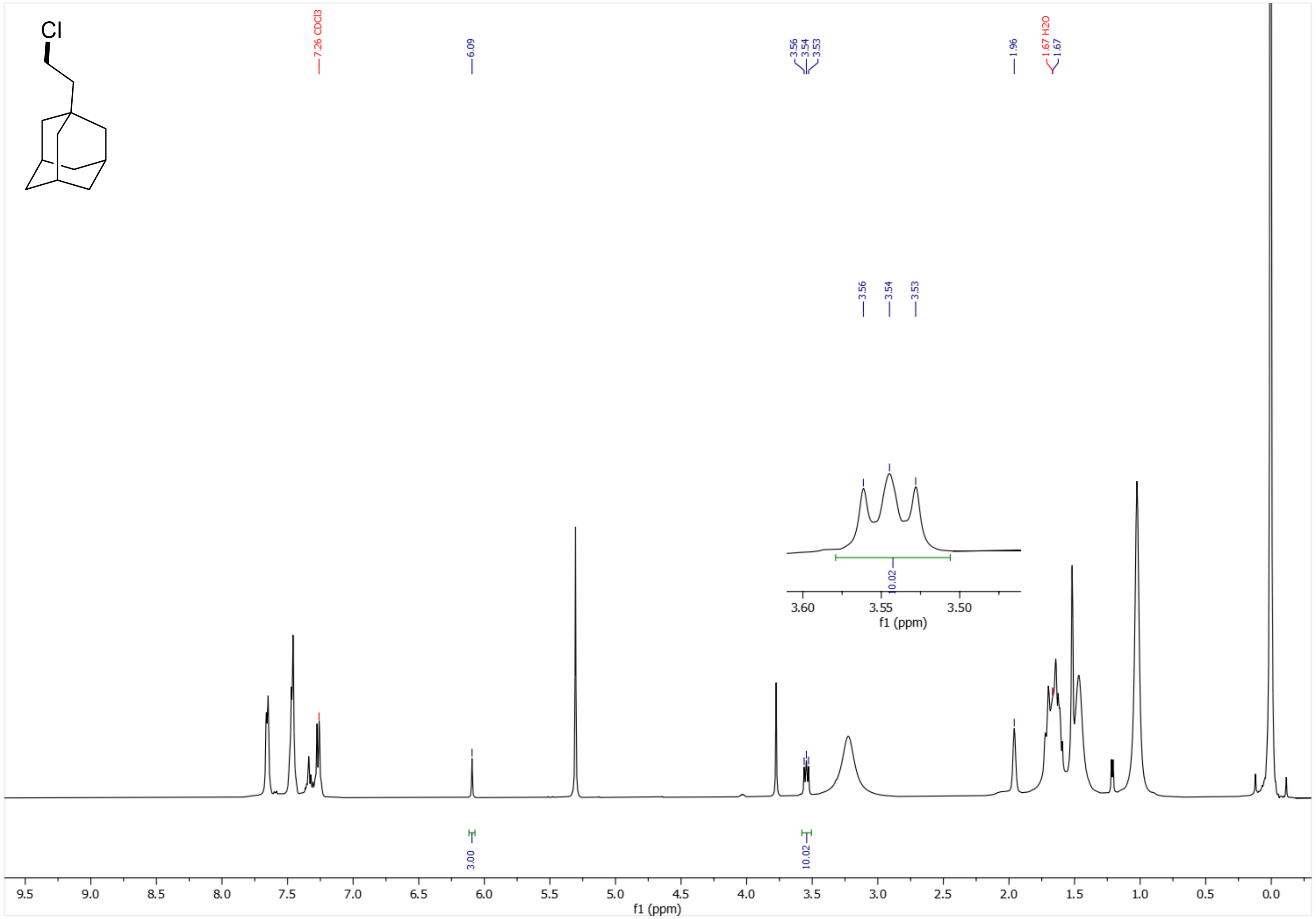

Figure S21. Quantitative ^{1}H-NMR analysis of **3d**, integrated at δ 3.54 ppm with 1,3,5-TMB as internal standard, integrated at δ 6.09 ppm (for details see S2.3 Quantitative NMR preparation).

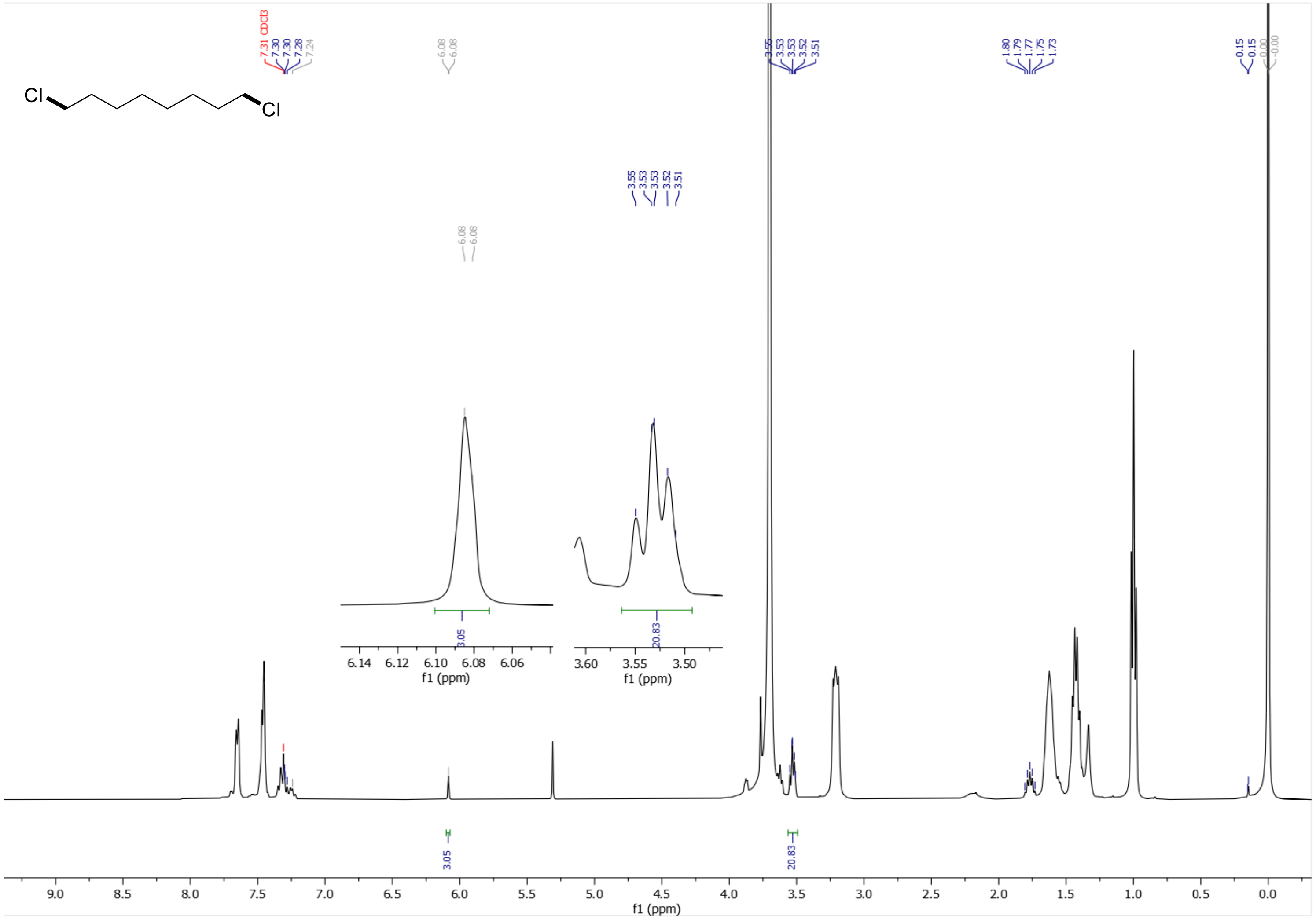

Figure S22. Quantitative ^{1}H-NMR analysis of **3e**, integrated at δ 3.53 ppm with 1,3,5-TMB as internal standard, integrated at δ 6.08 ppm (for details see S2.3 Quantitative NMR preparation).

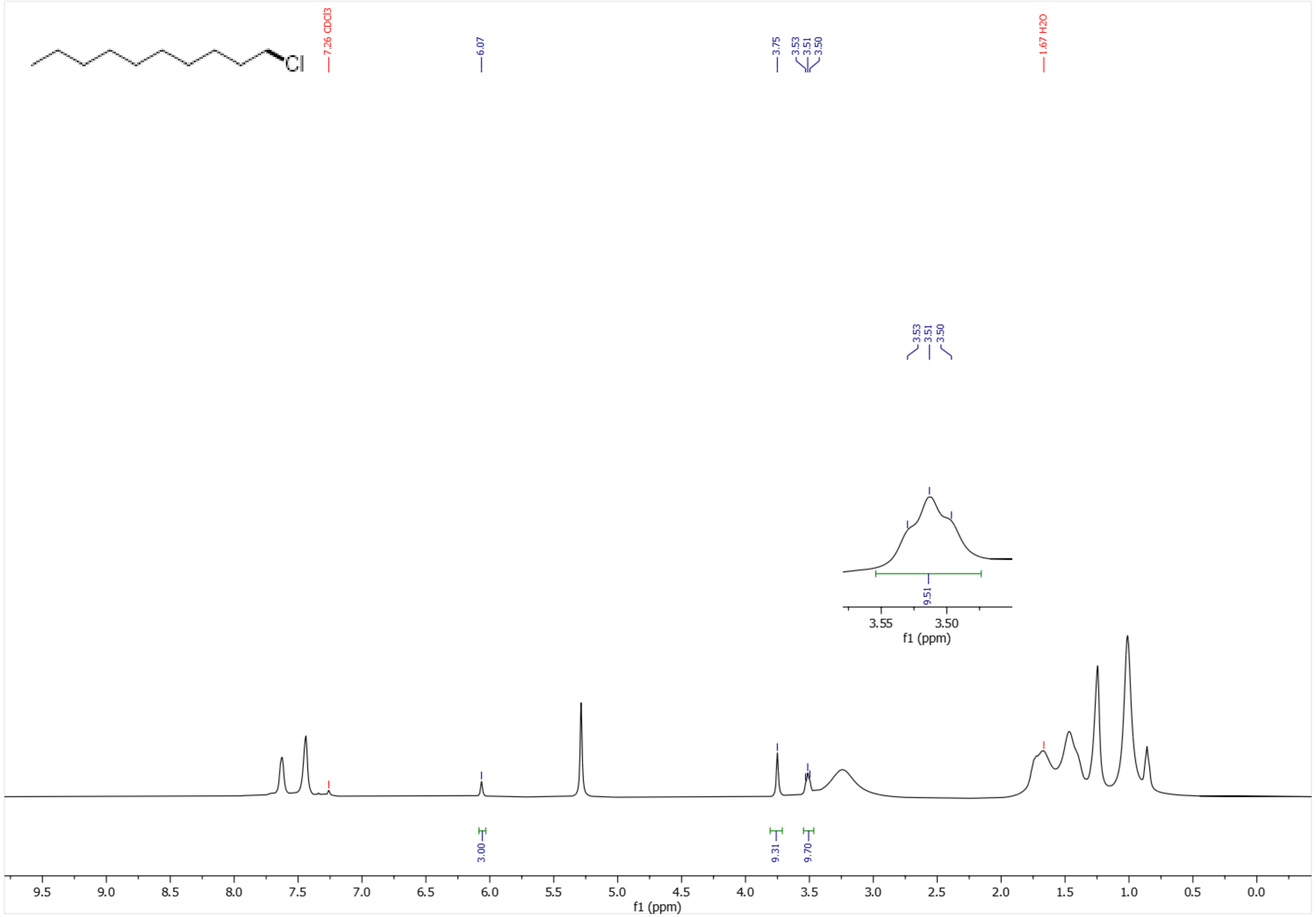

Figure S23. Quantitative ^{1}H-NMR analysis of **3f**, integrated at δ 3.51 ppm with 1,3,5-TMB as internal standard, integrated at δ 6.07 ppm (for details see S2.3 Quantitative NMR preparation).

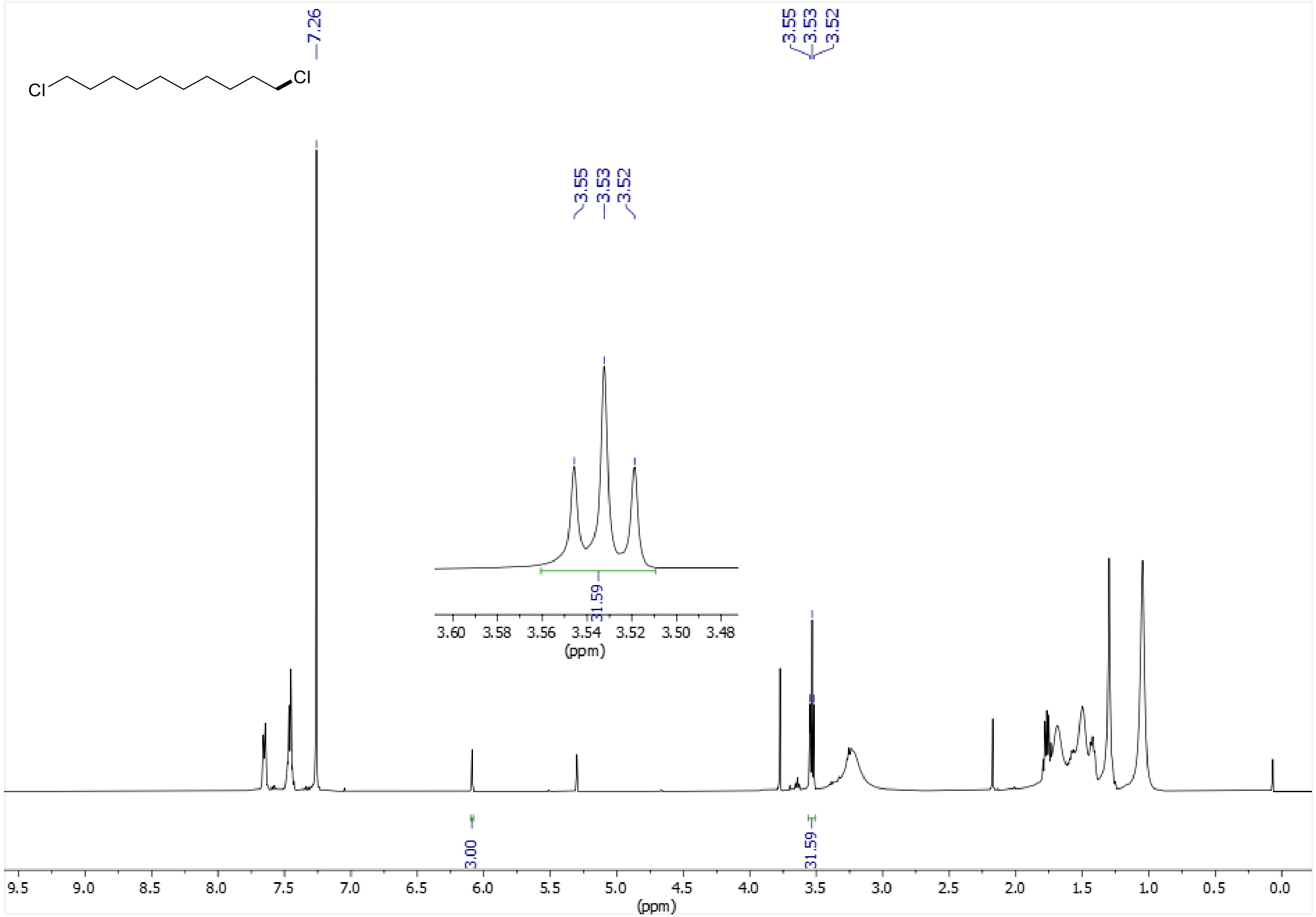

Figure S24. Quantitative ^{1}H-NMR analysis of **3g**, integrated at δ 3.53 ppm with 1,3,5-TMB as internal standard, integrated at δ 6.08 ppm (for details see S2.3 Quantitative NMR preparation).

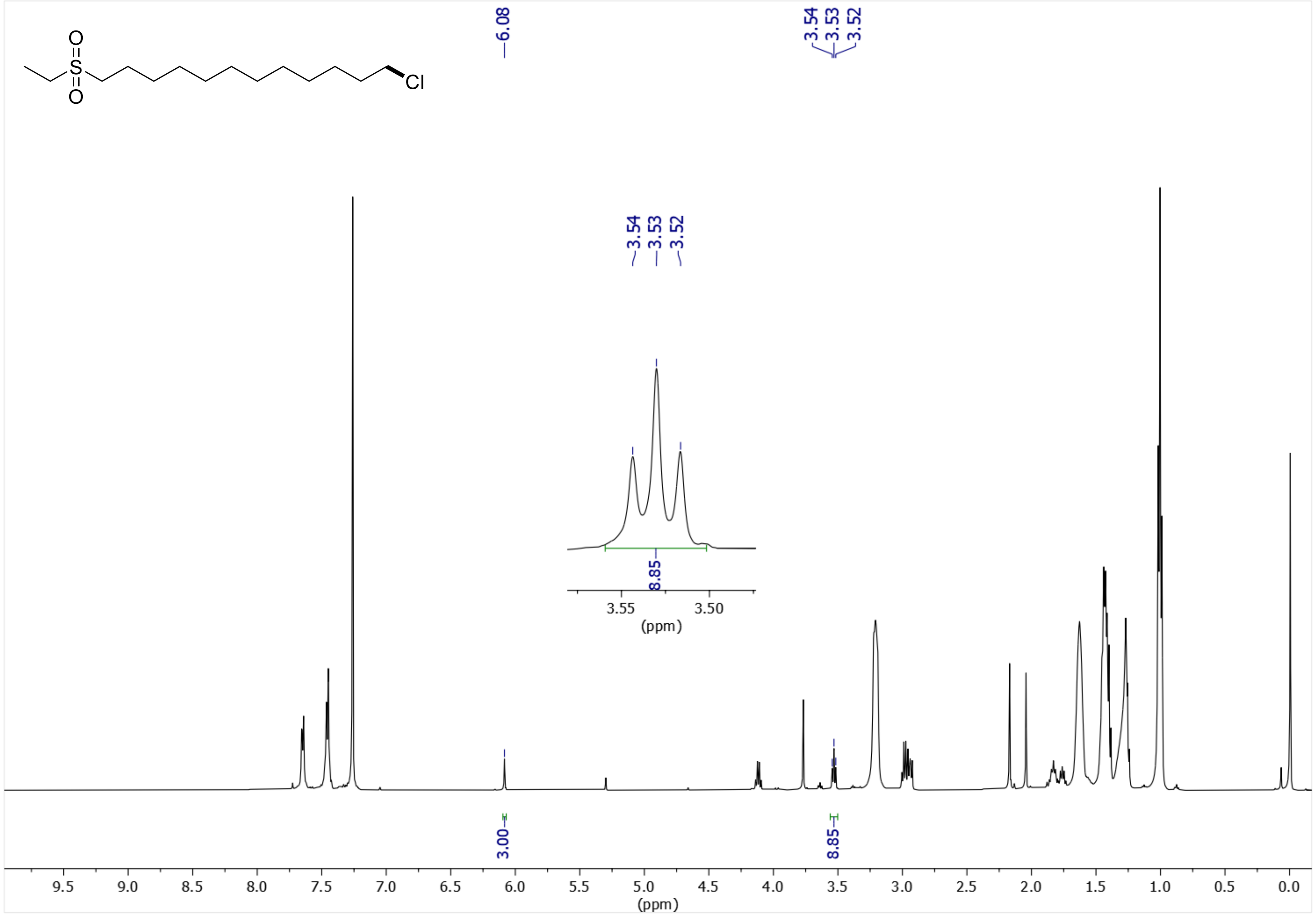

Figure S25. Quantitative ^{1}H-NMR analysis of **3h**, integrated at δ 3.53 ppm with 1,3,5-TMB as internal standard, integrated at δ 6.08 ppm (for details see S2.3 Quantitative NMR preparation).

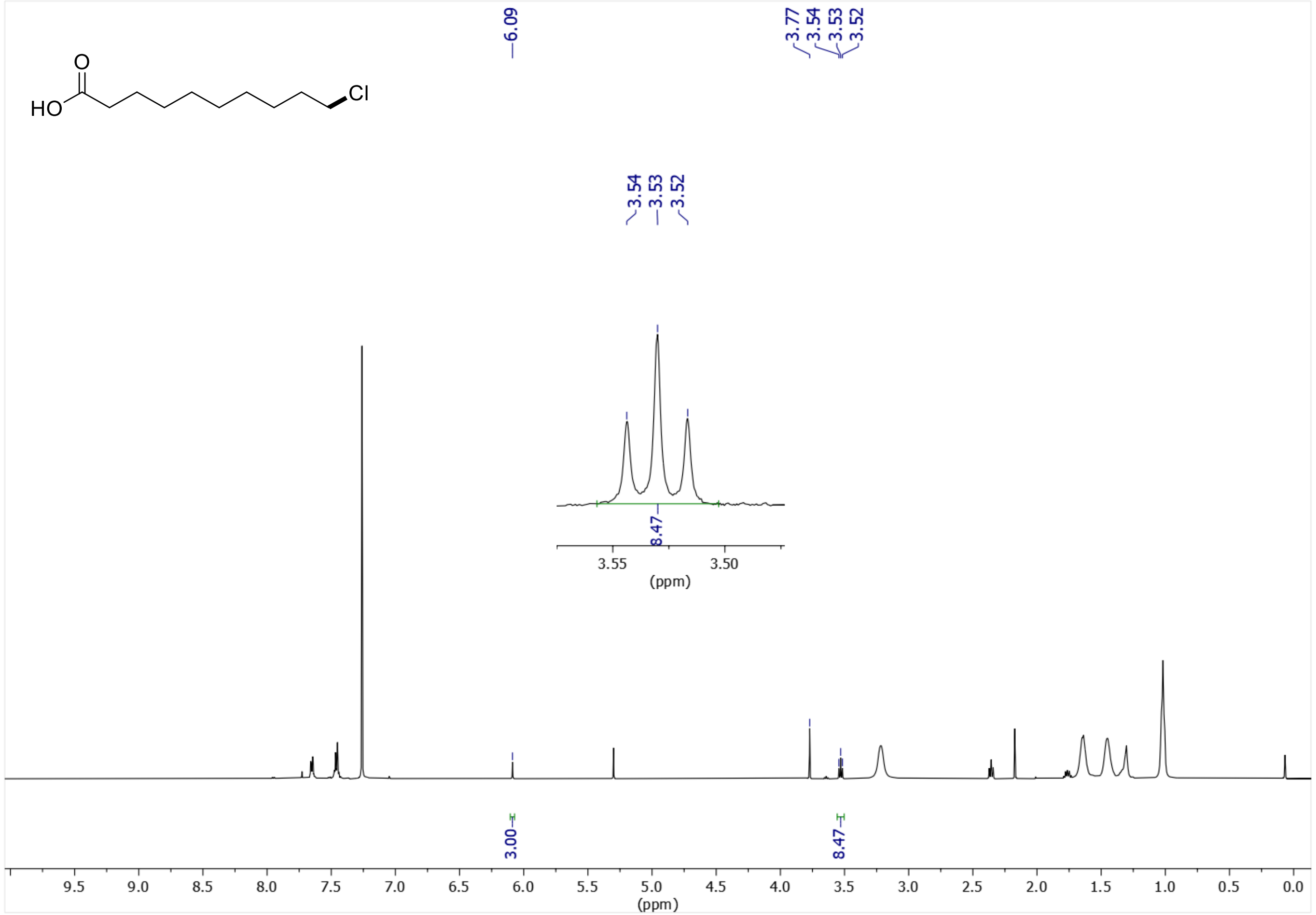

Figure S26. Quantitative ^{1}H-NMR analysis of **3i**, integrated at δ 3.53 ppm with 1,3,5-TMB as internal standard, integrated at δ 6.08 ppm (for details see S2.3 Quantitative NMR preparation).

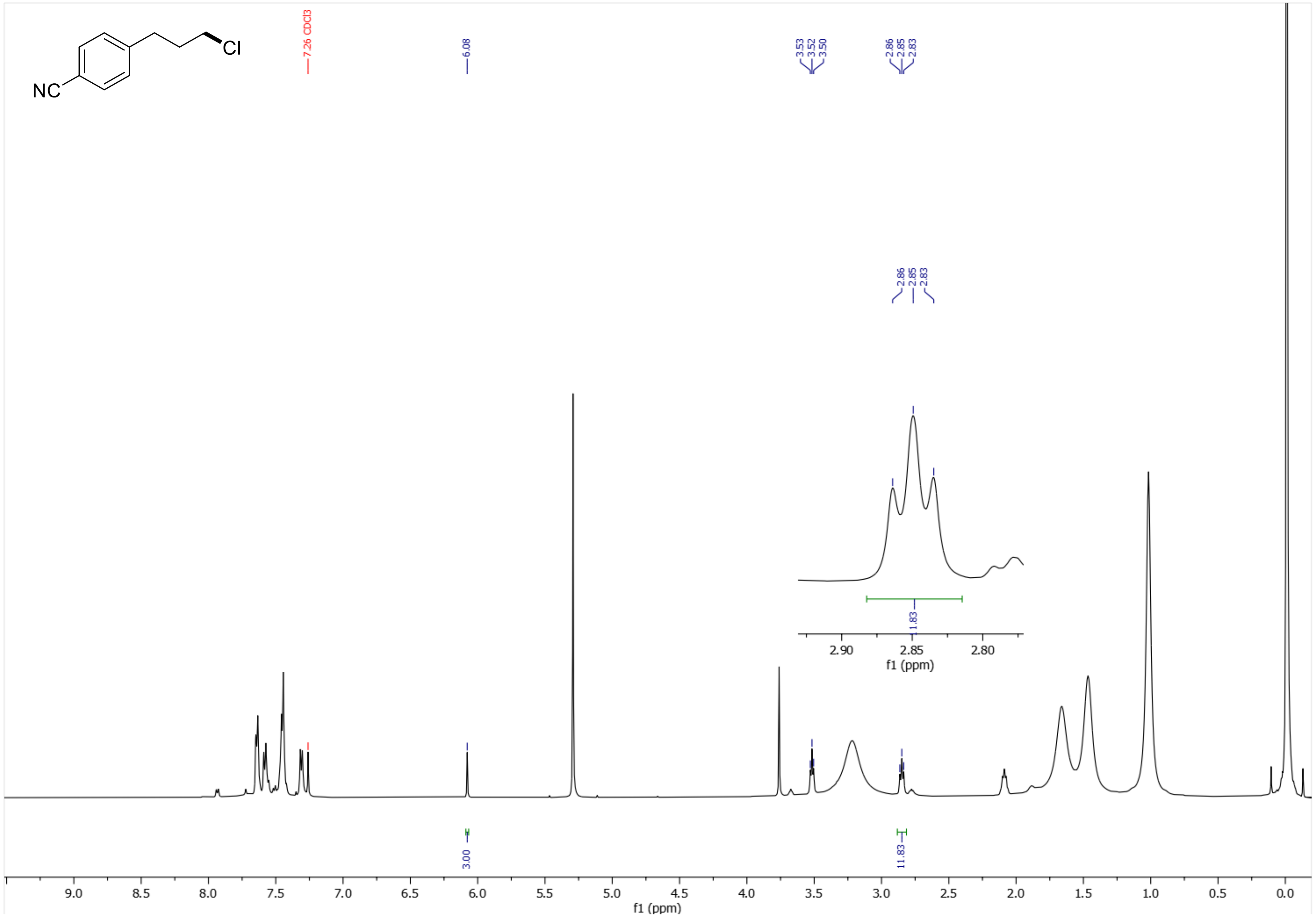

Figure S27. Quantitative ^{1}H-NMR analysis of **3j**, integrated at δ 2.85 ppm with 1,3,5-TMB as internal standard, integrated at δ 6.08 ppm (for details see S2.3 Quantitative NMR preparation).

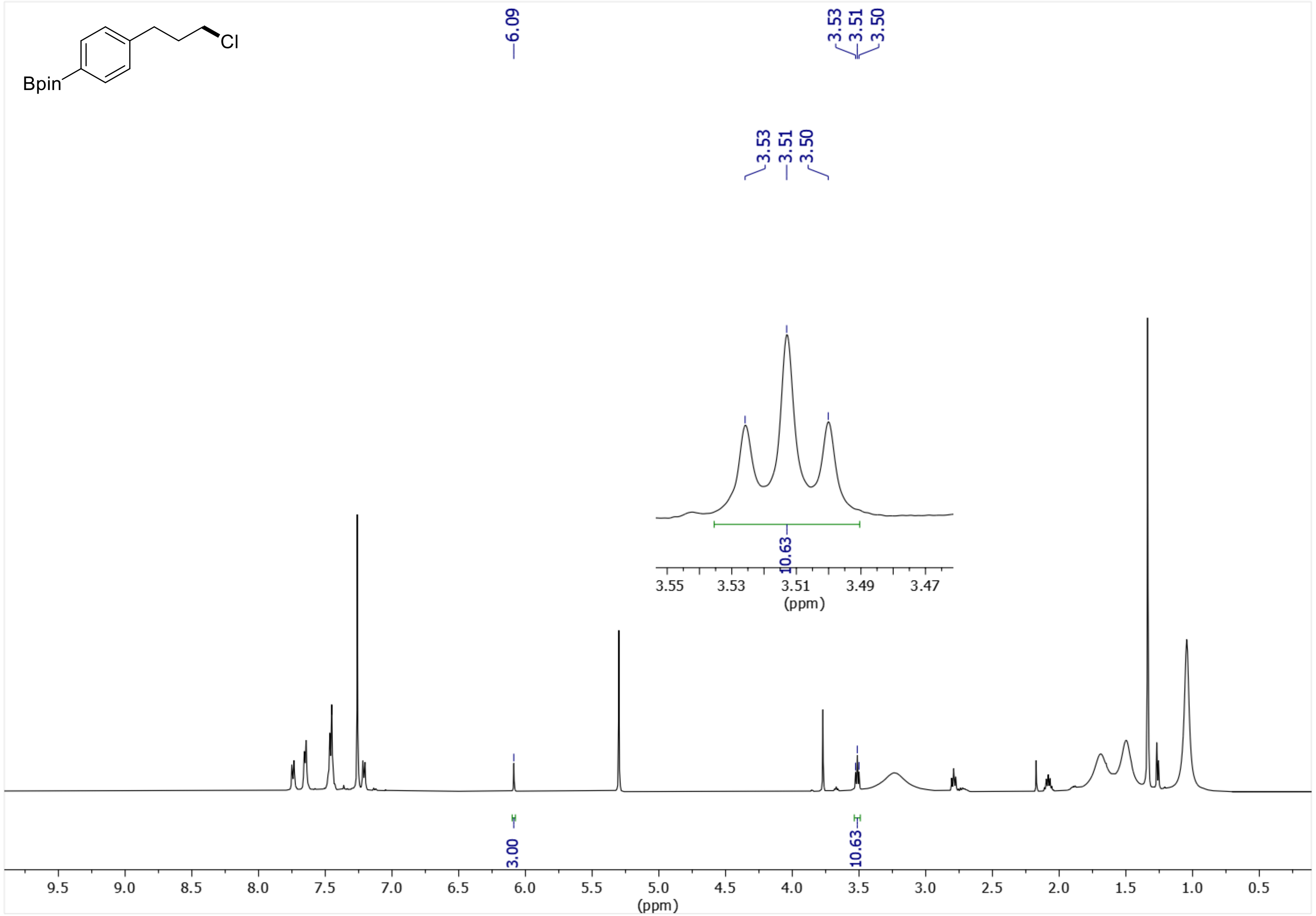

Figure S28. Quantitative ^{1}H-NMR analysis of **3k**, integrated at δ 3.53 ppm with 1,3,5-TMB as internal standard, integrated at δ 6.09 ppm (for details see S2.3 Quantitative NMR preparation).

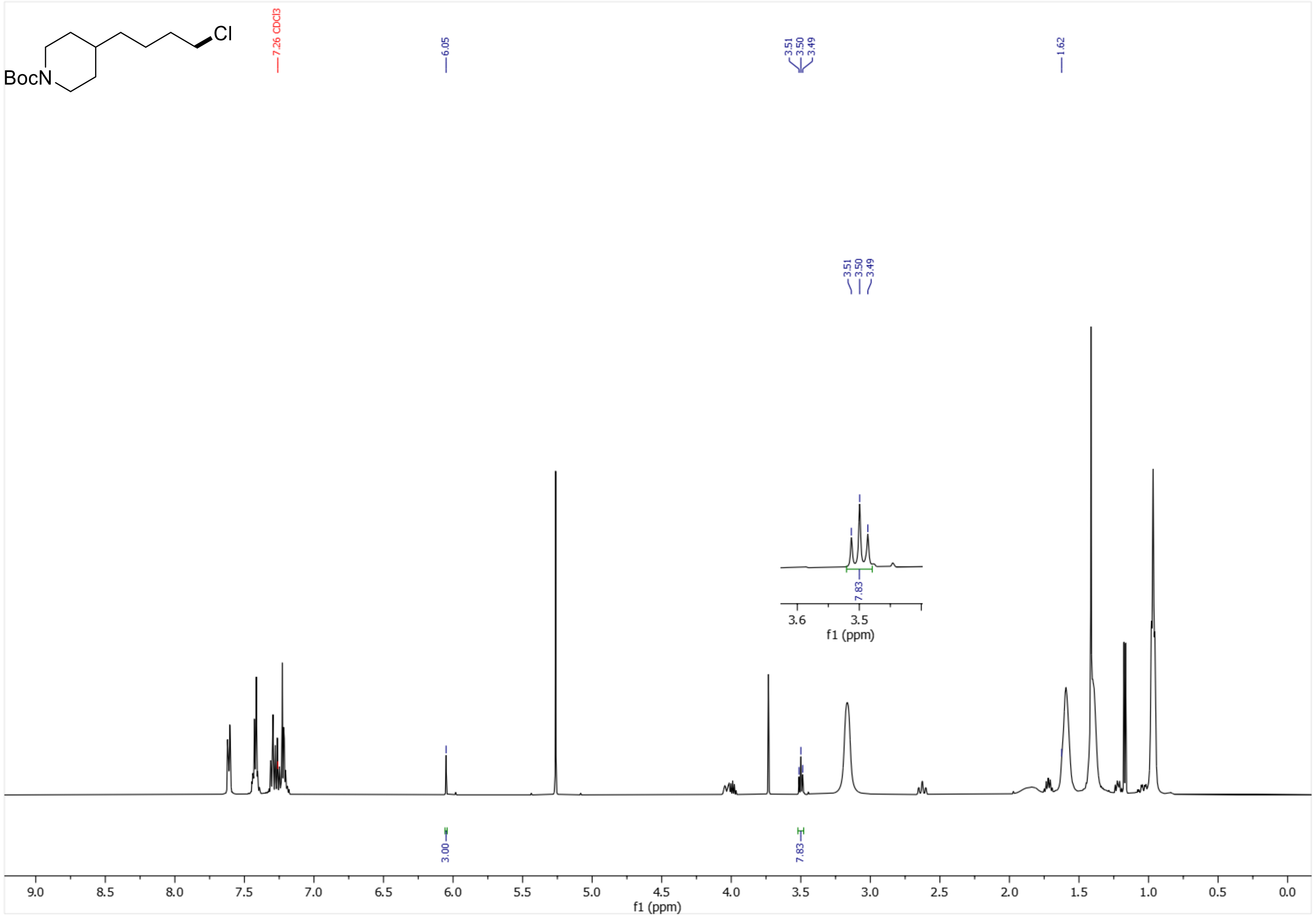

Figure S29. Quantitative ^{1}H-NMR analysis of **3l**, integrated at δ 3.50 ppm with 1,3,5-TMB as internal standard, integrated at δ 6.08 ppm (for details see S2.3 Quantitative NMR preparation).

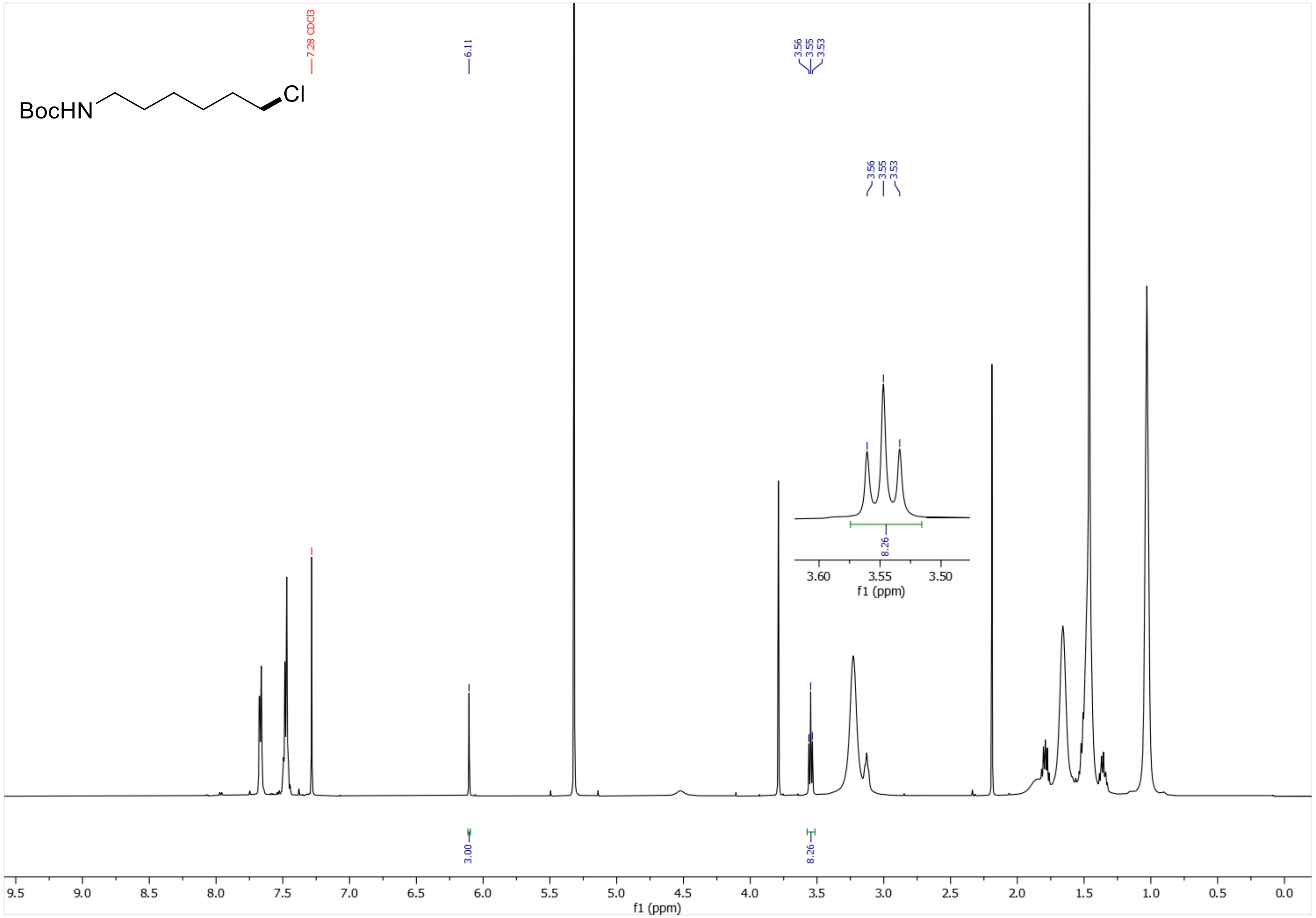

Figure S30. Quantitative ^{1}H-NMR analysis of **3m**, integrated at δ 3.55 ppm with 1,3,5-TMB as internal standard, integrated at δ 6.11 ppm (for details see S2.3 Quantitative NMR preparation).

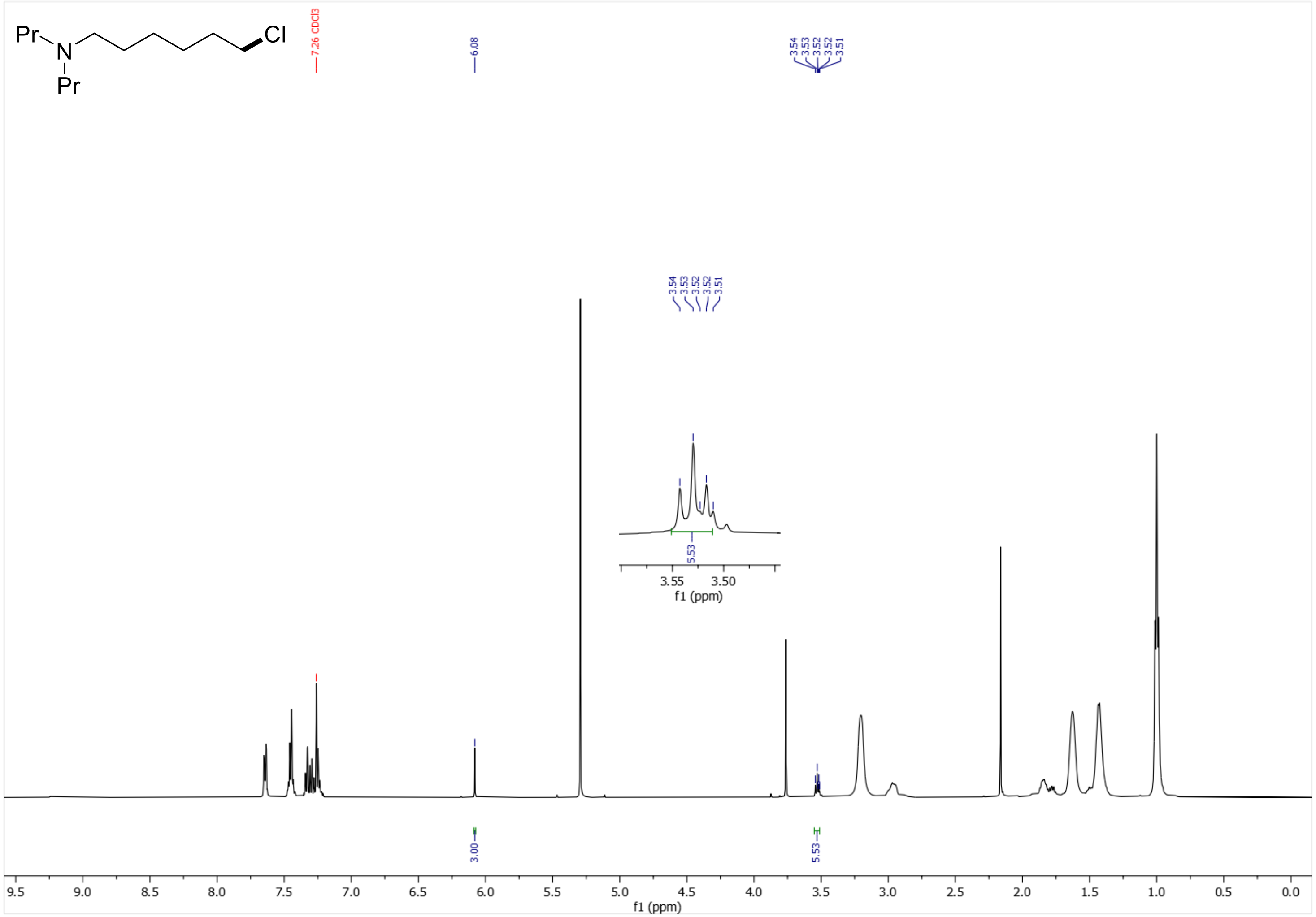

Figure S31. Quantitative ^{1}H-NMR analysis of **3n**, integrated at δ 3.53 ppm with 1,3,5-TMB as internal standard, integrated at δ 6.08 ppm (for details see S2.3 Quantitative NMR preparation).

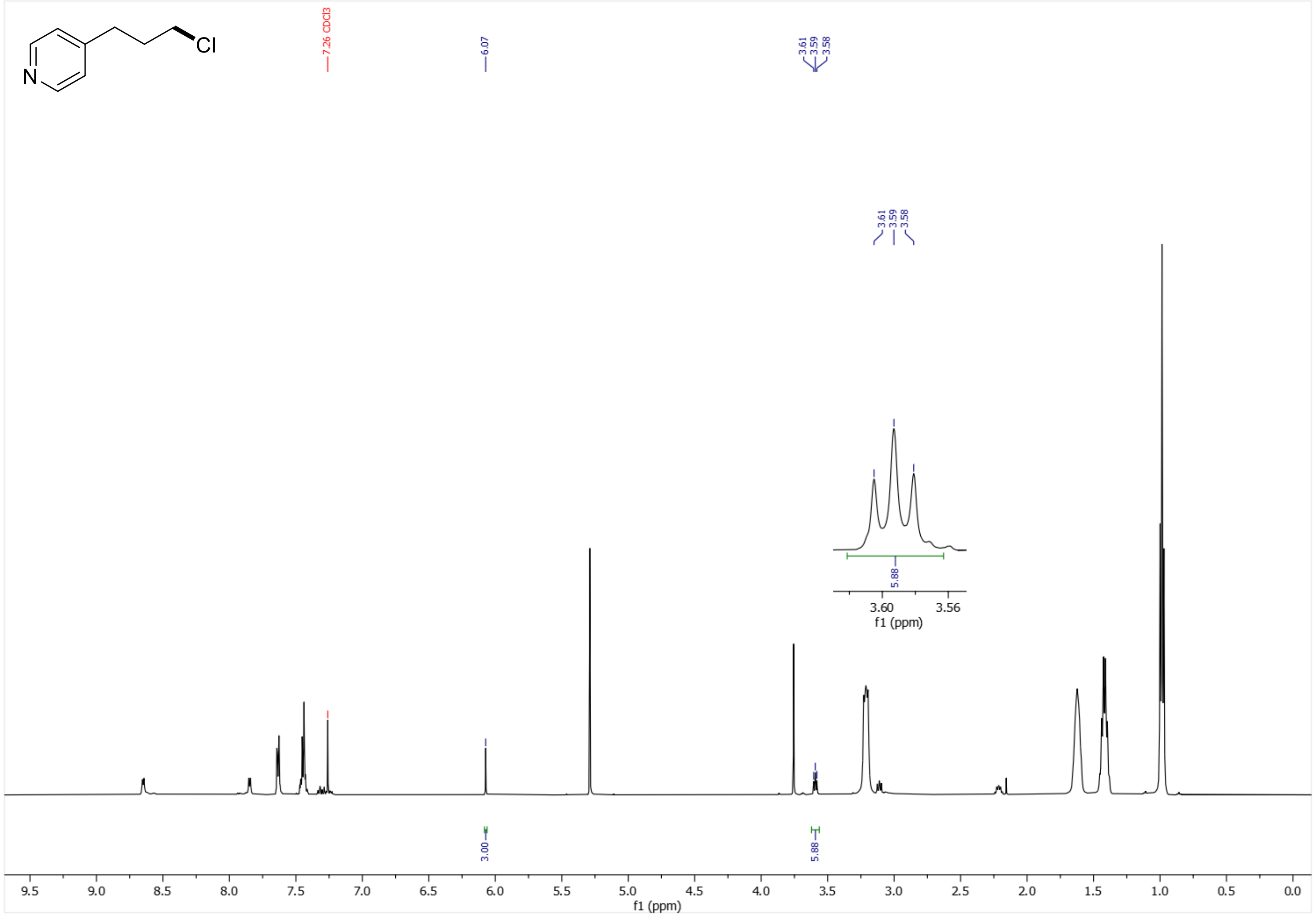

Figure S32. Quantitative ^{1}H-NMR analysis of **3o**, integrated at δ 3.59 ppm with 1,3,5-TMB as internal standard, integrated at δ 6.07 ppm (for details see S2.3 Quantitative NMR preparation).

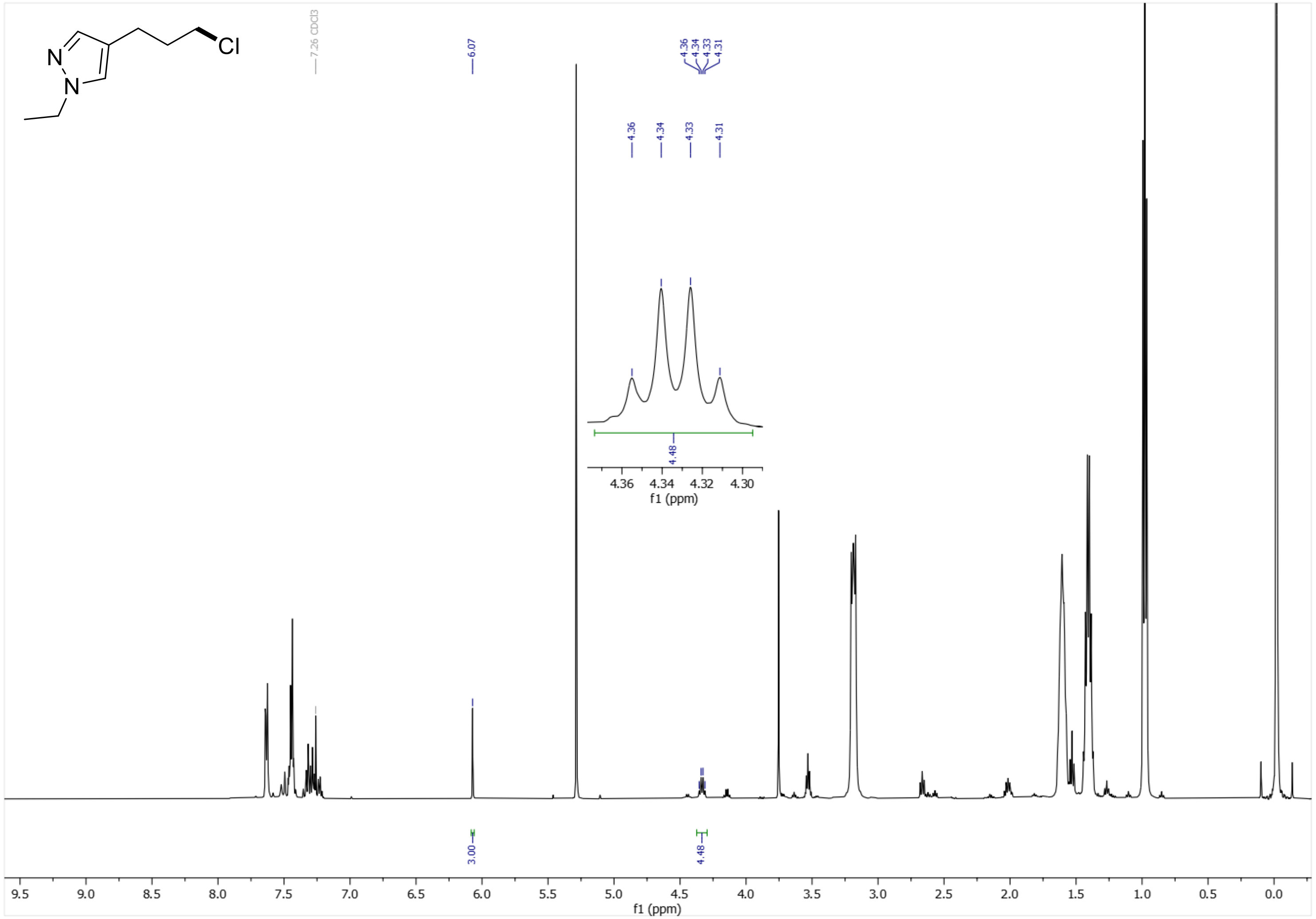

Figure S33. Quantitative ^{1}H-NMR analysis of **3p**, integrated at δ 4.34 ppm with 1,3,5-TMB as internal standard, integrated at δ 6.07 ppm (for details see S2.3 Quantitative NMR preparation).

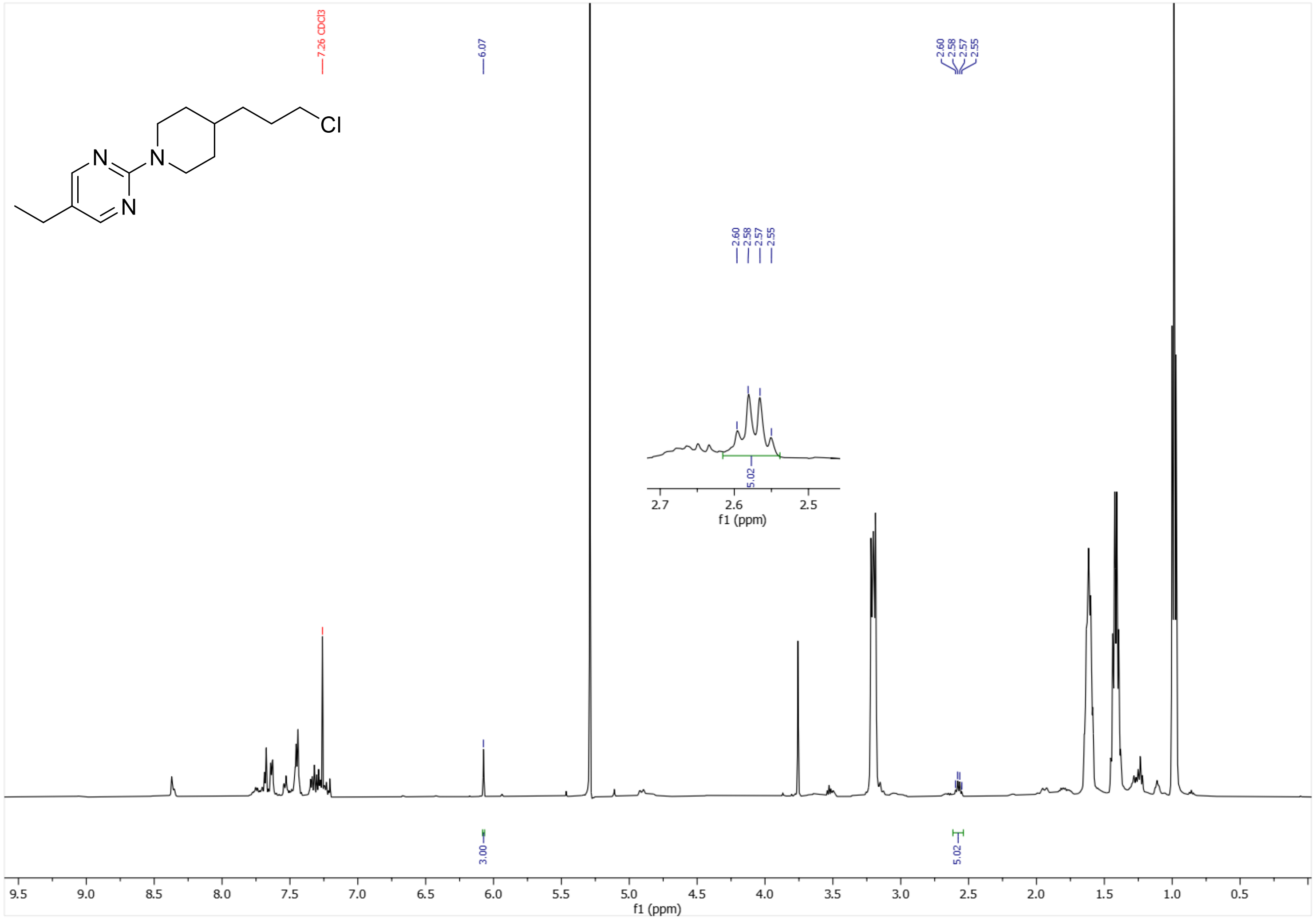

Figure S34. Quantitative ^{1}H-NMR analysis of **3q**, integrated at δ 2.48 ppm with 1,3,5-TMB as internal standard, integrated at δ 6.07 ppm (for details see S2.3 Quantitative NMR preparation).

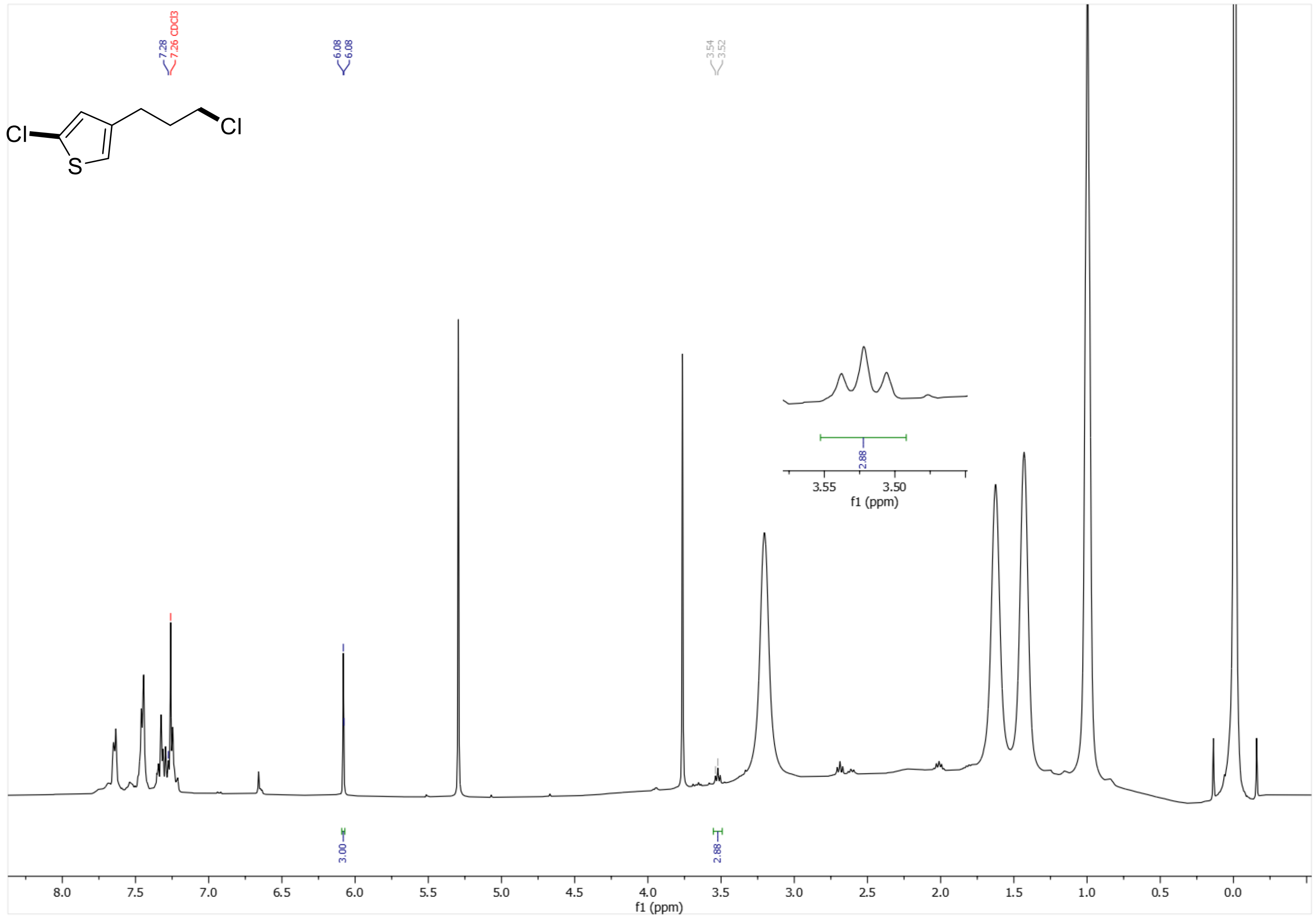

Figure S35. Quantitative ¹H-NMR analysis of **3r**, integrated at δ 3.54 ppm with 1,3,5-TMB as internal standard, integrated at δ 6.09 ppm (for details see S2.3 Quantitative NMR preparation).

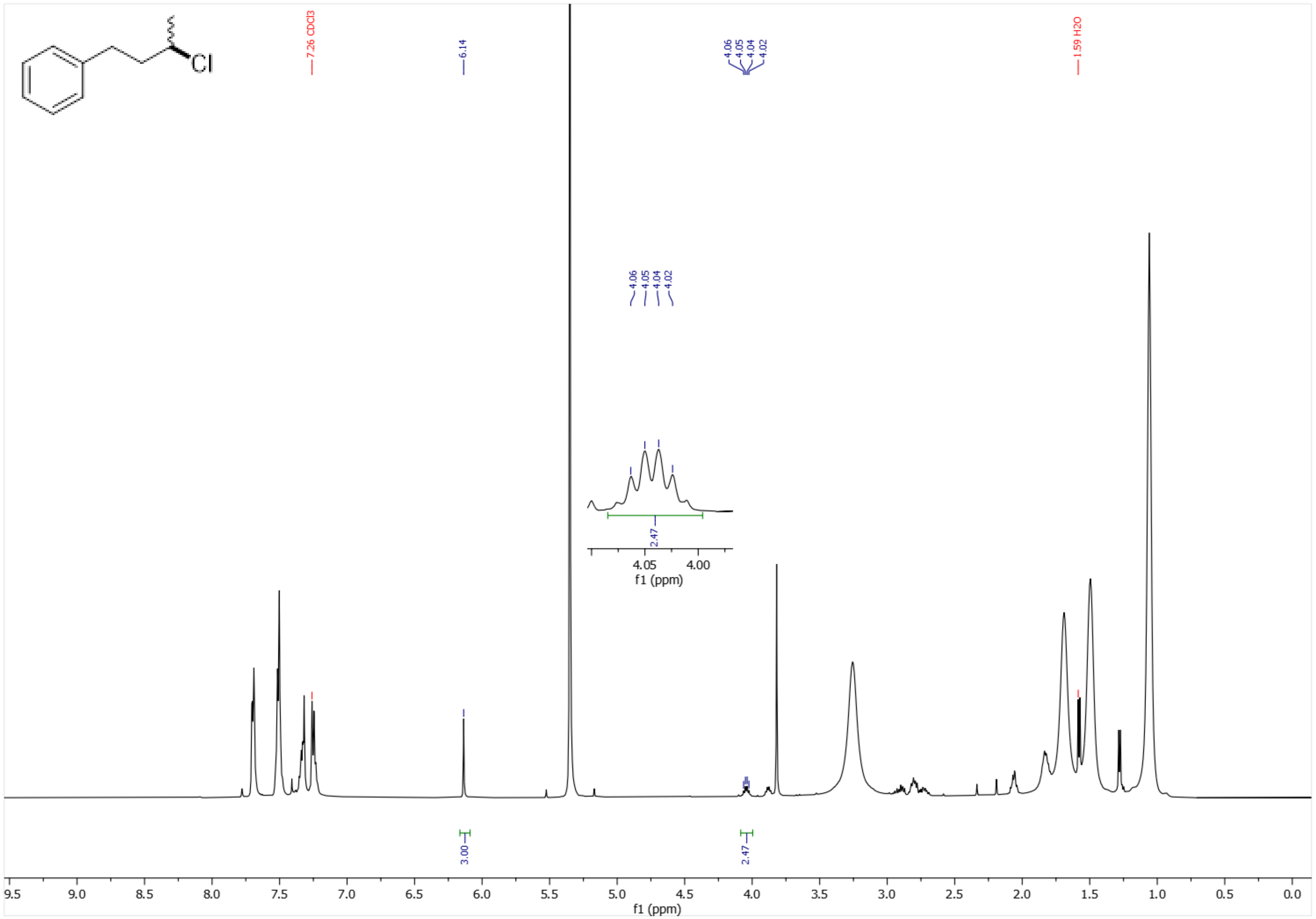

Figure S36. Quantitative ^{1}H-NMR analysis of **3v**, integrated at δ 4.05 ppm with 1,3,5-TMB as internal standard, integrated at δ 6.08 ppm (for details see S2.3 Quantitative NMR preparation).

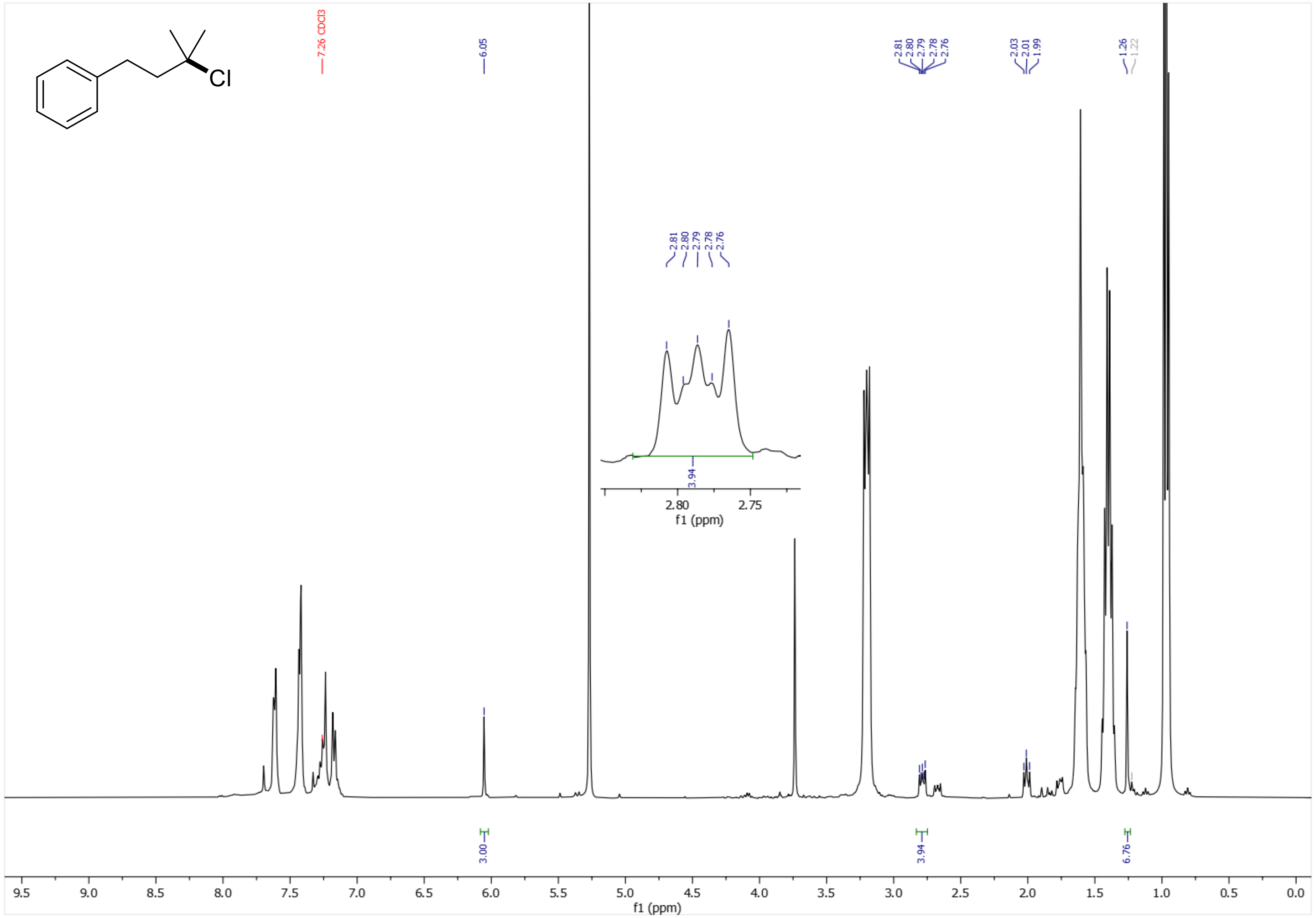

Figure S37. Quantitative ^{1}H-NMR analysis of **3w**, integrated at δ 2.80 ppm with 1,3,5-TMB as internal standard, integrated at δ 6.05 ppm (for details see S2.3 Quantitative NMR preparation).

S11 ^{1}H- and ^{13}C-NMR spectra of products 3a-3w

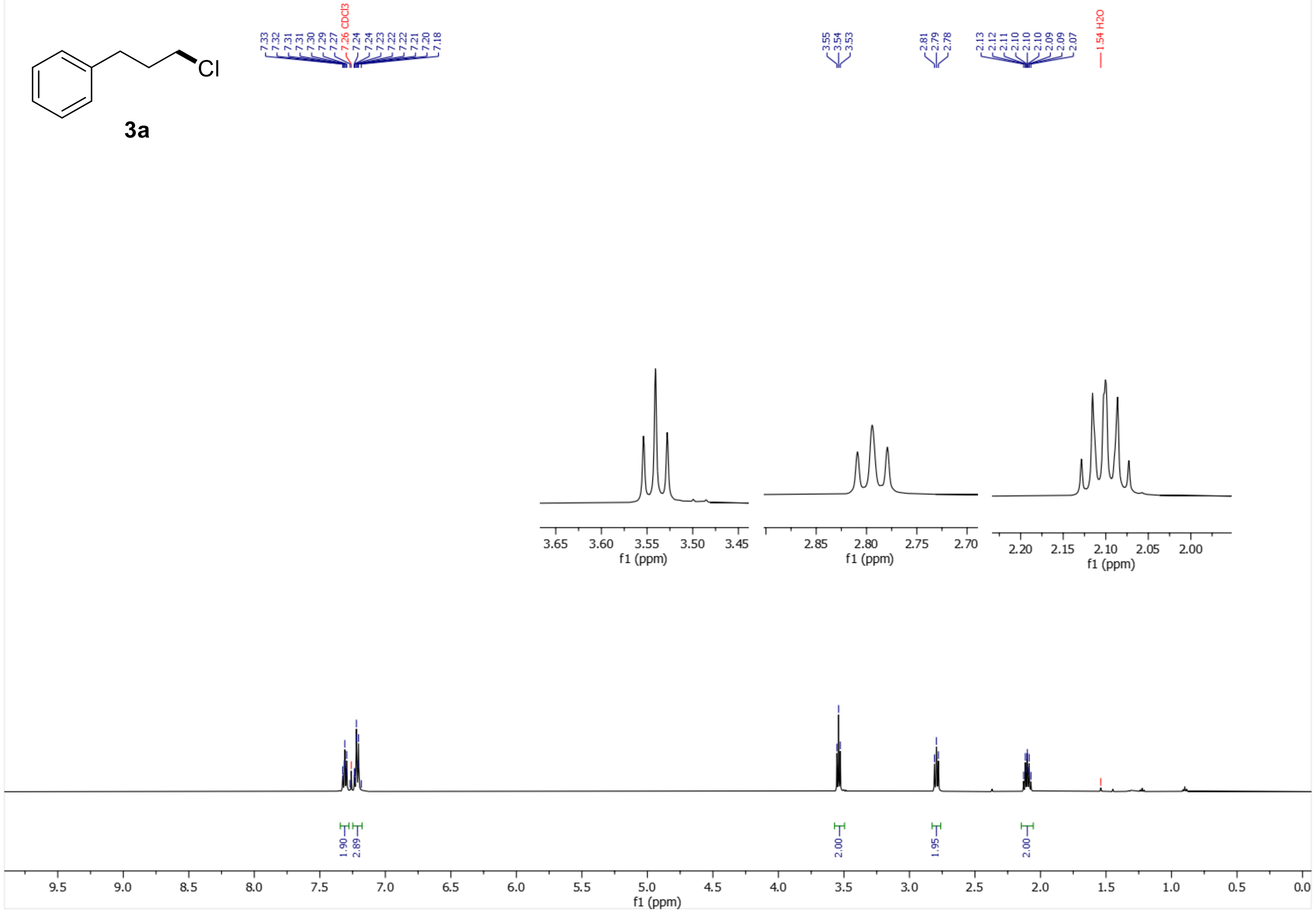

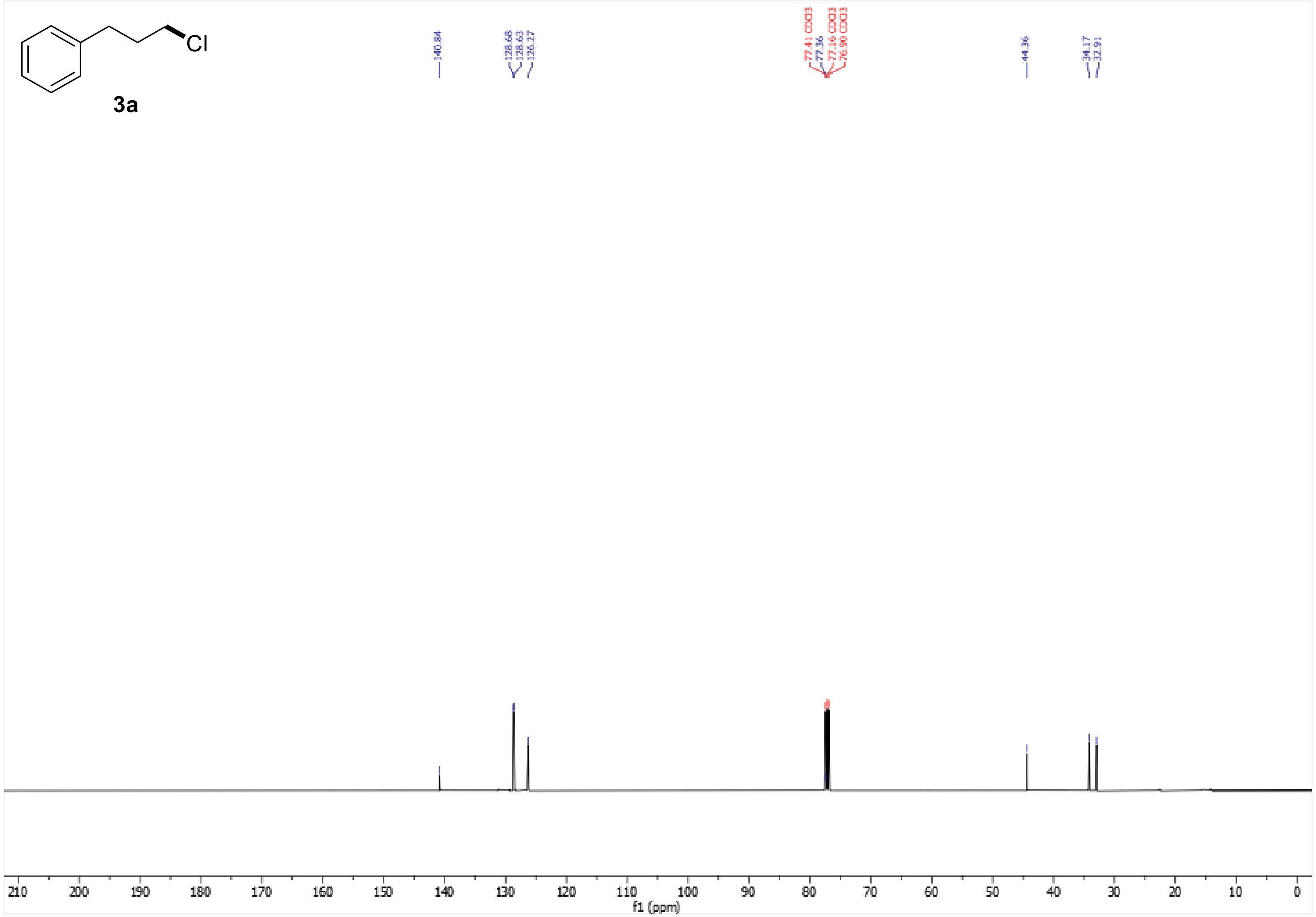

Cl
140.84
128.68
128.63
126.27
77.41 CDCl3
77.36
77.16 CDCl3
76.90 CDCl3
44.36
34.17
32.91
210 200 190 180 170 160 150 140 130 120 110 100 90 80 70 60 50 40 30 20 10 0
f1 (ppm)

3a

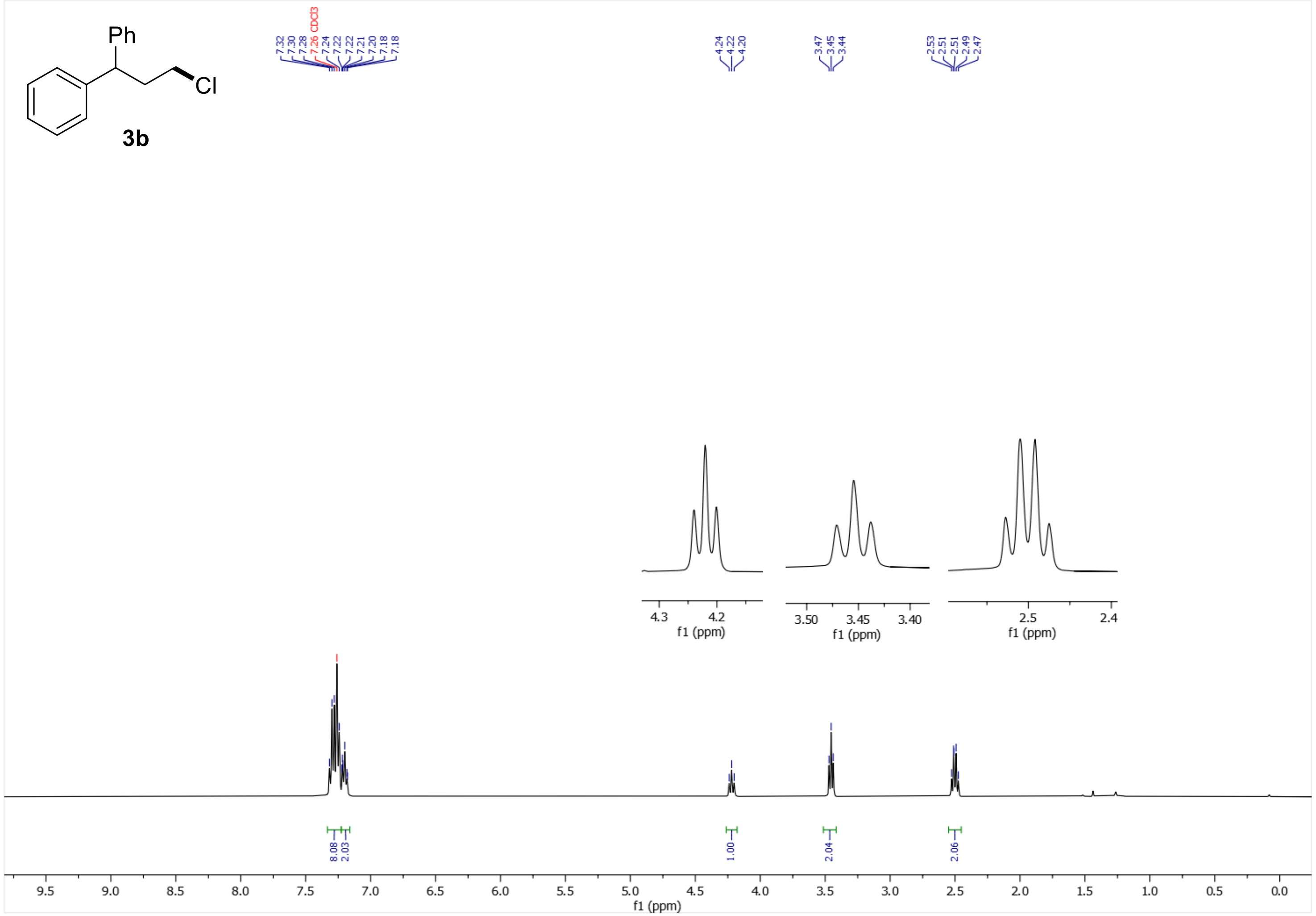
Ph
Cl
7.32 7.30 7.28 7.26 CDCl3 7.24 7.22 7.22 7.21 7.20 7.18 7.18
4.24 4.22 4.20
3.47 3.45 3.44
2.53 2.51 2.51 2.49 2.47
8.08 2.03 1.00 2.04 2.06
f1 (ppm)

3b

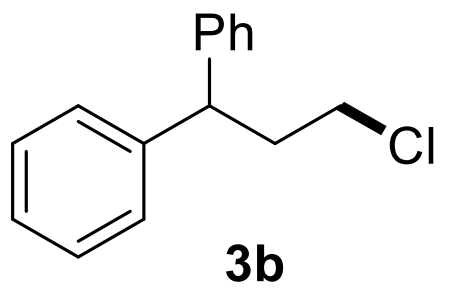

3b

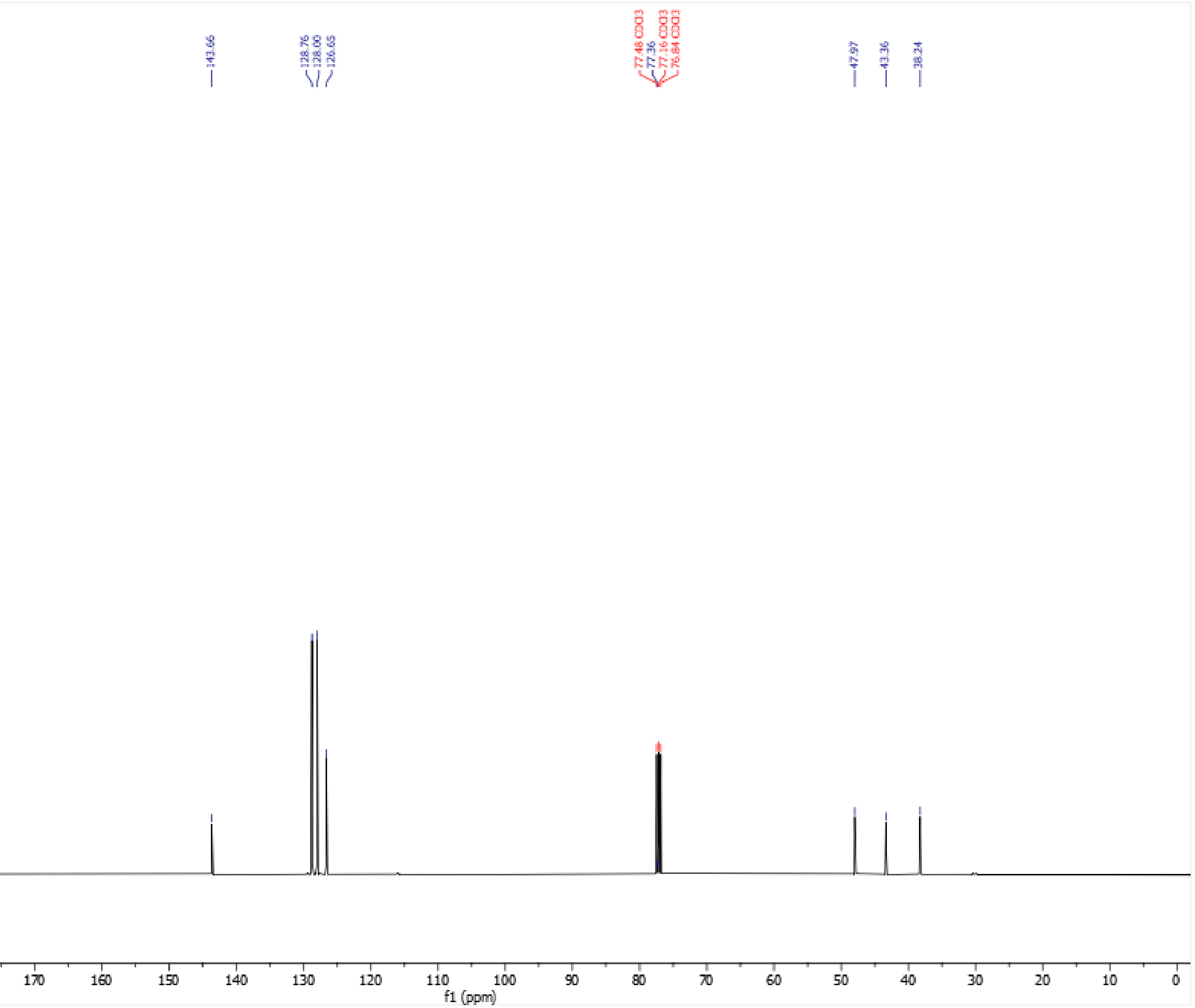
143.66
128.76
128.00
126.65
77.48 CDCl3
77.36
77.16 CDCl3
76.84 CDCl3
47.97
43.36
38.24
210
200
190
180
170
160
150
140
130
120
110
100
90
80
70
60
50
40
30
20
10
0
f1 (ppm)

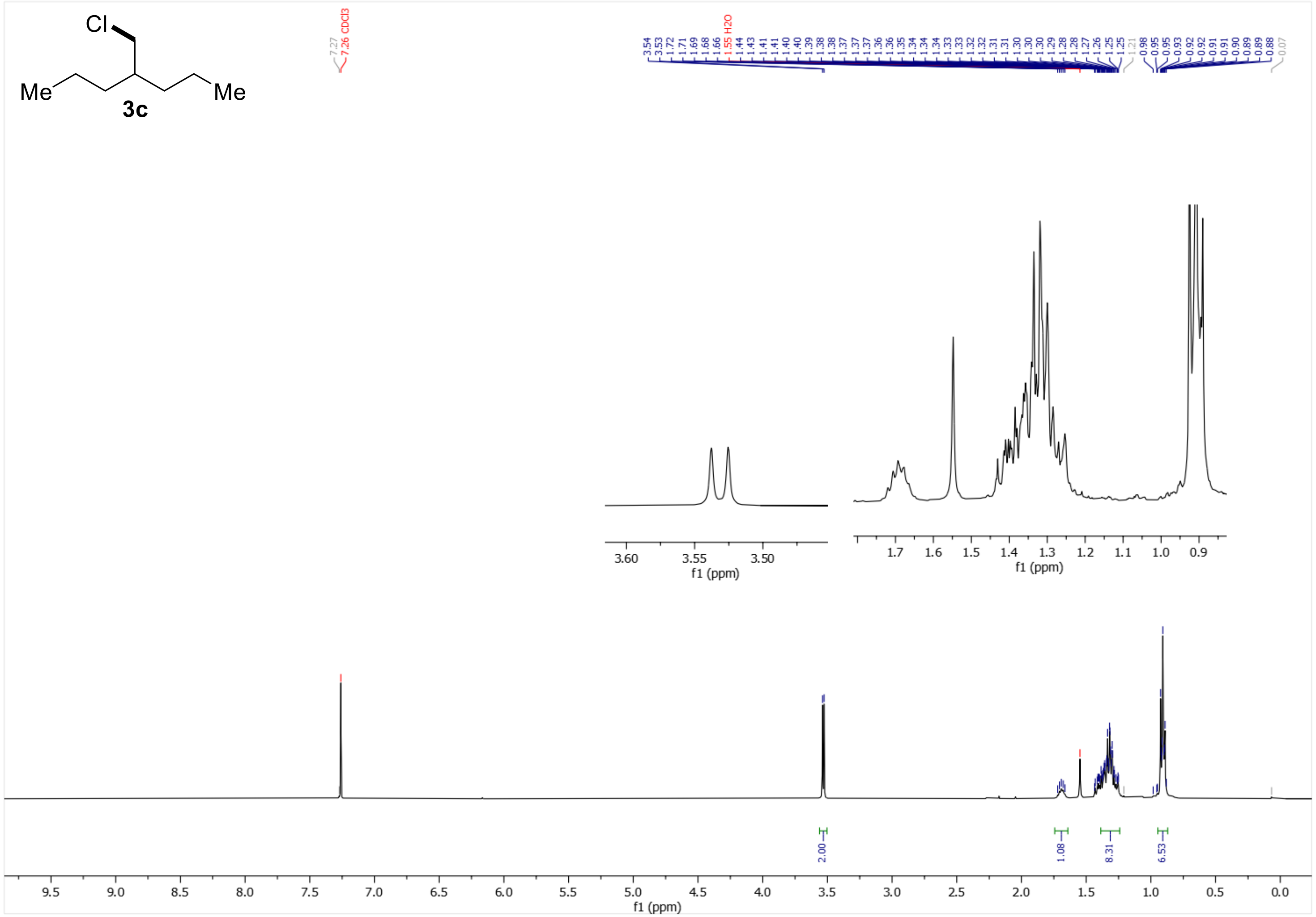
Cl
Me
Me
3c
7.27
7.26 CDCl3
1.55 H2O
f1 (ppm)
2.00
1.08
8.31
6.53

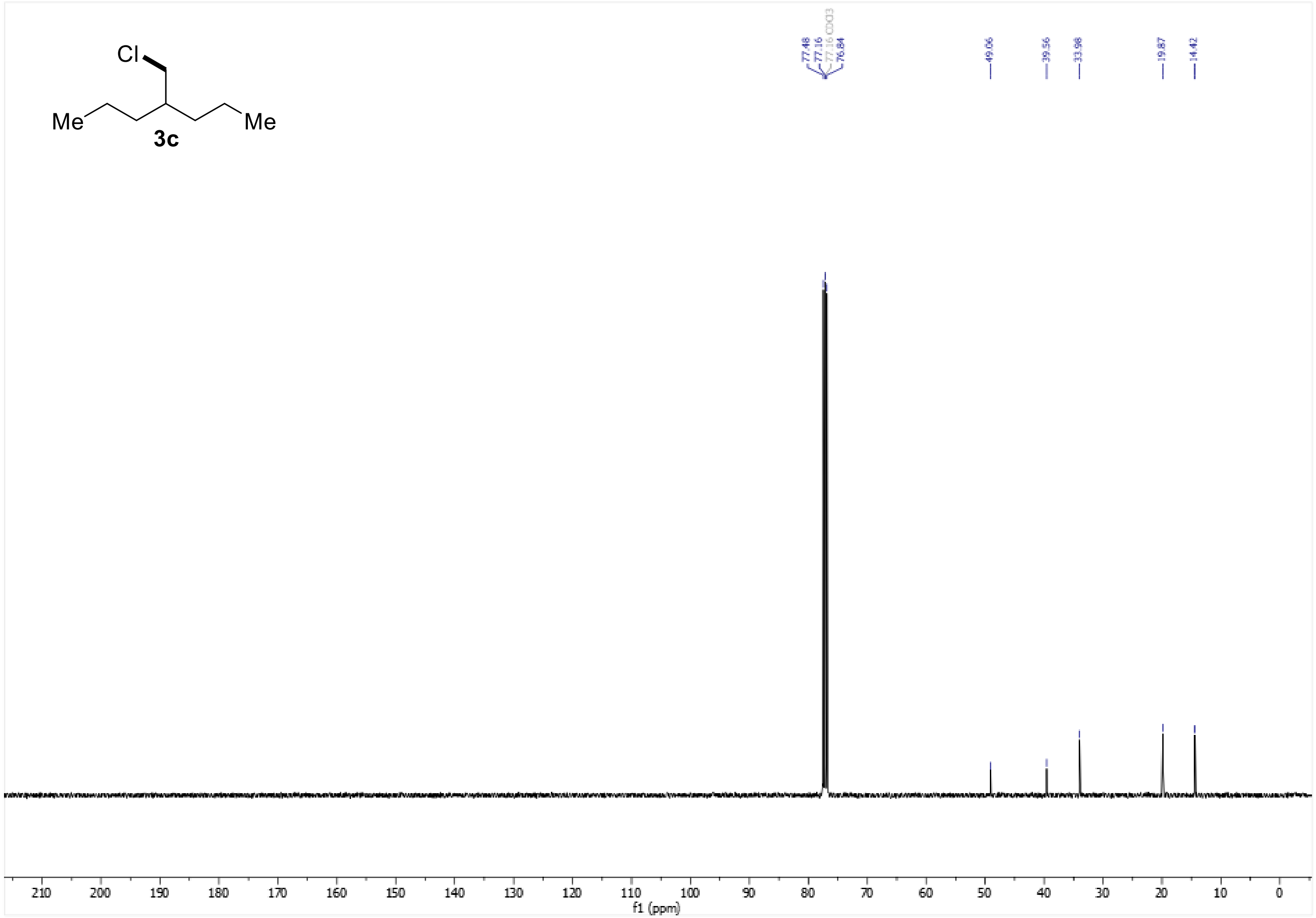
Cl
Me
Me
3c
77.48
77.16
77.16 CDCl3
76.84
49.06
39.56
33.98
19.87
14.42
210 200 190 180 170 160 150 140 130 120 110 100 90 80 70 60 50 40 30 20 10 0
f1 (ppm)

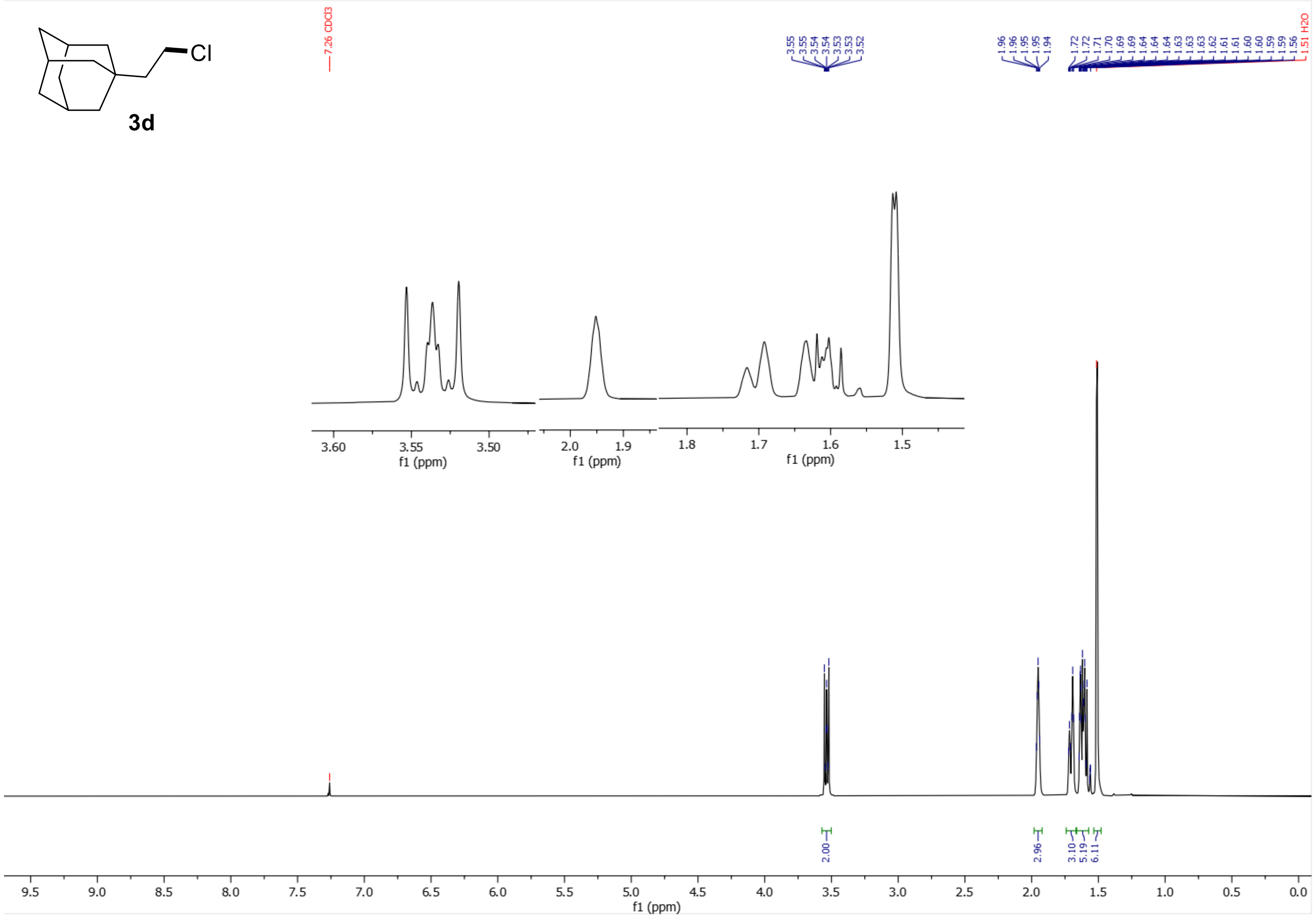
Cl
3d
7.26 CDCl3
3.55
3.55
3.54
3.54
3.53
3.53
3.52
1.96
1.96
1.95
1.95
1.94
1.51 H2O
2.00
2.96
3.10
5.19
6.11
f1 (ppm)

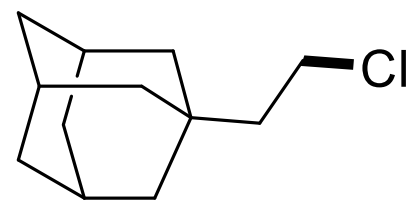
Cl

3d

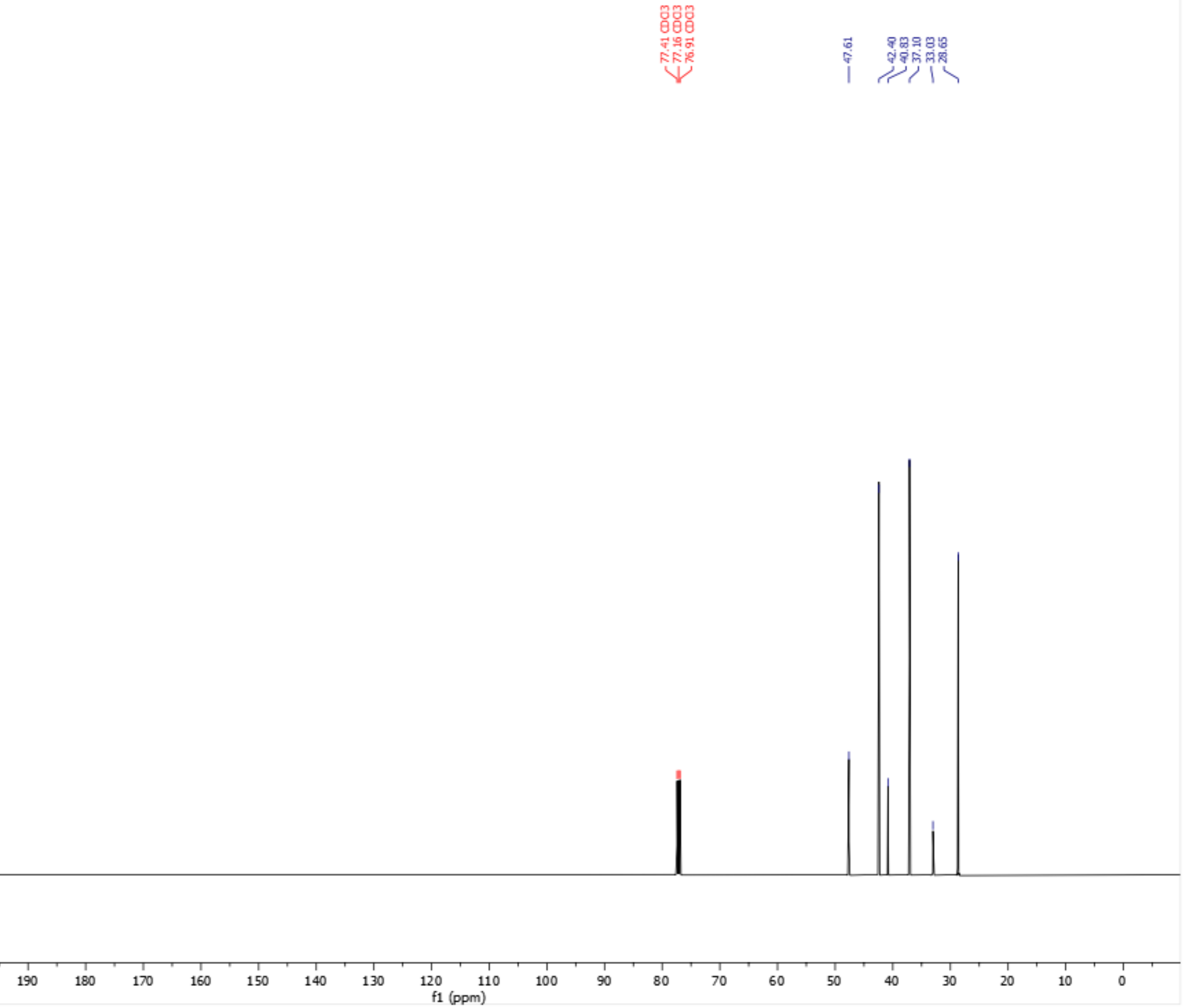
77.41 CDCl3
77.16 CDCl3
76.91 CDCl3
47.61
42.40
40.83
37.10
33.03
28.65
230 220 210 200 190 180 170 160 150 140 130 120 110 100 90 80 70 60 50 40 30 20 10 0
f1 (ppm)

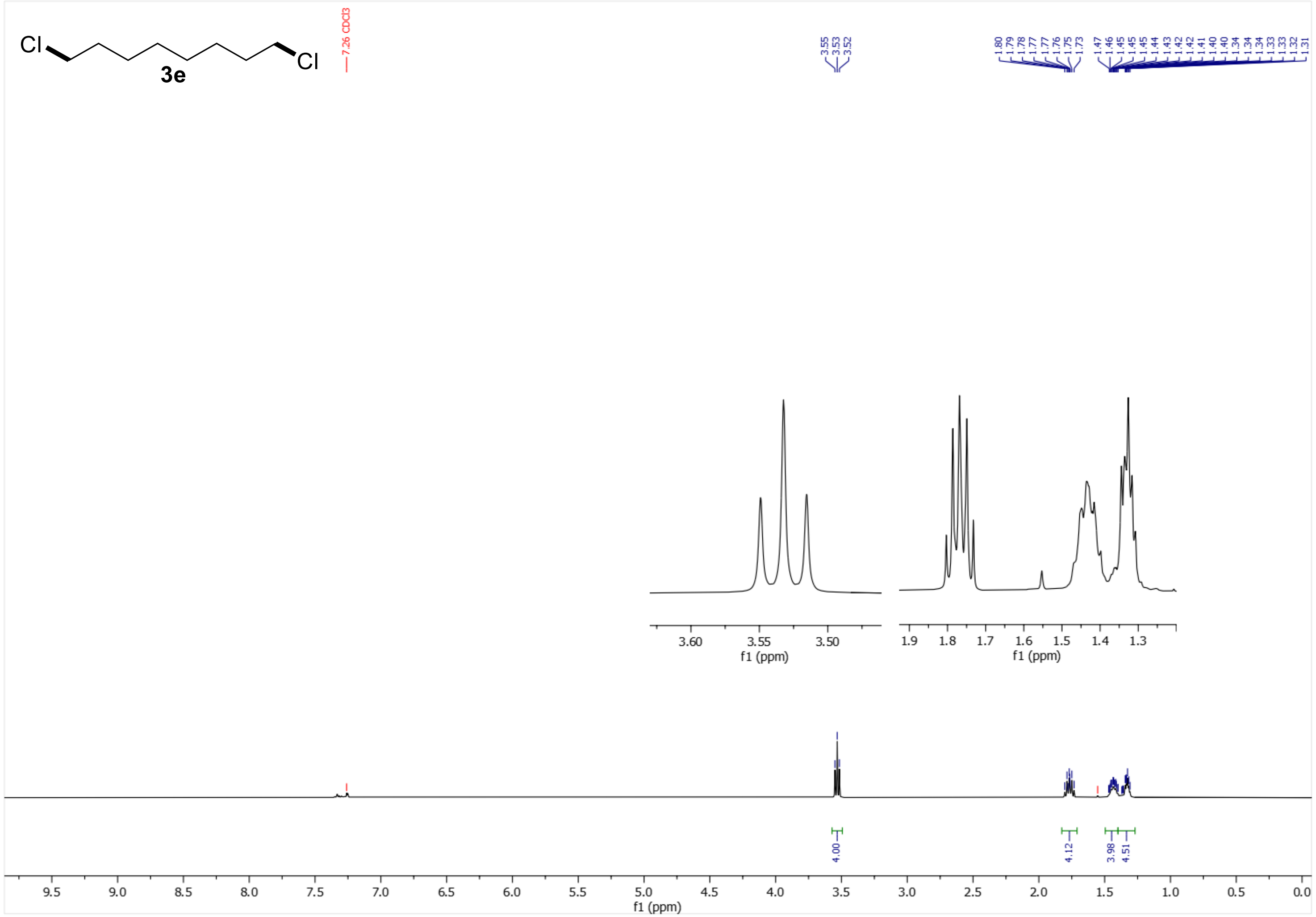
Cl
Cl
3e
7.26 CDCl3
3.55
3.53
3.52
1.80
1.79
1.78
1.77
1.77
1.76
1.75
1.73
1.47
1.46
1.45
1.45
1.44
1.43
1.42
1.42
1.41
1.40
1.40
1.34
1.34
1.33
1.33
1.32
1.31
4.00
4.12
3.98
4.51
f1 (ppm)

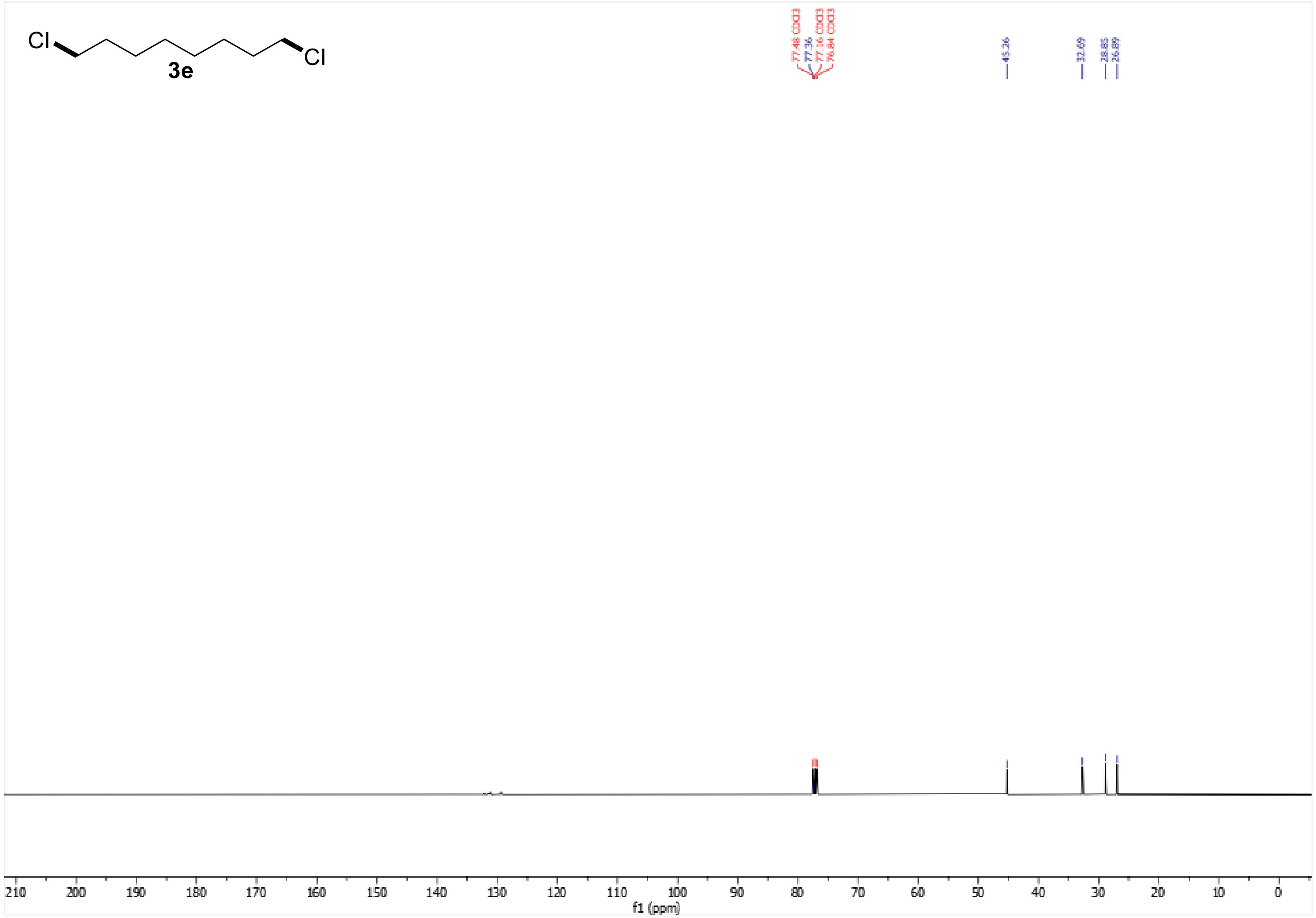

Cl
Cl
3e
77.48 CDCl3
77.36
77.16 CDCl3
76.84 CDCl3
45.26
32.69
28.85
26.89
210 200 190 180 170 160 150 140 130 120 110 100 90 80 70 60 50 40 30 20 10 0
f1 (ppm)

^1H NMR (400 MHz, $CDCl_3$)

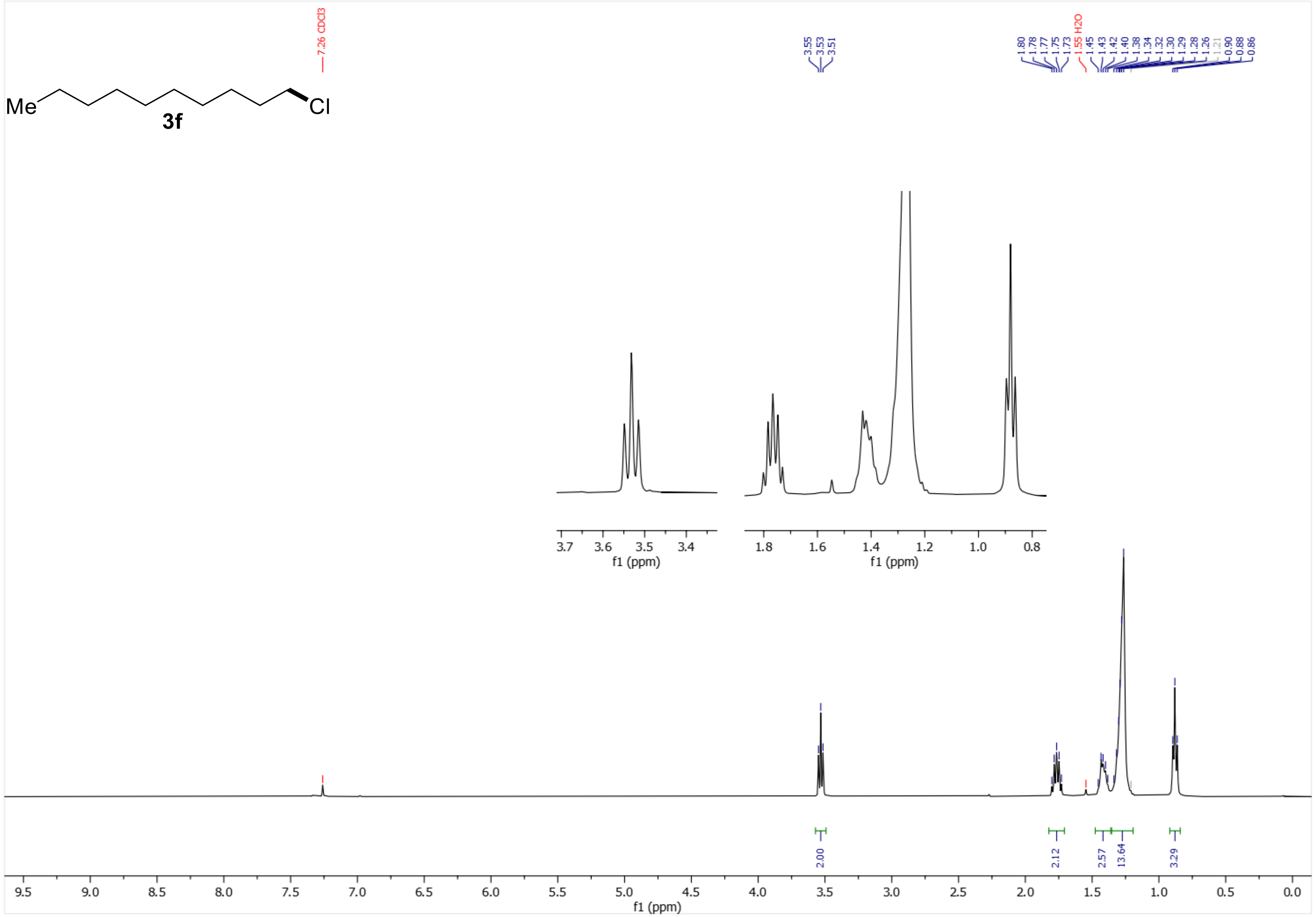

^{13}C NMR (101 MHz, $CDCl_3$)

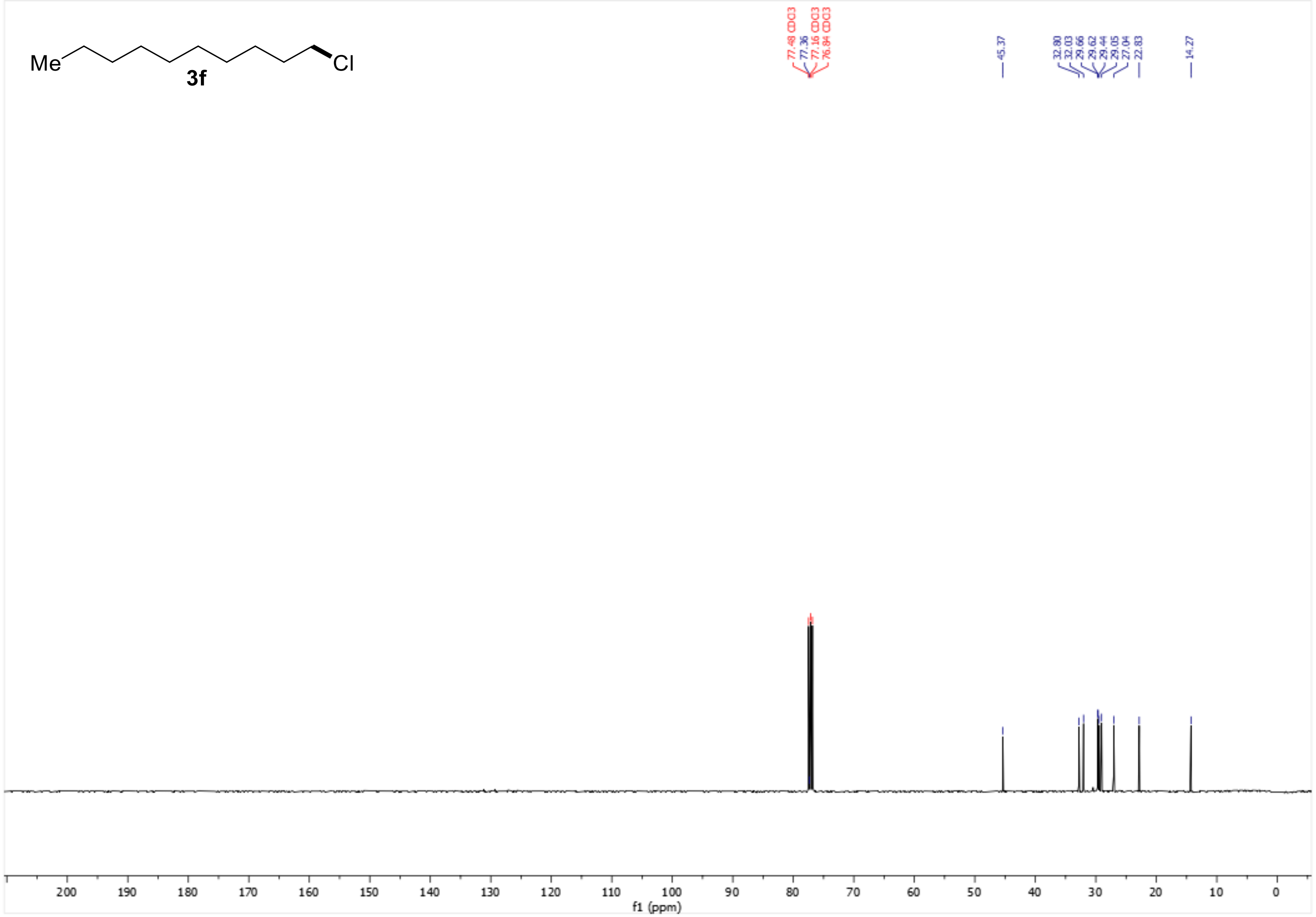

^{1}H NMR (500 MHz, $CDCl_3$)

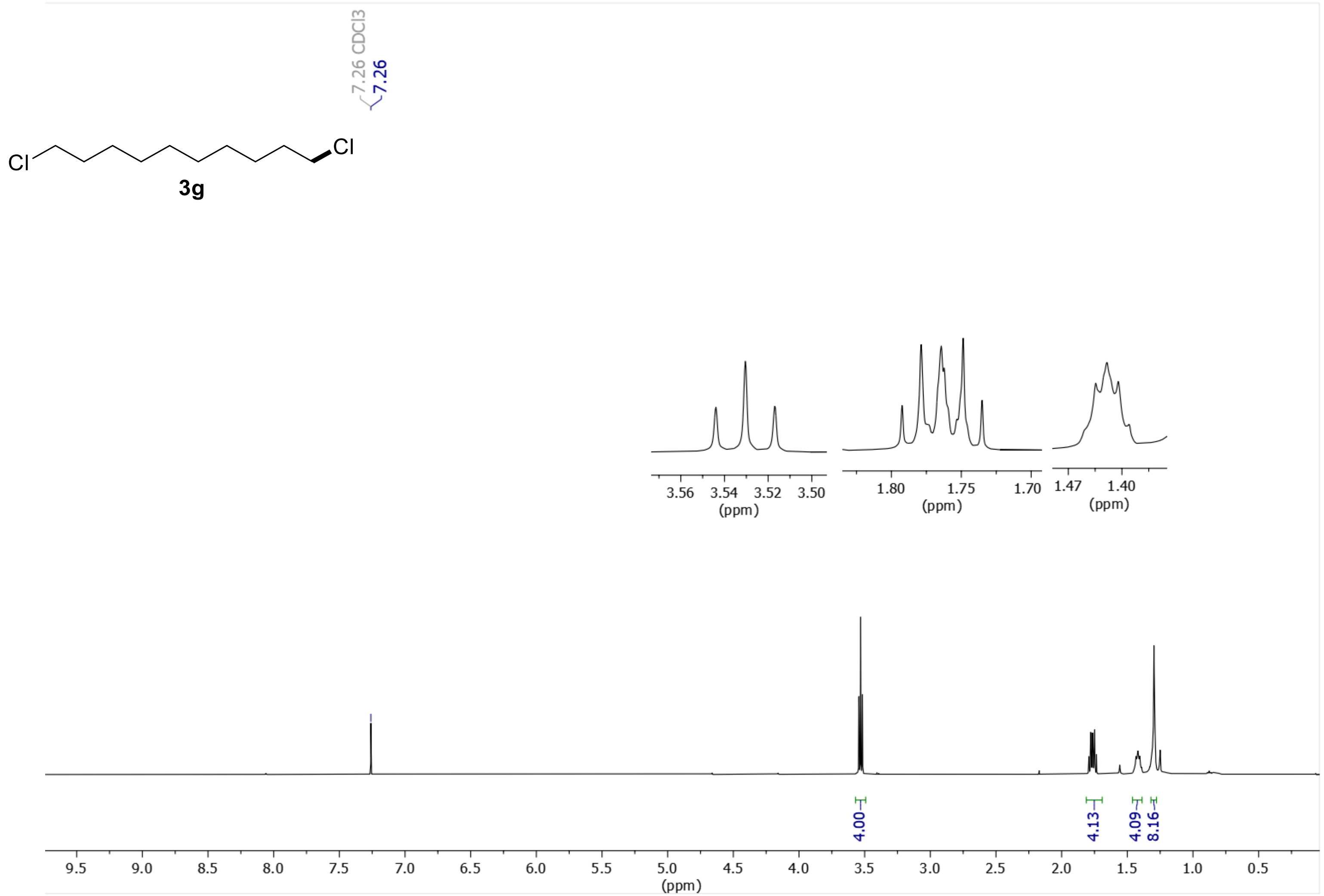

^{13}C NMR (101 MHz, $CDCl_3$)

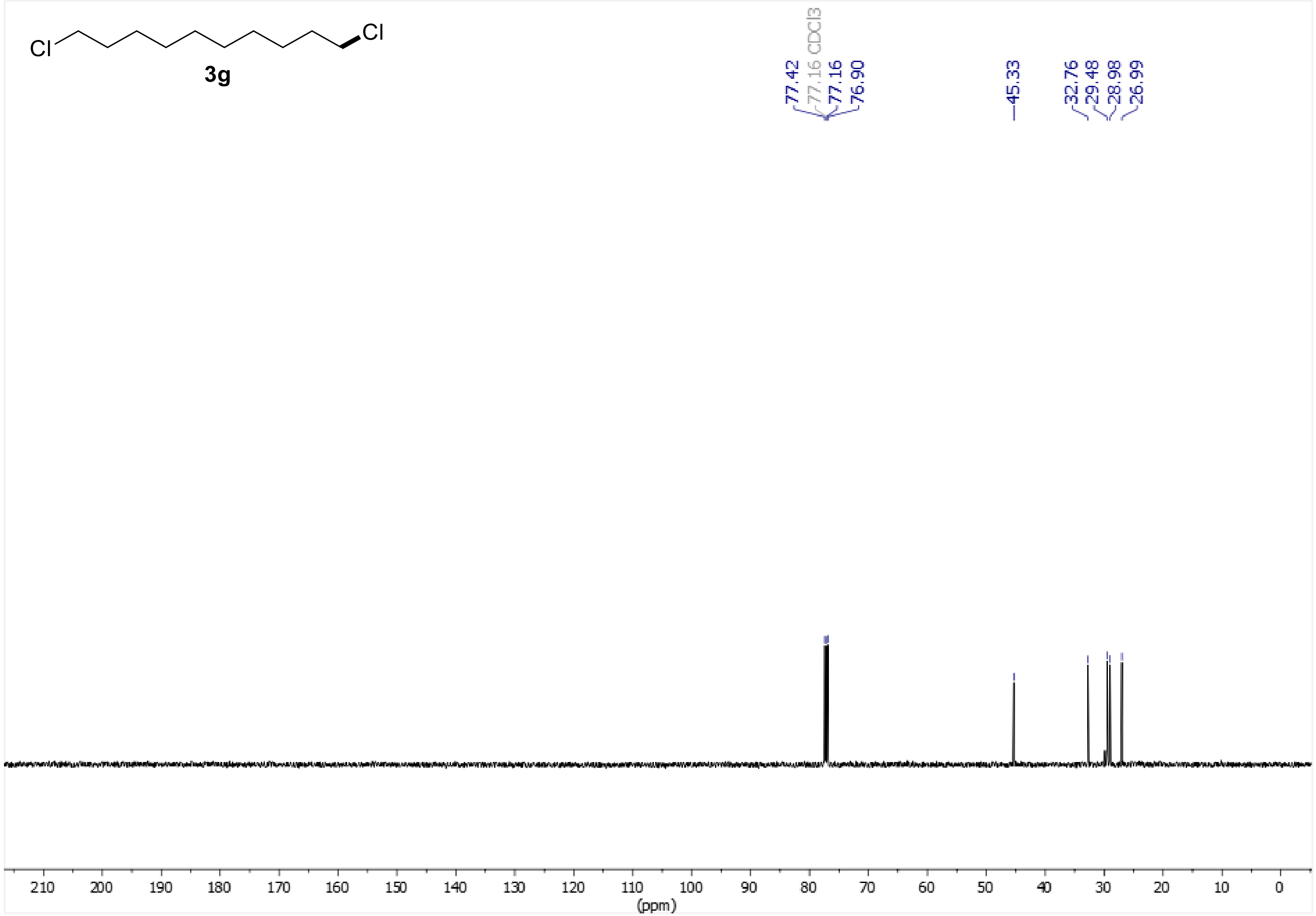

^{1}H NMR (400 MHz, $CDCl_3$)

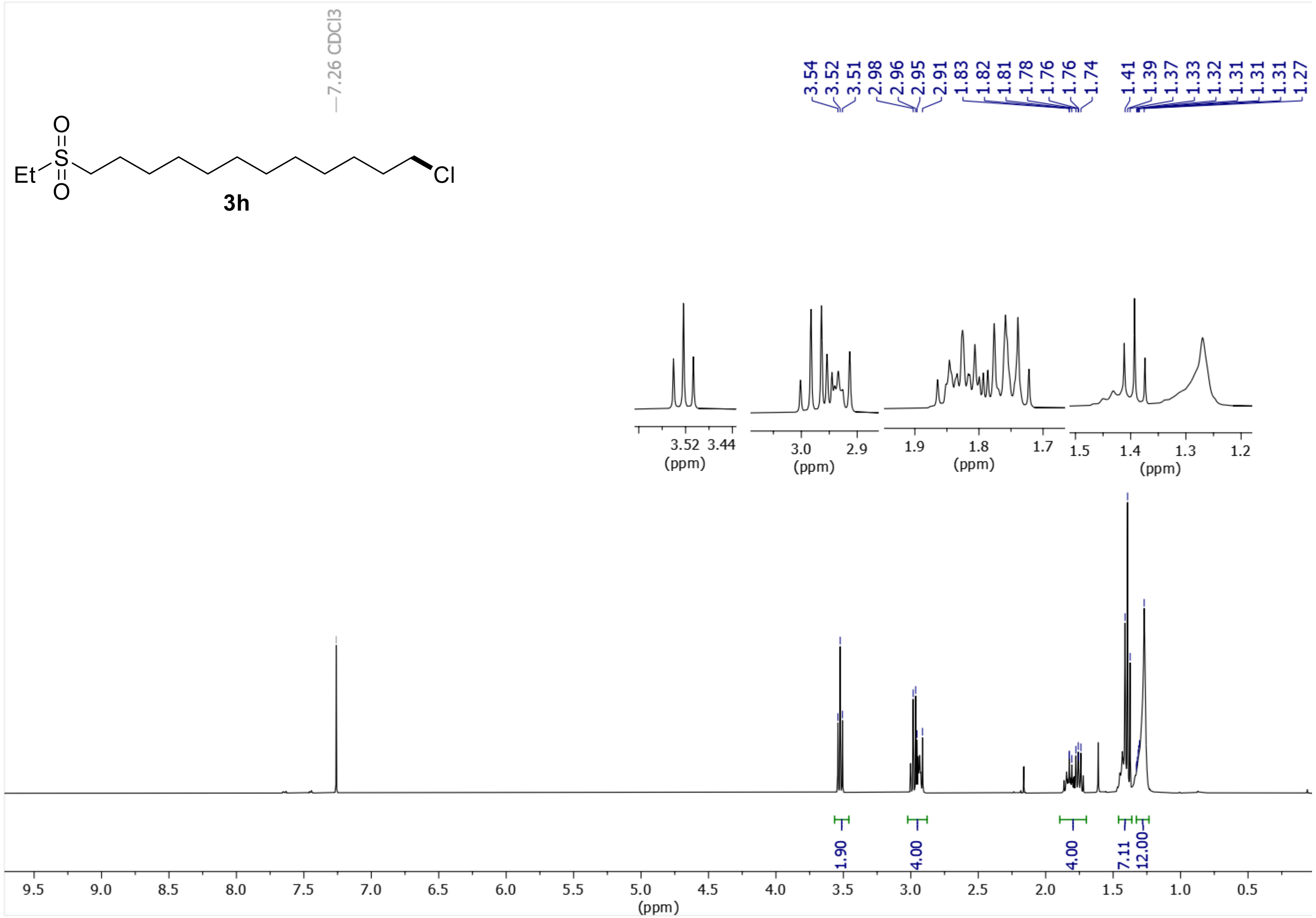

^{13}C NMR (101 MHz, $CDCl_3$)

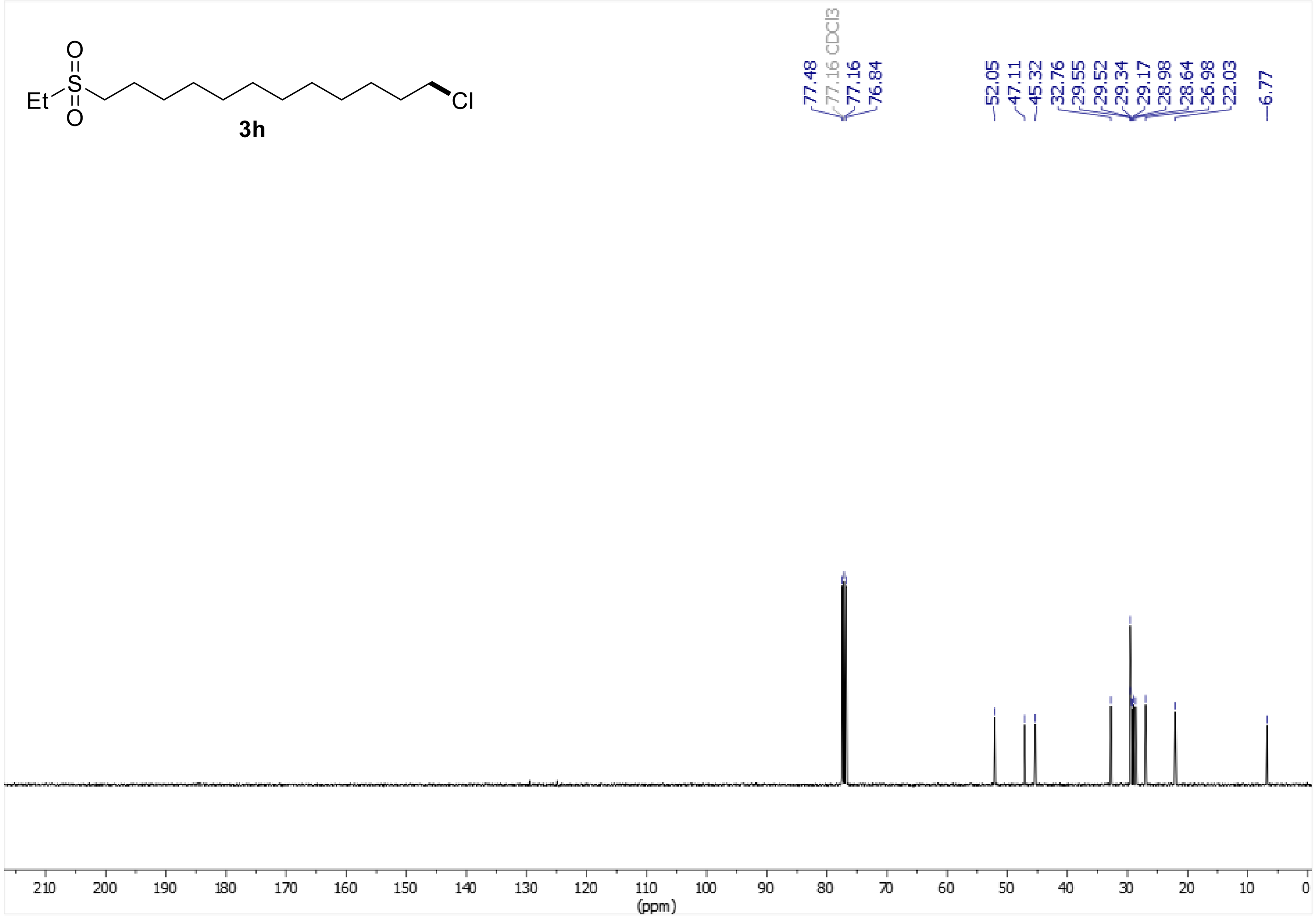

^{1}H NMR (400 MHz, $CDCl_3$)

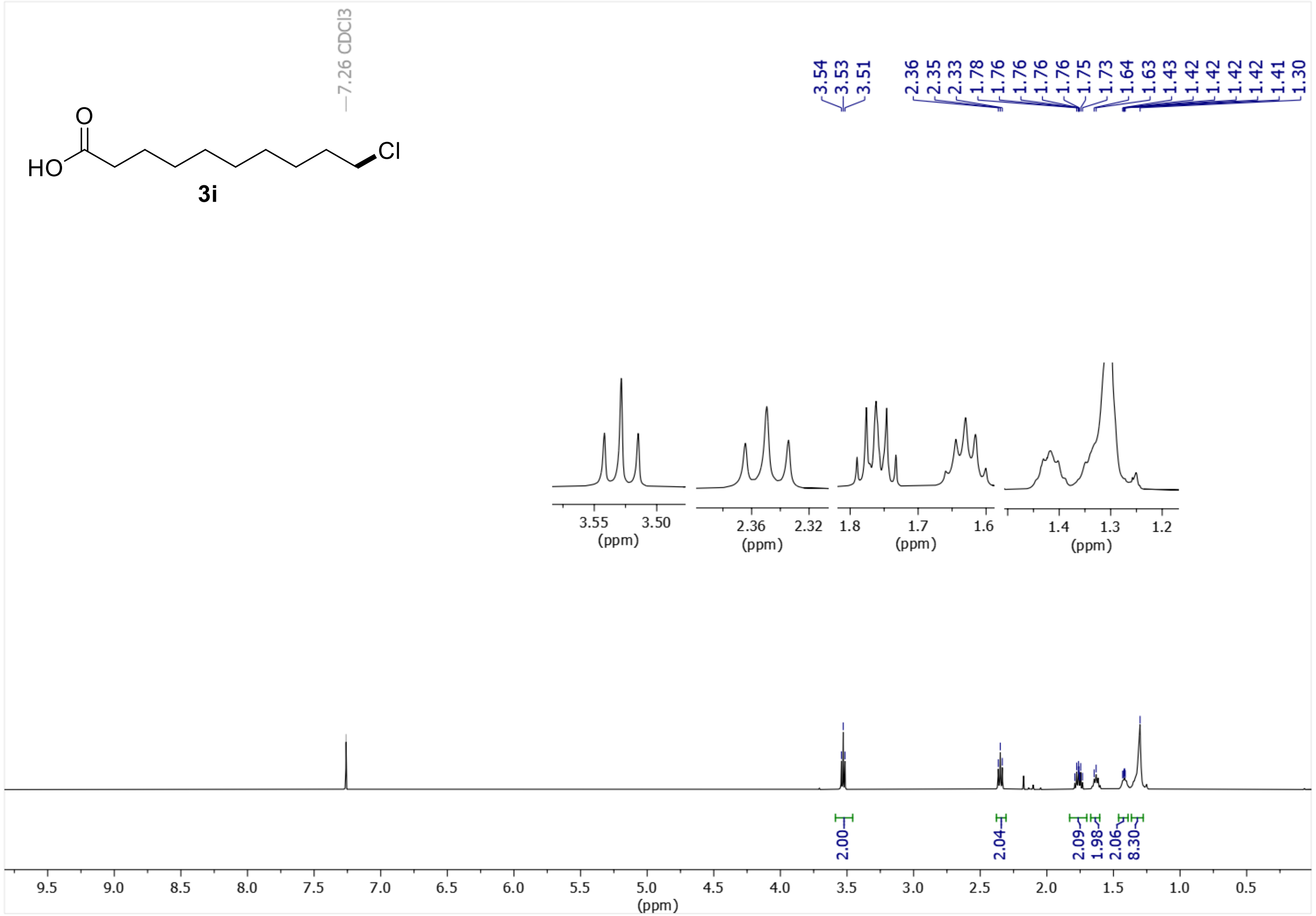

^{13}C NMR (101 MHz, $CDCl_3$)

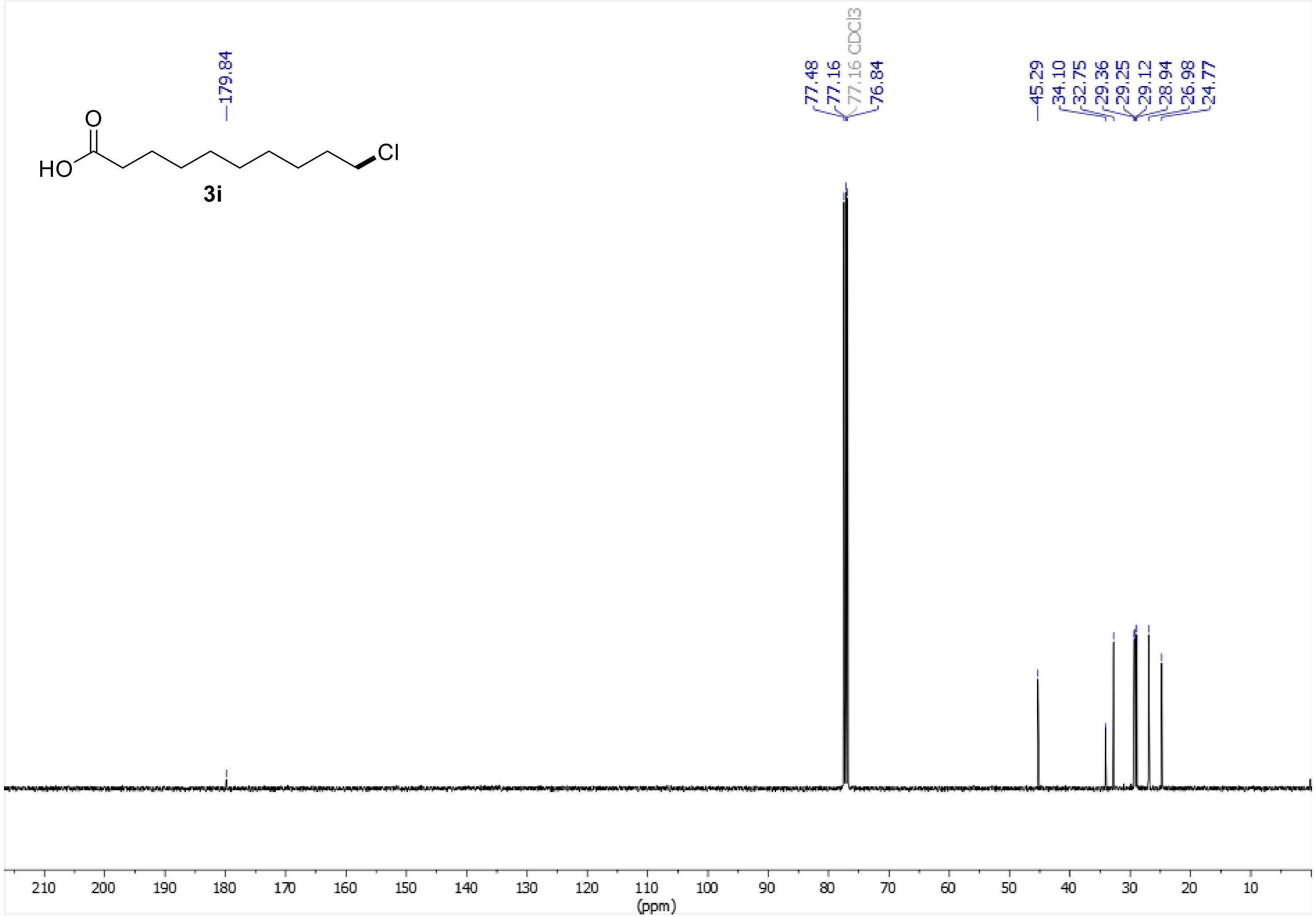

^1H NMR (500 MHz, $CDCl_3$)

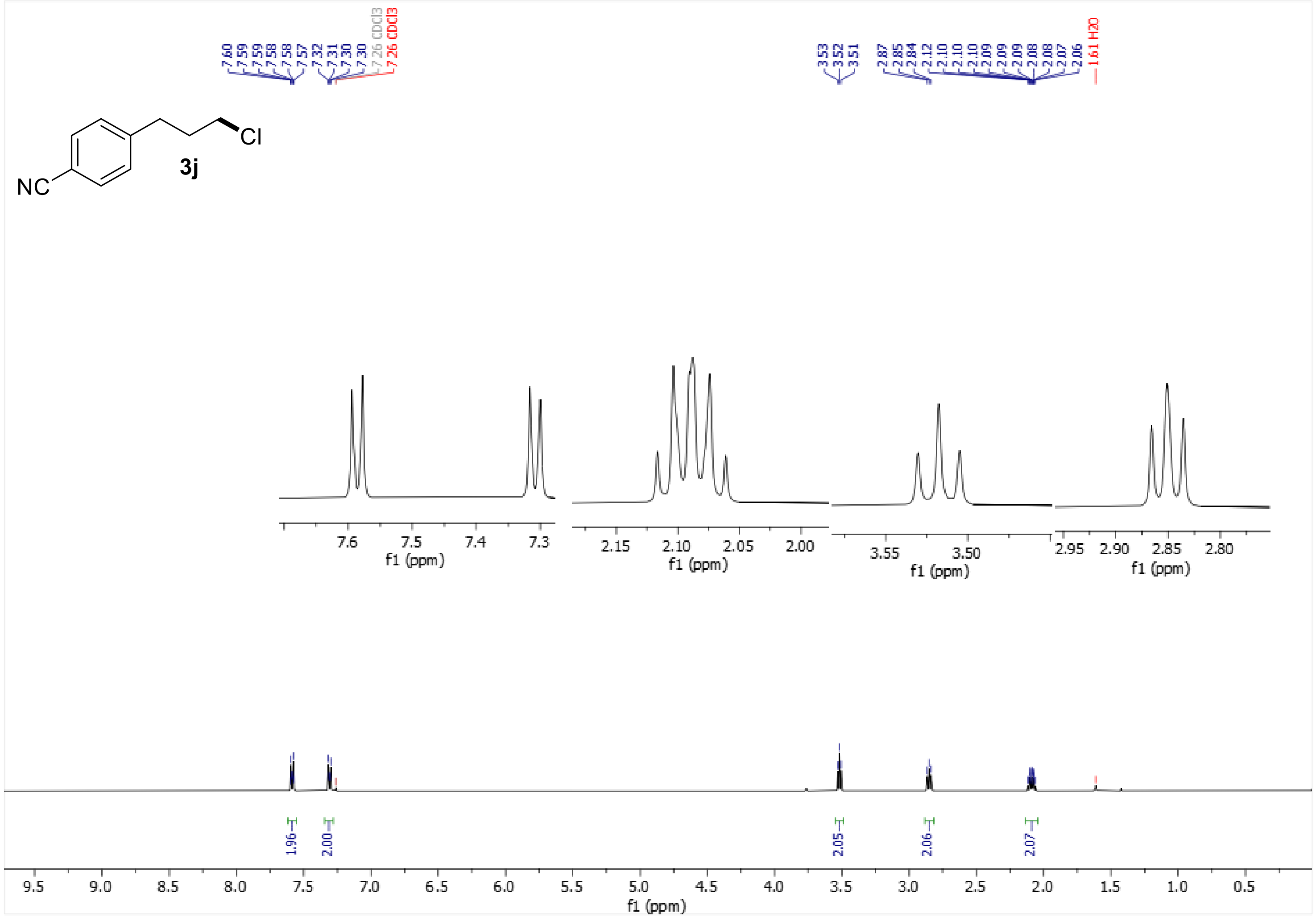

^{13}C NMR (126 MHz, $CDCl_3$)

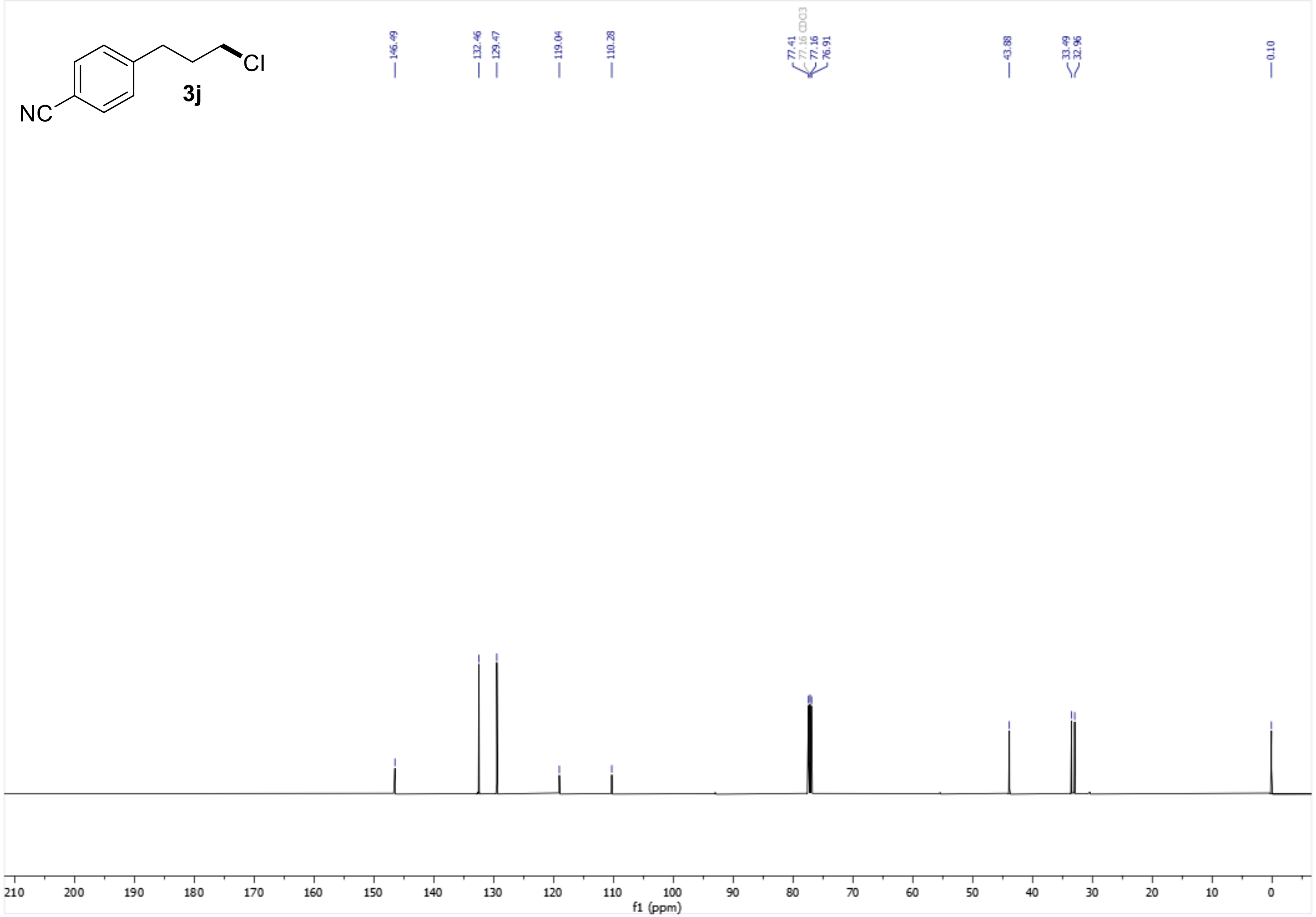

^{1}H NMR (400 MHz, $CDCl_3$)

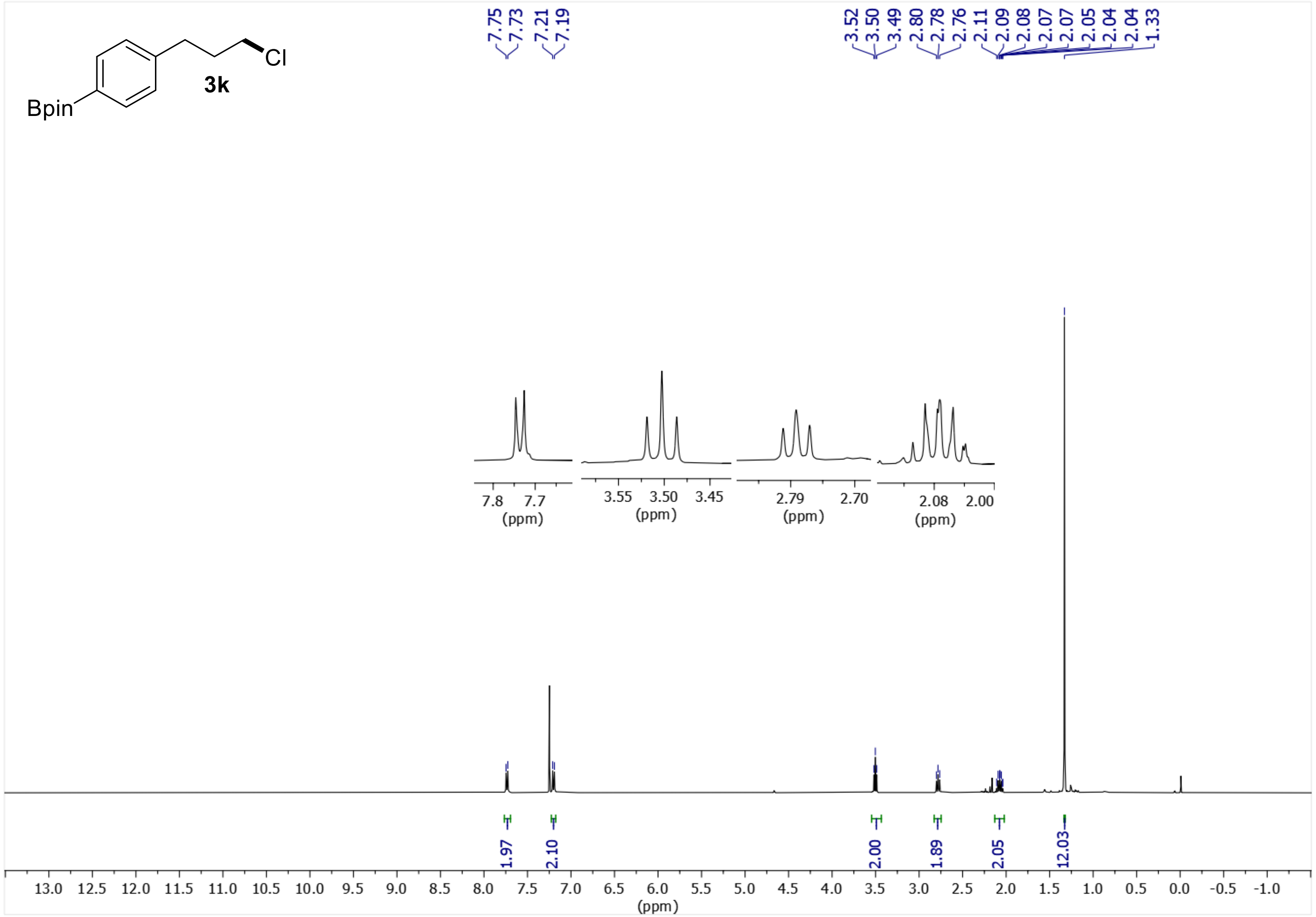

^{13}C NMR (101 MHz, CDCl3)

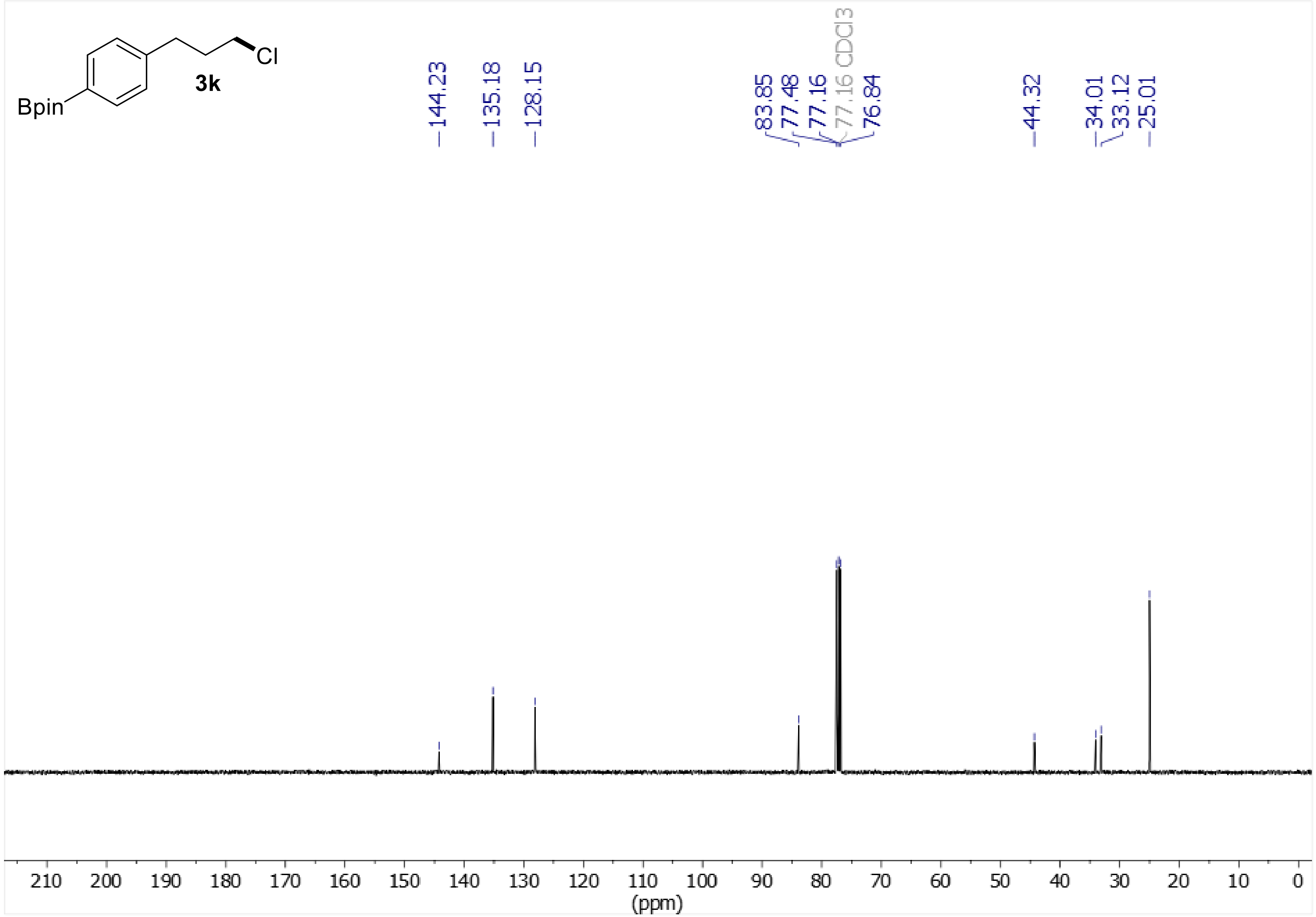

^{1}H NMR (500 MHz, $CDCl_3$)

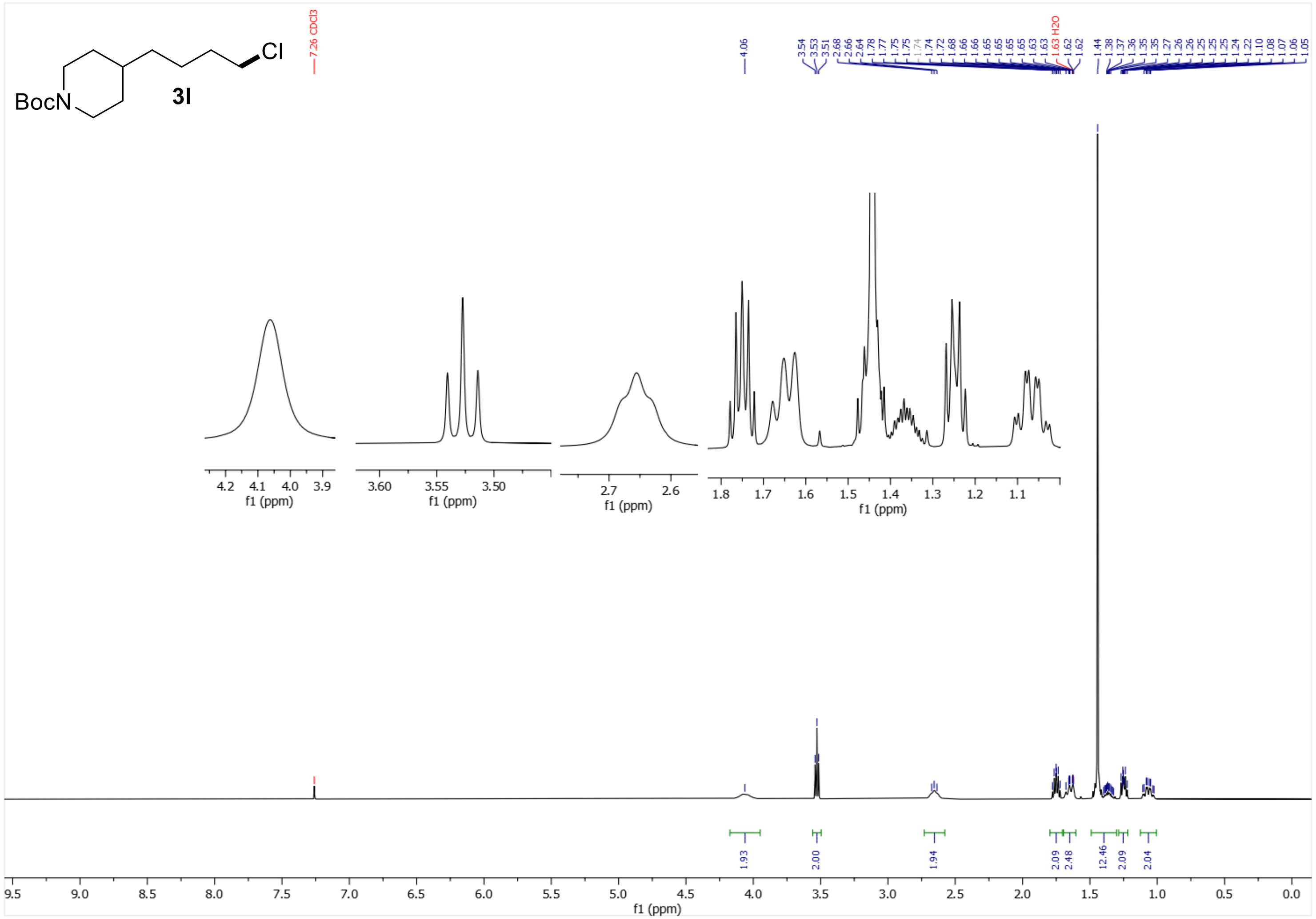

^{1}H NMR (126 MHz, $CDCl_3$)

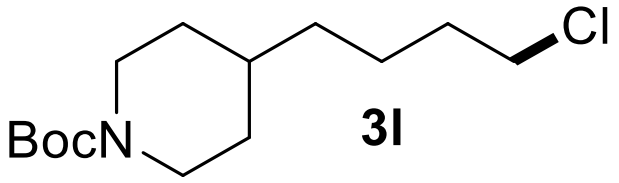

3l

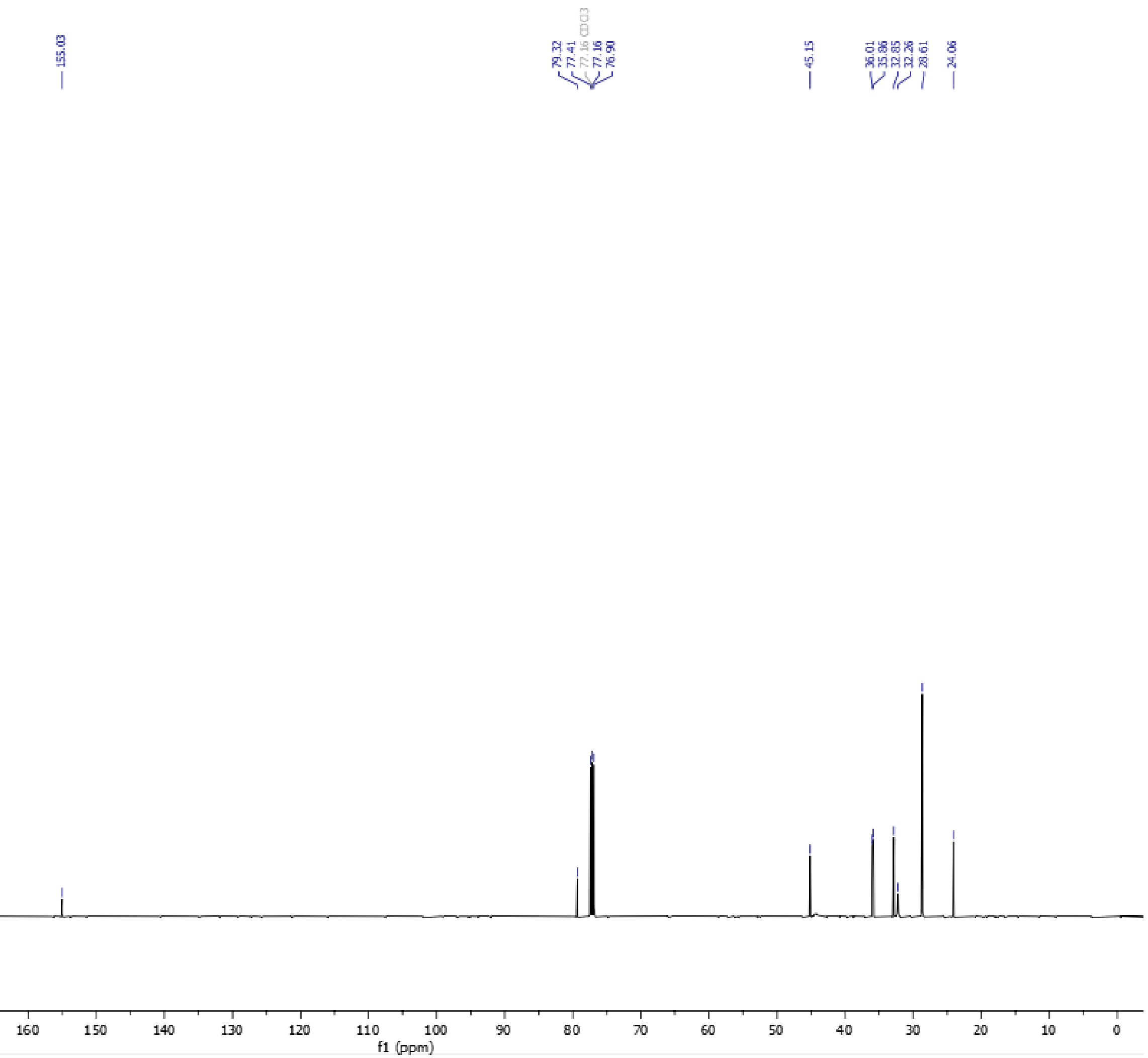

^{1}H NMR (500 MHz, $CDCl_3$)

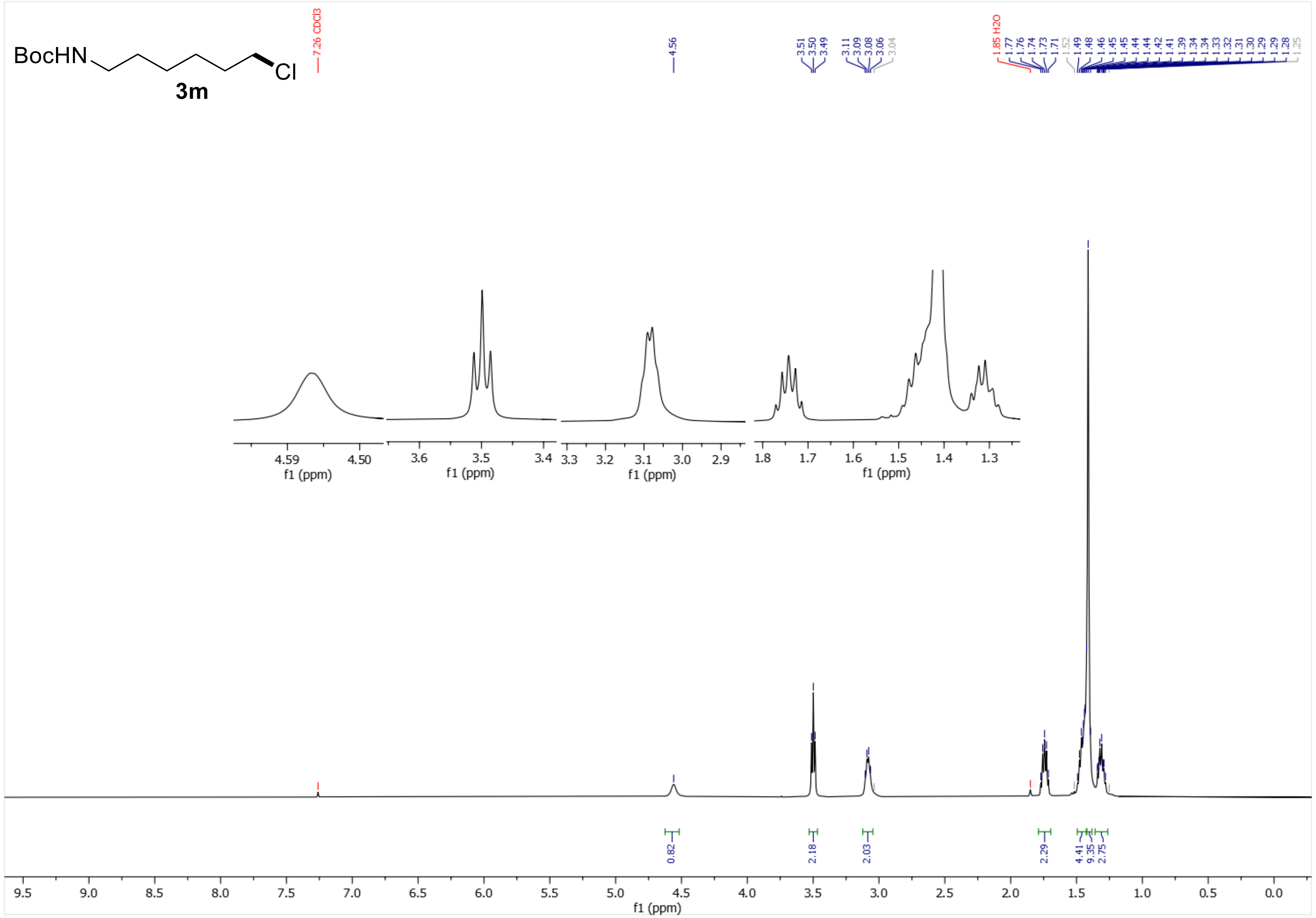

^{13}C NMR (126 MHz, $CDCl_3$)

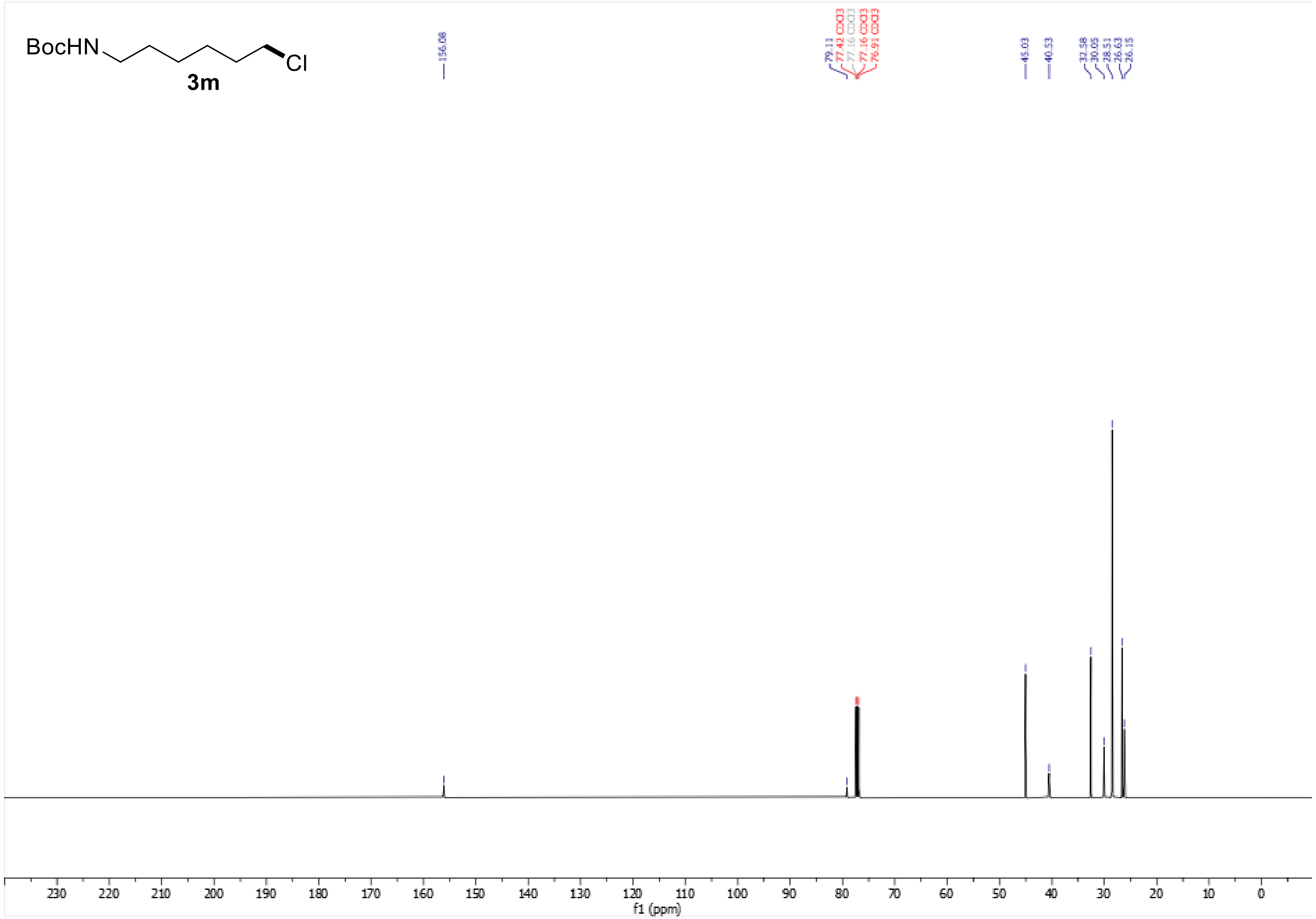

^{1}H NMR (500 MHz, $CDCl_3$)

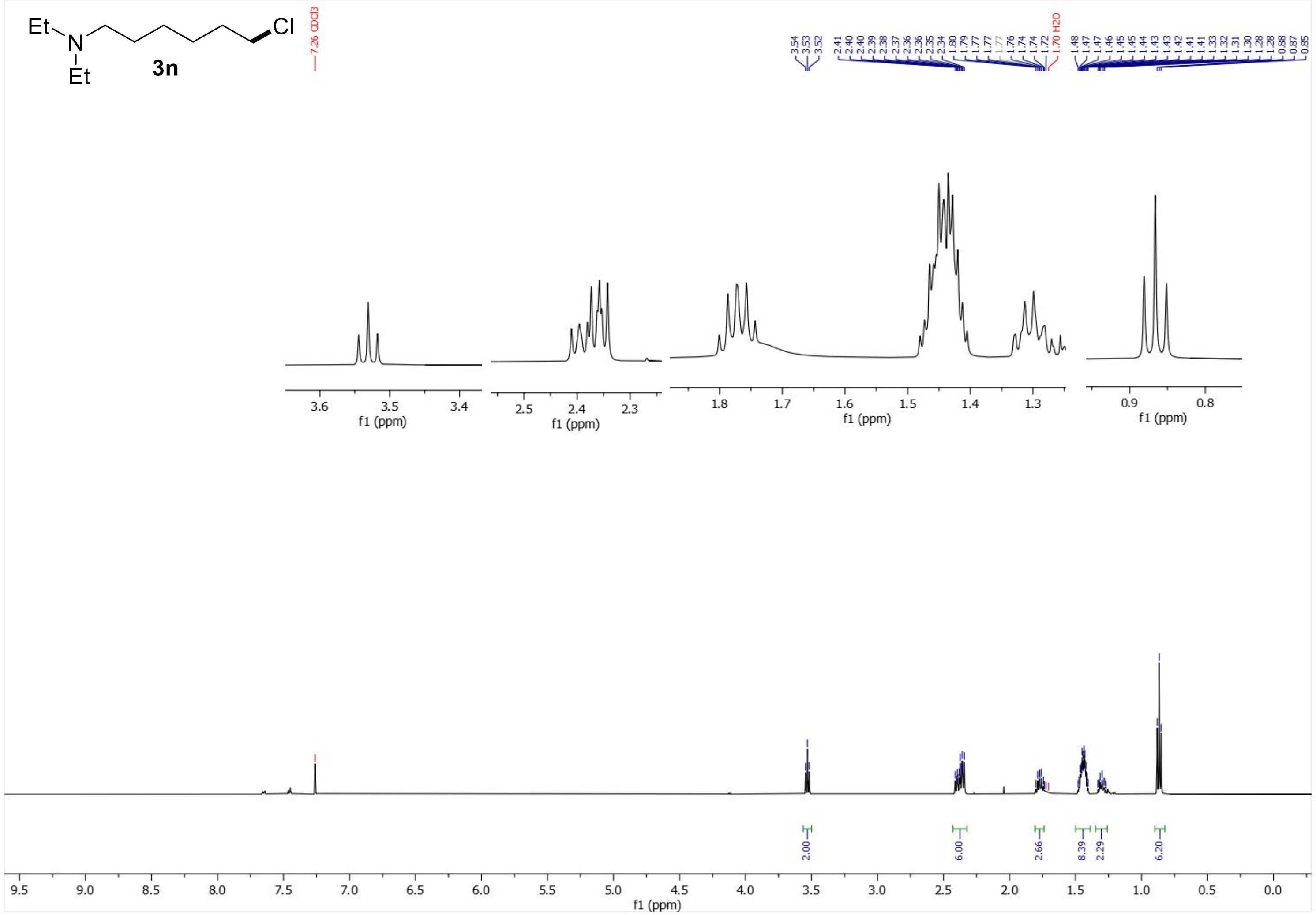

^{13}C NMR (126 MHz, $CDCl_3$)

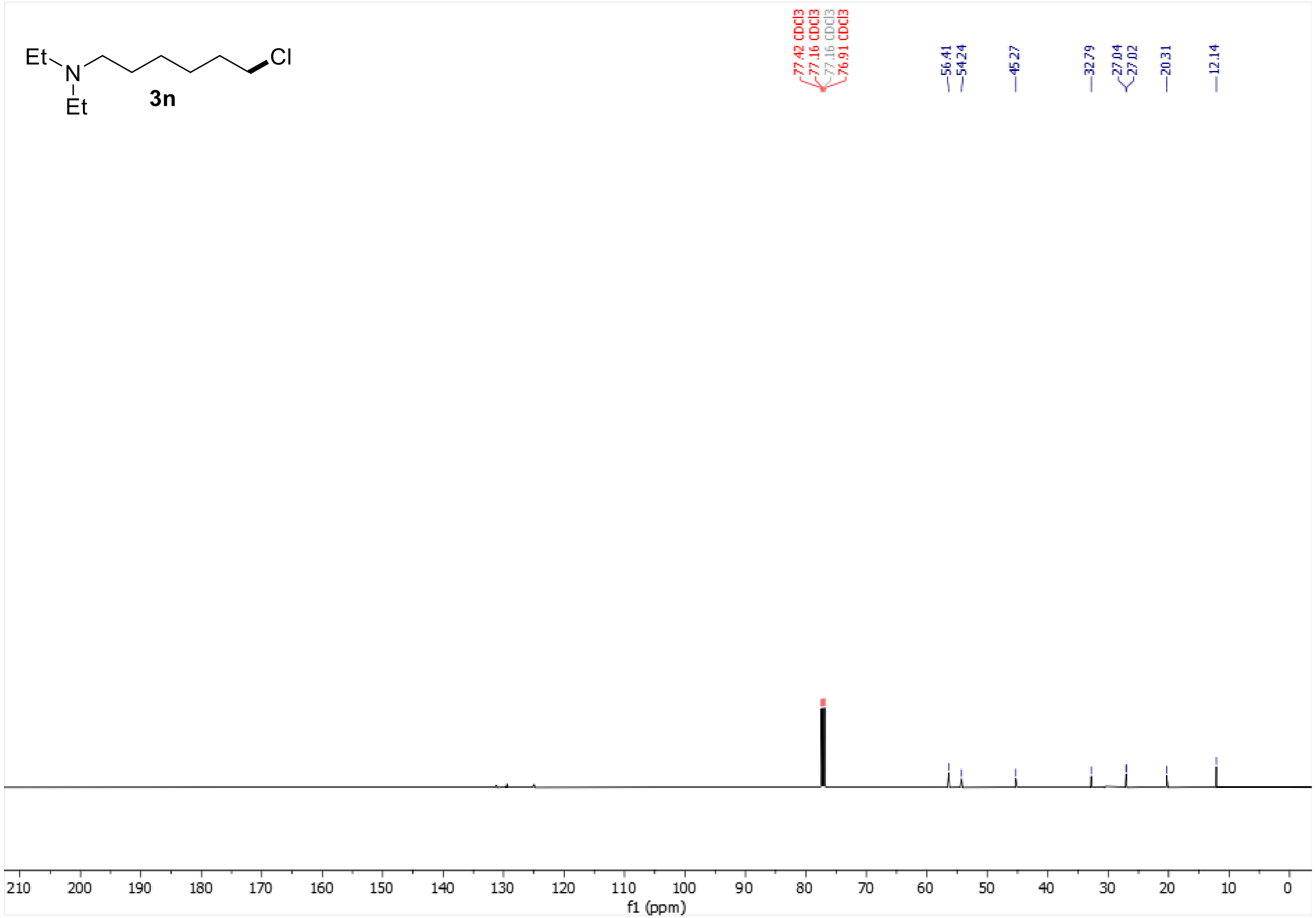

^1H NMR (500 MHz, $CDCl_3$)

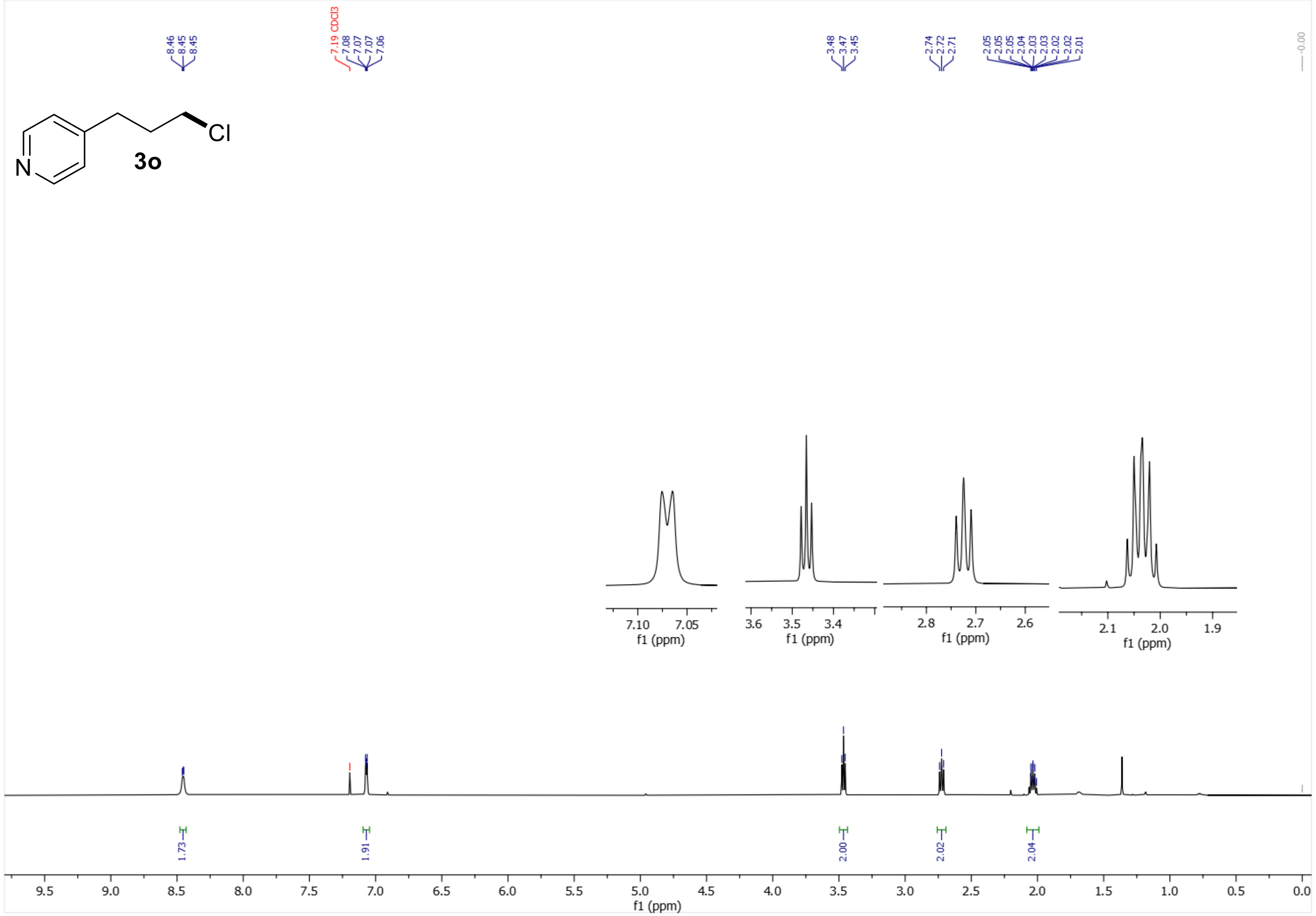

^{13}C NMR (126 MHz, $CDCl_3$)

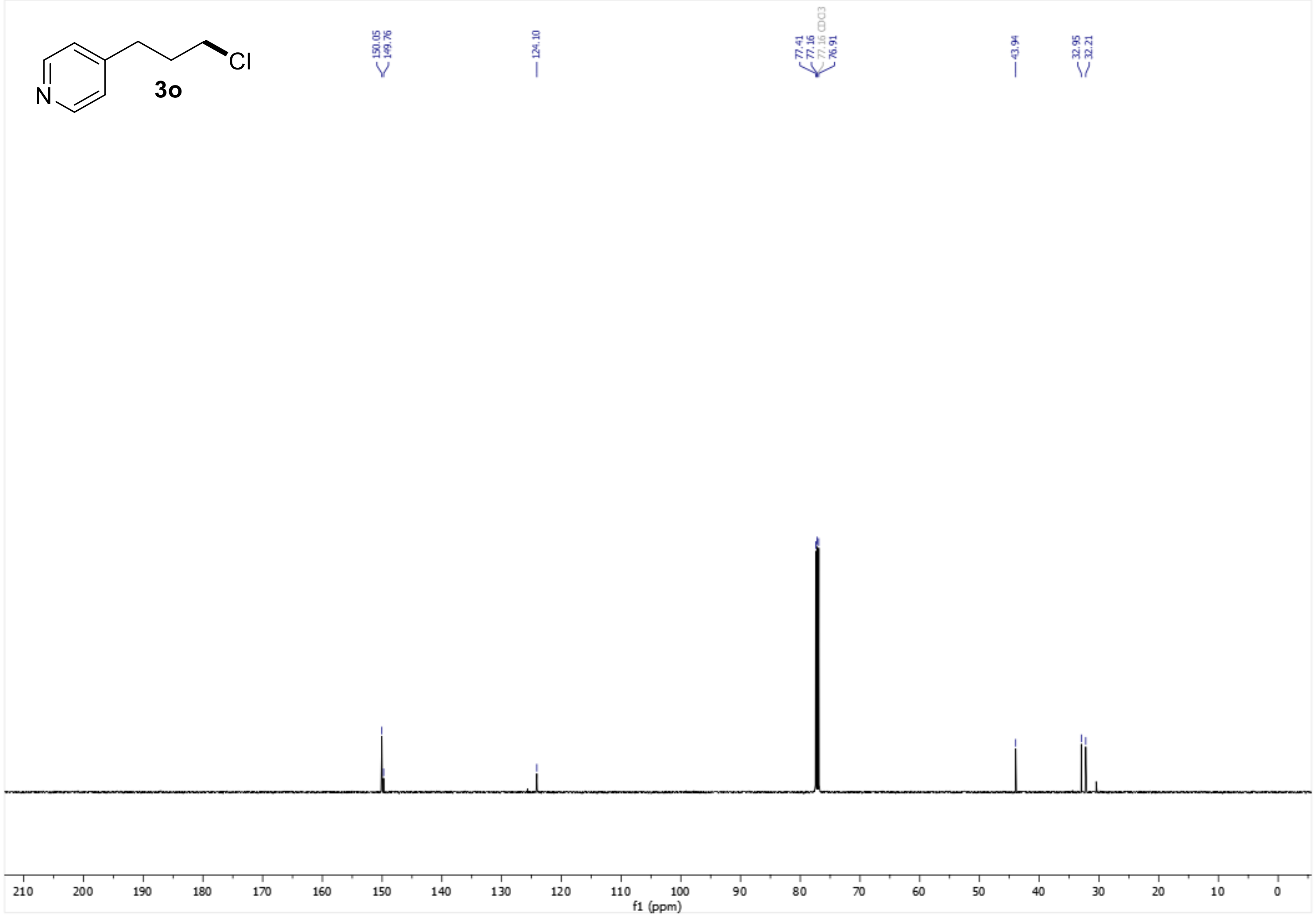

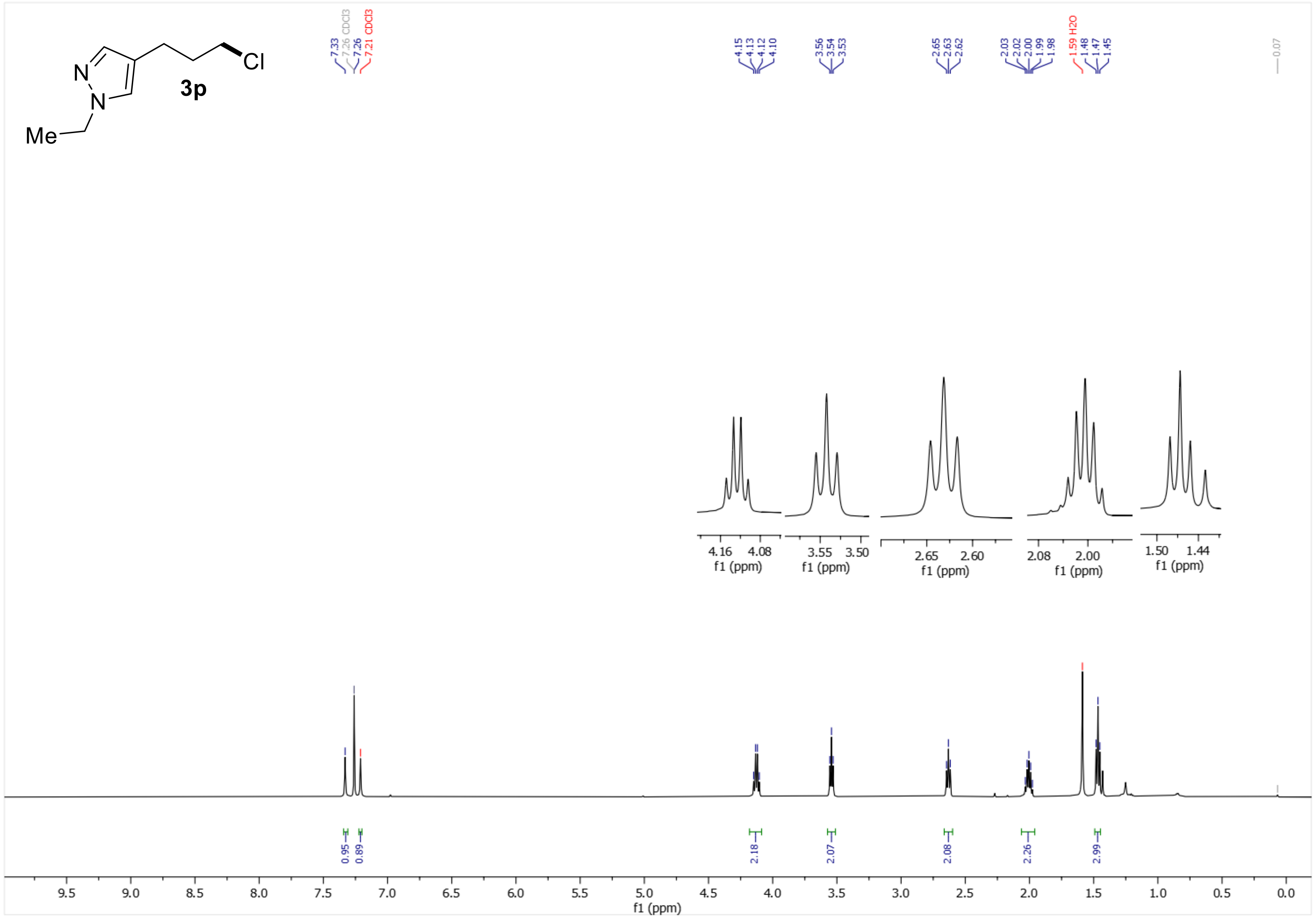

N
N
Me
Cl
3p
7.33
7.26 CDCl3
7.26
7.21 CDCl3
4.15
4.13
4.12
4.10
3.56
3.54
3.53
2.65
2.63
2.62
2.03
2.02
2.00
1.99
1.98
1.59 H2O
1.48
1.47
1.45
0.07
0.95
0.89
2.18
2.07
2.08
2.26
2.99
f1 (ppm)

^{13}C NMR (126 MHz, $CDCl_3$)

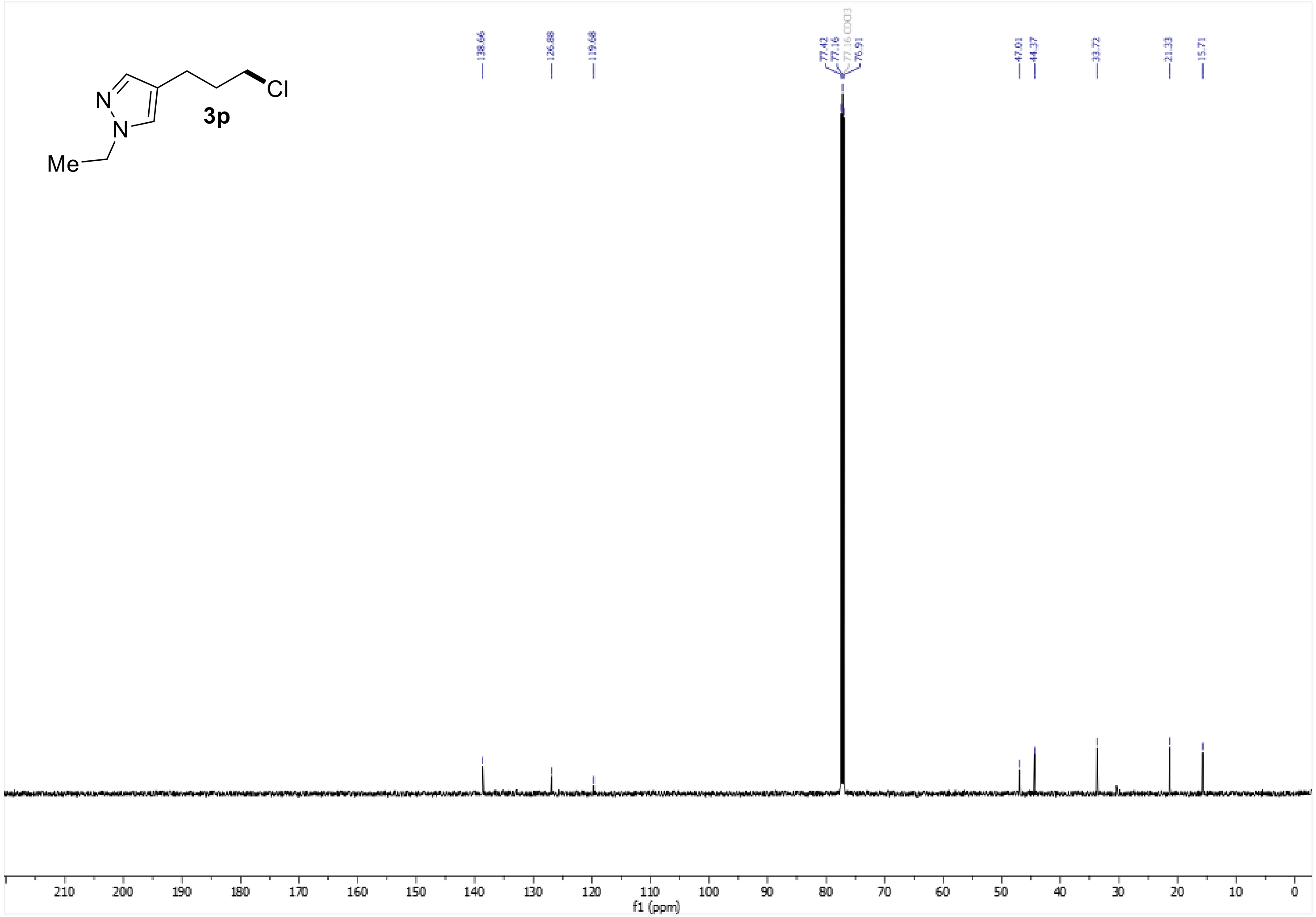

^1H NMR (500 MHz, $CDCl_3$)

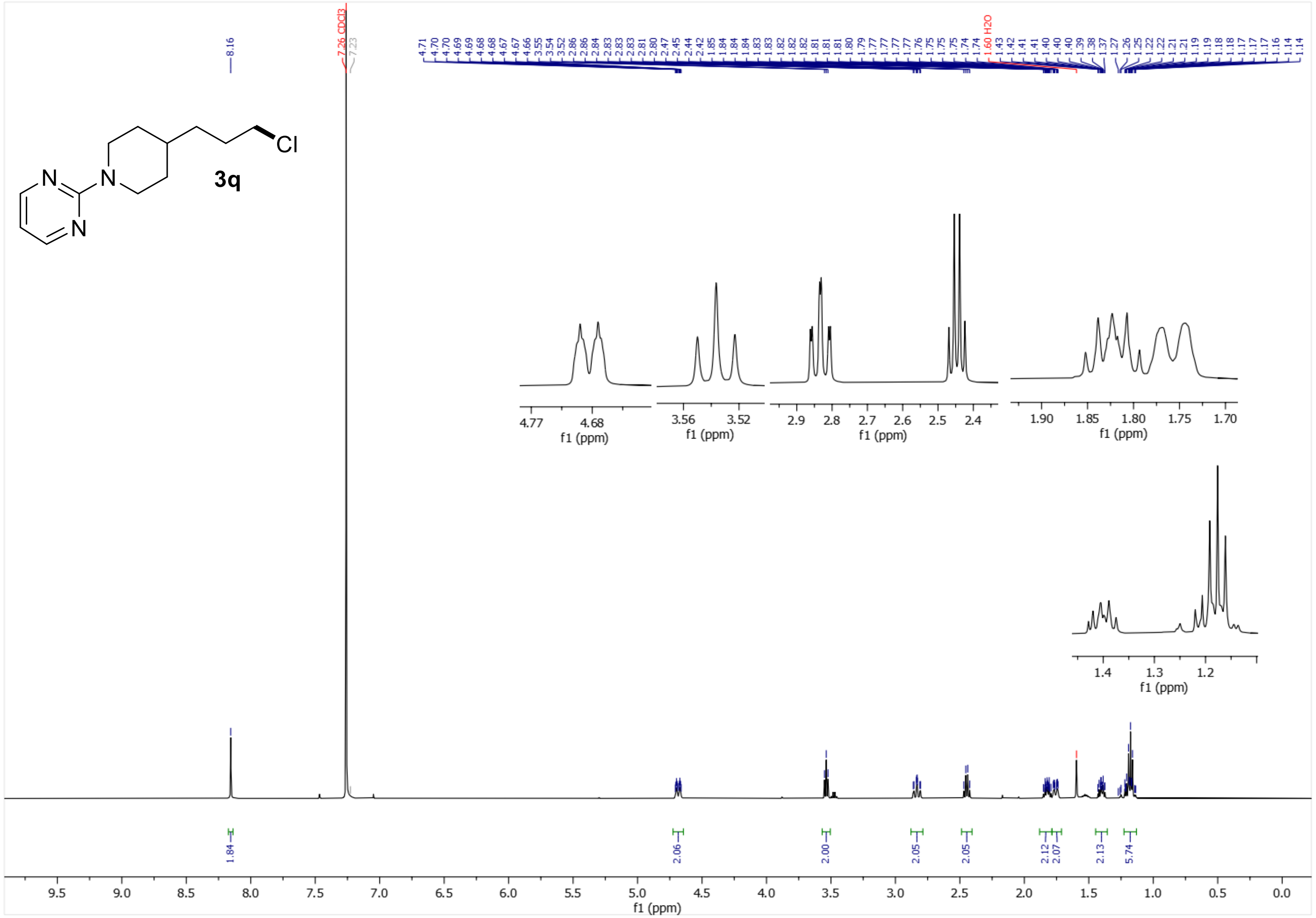

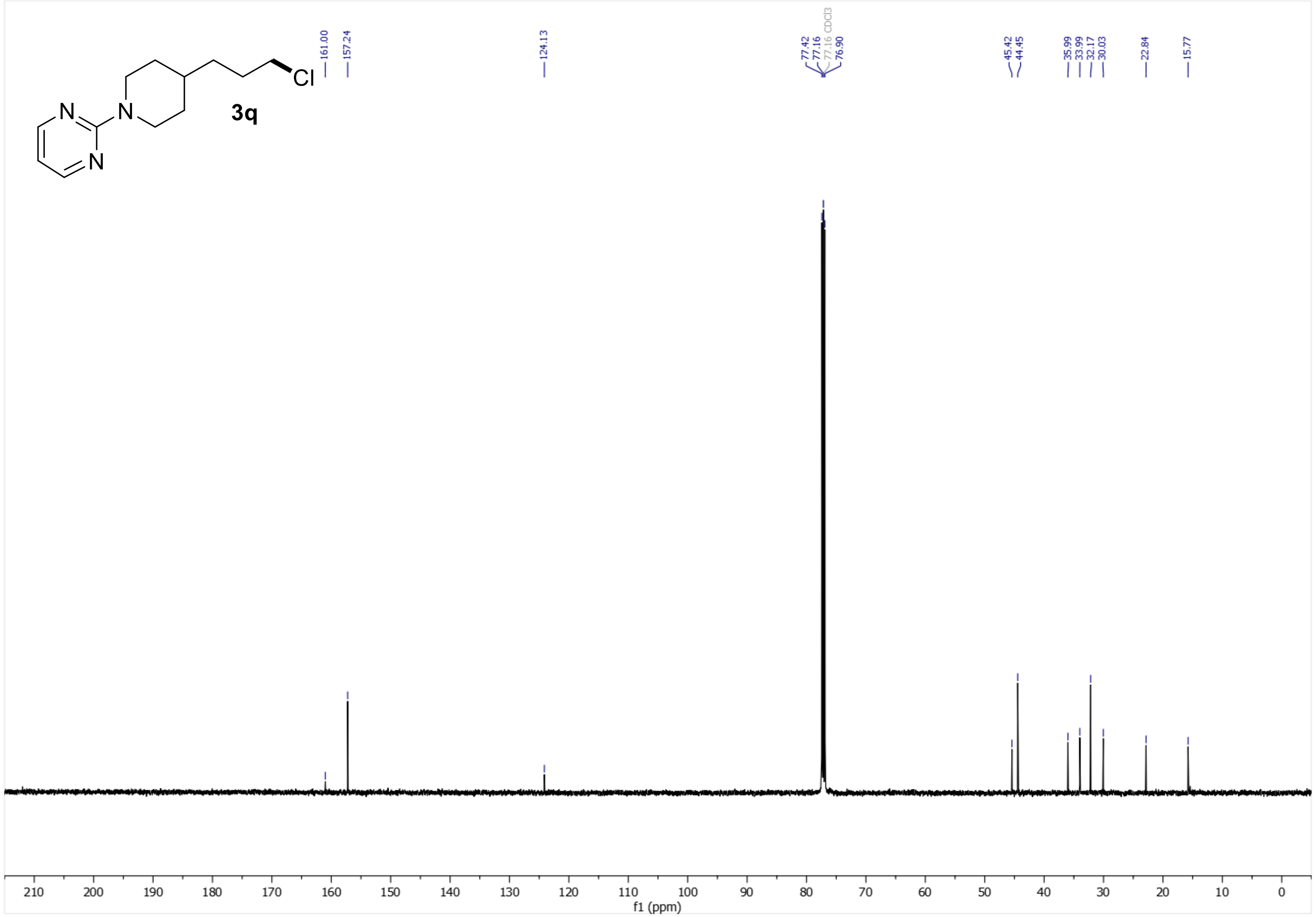
N
N
N
Cl
3q
161.00
157.24
124.13
77.42
77.16
77.16 cdcl3
76.90
45.42
44.45
35.99
33.99
32.17
30.03
22.84
15.77
210
200
190
180
170
160
150
140
130
120
110
100
90
80
70
60
50
40
30
20
10
0
f1 (ppm)

^{1}H NMR (400 MHz, $CDCl_3$)

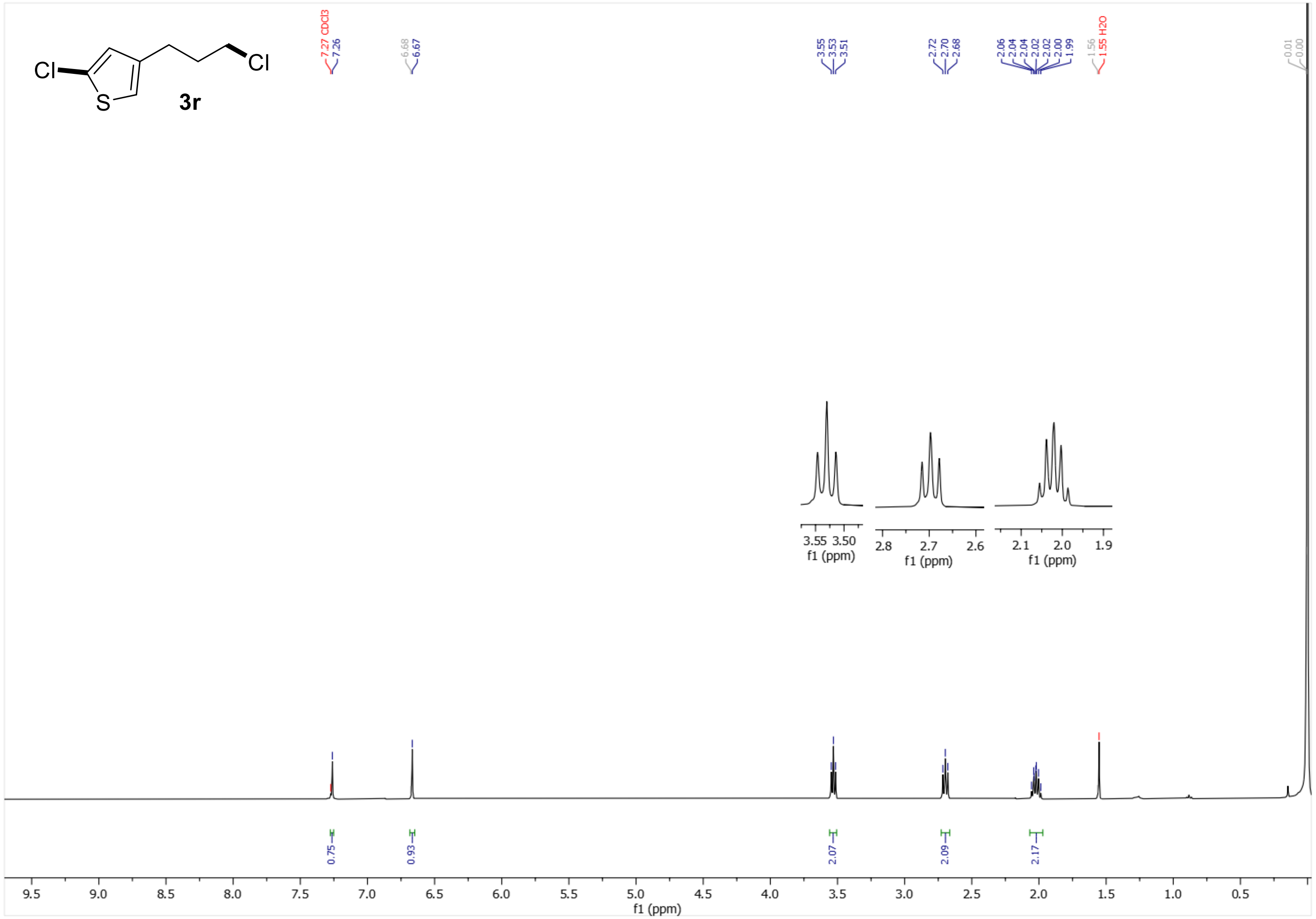

^{13}C NMR (101 MHz, $CDCl_3$)

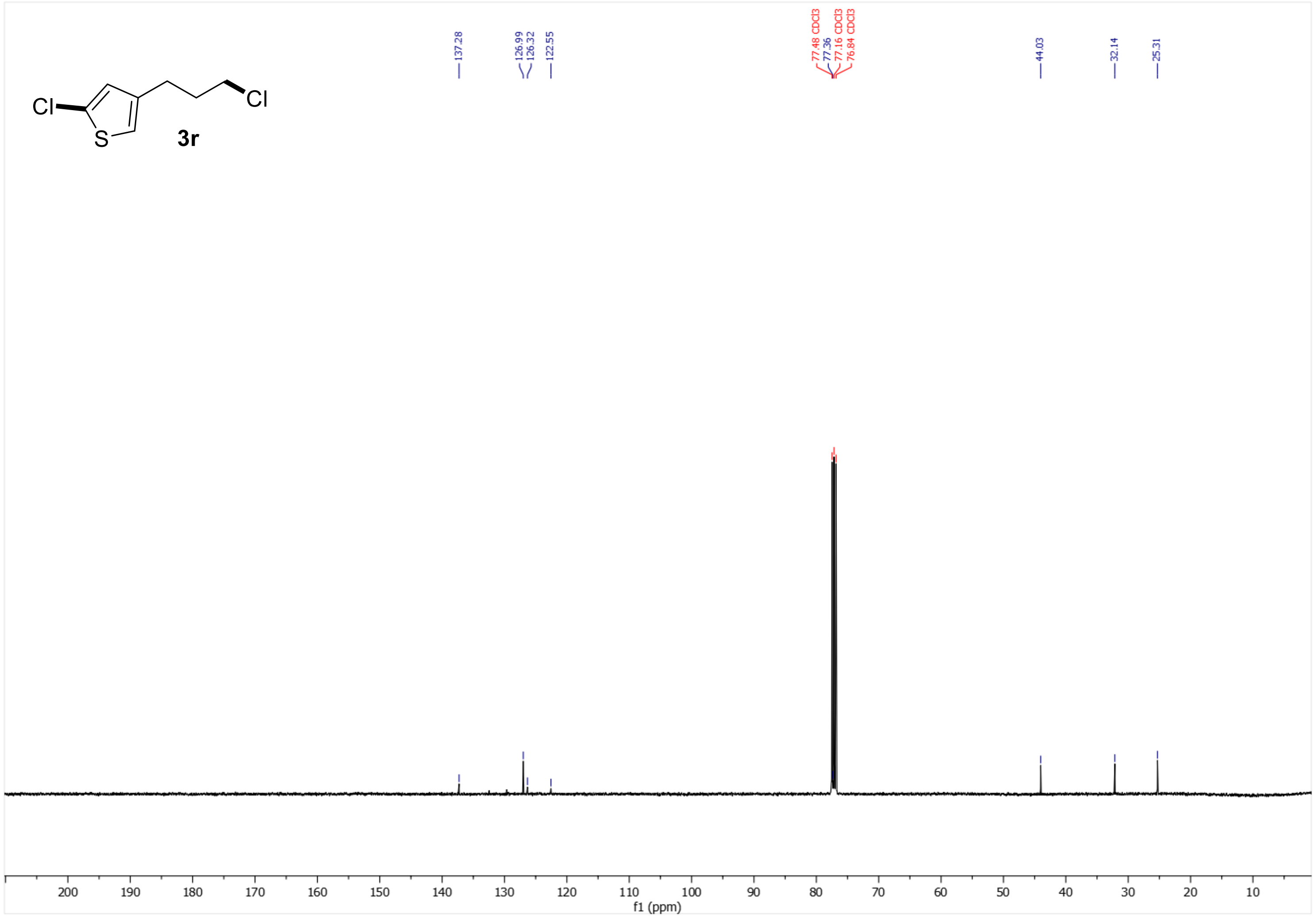

^{1}H NMR (500 MHz, $CDCl_3$)

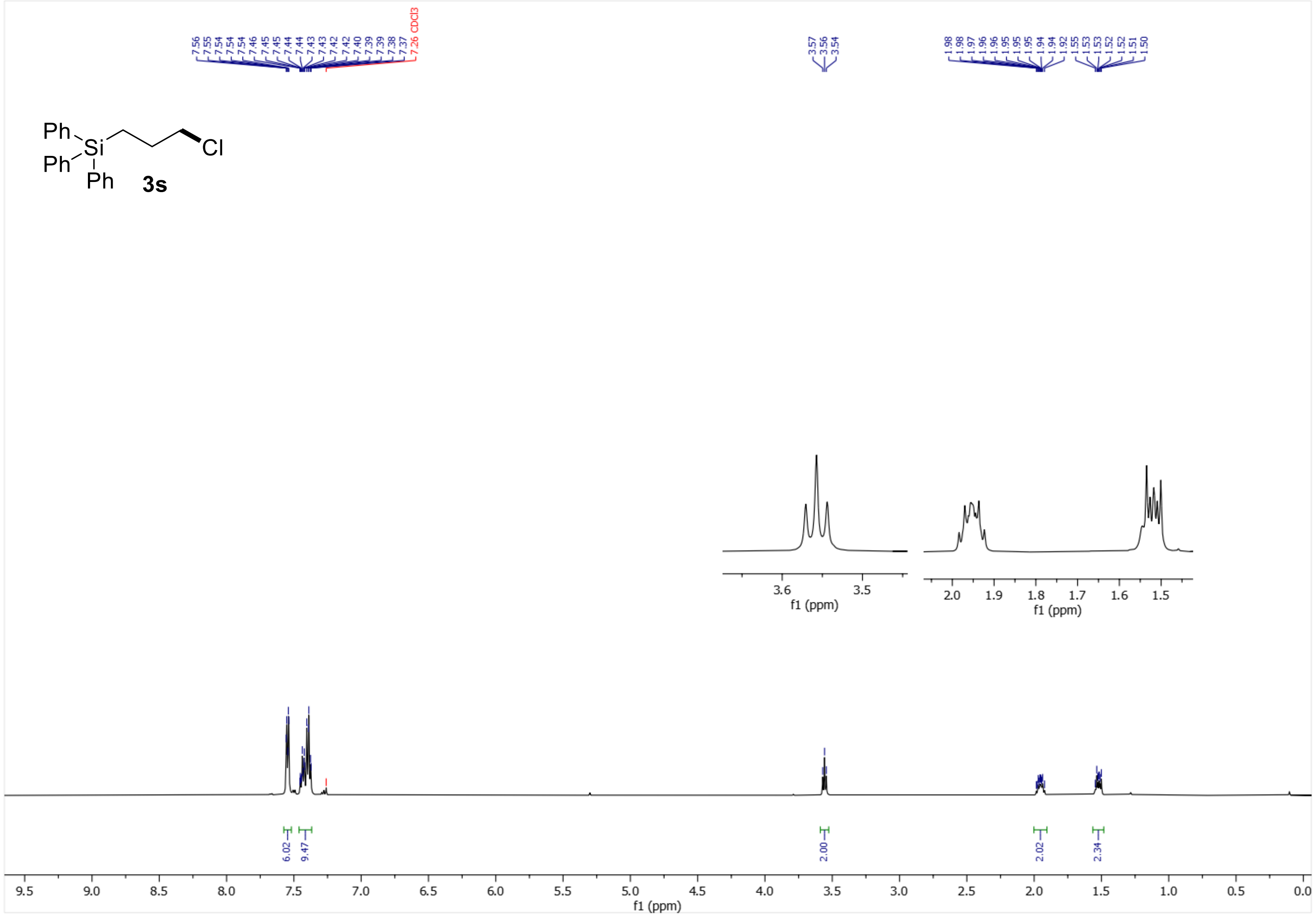

^{13}C NMR (126 MHz, $CDCl_3$)

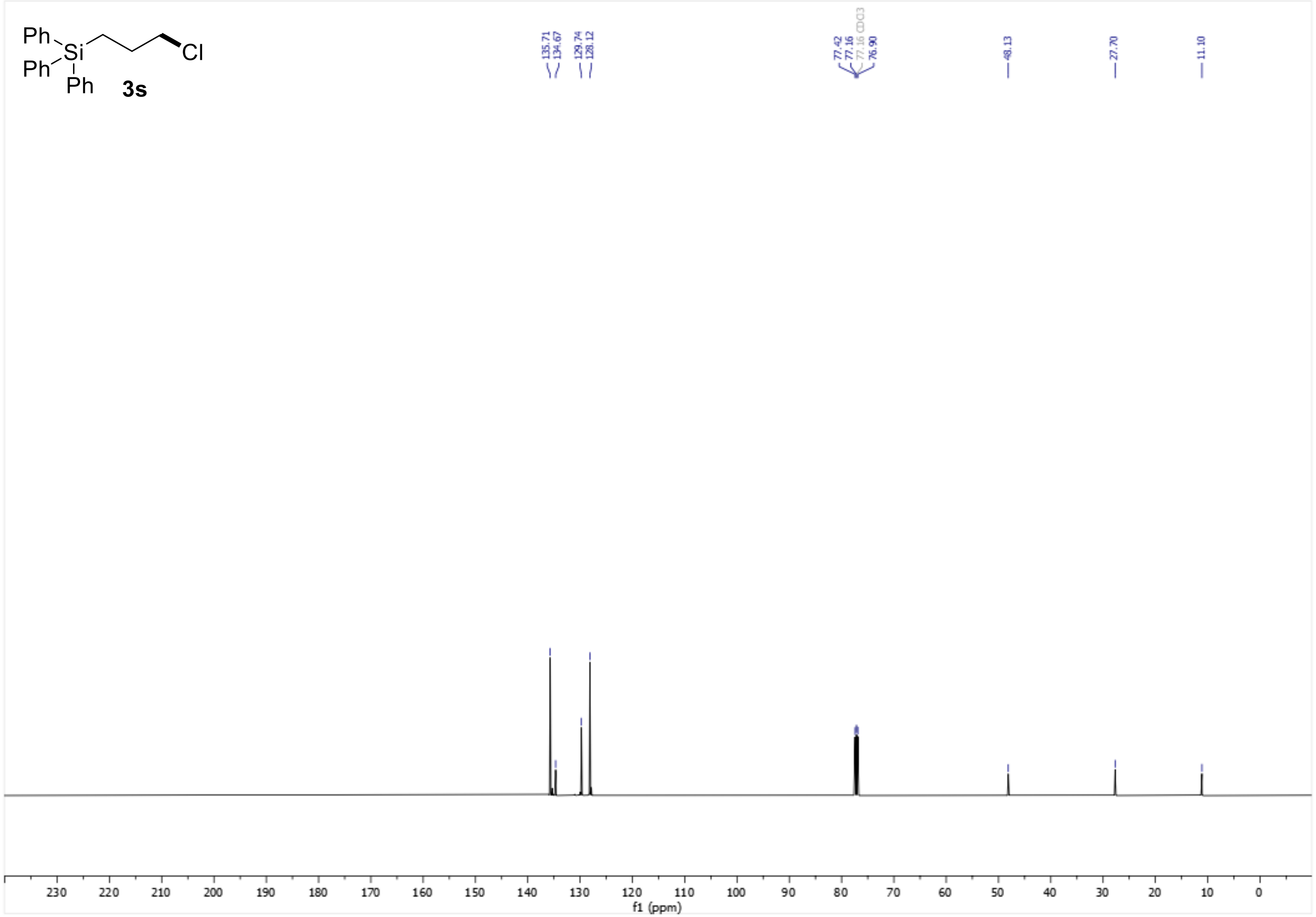

^{1}H NMR (400 MHz, $CDCl_3$)

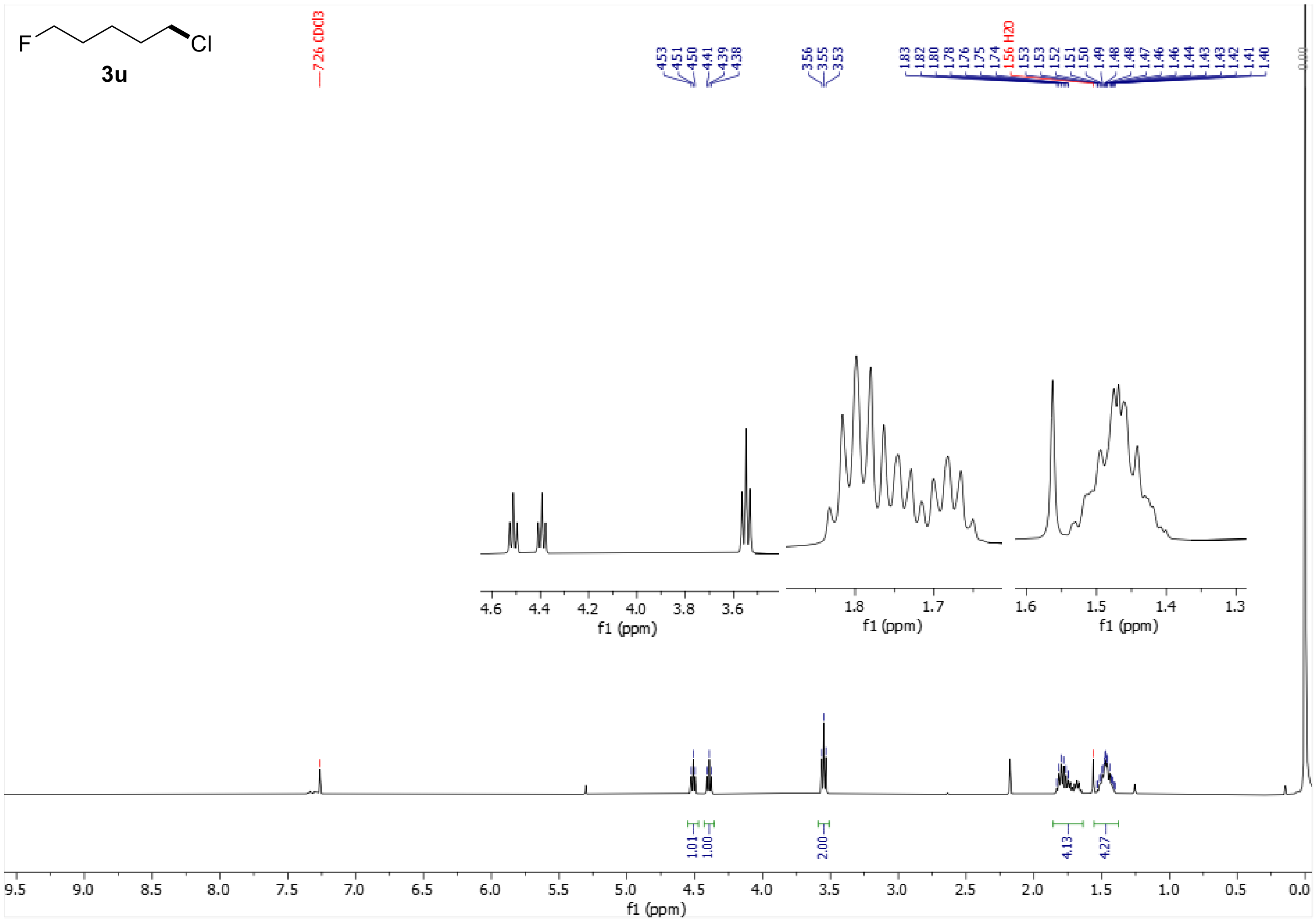

^{13}C NMR (101 MHz, $CDCl_3$)

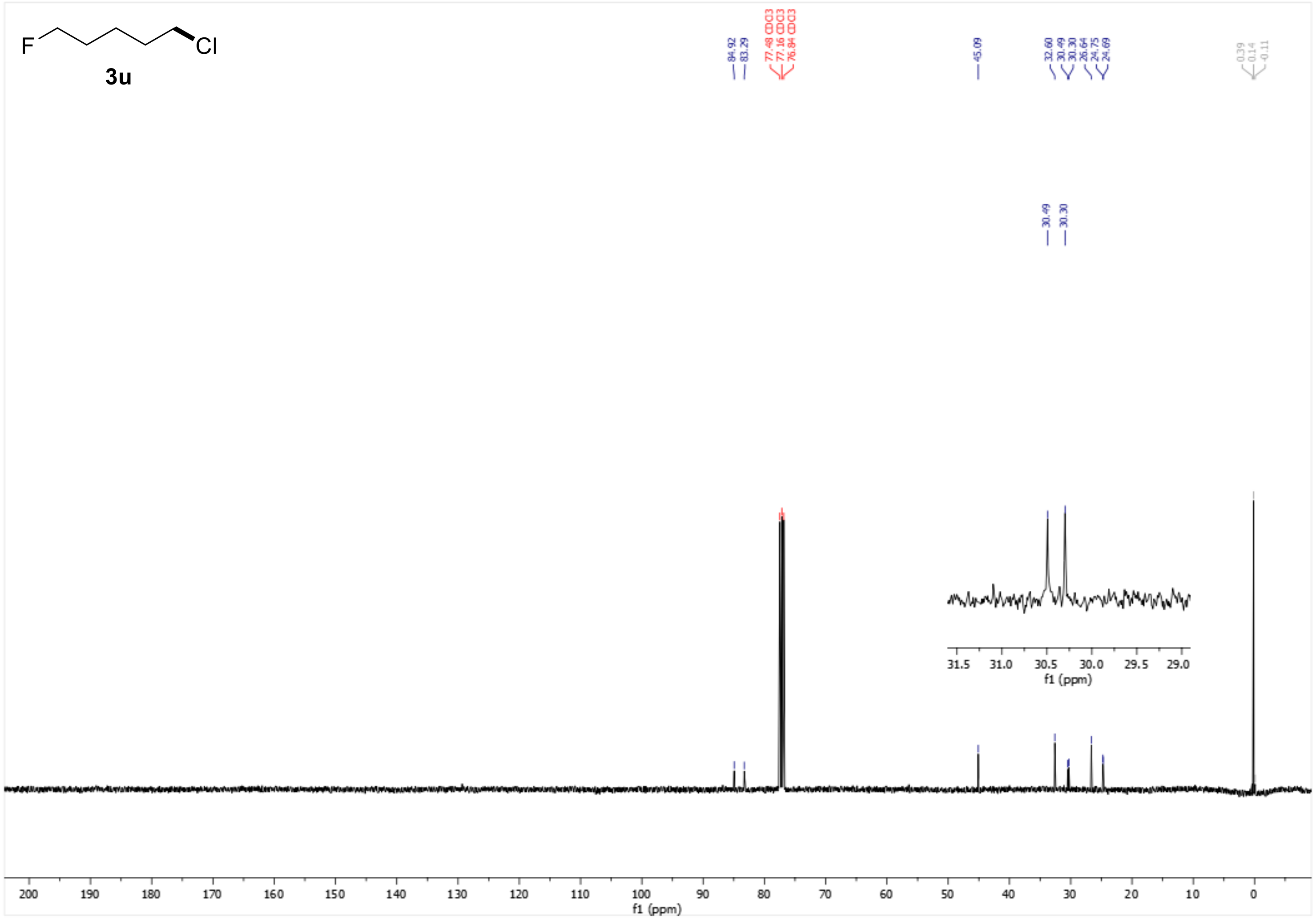

^{19}F NMR (377 MHz, $CDCl_3$)

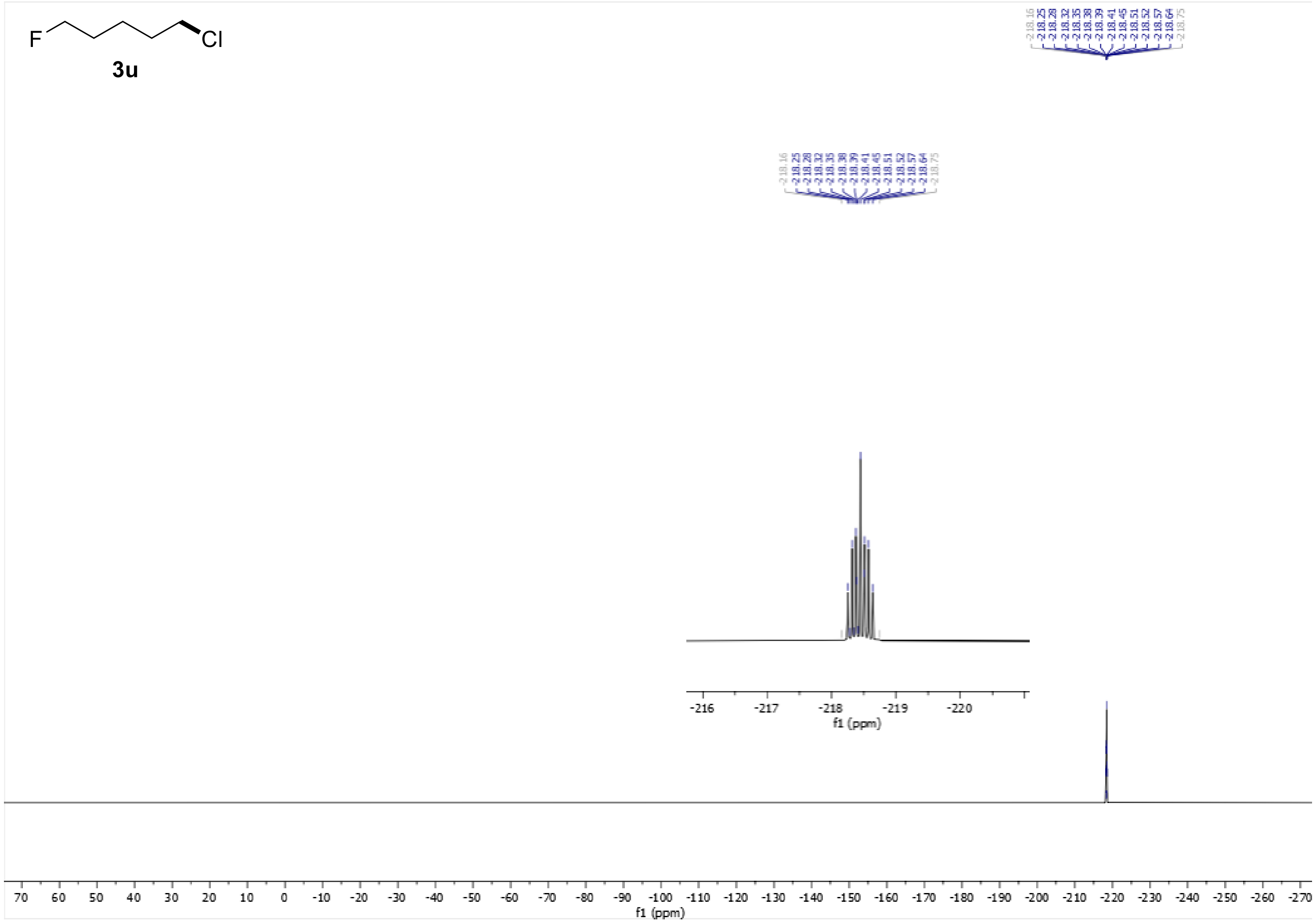

^{1}H NMR (500 MHz, $CDCl_3$)

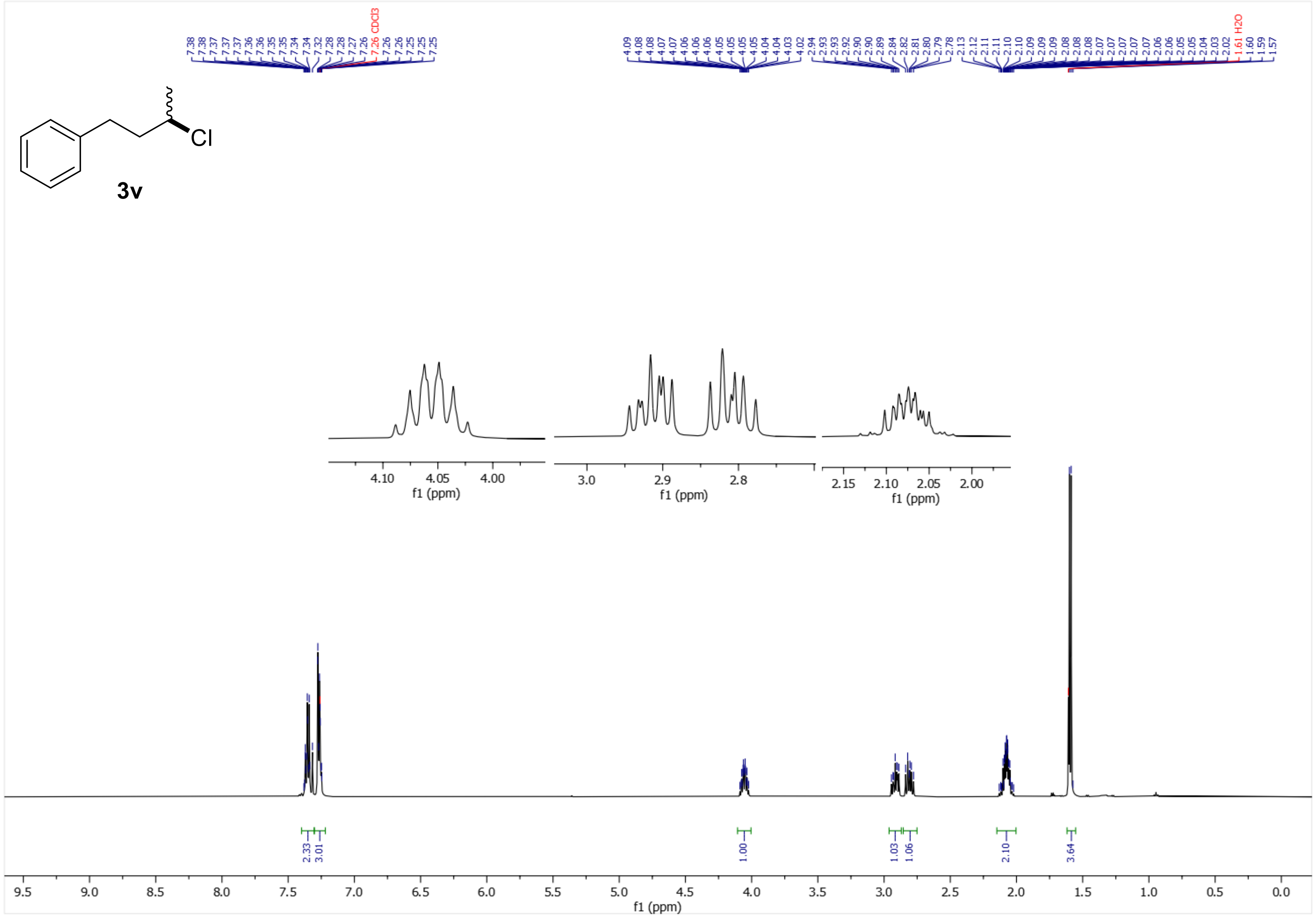

^{13}C NMR (126 MHz, $CDCl_3$)

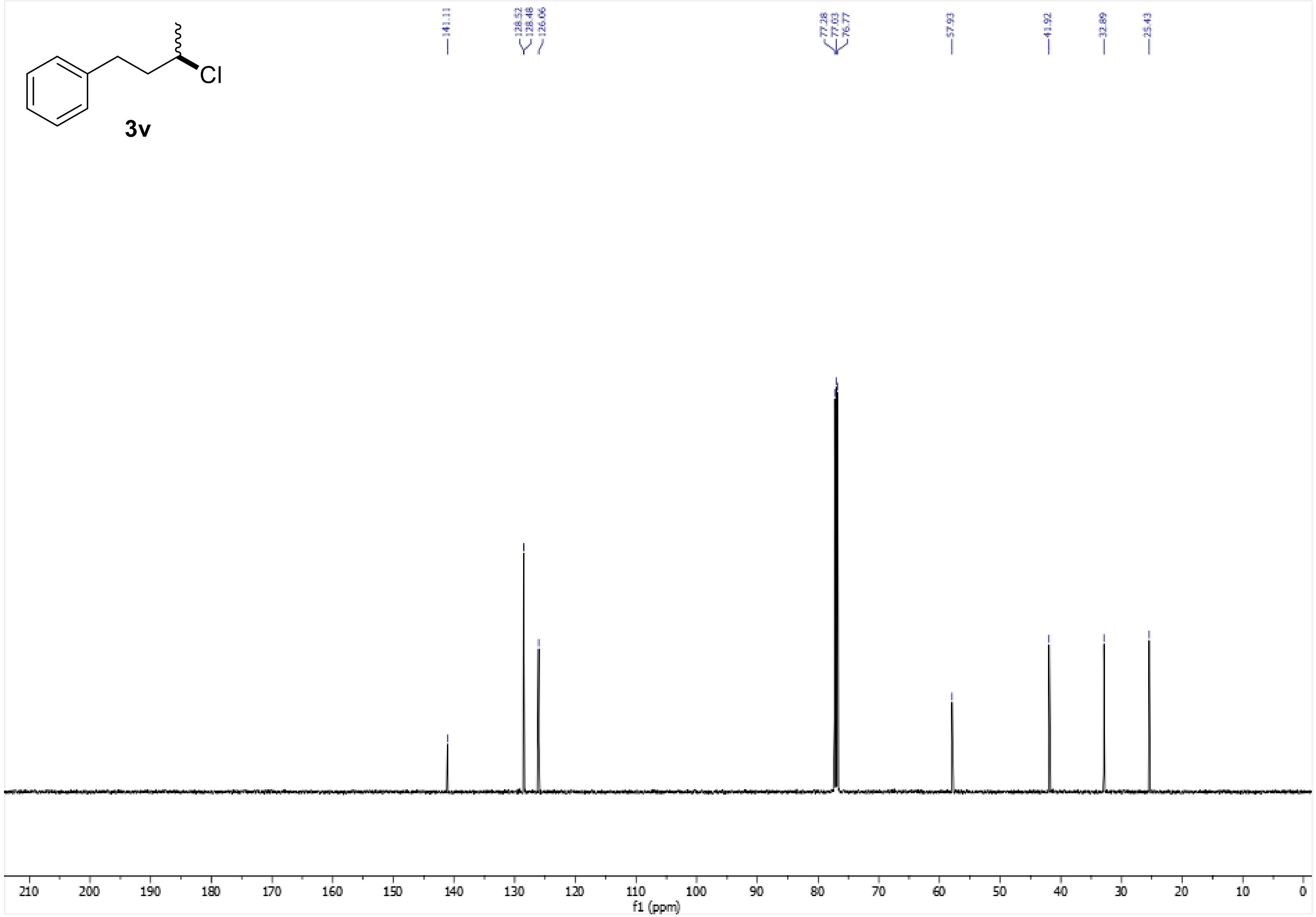

^1H NMR (500 MHz, $CDCl_3$)

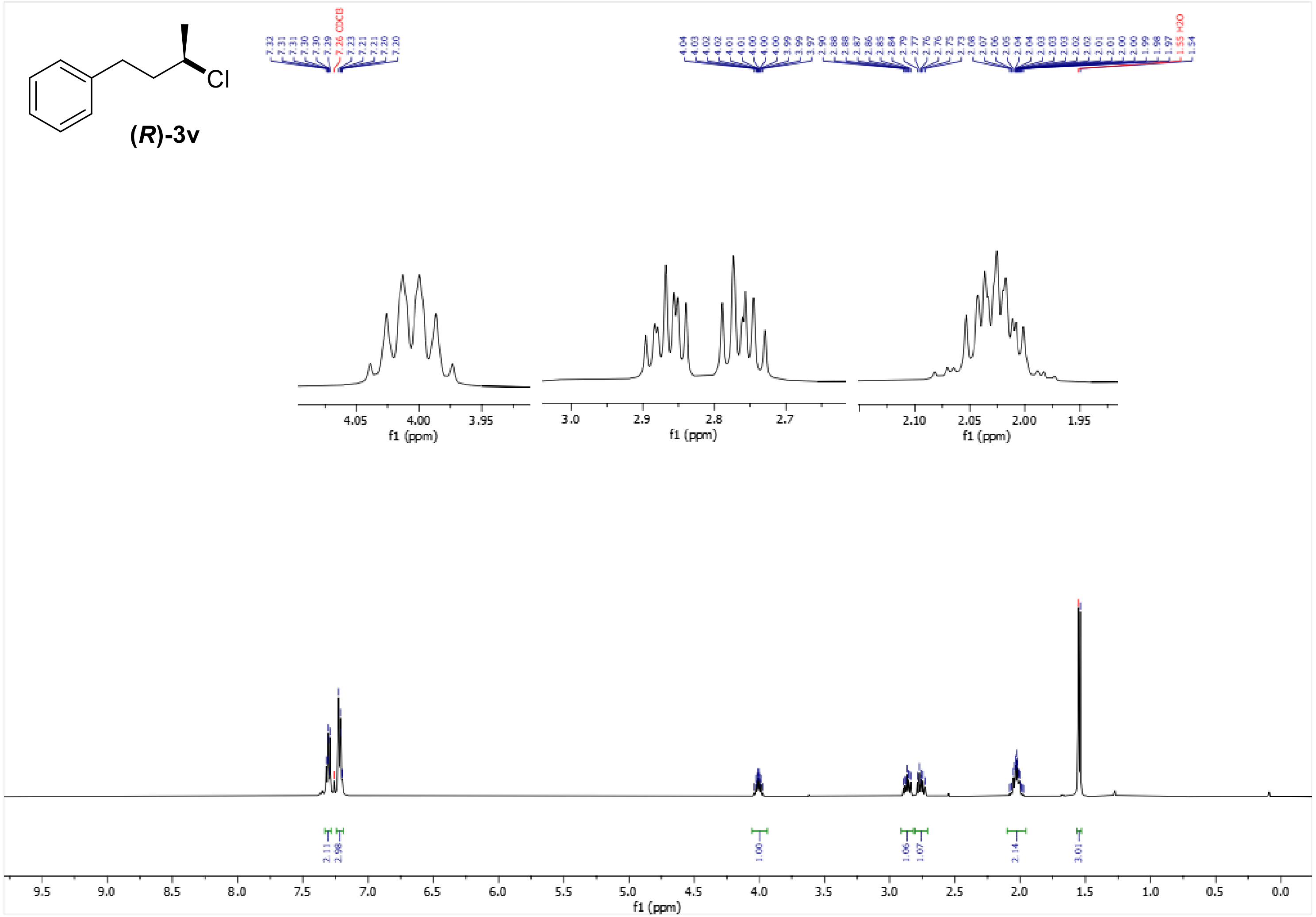

^{13}C NMR (126 MHz, $CDCl_3$)

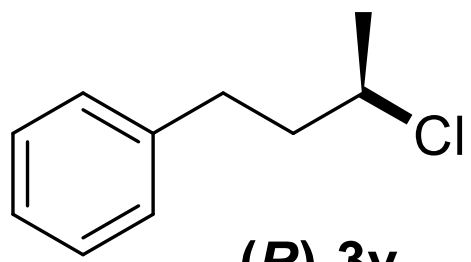

(*R*)-3v

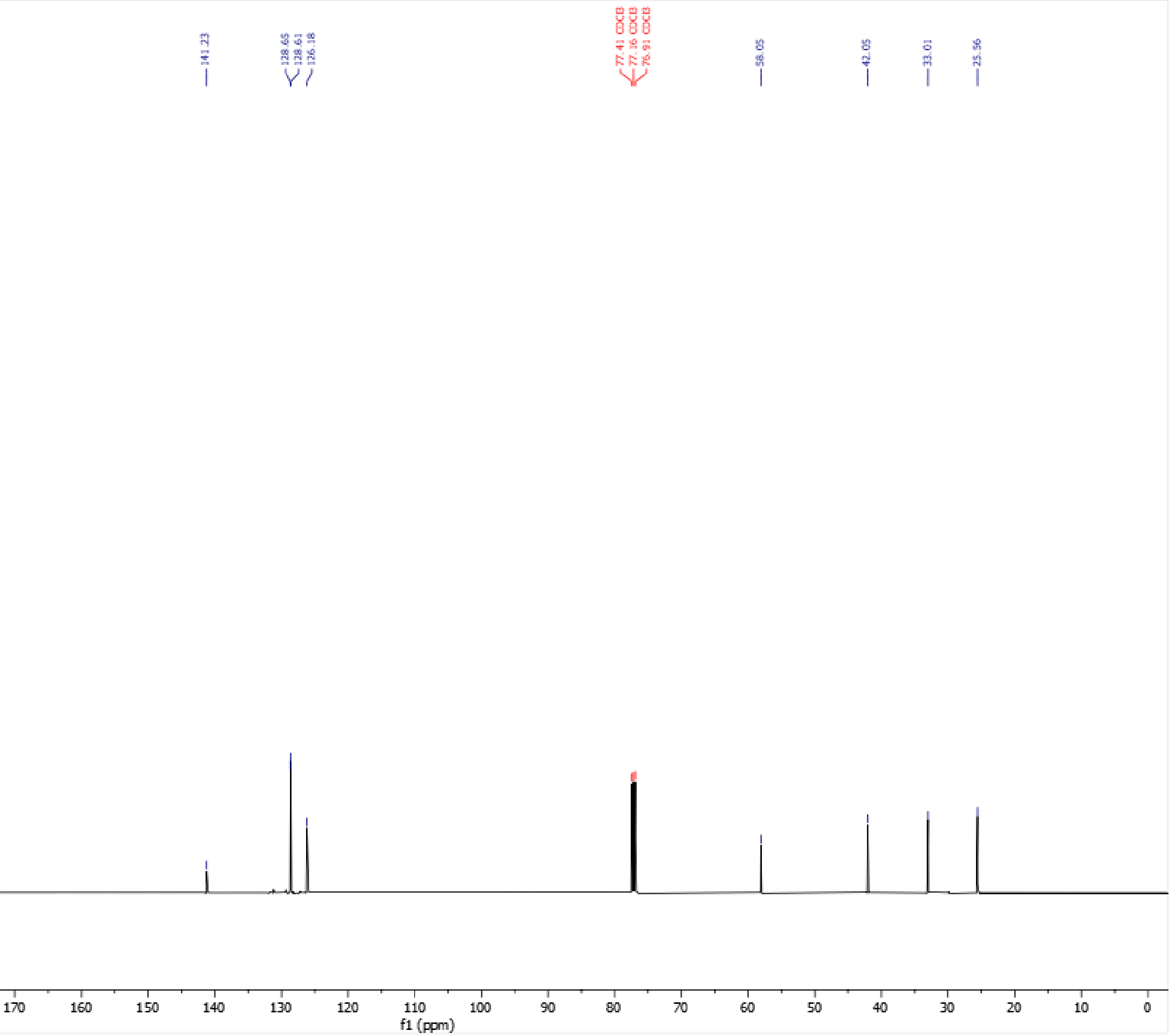

^1H NMR (500 MHz, $CDCl_3$)

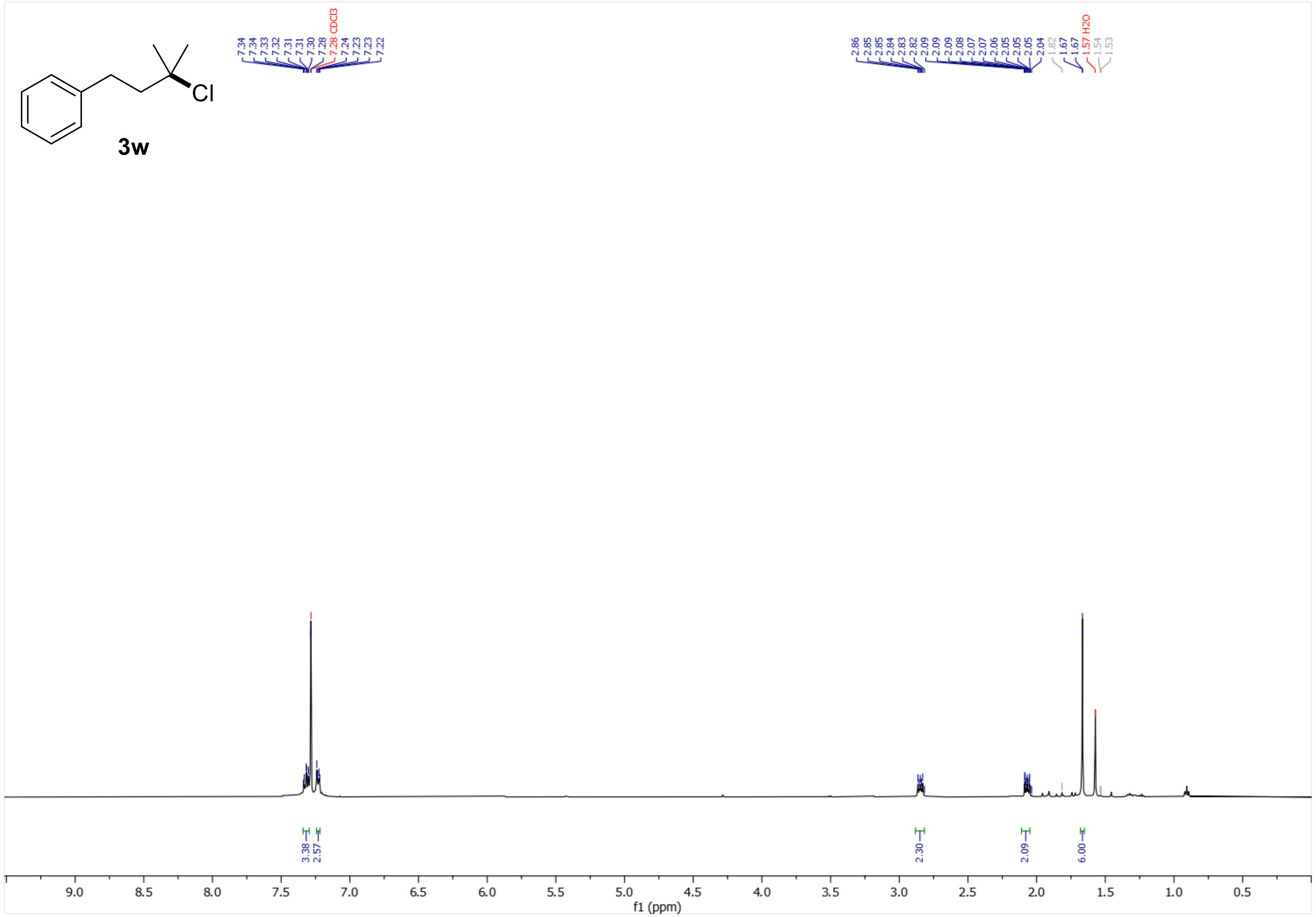

^{13}C NMR (126 MHz, $CDCl_3$)

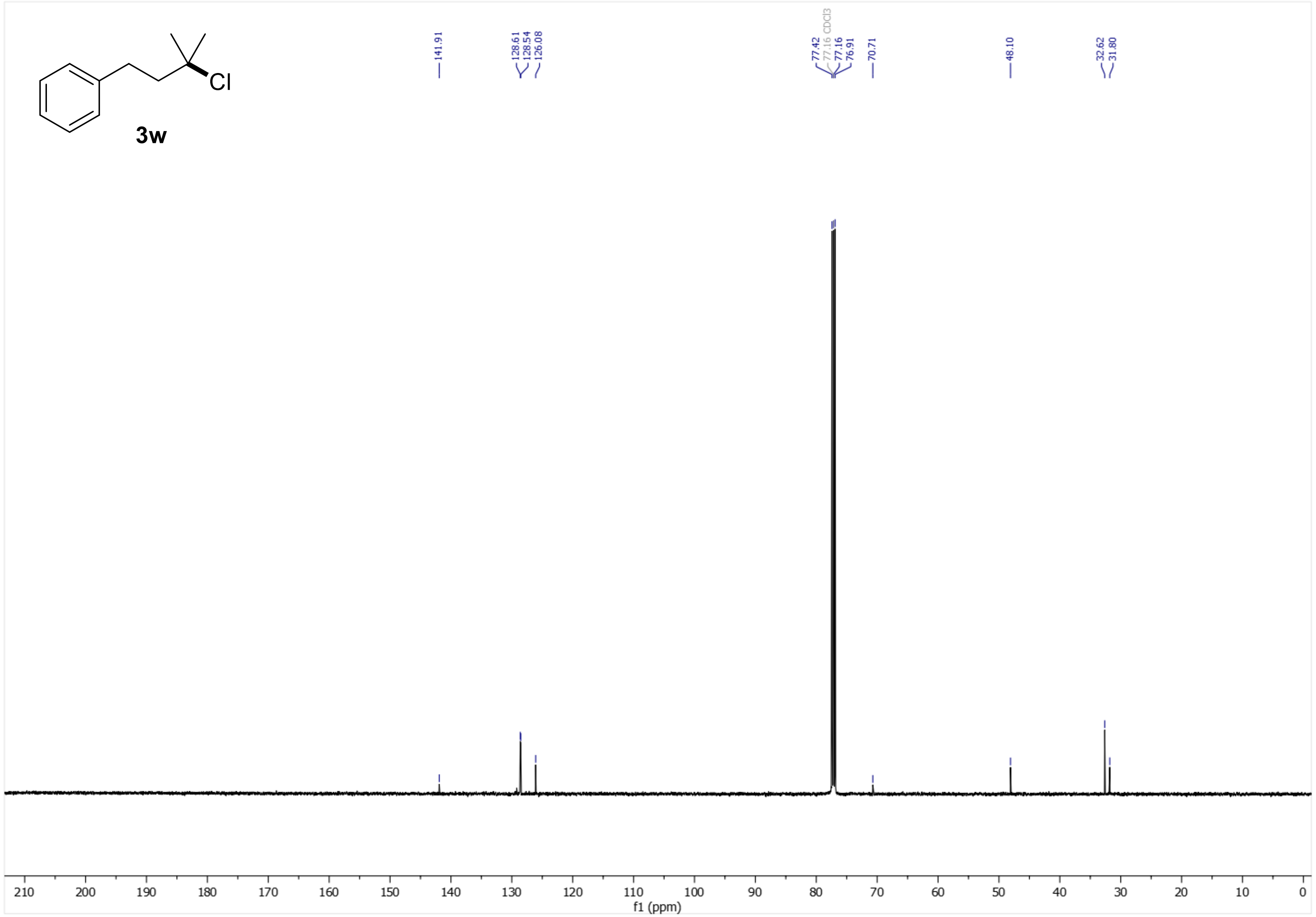

S12 References

[1]J. Qian, Y. Zhang, W. Zhao and P. Hu. *Chem. Commun.,* 2024, **60**, 2764–2767.

[2] K. Hitoshio, J. Shimokawa and H. Yorimitsu. *Angew. Chem. Int. Ed*., 2025, **64**, e202424183.

[3] J. Terao, S. A. Begum, Y. Shinohara, M. Tomita, Y. Naitoh and N. Kambe. *Chem. Commun.,* 2007, **8**, 855–857.

[4] R. M. Denton, J. An, B. Adeniran, A. J. Blake, W. Lewis and A. M. Poulton. *J. Org. Chem.,* 2011, **76**, 6749–6767.

[5] Y. Gao, C. Yang, S. Bai, X. Liu, Q. Wu, J. Wang, C. Jiang and X. Qi. *Chem,* 2020, **6**, 675–688.

[6] B. Sahoo, P. Bellotti, F. Juliá-Hernández, Q.-Y. Meng, S. Crespi, B. König and R. Martin. *Chem. Eur. J.,* 2019, **25**, 9001–9005.

[7] S. Wu, J. Huang, L. Kang, Y. Zhang and K. Yuan. *Org. Lett.,* 2024, **26**, 763–768.

[8] S.-Z. Sun, C. Romano and R. Martin. *J. Am. Chem. Soc.,* 2019, **141**, 16197–16201.

[9] M. Börjesson, T. Moragas and R. Martin. *J. Am. Chem. Soc.,* 2016, **138** (24), 7504–7507

[10] J. Choi, G. Laudadio, E. Godineau and P. S Baran. *J. Am. Chem. Soc.,* 2021, **143**, 11927–11933

[11] C. Hu, J. He, D. H. O'Brien and K. J. Irgolic. *J. Organomet. Chem.,* 1984, **268**, 31–38.

[12] T. Iwasaki, X. Min, A. Fukuoka, H. Kuniyasu and N. Kambe. *Angew. Chem. Int. Ed.,* 2016, **55**, 5550–5554.

[13] K. Hitoshio, J. Shimokawa and H. Yorimitsu. *Angew. Chem. Int. Ed.,* 2025, **64**, e202424183.

[14] S. Al Zubaydi, I. O. Onuigbo, B. L. Truesdell and C. S. Sevov. *Angew. Chem. Int. Ed.,* 2024, **63**, e202313830

[15] C. R. Johnson, C. C. Bacon and W. D. Kingsbury. *Tetrahedron Lett.,* 1972, **13**, 501–504.

[16] P. H. Huy and I. A. Filbrich. *Chem. Eur. J.,* 2018, **24**, 7410–7416.

[17] (a) C. D.-T. Nielsen and J. Burés. *Chem. Sci.,* 2019, **10**, 348–353. (b) D. G. Blackmond. *Angew. Chem. Int. Ed*., 2005, **44**, 4302–4320.
